# Identification and Characterization of MicroRNAs from Longitudinal Muscle and Respiratory Tree in Sea Cucumber (*Apostichopus japonicus*) Using High-Throughput Sequencing

**DOI:** 10.1371/journal.pone.0134899

**Published:** 2015-08-05

**Authors:** Hongdi Wang, Shikai Liu, Jun Cui, Chengze Li, Yucai Hu, Wei Zhou, Yaqing Chang, Xuemei Qiu, Zhanjiang Liu, Xiuli Wang

**Affiliations:** 1 Key Laboratory of Mariculture & Stock Enhancement in North China’s Sea, Ministry of Agriculture, Dalian Ocean University, Dalian 116023, China; 2 The Fish Molecular Genetics and Biotechnology Laboratory, Aquatic Genomics Unit, School of Fisheries, Aquaculture and Aquatic Sciences and Program of Cell and Molecular Biosciences, Auburn University, Auburn, AL 36849, United States of America; 3 School of Science, Dalian Ocean University, Dalian 116023, China; Chinese Academy of Fishery Sciences, CHINA

## Abstract

MicroRNAs (miRNAs), as a family of non-coding small RNAs, play important roles in the post-transcriptional regulation of gene expression. Sea cucumber (*Apostichopus japonicus*) is an important economic species which is widely cultured in East Asia. The longitudinal muscle (LTM) and respiratory tree (RPT) are two important tissues in sea cucumber, playing important roles such as respiration and movement. In this study, we identified and characterized miRNAs in the LTM and RPT of sea cucumber (*Apostichopus japonicus*) using Illumina HiSeq 2000 platform. A total of 314 and 221 conserved miRNAs were identified in LTM and RPT, respectively. In addition, 27 and 34 novel miRNAs were identified in the LTM and RPT, respectively. A set of 58 miRNAs were identified to be differentially expressed between LTM and RPT. Among them, 9 miRNAs (miR-31a-3p, miR-738, miR-1692, let-7a, miR-72a, miR-100b-5p, miR-31b-5p, miR-429-3p, and miR-2008) in RPT and 7 miRNAs (miR-127, miR-340, miR-381, miR-3543, miR-434-5p, miR-136-3p, and miR-300-3p) in LTM were differentially expressed with foldchange value being greater than 10. A total of 14,207 and 12,174 target genes of these miRNAs were predicted, respectively. Functional analysis of these target genes of miRNAs were performed by GO analysis and pathway analysis. This result provided in this work will be useful for understanding biological characteristics of the LTM and RPT of sea cucumber and assisting molecular breeding of sea cucumber for aquaculture.

## Introduction

MicroRNAs (miRNAs) are endogenous non-coding small RNAs with average length of 22 nucleotides, which play important roles in various physiological processes [1]. MiRNAs were initially reported to repress mRNA translation by binding to 3’UTRs of target mRNAs [2]. Later on, studies indicated that miRNAs are also involved in post-transcriptional regulation, and regulate 60% to 90% genes during transcription [3]. The study of miRNAs become a hot topic in numerous organisms in recent years; miRNAs have been sequenced and characterized in a total of 223 species (miRBase v21), including animals [4], plants [5] and even microorganisms [6].

Sea cucumber (*Apostichopus japonicus*) is one of echinoderm species, which is widely distributed along the coasts of Northeast Asia [7]. With great economic value, *A*. *japonicus* was massively cultured in the Asian coasts [8]. Molecular studies of *A*. *japonicas* in unraveling mechanisms of its biological characteristics and disease responses are increasingly conducted. Several miRNA studies in *A*. *japonicus* using the high-through sequencing method were reported in recent years. Li et al. performed small RNA-Seq of healthy and skin-ulceration-syndrome sea cucumbers, in which they found that two miRNAs were significantly differentially expressed between the two libraries [9]. Similarly, Chen et al. performed high-throughput sequencing to identify differentially expressed miRNAs in the intestine between normal and aestivation sea cucumbers. Seven miRNAs differentially expressed between the two libraries were identified, which revealed that miRNAs were involved in the aestivation in sea cucumber [10]. Another studies reported the high-throughput miRNA sequencing of respiratory tree tissue in normal and aestivation sea cucumbers, and revealed that four miRNAs were significantly over-expressed during aestivation in the respiratory tree [11]. A recent study by our group and collaborators reported the identification of significantly up-regulated expression of miRNAs in tube foot of sea cucumber using high-throughput sequencing [12].

Previous studies have indicated that miRNAs were differentially expressed under different physiological conditions and among various tissues. It’s valuable to identify and characterize miRNAs in various important organs. Longitudinal muscle (LTM) is one of the most important movement structures of sea cucumber. In addition, it is the major part of body wall, which constitutes the main product of sea cucumber [7]. Meanwhile, the dieretic longitudinal muscle bands in sea cucumber could be more valuable product than the body wall [13]. The LTM was widely used as a material for studies on the development and differentiation of echinoderm, the gene cloning and expression, molecular mechanisms of muscle contraction [14–18]. Respiratory tree (RTP), another characteristic organ of sea cucumber, is translucent branching diverticula, which performs multiple functions in sea cucumber [19]. It was used as model organ to investigate the regeneration, ultrastructural morphological observation, and immune defense functions [20]. The mechanisms underlying the biological functions of the RPT in sea cucumber draw great interests in recent years. Many genes in the RPT of sea cucumber were cloned and their expressions were determined [21–23]. The transcriptome and miRNAome of RPT in aestivation sea cucumbers were also characterized to identify differentially expressed genes between aestivation and non-aestivation conditions [24,25]. However, to our knowledge, no study on the tissue-specific gene expression analysis in RPT is reported.

In this work, we performed deep sequencing of the LTM and RPT small RNA transcriptome using Illumina HiSeq 2000 platform. The main objectives are to identify and characterize miRNAs from these two important organs, and to identify tissue-specifically expressed miRNAs between these two tissues. This study will be helpful for the further molecular function research in the RPT and LTM of sea cucumber, and will provide valuable genomic resources to assist the molecular breeding of sea cucumber.

## Results

### Deep sequencing of small RNA transcriptome

**Fig 1:**
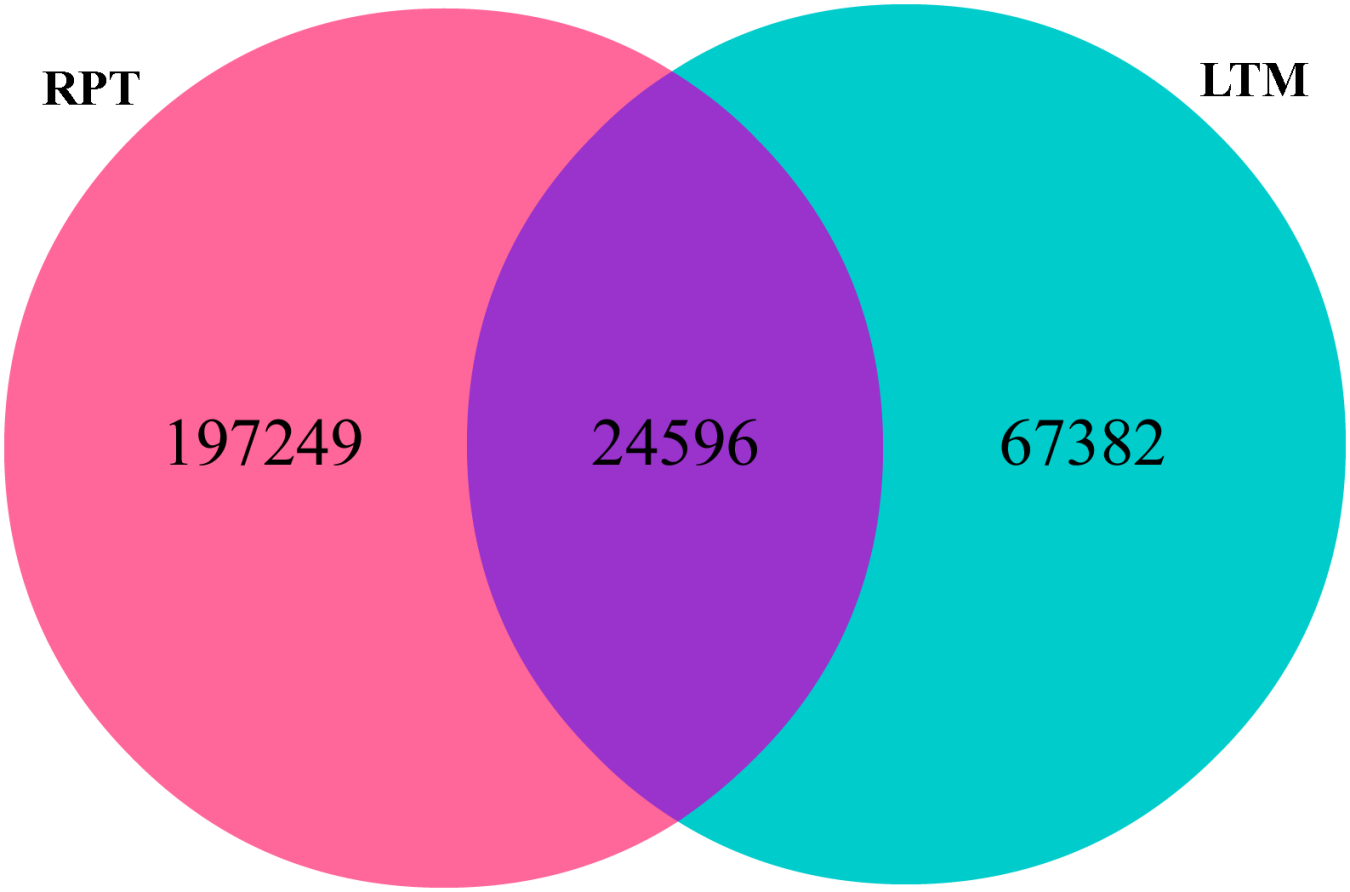
Common and specific unique reads obtained by deep sequencing of small RNA transcriptome in the longitudinal muscle (LTM) and respiratory tree (RPT). The pink denotes the specific unique reads from RPT, the green denotes the specific unique reads from LTM, and the purple denotes the common unique reads obtained from both LTM and RPT.

Deep sequencing of the two small RNA libraries yielded 11,207,408 and 11,032,104 raw reads for LTM and RPT, respectively. After trimming, 11,018,885 and 10,777,407 clean reads were obtained, including 91,978 and 221,845 unique reads for LTM and RPT, respectively (Table 1). The specific and common unique reads between LTM and RPT were identified as shown in Fig 1, which included 24,596 common reads generated from both LTM and RPT, while 67,382 and 197,249 unique reads were generated only from LTM and RPT, respectively.

**Table 1:** Summary of the small RNA transcriptome sequencing of the *A*. *japonicus* longitudinal muscle and respiratory tree.

	Longitudinal muscle		Respiratory Tree	
	Number of sequenced reads	Percentage	Number of sequenced reads	Percentage
***Total reads***	11207408	100%	11032104	100%
***Low quality score***	3522	0.03%	4318	0.04%
***5*** ^***’***^ ***adapter contaminant***	435	0.00%	208	0.00%
***3*** ^***’***^ ***adapter null and insert null***	175469	1.57%	206901	1.88%
***Poly A/T/G/C***	9097	0.08%	43003	0.39%
***N%>10%***	0	0%	267	0.00%
***Clean reads***	11018885	98.32%	10777407	97.69%

**Fig 2:**
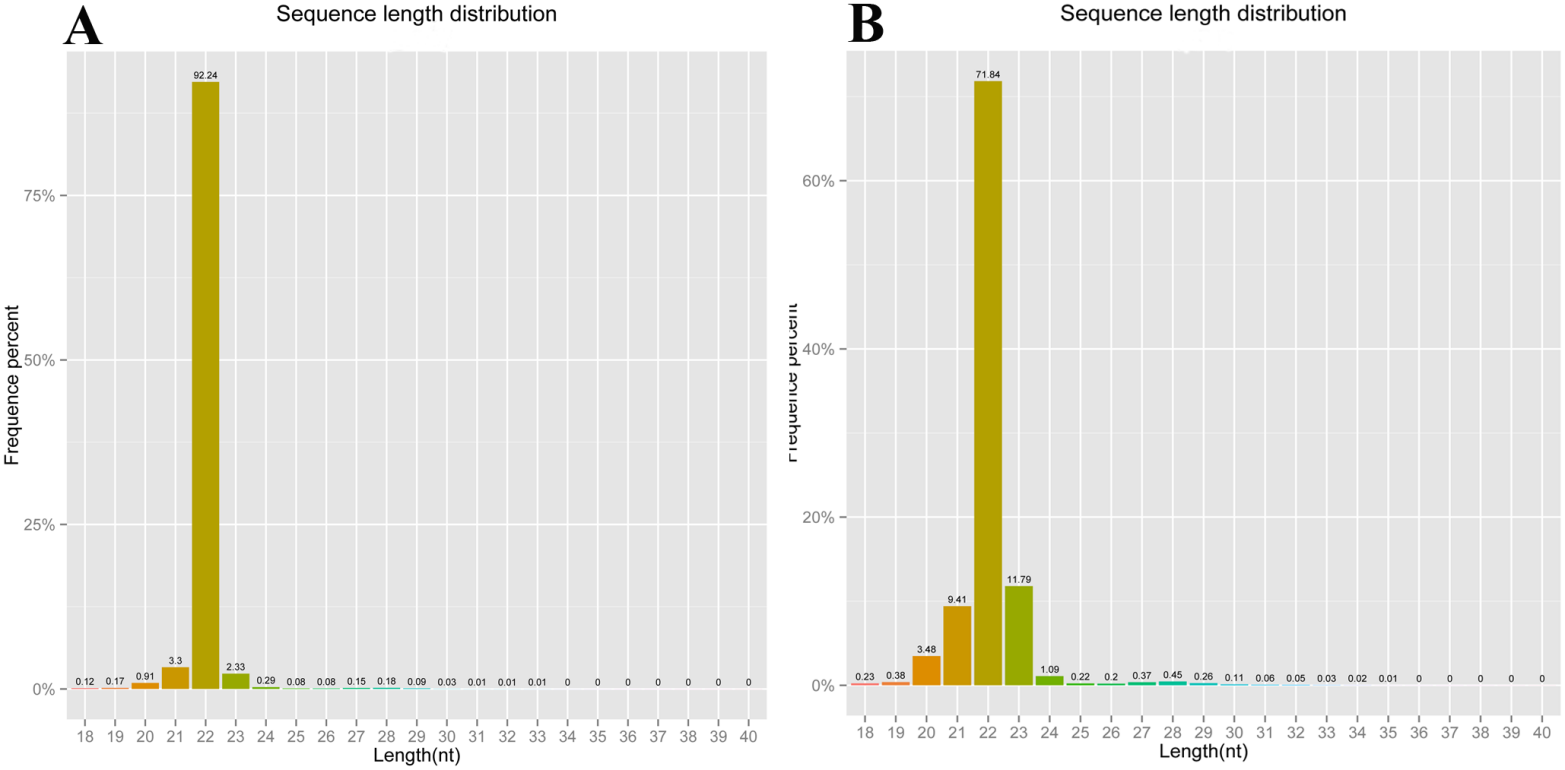
Length distribution of small RNAs identified from the LTM (A) and RPT (B) of sea cucumber (*A*. *japonicus*).

The majority of identified small RNAs were with lengths of 20–23 nt for LTM, and 20–24 nt for RPT, as shown in Fig 2. Small RNAs with length of 22 nt were the most abundant (Fig 2). Based on the annotation with Rfam database, 7,619 small RNAs in the LTM were annotated, including rRNA (6,752), tRNA (733), snRNA (62) and snoRNA (72). Similarly, 28,657 small RNAs in the RPT were annotated, including rRNA (20939), tRNA (7131), snRNA (261) and snoRNA (326). The detailed information of identified small RNAs were provided in Table 2.

**Table 2:** Summary of small RNA annotation.

	Longitudinal muscle		Respiratory Tree	
	Number of sequenced reads	Percentage	Number of sequenced reads	Percentage
***All clean reads***	11,018,885	100.00%	10,777,407	100.00%
***miRNA***	9,765,428	88.62%	8,376,744	77.73%
***rRNA***	6752	0.06%	20,939	0.19%
***tRNA***	733	0.00%	7131	0.07%
***snRNA***	62	0.00%	261	0.00%
***snoRNA***	72	0.00%	326	0.00%
***novel miRNA***	62,749	0.57%	173,590	1.61%
***others***	1,183,089	10.74%	2,198,416	20.40%

**Fig 3:**
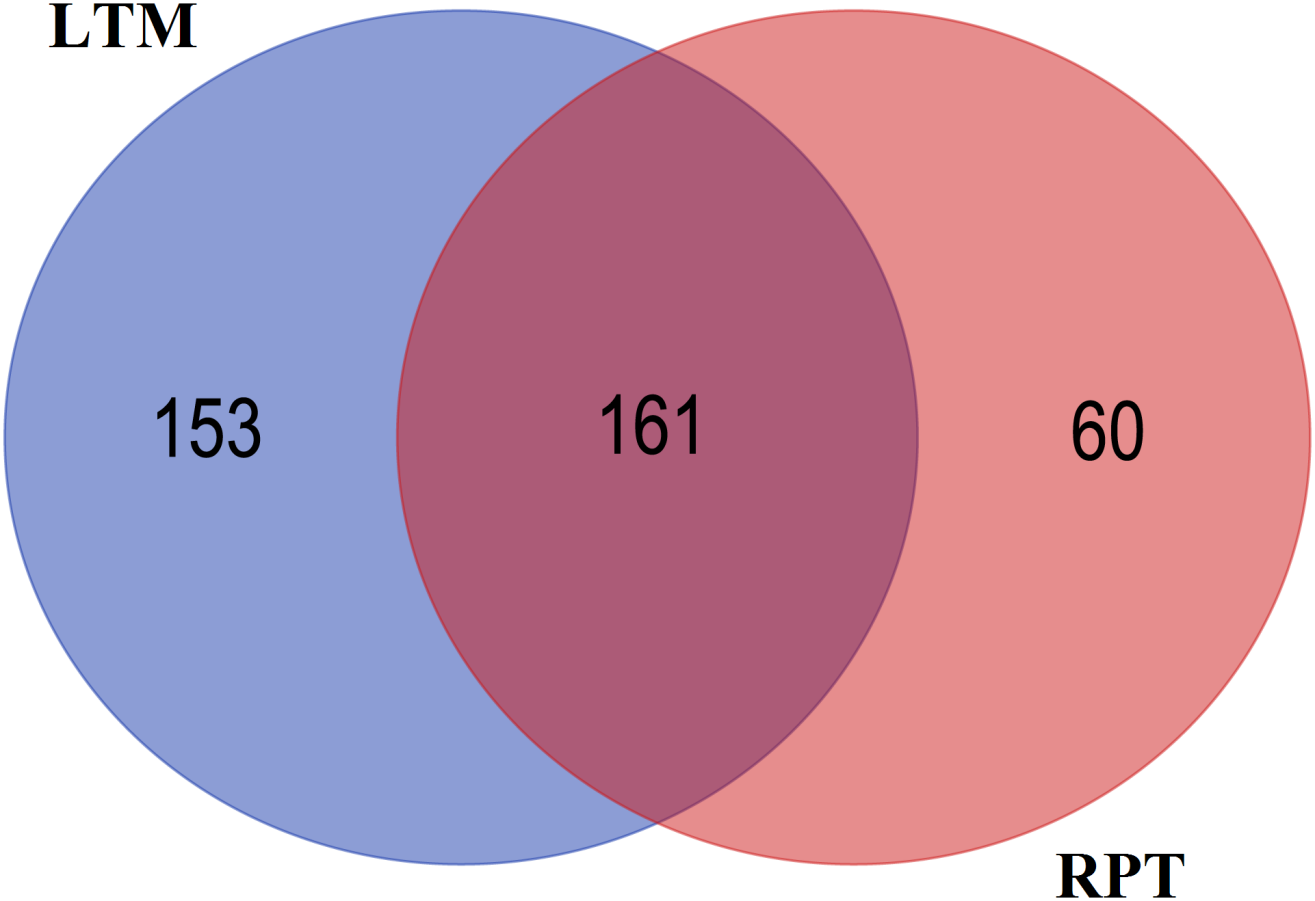
Common and specific conserved miRNAs in the longitudinal muscle (LTM) and respiratory tree (RPT). The blue denotes the specific conserved miRNAs identified from LTM. The pink denotes specific conserved miRNAs identified in RPT, and the purple denotes the common conserved miRNAs in both LTM and RPT.

After removal of reads from other types of small RNAs identified as above, the remaining reads were used miRNA identification. A total of 221 conserved miRNAs were identified in LTM (S1 Table), and a total of 314 small RNAs were identified in RPT (S2 Table). As shown in Fig 3, a total of 161 conserved miRNAs were identified in both tissues, and 153 and 60 miRNAs were only found in LTM or RPT, respectively.

The novel miRNAs were predicted from the remainder of small RNA reads. A total of 27 and 34 novel miRNAs were identified in the LTM and RPT, respectively. The detailed information of the identified novel miRNAs was provied in Table 3 and Table 4. The secondary structures of the novel miRNAs were provided in the additional S1 and S2 Files.

**Table 3:** Novel miRNAs identified from the longitudinal muscle of sea cucumber.

Provisional ID	miRDeep2 score	Read counts	miRNA sequences	Consensus precursor sequence
Scaffold22_17	2.6e+3	5136	ugagguaguagguuauauagu	ugagguaguagguuauauaguuuggagauacacucaauggcgauagcuauacagccugcuagcuuucc
Scaffold759_623	2.5e+3	5003	agcugguaaacgggaaccaaau	agcugguaaacgggaaccaaaucgugaaguaaaugcggauuugguccccuucaaccagccgu
Scaffold912_690	1.7e+3	3527	ucacacacaaccacaggaaguu	cuuucuguggcugucguguuaagugugauucuaugaagacacuucacacacaaccacaggaaguu
Scaffold3711_1014	1.0e+2	201	caaaaaaucacugucggccacug	uggaagacuagugauuuuguuguugugaaugucaaagaacuaacaaaaaaucacugucggccacug
Scaffold2497_965	5.0e+1	97	caucgccaccaaguguacuuca	caucgccaccaaguguacuucaguggacauaugucguuuuuaaaucugagcacacuugguagcggu
Scaffold554_533	3.9e+1	75	cuugugcgugcgacagcgacu	uugcugucacgcggcacaagagagcaaucaugucuauacacucuugugcgugcgacagcgacu
Scaffold652_587	2.5	3210	ccaaggugugcuagugaugaca	cagucacuaccacaccuucgguggcuuuuuauagcuucaccaaggugugcuagugaugaca
Scaffold391_373	2.4	380	uauugcacucgucccggccu	gucgugaggaguugcaauuuguccacaugauaauaaucaucauauugcacucgucccggccu
Scaffold360_346	2.4	203	uugcauagucacaaaagugauu	ucauuuuuguguuuaugcaacuuuuguauuuucaccgaucagaguaguugcauagucacaaaagugauu
Scaffold391_377	2.4	308	uauugcacucgucccggcca	gucaggaugcuugcagugcuguggcuugcucgucuuaugacgagcaacacuaaagcuuucuuaguauugcacucgucccggcca
Scaffold823_655	2.4	72	ugcagcaugauguaguggugu	gccgcuacaucuaguugcauugugacgucuguuucagggaaaugcagcaugauguaguggugu
Scaffold1492_853	2.4	154	uuuguucguucggcucgcguca	gcacgagcucaacgugcaaaacuugagauaaggucagcuguucggcuggccgcuacaugcuguuuuguucguucggcucgcguca
Scaffold22_15	2.3	2488	ucccugagacccuaacuugu	ucccugagacccuaacuugugaugugcuuuuaucaaaucacacagguugguaucucaggaauu
Scaffold391_371	2.2	3404	uauugcacuugucccggccugc	aagucggaccgagcgcaauguuguuccucuauugagguuuuucgaauauugcacuugucccggccugc
Scaffold762_627	2.1	1855	uaauacugucuggugaugau	cauacuggacagcauuggacgugauuggaucguucucuaauacugucuggugaugau
Scaffold111_150	2.1	2935	uaguacuggcauauggacauug	uaguacuggcauauggacauuguuguaauaaucuuacucucaauguucaucuguccguacugcc
Scaffold191_220	2.1	3497	uuacuguugaugucagccccu	ggcugguuucuccaguaauuuguaucuucauuauagcaucaauuacuguugaugucagccccu
Scaffold97_135	2.0	12	aggcaagauguuggcauagc	aggcaagauguuggcauagcugugauuuaaauauuaacccagcugugucuucauacugccauu
Scaffold391_375	1.9	684	uauugcacuugucccggccu	gucgugacucgugcccaauauucaguguuacugcacaucaauauugcacuugucccggccu
Scaffold2335_956	1.8	14	uauuucaggcaguauacu	uauuucaggcaguauacugguaaaggguuuuauuugcaccauucuuaccuguugcuaccugaaauuaau
Scaffold47_56	1.8	14	uauuucaggcaguauacu	uauuucaggcaguauacugguaaaggguuuuauuugcaccauucuuaccuguugcuaccugaaauuaau
Scaffold365_353	1.7	152	uuauugcuugagaauacacgu	agguauucucgagugaauaauacagaaaagccugauguuauugcuugagaauacacgu
Scaffold39_50	1.4	319	ugaauacaucugcugguuu	accaacagguguuauucucaugugugauguacagaucugugaauacaucugcugguuu
Scaffold280_281	1.4	15927	ugaaagacauggguaguga	ugaaagacauggguagugagauuugacuaucacaaaacaaucucacuauucuguuuuucccc
Scaffold285_287	1.4	72	uaaggcacgcggugaaugc	guucacugugauccuugauuuauauucuaaaacaauuaaggcacgcggugaaugc
Scaffold838_659	1.2	3461	uggacggagaacugauaag	uaucauucucuugcccggccgaauacuauguuauugaaauugcgcuggacggagaacugauaag
Scaffold759_626	1.1	9549	uggaauguaaagaaguaug	uacuucuuuagaauuccauacugaaucuccucaacucuauggaauguaaagaaguaug

**Table 4:** Novel miRNA candidates identified from the respiratory tree of sea cucumber.

Provisional ID	miRDeep2 score	Read counts	miRNA sequences	Consensus precursor sequence
Scaffold22_117	6.0e+3	11832	ugagguaguagguuauauagu	ugagguaguagguuauauaguuuggagauacacucaauggcgauagcuauacagccugcuagcuuucc
Scaffold912_2075	3.3e+3	6572	ucacacacaaccacaggaaguu	cuuucuguggcugucguguuaagugugauucuaugaagacacuucacacacaaccacaggaaguu
Scaffold2497_3005	2.9e+3	5745	caucgccaccaaguguacuuca	caucgccaccaaguguacuucaguggacauaugucguuuuuaaaucugagcacacuugguagcggu
Scaffold198_750	3.5e+2	688	ucuuugguuaucuagcuguaug	ucuuugguuaucuagcuguaugagugaugucaaugcaucauaaagcuagguuaccaaagaua
Scaffold3711_3191	3.1e+2	625	caaaaaaucacugucggccacu	uggaagacuagugauuuuguuguugugaaugucaaagaacuaacaaaaaaucacugucggccacu
Scaffold1492_2593	2.4e+2	485	uuuguucguucggcucgcguca	cacgagcucaacgugcaaaacuugagauaaggucagcuguucggcuggccgcuacaugcuguuuuguucguucggcucgcguca
Scaffold360_1160	2.1e+2	414	uugcauagucacaaaagugauu	ucauuuuuguguuuaugcaacuuuuguauuuucaccgaucagaguaguugcauagucacaaaagugauu
Scaffold365_1174	1.5e+2	303	cguauuaacaauguggcugaug	cguauuaacaauguggcugaugaggaaucuuaugaaccaucagccucgcugucaauacga
Scaffold759_1903	1.2e+2	236	agcugguaaacgggaaccaaau	agcugguaaacgggaaccaaaucgugaaguaaaugcggauuugguccccuucaaccagccgu
Scaffold1117_2260	6.1e+1	129	uaacggagaaucaggguucgauu	cugcccuaucaacuuucgaugguacguuaugcgccuaccauggucgucacggguaacggagaaucaggguucgauu
Scaffold518_1542	6.1e+1	129	uaacggagaaucaggguucgauu	cugcccuaucaacuuucgaugguacguuaugcgccuaccauggucgucacggguaacggagaaucaggguucgauu
Scaffold1239_2353	3.1	22	aucacgucgggaucacca	gugaccccgacgugauucgaacacgcagccuucugauuuggagucagaaaguugcguguucggaucacgucgggaucacca
Scaffold652_1761	2.5	6455	ccaaggugugcuagugaugaca	cagucacuaccacaccuucgguggcuuuuuauagcuucaccaaggugugcuagugaugaca
Scaffold391_1237	2.4	342	uauugcacucgucccggccugc	uggucgugaggaguugcaauuuguccacaugauaauaaucaucauauugcacucgucccggccugc
Scaffold391_1241	2.4	106	uauugcacucgucccggcca	gucaggaugcuugcagugcuguggcuugcucgucuuaugacgagcaacacuaaagcuuucuuaguauugcacucgucccggcca
Scaffold823_1975	2.4	23	ugcagcaugauguaguggu	cgcuacaucuaguugcauugugacgucuguuucagggaaaugcagcaugauguaguggu
Scaffold22_115	2.3	1016	ucccugagacccuaacuugu	ucccugagacccuaacuugugaugugcuuuuaucaaaucacacagguugguaucucaggaauu
Scaffold554_1605	2.3	581	cuugugcgugcgacagcgacu	uugcugucacgcggcacaagagagcaaucaugucuauacacucuugugcgugcgacagcgacu
Scaffold391_1235	2.2	5223	uauugcacuugucccggccugc	aagucggaccgagcgcaauguuguuccucuauugagguuuuucgaauauugcacuugucccggccugc
Scaffold191_722	2.1	3737	uuacuguugaugucagccccu	ggcugguuucuccaguaauuuguaucuucauuauagcaucaauuacuguugaugucagccccu
Scaffold111_460	2.1	10733	uaguacuggcauauggacauug	uaguacuggcauauggacauuguuguaauaaucuuacucucaauguucaucuguccguacugcc
Scaffold762_1911	2.1	74312	uaauacugucuggugaugau	cauacuggacagcauuggacgugauuggaucguucucuaauacugucuggugaugau
Scaffold97_413	2.0	4027	aggcaagauguuggcauagc	aggcaagauguuggcauagcugugauuuaaauauuaacccagcugugucuucauacugccauu
Scaffold98_416	2.0	18	aagcgggaucgggacgccgg	ggcguuuaucgaucgccaugcuuacuacugcccgauuggcuuggcaagcgggaucgggacgccgg
Scaffold299_1022	2.0	181	uagcaccaugagaaagcagu	uguuuucuauuggugcauagaguccuuguuuuacuucuucuagcaccaugagaaagcagu
Scaffold391_1239	1.9	529	uauugcacuugucccggccu	gucgugacucgugcccaauauucaguguuacugcacaucaauauugcacuugucccggccu
Scaffold2335_2950	1.8	71	uauuucaggcaguauacu	uauuucaggcaguauacugguaaaggguuuuauuugcaccauucuuaccuguugcuaccugaaauuaau
Scaffold47_222	1.8	71	uauuucaggcaguauacu	uauuucaggcaguauacugguaaaggguuuuauuugcaccauucuuaccuguugcuaccugaaauuaau
Scaffold636_1736	1.7	604	aacccuguagauccgaauu	aacccuguagauccgaauuuguguccgaguuucucgccucugguagucacagauucguaucucuggguaac
Scaffold285_985	1.4	27	uaaggcacgcggugaaugc	guucacugugauccuugauuuauauucuaaaacaauuaaggcacgcggugaaugc
Scaffold39_194	1.4	417	ugaauacaucugcugguuu	accaacagguguuauucucaugugugauguacagaucugugaauacaucugcugguuu
Scaffold280_975	1.4	15771	ugaaagacauggguaguga	ugaaagacauggguagugagauuugacuaucacaaaacaaucucacuauucuguuuuucccc
Scaffold838_1989	1.2	16431	uggacggagaacugauaag	uaucauucucuugcccggccgaauacuauguuauugaaauugcgcuggacggagaacugauaag
Scaffold759_1906	1.1	5735	uggaauguaaagaaguaug	uacuucuuuagaauuccauacugaaucuccucaacucuauggaauguaaagaaguaug

With all the identified miRNAs (conserved and novel miRNAs), a total of 14,207 and 12,174 target genes were predicted in the LTM and RPT, respectively. These target genes were annotated followed by GO analysis (S1 Fig). GO analysis showed that these target genes were involved in a large number of physiological processes at the level 2.

### Analysis of differentially expressed miRNAs

**Fig 4:**
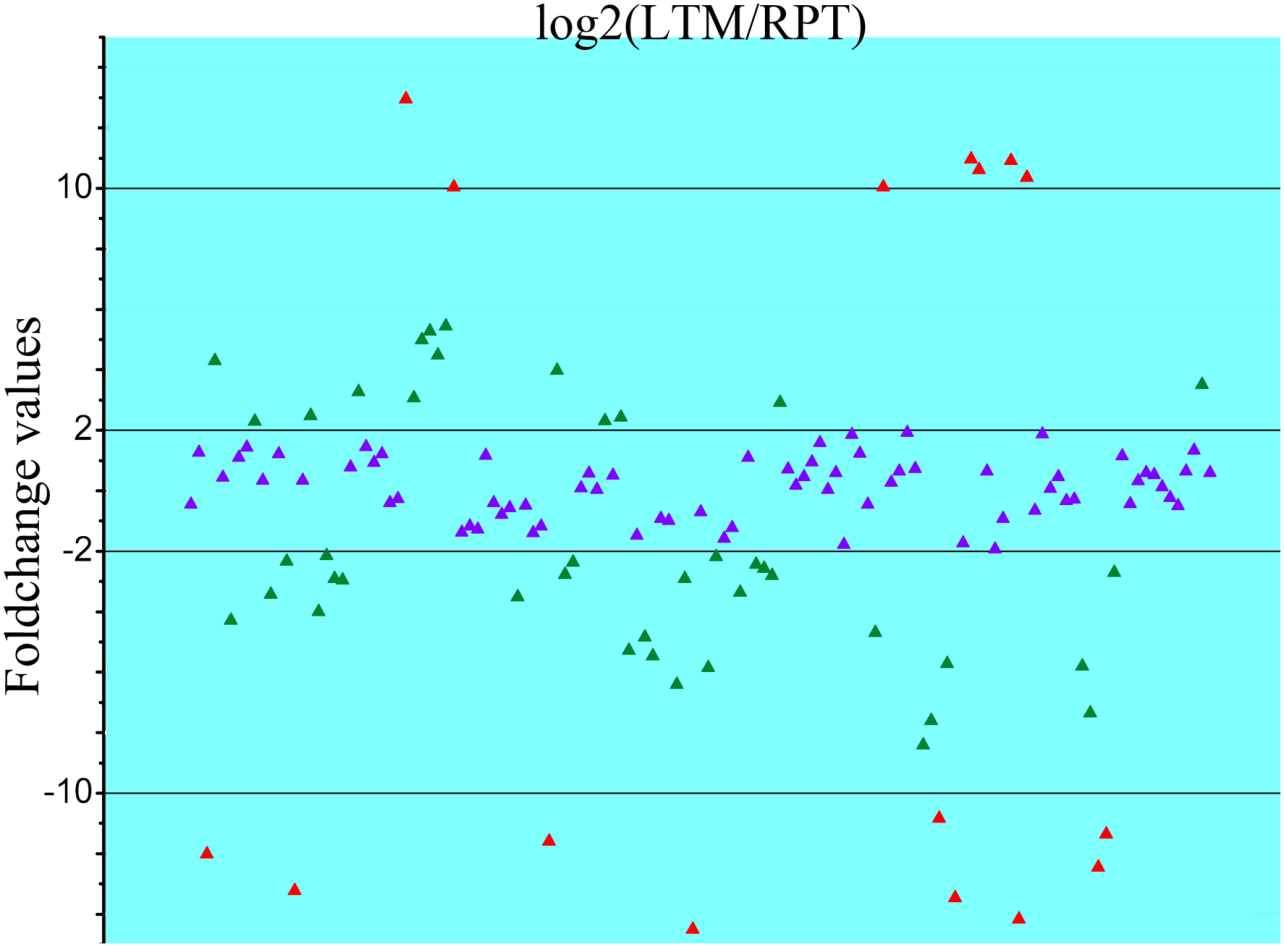
Differential expression of miRNAs between LTM and RPT. miRNAs with foldchanges between -10 and 10 were highlighted in red; foldchanges between -2 and -10 or between 2 and 10 were highlighted in green; and foldchanges between -2 and 2 were highlighted in purple.

A total of 58 miRNAs with foldchange values >2 or <-2 were identified as significantly differentially expressed miRNAs (Fig 4). Seven miRNAs, including miR-127, miR-340, miR-381, miR-3543, miR-434-5p, miR-136-3p, miR-300-3p were expressed at higher levels in LRM (Foldchange values >10). Nine miRNAs, including miR-31a-3p, miR-738, miR-1692, let-7a, miR-72a, miR-100b-5p, miR-31b-5p, miR-429-3p, miR-2008, were expressed at higher level in RPT (Foldchange values <-10).

### qRT-PCR validation

**Fig 5:**
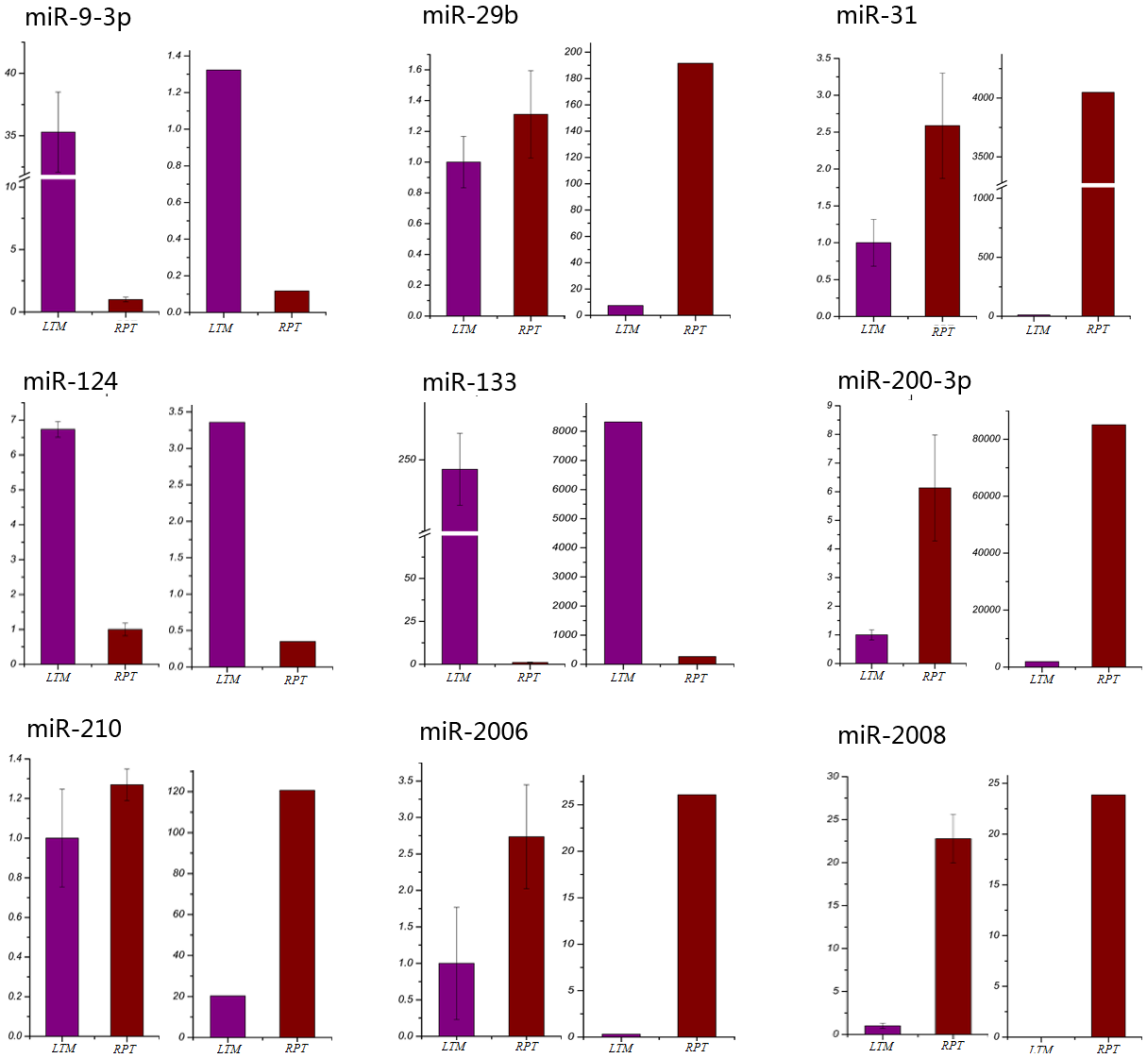
Validation of expression analysis of miRNAs using qRT-PCR. Purple bars indicate the expression in the LTM tissue, while dark red bars indicate the expression in the tissue of RPT.

Nine miRNAs were selected to validate expression analysis by performing qRT-PCR analysis in the LTM and RPT tissues. These included miR-9-3p, miR-29b, miR-31, miR-124, miR-133, miR-200-3p, miR-210, miR-2006 and miR-2008. As shown in Fig 5, the results of qRT-PCR and small RNA-Seq expression analysis for the nine miRNAs in the LTM and RPT were compared, suggesting that high consistence was observed for majority of miRNAs except for miR-29b and miR-210 (Fig 5).

### Functional analysis of significantly differentially expressed miRNAs

**Fig 6:**
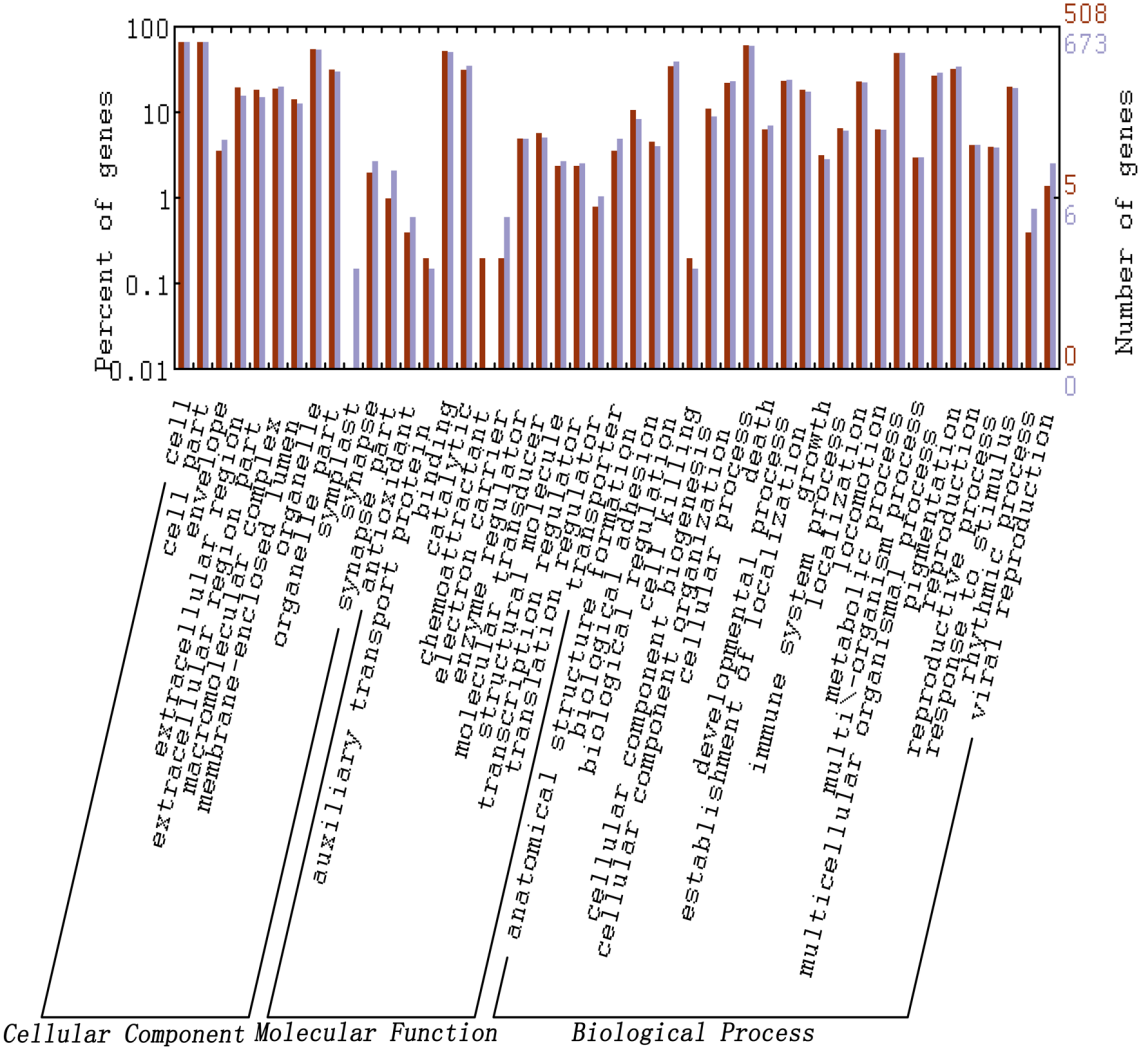
GO analysis for predicted target genes of differentially expressed miRNAs. The dark red bars indicate the gene numbers/percentages within each GO term at level 2 in the LTM, and the grey bars indicated the gene numbers/percentages within each GO term at level 2 in the RPT.

**Fig 7:**
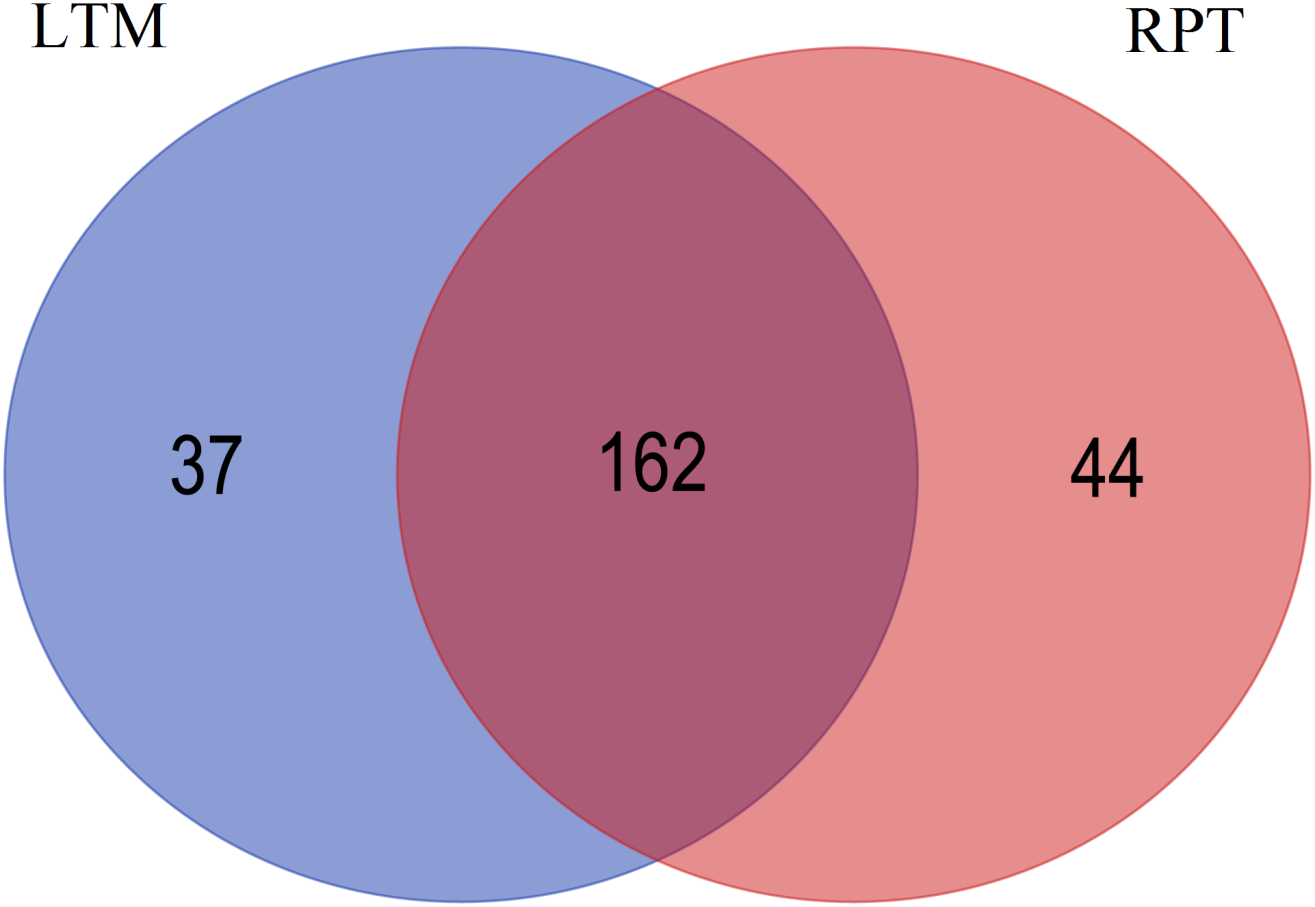
Common and shared target gene KEGG pathways between LTM and RPT. The blue indicates the specific pathways in the LTM, the pink indicates the specific pathways in the RPT, and the dark red indicates the common pathways shared by LTM and RPT.

The target gene prediction for differentially expressed miRNAs with foldchange values >10 or <-10 revealed that 985 and 1,289 genes were predicted in LTM and RPT, respectively. The sequences of these target genes were listed in S3 and S4 Files. GO analysis showed that these genes were involved in numerous processes at GO level 2 (Fig 6). No significant difference in GO terms were observed between LTM and RPT. KEGG analysis indicated that a total of 37 and 44 KEGG pathways were only found in LTM or RPT, respectively. A total of 162 KEGG pathways were shared by LTM and RPT (Fig 7). The details information of KEGG pathways were provided in S3 Table.

## Discussion

Longitudinal muscle and respiratory tree are two of the important organs in sea cucumber. Generation of genomic resources, such as miRNAs, is essential for studying the biological roles of the longitudinal muscle and respiratory tree in sea cucumber. In this study, we performed deep sequencing small RNAs in the two tissues, identified miRNAs, and determined their differential expression profiles between the two tissues

Over 11 million short expressed reads were generated for the two tissues, respectively. Comparing to the data generated in previous studies [10–12], similar throughput of datasets were generated in this work, providing sufficient high quality data for data analysis. A total of 91,978 unique reads and 221,845 unique were identified from LTM and RPT, respectively. The dramatic differences in read numbers between LTM and RPT may suggest the potential differences in miRNA complexity, consistent with the observation that RPT is involved in more biological roles with much more complex molecular regulation than the LTM [26,27].

The small RNA length distribution showed that 20-24nt was the main length in both the LTM and RPT, which is the typical size range of miRNAs. This is consistent with other miRNA studies in echinoderm animals [12,28–30]. A total of 221 and 314 conserved miRNAs were identified from the RPT and LTM, of which 161 miRNAs were identified from both RPT and LTM, while 153 and 60 miRNAs were only identified from LTM and RPT, respectively. The miRNAs identified from specific tissues could play some specific roles in LTM or RPT, respectively.

The most abundant miRNAs were miR-1c, miR-10, and miR-71c-5p in the LTM, while the most abundant miRNAs were miR-1c, miR-10, and miR-200-3p in the RPT. This observation was consistent with previous studies [9,10,12], in which miR-1c and miR-10 were reported to be highly expressed. Taken together, it’s clear that miR-1c and miR-10 were the top two abundant miRNAs in the various tissues of sea cucumber examined to date.

Due to the lack of sequenced genome of sea cucumber, the genome assembly of purple sea urchin [31], the closest species to *A*. *japonicas*, was used to assist the identification of novel miRNAs in *A*. *japonicas*. A total of 27 and 34 novel miRNAs were identified in the LTM and RPT. Notably, some novel miRNAs may remain unidentified due to the divergence between sea cucumber and sea urchin classes [30].

The expression analysis identified 58 miRNAs that were differentially expressed between LTM and RPT, including nine miRNAs that were expressed at much higher levels (foldchange >10) in LTM and seven miRNAs in RPT. The nine miRNAs that were highly expressed in the RPT included miR-31a-3p, miR-738, miR-1692, let-7a, miR-72a, miR-100b-5p, miR-31b-5p, miR-429-3p, and miR-2008. In rat, miR-31a-3p could be up-regulated after the spinal cord injury, indicating that miR-31a-3p could participate in the spinal cord repair [32]. RPT, as an important respiratory organ in sea cucumber, delivers oxygen and releases carbon-dioxide. The nerve cell in RPT could be injured by some toxins exists in aquaculture seawater [33]. The miR-31a-3p is likely to be involved in the repair process in RPT. MiR-738 was only identified in fish species such as common carp and zebrafish. MiR-738 was found in the common fish immune organ, indicating that it could play important roles in the immune system [34,35]. Let-7a, as one of important member of let-7 family, has been found to perform protective roles for some cardiovascular diseases [36,37], suggesting that highly expressed let-7a in the RPT can function to repair the RPT tissues when the RPT tissue was injured by some harmful materials in sea water. MiR-2008 was associated with powdery mildew infection in wheat [38], which was induced after infection. MiR-2008 was observed to be differentially expressed between skin ulceration syndrome and health sea cucumbers [9,39], suggesting that miR-2008 was involved in the immune response. The function of other up-regulated miRNAs in RPT, including miR-1692, miR-72a, miR-100b-5p and miR-429-3p, were not reported in any species. The regulation pattern and specific functions of these miRNAs deserves further study in the future, especially in the RPT of sea cucumber.

Seven miRNAs highly expressed in the LTM included miR-127, miR-340, miR-381, miR-3543, miR-434-5p, miR-136-3p, and miR-300-3p. MiR-127 is a multi-function miRNA that has been widely investigated [40]. MiR-127 has been found to play important roles in the development and regulation of lung, liver, genital cell and immune system [41–53]. A study in rat found that miR-127 could negatively regulate the bone mass [44], suggesting that miR-127 could take part in the spicule development in sea cucumber. Studies of miR-340 have widely performed in human diseases, but few in sea cucumber. MiR-340 was reported to be involved in regulating the signaling progresses [45,46], suppressing the tumor growth [47,48] and playing important roles in the heart failure diseases [49]. However, function of miR-340 in muscle tissue was not examined yet, which requires further studies in the future. miR-381 could inhibit transcripts of several genes, such as LRRC4 [50,51], WEE1 [52], MDR1[53] and SMARCB1[54], which all associated with different kinds of tumors. The homologs of these target genes may exist and be functional in sea cucumber, therefore, the regulation mechanism between miR-381 and its target genes could be used for the future study in the LTM of sea cucumber. miR-434-5p was reported to mediate the whitening and lightening of the human and rat skin and it can be used as a new additives in the skin caring productions [55]. This is important to sea cucumber production because skin associated health care issue is one of major concerns, and miR-434-5p could provide a way for health caring [56]. miR-136-3p was reported binding the LHR directly to luteinizing hormone receptor (LHR) to down-regulate LHR mRNA in human, while it was highly expressed after human chorionic gonadotropin (hCG) [57]. Notably, many anticancer miRNAs were found in the LTM of sea cucumber. MiRNAs in food could target and regulate gene transcription through food intake [58]. Therefore, it’s speculated that humans may acquire anticancer miRNAs by taking sea cucumber as food, which may be related to the anticancer effects of sea cucumber [59], though the underlying molecular processes remain largely unexplored.

## Materials and Methods

### Ethics statement

All procedures involving the handling and treatment of sea cucumber during this study were approved by the Animal Care and Use committee of Key Laboratory of Mariculture & Stock Enhancement in North China’s Sea at Dalian Ocean University.

### Animals and RNA extraction

Healthy sea cucumbers with weight of 180g-200g were provided by the Key Laboratory of Mariculture in North China (Dalian, Liaoning). These cucumbers were kept in the seawater aquaria without feeding at 18–20°C for 10 days before sampling. Two tissues including longitudinal muscle (LTM) and respiratory tree (RPT) were dissected from each individual and stored in RNAlater (Ambion®). The samples were kept at 4°C for 24 hours and then transferred to -80°C freezer until RNA extraction. Total RNA of the LTM and RPT were extracted using Trizol reagent (Takara, Dalian) according to the manufacturer’s instruction. The quality of total RNA was determined using RNA Nano 6000 Assay Kit of the Agilent Bioanalyzer 2100 system (Agilent Technologies, CA, USA).

### Deep sequencing and Data Analysis

Small RNA sequencing and data analysis was as described in the previous study [12]. Two small RNA sequencing libraries (one per each tissue) were constructed and sequenced using the Illumina HiSeq 2000 platform.

The raw data were trimmed to get the clean and high quality reads by removing reads with low sequencing quality scores, less than 18 nt in length, with 5’ adapters but lost 3’ adapters, and with low complexity sequences. Clean reads with length of 18–40 nt were used for further analyses. The length distribution and the unique reads were analyzed. The specific and common unique reads were also be analyzed. The clean reads were searched against the Rfam database (http://rfam.sanger.ac.uk/) to annotate tRNA, rRNA, snoRNA and snRNA. The conserved miRNA of sea cucumber were identified by searching against all metazoan species known miRNAs from miRbase 21.0 database. Only miRNAs with the perfect matches were considered as the conserved miRNAs. The method and the criteria to identify novel miRNAs were described as in the previous study [12].

The raw datasets of small RNA-Seq used in this study have been deposited to NCBI sequence read archive (SRA) with the accession numbers of SRA242867 (longitudinal muscle) and SRA242842 (respiratory tree).

### Target gene prediction and analysis

The 3’-UTRs and 5’-UTRs extracted from the sea cucumber transcriptome assembly [60] were used as the candidate database to predict the target genes of all miRNAs [12] using MiRanda-3.3 [61]. The predicted target genes were aligned to the annotated result of Du et al [60] to get the annotation information of the predicted target genes. The Gene Ontology (GO) analysis of the annotated predicted target genes was performed using bioDBnet and WEGO [62,63].

### Differentially expressed miRNA analyses and qRT-PCR validation

To identify the differentially expressed miRNAs between the LTM and RPT, the short reads of the conserved miRNAs were first normalized in transcripts per million (TPM) followed by the method of Chi [64]. The fold change of differential expression was determined according to the follow formula:

$$Foldchange=\log_{2}\frac{TPM_{LTM}}{TPM_{RPT}}$$

Where TPM_LTM_ indicates the transcripts per million of miRNAs identified in the LTM tissue, while TPM_RPT_ indicates the transcripts per million of miRNAs identified in the RPT tissue. MiRNAs which had Foldchange values >2 or <-2 were considered as significantly differentially expressed miRNAs.

To validate the data analysis results, a number of differentially expressed miRNAs were randomly selected to perform qRT-PCR. Stem-loop RT-PCR for miRNA [65] and qRT-PCR were performed as describe previously [12]. *Cytb* gene was used as reference gene during the qRT-PCR. The information of all primers used for stem-loop RT-PCR and qRT-PCR were provided in S4 Table. The result of qRT-PCR was calculated using the 2^-ΔΔCt^ method [66].

### Functional analyses of differentially expressed miRNAs

To ensure the accurate functional analysis of differentially expressed miRNAs, only miRNAs with foldchange greater than 10 were used for the analysis. The target genes were predicted using miRanda-3.3, the functional analysis was performed as mentioned above. In addition, the KEGG pathways which contain the target genes were selected. The different KEGG pathways between LTM and RPT were identified.

## Supporting Information

S1 Fig GO analysis result at level 2 for all the identified miRNAs. (TIF)

S1 File The secondary structures of the novel miRNAs in LTM. (ZIP)

S2 File The secondary structures of the novel miRNAs in RPT. (ZIP)

S3 File The miRNAs’ target gene sequences in LTM. (ZIP)

S4 File The miRNAs’ target gene sequences in RPT. (ZIP)

S1 Table Detail information of LTM conserved miRNA. (XLS)

S2 Table Detail information of RPT conserved miRNA. (XLS)

S3 Table Details information of different expressional miRNAs’ KEGG pathways. (XLS)

S4 Table Information of all primers used for stem-loop RT-PCR and qRT-PCR. (XLS)
