# Supplementary figures and images for: Identification and Characterization of MicroRNAs from Longitudinal Muscle and Respiratory Tree in Sea Cucumber (Apostichopus japonicus) Using High-Throughput Sequencing
Source: PLoS One. 2015 Aug 5;10(8):e0134899. doi: 10.1371/journal.pone.0134899 (PMC4526669; doi:10.1371/journal.pone.0134899)

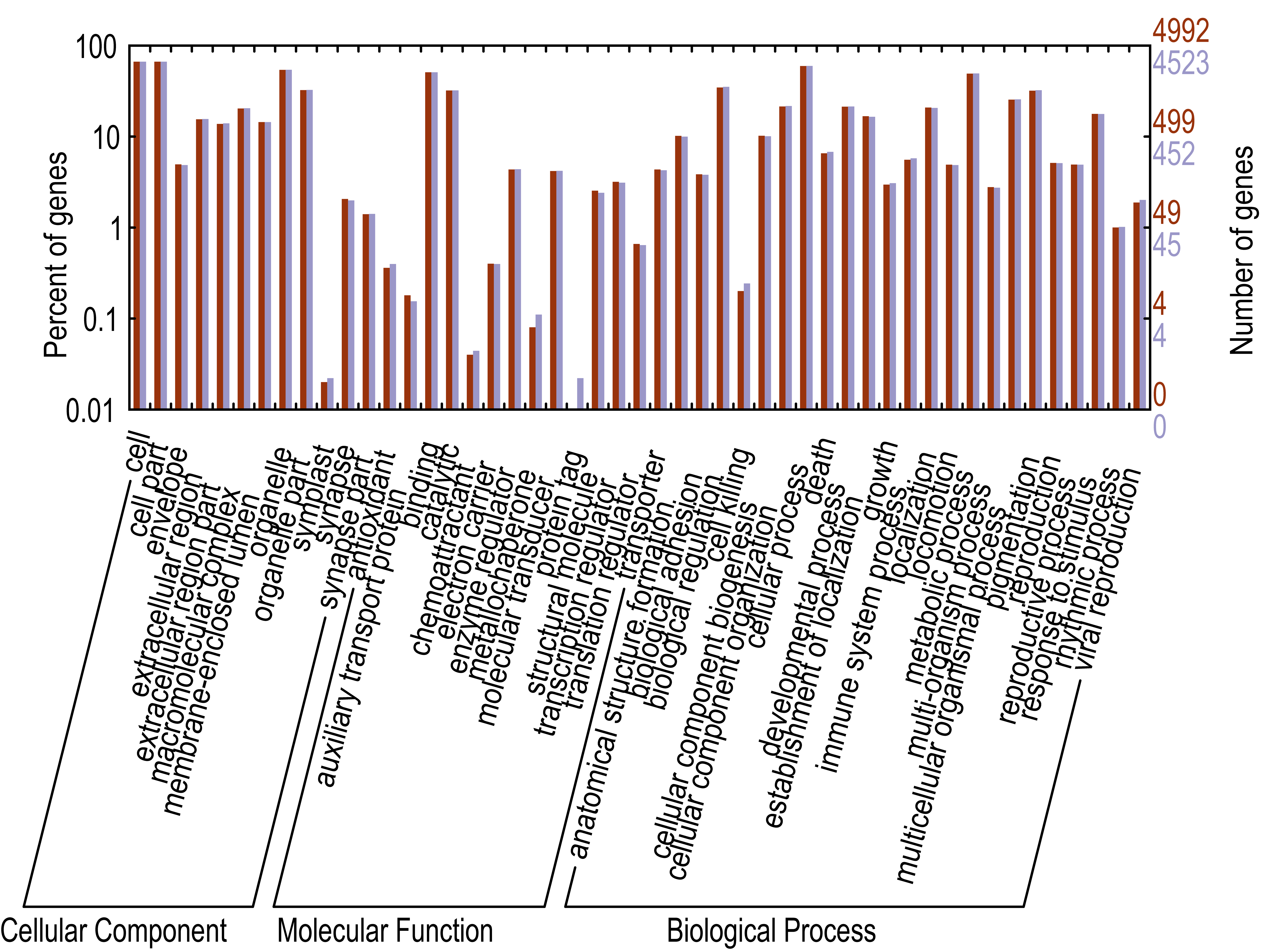

Supplement: S1 Fig — (TIF) [file pone.0134899.s001.tif]

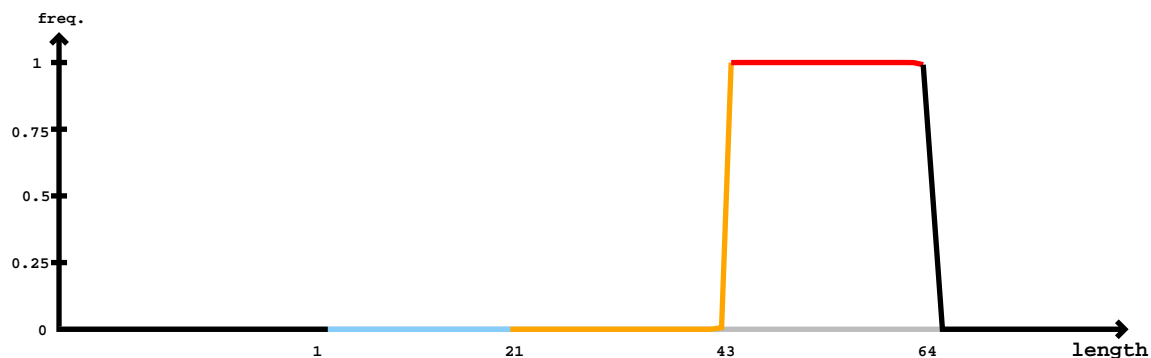

## Mature

[illegible]

Supplement: S1 File — (ZIP) [file pone.0134899.s002.zip › S1 File/The secondary structures of the novel miRNAs in LTM/Scaffold191_220.pdf]

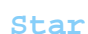[illegible]

Supplement: S1 File — (ZIP) [file pone.0134899.s002.zip › S1 File/The secondary structures of the novel miRNAs in LTM/Scaffold2335_956.pdf]

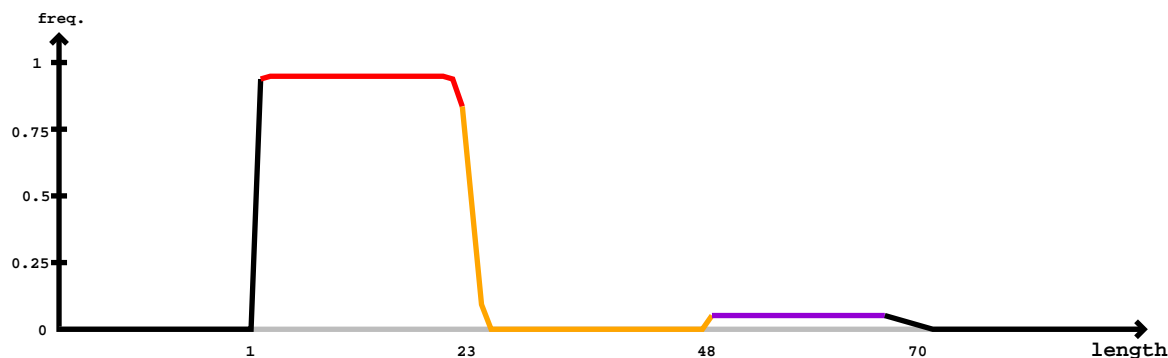

Star

[illegible]

Supplement: S1 File — (ZIP) [file pone.0134899.s002.zip › S1 File/The secondary structures of the novel miRNAs in LTM/Scaffold2497_965.pdf]

[illegible]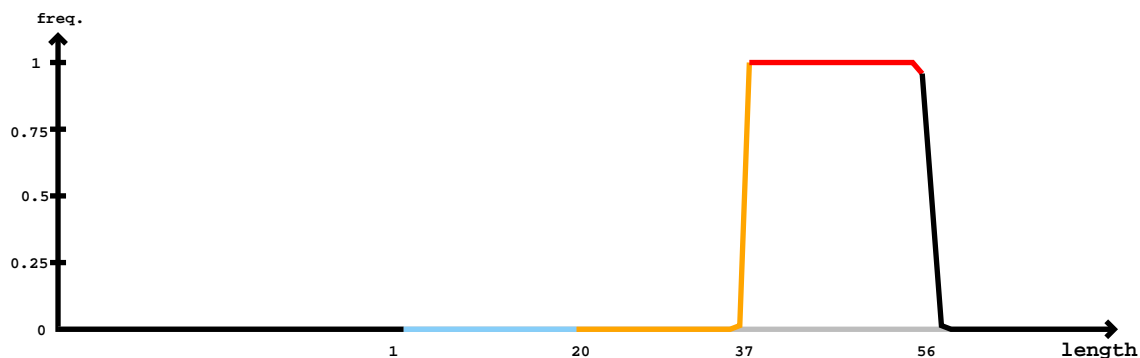

## Mature

[illegible]

Supplement: S1 File — (ZIP) [file pone.0134899.s002.zip › S1 File/The secondary structures of the novel miRNAs in LTM/Scaffold285_287.pdf]

[illegible]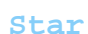

Supplement: S1 File — (ZIP) [file pone.0134899.s002.zip › S1 File/The secondary structures of the novel miRNAs in LTM/Scaffold391_377.pdf]

[illegible]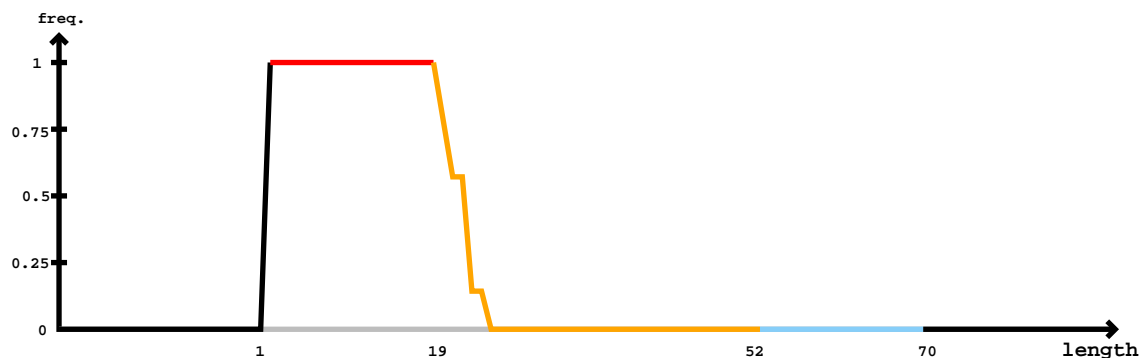

Star

[illegible]

Supplement: S1 File — (ZIP) [file pone.0134899.s002.zip › S1 File/The secondary structures of the novel miRNAs in LTM/Scaffold47_56.pdf]

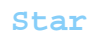[illegible]

Supplement: S1 File — (ZIP) [file pone.0134899.s002.zip › S1 File/The secondary structures of the novel miRNAs in LTM/Scaffold97_135.pdf]

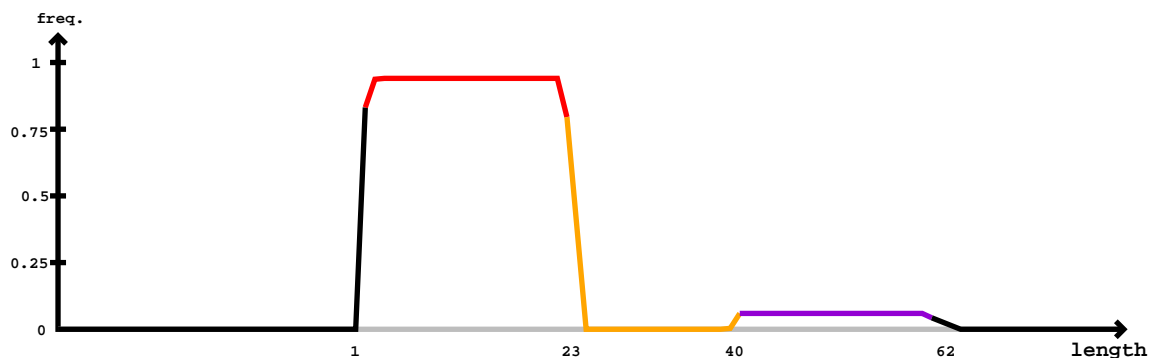

Star

[illegible]

Supplement: S2 File — (ZIP) [file pone.0134899.s003.zip › S2 File/The secondary structures of the novel miRNAs in RPT/Scaffold365_1174.pdf]

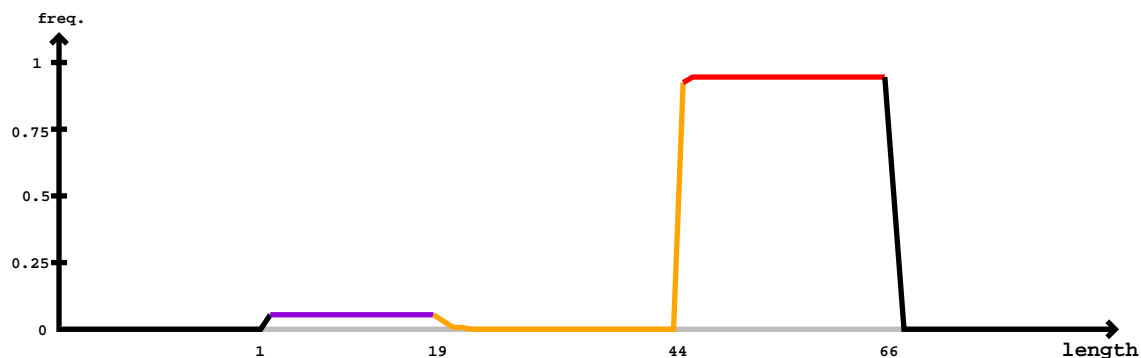

## Mature

[illegible]

Supplement: S2 File — (ZIP) [file pone.0134899.s003.zip › S2 File/The secondary structures of the novel miRNAs in RPT/Scaffold3711_3191.pdf]

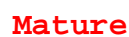[illegible]

Supplement: S2 File — (ZIP) [file pone.0134899.s003.zip › S2 File/The secondary structures of the novel miRNAs in RPT/Scaffold39_194.pdf]

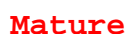[illegible]

Supplement: S2 File — (ZIP) [file pone.0134899.s003.zip › S2 File/The secondary structures of the novel miRNAs in RPT/Scaffold98_416.pdf]
