# Supplementary material for: Identification and Characterization of MicroRNAs from Longitudinal Muscle and Respiratory Tree in Sea Cucumber (Apostichopus japonicus) Using High-Throughput Sequencing
Source: PLoS One. 2015 Aug 5;10(8):e0134899. doi: 10.1371/journal.pone.0134899 (PMC4526669; doi:10.1371/journal.pone.0134899)
Supplement: S1 File — (ZIP) [file pone.0134899.s002.zip › S1 File/The secondary structures of the novel miRNAs in LTM/Scaffold111_150.pdf]

Provisional ID	:	Scaffold111_150
Score total	:	2.1
Score for star read(s)	:	-1.3
Score for read counts	:	0
Score for mfe	:	1.8
Score for randfold	:	1.6
Score for cons. seed	:	
Total read count	:	2935
Mature read count	:	2935
Loop read count	:	0
Star read count	:	0

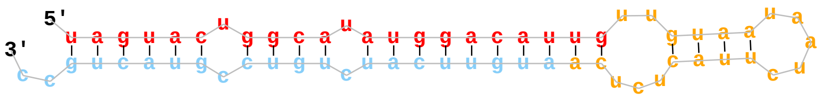

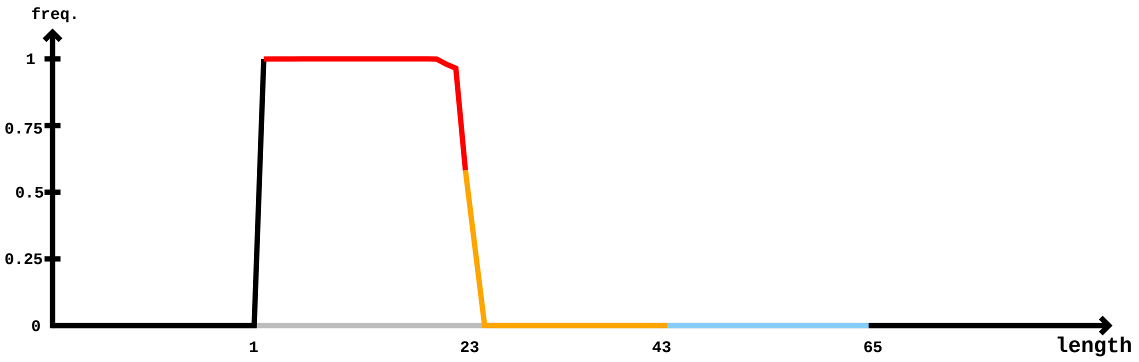

Mature Star

```
5'- acacauugcuggguucuugauaguacuggcauauggacauuguuguaauaaucuuacucucaauguucaucuguccguacugccaagugccuggccuuacauucauuc  -3'   exp
    .......(((((((.((((.((((((.((((.((((((((((..((((.....))))...)))))))))).)))).)))))).)))).))))))).............   reads   mm   sample
    ....................uaguacuggcauauggaU......................................................................   1       1    seq
    ....................uaguacuggcauauggac......................................................................   1       0    seq
    ....................uaguacuggcauauggaca.....................................................................   49      0    seq
    ....................uaguacuggcauauggacU.....................................................................   4       1    seq
    ....................uaguacuggcauUuggaca.....................................................................   1       1    seq
    ....................uaguacuggUauauggaca.....................................................................   1       1    seq
    ....................uaguacuggcauGuggaca.....................................................................   1       1    seq
    ....................uaguacuggcauaCggacau....................................................................   1       1    seq
    ....................uaguacuggcauauggGcau....................................................................   3       1    seq
    ....................uagCacuggcauauggacau....................................................................   1       1    seq
    ....................uaguacuggcauauggacaC....................................................................   21      1    seq
    ....................uGguacuggcauauggacau....................................................................   2       1    seq
    ....................uagGacuggcauauggacau....................................................................   4       1    seq
    ....................uaguacuggcauauggacGu....................................................................   1       1    seq
    ....................uaguacAggcauauggacau....................................................................   1       1    seq
    ....................uaguacuggcauauggacaG....................................................................   2       1    seq
    ....................uaguacugAcauauggacau....................................................................   2       1    seq
    ....................uaguacCggcauauggacau....................................................................   2       1    seq
    ....................uaguacuggcauauggacaA....................................................................   4       1    seq
    ....................uaguacuggcaCauggacau....................................................................   1       1    seq
    ....................uaguacGggcauauggacauu...................................................................   17      1    seq
    ....................uagCacuggcauauggacauu...................................................................   62      1    seq
    ....................uaguacuggcauauggacaCu...................................................................   22      1    seq
    ....................uaguacuggAauauggacauu...................................................................   1       1    seq
    ....................uagGacuggcauauggacauu...................................................................   149     1    seq
    ....................uaguacuAgcauauggacauu...................................................................   25      1    seq
    ....................uaguacugAcauauggacauu...................................................................   14      1    seq
    ....................uaguacuUgcauauggacauu...................................................................   3       1    seq
    ....................uaguacugUcauauggacauu...................................................................   1       1    seq
    ....................uaguacuggcauauCgacauu...................................................................   5       1    seq
    ....................uUguacuggcauauggacauu...................................................................   10      1    seq
    ....................uaguacuggcaAauggacauu...................................................................   2       1    seq
    ....................uaguacuggcauauggaUauu...................................................................   16      1    seq
    ....................uaguacuggcauaAggacauu...................................................................   10      1    seq
```

Mature Star

acacauugcugguucuugauaguacuggcauauggacauuguuguaauaaucuuacucucaauguucaucuguccguacugccaagugccuggccuuacauucauuc			
...................uaguacuCgcauauggacauu...	2	1	seq
...................uaguacuggcauauggCcauu...	5	1	seq
...................uaguaUuggcauauggacauu...	9	1	seq
...................uaguacuggcauauggGcauu...	77	1	seq
...................uaguUcuggcauauggacauu...	6	1	seq
...................uaguacuggcauauggUcauu...	4	1	seq
...................uaguacuggcauauggacUuu...	3	1	seq
...................uaguaGuggcauauggacauu...	3	1	seq
...................uaguacuggcCuauggacauu...	2	1	seq
...................uaguacuggcauaugCacauu...	3	1	seq
...................uaCuacuggcauauggacauu...	2	1	seq
...................uaguacuggcauauUgacauu...	6	1	seq
...................uaguacuggcUuauggacauu...	4	1	seq
...................uaUuacuggcauauggacauu...	71	1	seq
...................uaguacuggcauaugAacauu...	21	1	seq
...................uCguacuggcauauggacauu...	3	1	seq
...................uaguacuggcGuauggacauu...	62	1	seq
...................uaguacuggcauauAgacauu...	26	1	seq
...................uaguacuggcaGauggacauu...	3	1	seq
...................uagAacuggcauauggacauu...	29	1	seq
...................uaguacuggcauGuggacauu...	27	1	seq
...................uaAuacuggcauauggacauu...	14	1	seq
...................uaguacuggcauUuggacauu...	12	1	seq
...................uaguacAggcauauggacauu...	20	1	seq
...................uaguacuggcaCauggacauu...	33	1	seq
...................uaguacuggcauauggacaGu...	12	1	seq
...................uaguacuggcauauggacaAu...	3	1	seq
...................uaguacuggUauauggacauu...	38	1	seq
...................uaguacuggcauauggacGuu...	63	1	seq
...................uaguacCggcauauggacauu...	41	1	seq
...................uaguCcuggcauauggacauu...	1	1	seq
...................uaguacuggcauauggaGauu...	2	1	seq
...................uaguacuggGauauggacauu...	1	1	seq
...................uaguacuggcauaugUacauu...	3	1	seq
...................uaguacuggcauauggacCuu...	5	1	seq
...................uGguacuggcauauggacauu...	62	1	seq
...................uaguacuggcauaGggacauu...	4	1	seq
...................uaguacuggcauaCggacauu...	38	1	seq
...................uaguaAuggcauauggacauu...	1	1	seq
...................uaguGcuggcauauggacauu...	64	1	seq
...................uaguaUuggcauauggacauug..	28	1	seq
...................uaguacuggcGuauggacauug..	95	1	seq
...................uagAacuggcauauggacauug..	41	1	seq
...................uaguacuggcauCuggacauug..	3	1	seq
...................uaguacuggcauaugUacauug..	1	1	seq
...................uaguacugUcauauggacauug..	7	1	seq
...................uaguaAuggcauauggacauug..	4	1	seq
...................uaguacuAgcauauggacauug..	27	1	seq
...................uaguacuggcauauCgacauug..	4	1	seq
...................uaguacugAcauauggacauug..	19	1	seq
...................uaguacuggcauauggacGuug..	98	1	seq
...................uaAuacuggcauauggacauug..	28	1	seq
...................uaguacuggcaAauggacauug..	3	1	seq
...................uaguacuggcauGuggacauug..	39	1	seq
...................uaguacuggcauauggUcauug..	13	1	seq
...................uaguacuggcauaCggacauug..	62	1	seq
...................uaguacuggcauauggacaGug..	13	1	seq
...................uaguacuggcauUuggacauug..	3	1	seq
...................uaguacuggcauauggacaCug..	45	1	seq
...................uaguacuggUauauggacauug..	40	1	seq
...................uaguacuggcauaGggacauug..	1	1	seq
...................uaguacuggcauaAggacauug..	18	1	seq
...................uaguacCggcauauggacauug..	62	1	seq
...................uaguacuggcauauggacaAug..	5	1	seq
...................uaguacuggGauauggacauug..	1	1	seq
...................uaguacuggcUuauggacauug..	11	1	seq
...................uaguacGggcauauggacauug..	21	1	seq
...................uaUuacuggcauauggacauug..	86	1	seq
...................uaguacuggcCuauggacauug..	5	1	seq
...................uaguacuggcauauggCcauug..	3	1	seq

```
                    Mature                                    Star
acacauugcuggguucuugauaguacuggcauauggacauuguuguaauaaucuuacucucaauguucaucuguccguacugccaagugccuggccuuacauucauc
....................uaguUcuggcauauggacauug.................................................................	4	1	seq
....................uaguGcuggcauauggacauug.................................................................	107	1	seq
....................uaguacAggcauauggacauug.................................................................	28	1	seq
....................uagGacuggcauauggacauug.................................................................	234	1	seq
....................uaguacuggcauauggaGauug.................................................................	4	1	seq
....................uaguacuggcauaugAacauug.................................................................	28	1	seq
....................uaguacuggcauauAgacauug.................................................................	26	1	seq
....................uaguacuggcaCauggacauug.................................................................	46	1	seq
....................uaguacuggcauauggacUuug.................................................................	7	1	seq
....................uaguacuggcaGauggacauug.................................................................	4	1	seq
....................uaguacuCgcauauggacauug.................................................................	1	1	seq
....................uagCacuggcauauggacauug.................................................................	89	1	seq
....................uaguacugCcauauggacauug.................................................................	4	1	seq
....................uaguacuggcauauggacCuug.................................................................	6	1	seq
....................uaguacuggAauauggacauug.................................................................	1	1	seq
....................uaguacuggcauauUgacauug.................................................................	15	1	seq
....................uaguacuUgcauauggacauug.................................................................	8	1	seq
....................uaguacuggcauauggaUauug.................................................................	25	1	seq
....................uaguCcuggcauauggacauug.................................................................	2	1	seq
....................uaguacuggcauauggGcauug.................................................................	120	1	seq
....................uaCuacuggcauauggacauug.................................................................	1	1	seq
....................uaguacuggcaCauggacauugu................................................................	4	1	seq
....................uaguacuggcauGuggacauugu................................................................	4	1	seq
....................uaAuacuggcauauggacauugu................................................................	4	1	seq
....................uaguGcuggcauauggacauugu................................................................	13	1	seq
....................uaUuacuggcauauggacauugu................................................................	9	1	seq
....................uagGacuggcauauggacauugu................................................................	20	1	seq
....................uaguacAggcauauggacauugu................................................................	1	1	seq
....................uaguacuggcauauggacaCugu................................................................	4	1	seq
....................uaguacuggcauauggacUuugu................................................................	1	1	seq
....................uaguacuggcauaugAacauugu................................................................	3	1	seq
....................uagCacuggcauauggacauugu................................................................	9	1	seq
....................uaguacuggcauauggaGauugu................................................................	1	1	seq
....................uaguacugAcauauggacauugu................................................................	3	1	seq
....................uaguacuAgcauauggacauugu................................................................	4	1	seq
....................uaguacuggGauauggacauugu................................................................	1	1	seq
....................uaguacuggcGuauggacauugu................................................................	12	1	seq
....................uaCuacuggcauauggacauugu................................................................	1	1	seq
....................uaguacuggcauaCggacauugu................................................................	5	1	seq
....................uaguacuggcauauggacGuugu................................................................	11	1	seq
....................uaguacugUcauauggacauugu................................................................	1	1	seq
....................uaguacuggcauauAgacauugu................................................................	6	1	seq
....................uaguaAuggcauauggacauugu................................................................	1	1	seq
....................uaguaUuggcauauggacauugu................................................................	2	1	seq
....................uaguacuggcaGauggacauugu................................................................	2	1	seq
....................uaguacuggcauauggaAauugu................................................................	2	1	seq
....................uagAacuggcauauggacauugu................................................................	6	1	seq
....................uaguacuggcauauggCcauugu................................................................	1	1	seq
....................uaguUcuggcauauggacauugu................................................................	1	1	seq
....................uaguacuggcauauggGcauugu................................................................	16	1	seq
....................uaguacuggUauauggacauugu................................................................	1	1	seq
....................uaguacuggcUuauggacauugu................................................................	1	1	seq
....................uaguacCggcauauggacauugu................................................................	11	1	seq
....................uaguacuAgcauauggacauuguu...............................................................	1	1	seq
.....................Uguacuggcauauggacauu..................................................................	1	1	seq
........................acuggcauauggacauug.................................................................	1	0	seq
```
