# Supplementary material for: Identification and Characterization of MicroRNAs from Longitudinal Muscle and Respiratory Tree in Sea Cucumber (Apostichopus japonicus) Using High-Throughput Sequencing
Source: PLoS One. 2015 Aug 5;10(8):e0134899. doi: 10.1371/journal.pone.0134899 (PMC4526669; doi:10.1371/journal.pone.0134899)
Supplement: S1 File — (ZIP) [file pone.0134899.s002.zip › S1 File/The secondary structures of the novel miRNAs in LTM/Scaffold1492_853.pdf]

Provisional ID : Scaffold1492_853
Score total : 2.4
Score for star read(s) : -1.3
Score for read counts : 0
Score for mfe : 2.1
Score for randfold : 1.6
Score for cons. seed :
Total read count : 154
Mature read count : 154
Loop read count : 0
Star read count : 0

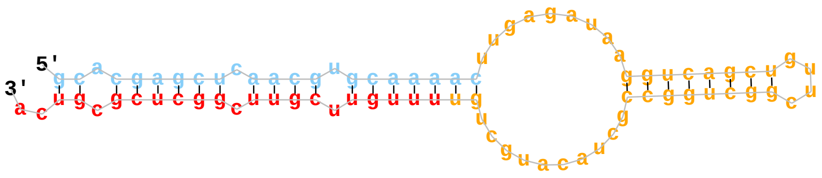

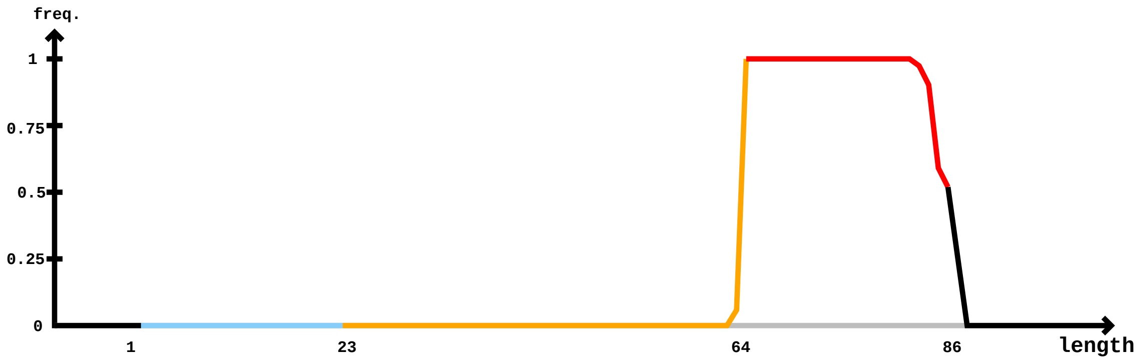

Star Mature

```
5'- caguuuggcacgagcucaacgugcaaaacuugagauaaggucagcuguucggcuggccgcuacaugcuguuuuguucguucggcucgcgucaaauugcagucacacua   -3'    exp
    ((((((((.((((((.((((.(((((((.........((((((((....))))))))..........))))))).)))).)))))).))))))))..............   reads   mm   sample
    .....................................................................uuGuguucguucggcucgc....................   1   1   seq
    .....................................................................uuuuguucguucggcucgc....................   1   0   seq
    .....................................................................uuuuguucguucggcucgcA...................   3   1   seq
    .....................................................................uuuCguucguucggcucgcgu..................   1   1   seq
    .....................................................................uuuGguucguucggcucgcgu..................   1   1   seq
    .....................................................................uuuuguucguucggcCcgcgu..................   1   1   seq
    .....................................................................uuuuguucguucggcucAcguc.................   1   1   seq
    ......................................................................uuuguucguucggcucgU....................   1   1   seq
    ......................................................................uuuguucguucggcucgc....................   1   0   seq
    ......................................................................uuuguucguucggcCcgcg...................   1   1   seq
    ......................................................................uuuguucguucggcucgcA...................   3   1   seq
    ......................................................................uuuguucguucggcucgcg...................   4   0   seq
    ......................................................................uuuguucguucggcCcgcgu..................   1   1   seq
    ......................................................................uuuguucguucggcucgcgG..................   8   1   seq
    ......................................................................uuuguucgCucggcucgcgu..................   1   1   seq
    ......................................................................uuuguucguucggcAcgcgu..................   1   1   seq
    ......................................................................uuGguucguucggcucgcgu..................   2   1   seq
    ......................................................................uuCguucguucggcucgcgu..................   1   1   seq
    ......................................................................uuuAuucguucggcucgcgu..................   1   1   seq
    ......................................................................uCuguucguucggcucgcgu..................   1   1   seq
    ......................................................................uuuguucguucggcucgUgu..................   3   1   seq
    ......................................................................uuuguucguucggcucUcgu..................   1   1   seq
    ......................................................................Cuuguucguucggcucgcgu..................   2   1   seq
    ......................................................................uuuguucguucggcucgcAu..................   1   1   seq
    ......................................................................uuuguuGguucggcucgcgu..................   1   1   seq
    ......................................................................uuuguucguucggcucgcgA..................   9   1   seq
    ......................................................................uuuguuUguucggcucgcgu..................   1   1   seq
    ......................................................................uuuUuucguucggcucgcgu..................   3   1   seq
    ......................................................................uuuguucguucAgcucgcgu..................   2   1   seq
    ......................................................................uuuguuAguucggcucgcgu..................   1   1   seq
    ......................................................................Guuguucguucggcucgcgu..................   2   1   seq
    ......................................................................uuuCuucguucggcucgcgu..................   1   1   seq
    ......................................................................uuAguucguucggcucgcgu..................   1   1   seq
    ......................................................................uuuguucgAucggcucgcgu..................   1   1   seq
```

Star **Mature**

```
caguuuggcacgagcucaacgugcaaaacuugagauaaggucagcuguucggcuggccgcuacaugcuguuuuguucguucggcucgcgucaaauugcagucacacua
......................................................................uuuguucguucggcucgGguc.................   1   1   seq
......................................................................uuuguucguucggcucAcguc.................   2   1   seq
......................................................................uCuguucguucggcucgcguc.................   1   1   seq
......................................................................uuAguucguucggcucgcguc.................   1   1   seq
......................................................................uuuUuucguucggcucgcguc.................   1   1   seq
......................................................................uuuguucguucAgcucgcguc.................   1   1   seq
......................................................................uuuguucguucggcuUgcguc.................   1   1   seq
......................................................................uuugCucguucggcucgcguc.................   1   1   seq
......................................................................uuuguucguucggcucgcgGc.................   1   1   seq
......................................................................uuugCucguucggcucgcguca................   6   1   seq
......................................................................uuuguucgCucggcucgcguca................   2   1   seq
......................................................................uuuguCcguucggcucgcguca................   8   1   seq
......................................................................uuuguuGguucggcucgcguca................   2   1   seq
......................................................................uuuguucguucgAcucgcguca................   1   1   seq
......................................................................uuuguucguucggcucCcguca................   1   1   seq
......................................................................uuuguucguucggcucgcgAca................   1   1   seq
......................................................................uuuguucguucggcucgcUuca................   1   1   seq
......................................................................uuCguucguucggcucgcguca................   6   1   seq
......................................................................uuuguucguucggcCcgcguca................   4   1   seq
......................................................................uuuguucguucAgcucgcguca................   2   1   seq
......................................................................uuuguucguucggUucgcguca................   1   1   seq
......................................................................uuGguucguucggcucgcguca................   6   1   seq
......................................................................uuuguucguucggcGcgcguca................   1   1   seq
......................................................................uuuguucguucggcucgUguca................   3   1   seq
......................................................................uuuguucguucggcuAgcguca................   1   1   seq
......................................................................uuuguucguuUggcucgcguca................   5   1   seq
......................................................................uuuAuucguucggcucgcguca................   2   1   seq
......................................................................uuuUuucguucggcucgcguca................   14  1   seq
......................................................................uuuguucguucggcucUcguca................   1   1   seq
......................................................................uuAguucguucggcucgcguca................   3   1   seq
......................................................................uuuguucguucggcucAcguca................   5   1   seq
......................................................................uuuguucguucggcucgcgGca................   2   1   seq
......................................................................uuugGucguucggcucgcguca................   1   1   seq
......................................................................uuuguucgAucggcucgcguca................   1   1   seq
```
