# Supplementary material for: Identification and Characterization of MicroRNAs from Longitudinal Muscle and Respiratory Tree in Sea Cucumber (Apostichopus japonicus) Using High-Throughput Sequencing
Source: PLoS One. 2015 Aug 5;10(8):e0134899. doi: 10.1371/journal.pone.0134899 (PMC4526669; doi:10.1371/journal.pone.0134899)
Supplement: S1 File — (ZIP) [file pone.0134899.s002.zip › S1 File/The secondary structures of the novel miRNAs in LTM/Scaffold22_15.pdf]

Provisional ID	: Scaffold22_15
Score total	: 2.3
Score for star read(s)	: -1.3
Score for read counts	: 0
Score for mfe	: 2
Score for randfold	: 1.6
Score for cons. seed	:
Total read count	: 2488
Mature read count	: 2488
Loop read count	: 0
Star read count	: 0

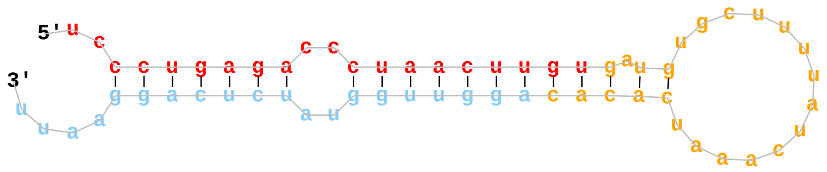

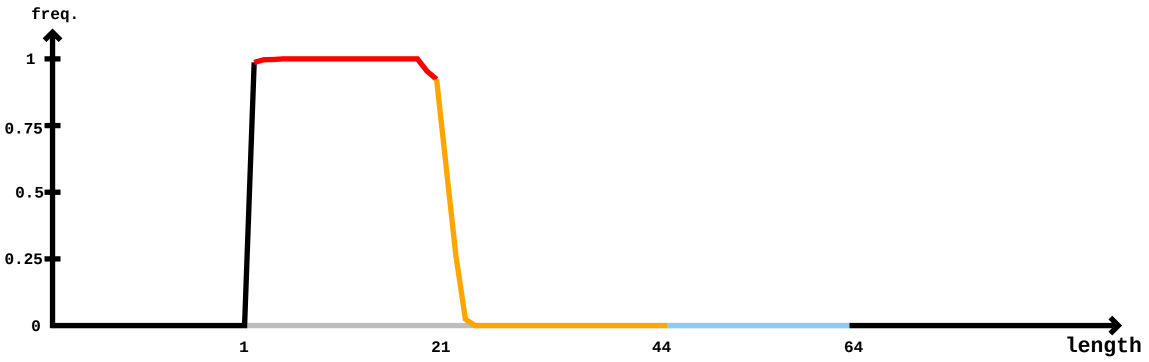

Mature Star

```
5'- agagagacugccccccaaaucccugagacccuaacuugugaugugcuuuuaucaaaucacacagguugguaucucaggaauuuggugggcgugucaugccaacgaucuc  -3'   exp
    .((((((((((((.((((((.(((((((..((((((((((.((..............))))))))))))..))))))).)))))).))))).)))..........))))    reads   mm   sample
    ...................Acccugagacccuaacuu........................................................................    1       1    seq
    ...................ucccCgagacccuaacuu........................................................................    1       1    seq
    ...................ucccugagacccCaacuu........................................................................    1       1    seq
    ...................uUccugagacccuaacuu........................................................................    3       1    seq
    ...................ucccugagacccuaacuG........................................................................    2       1    seq
    ...................ucccugagacccuaacuC........................................................................    13      1    seq
    ...................ucccugagacccuaacuu........................................................................    91      0    seq
    ...................ucccugagacUcuaacuu........................................................................    1       1    seq
    ...................ucUcugagacccuaacuu........................................................................    1       1    seq
    ...................ucccugagacccuaacuA........................................................................    1       1    seq
    ...................ucccugagacccuaacuug.......................................................................    62      0    seq
    ...................ucccugagacccuaacuCg.......................................................................    1       1    seq
    ...................ucccugagacccuaacuuA.......................................................................    4       1    seq
    ...................ucccugagacccuaacuuU.......................................................................    9       1    seq
    ...................Acccugagacccuaacuugu......................................................................    5       1    seq
    ...................uccGugagacccuaacuugu......................................................................    3       1    seq
    ...................ucccugagacccuaUcuugu......................................................................    2       1    seq
    ...................ucccugagacccuUacuugu......................................................................    1       1    seq
    ...................ucccCgagacccuaacuugu......................................................................    13      1    seq
    ...................ucccugagacccCaacuugu......................................................................    7       1    seq
    ...................ucccugagacUcuaacuugu......................................................................    15      1    seq
    ...................Gcccugagacccuaacuugu......................................................................    20      1    seq
    ...................ucccugagacGcuaacuugu......................................................................    1       1    seq
    ...................ucccugagacccuaacCugu......................................................................    10      1    seq
    ...................ucccugagacccAaacuugu......................................................................    3       1    seq
    ...................ucccugUgacccuaacuugu......................................................................    2       1    seq
    ...................ucccuAagacccuaacuugu......................................................................    6       1    seq
    ...................ucccugagacccuaacuugG......................................................................    85      1    seq
    ...................ucccugagacccuaacuuCu......................................................................    1       1    seq
    ...................uGccugagacccuaacuugu......................................................................    5       1    seq
    ...................ucccugaAacccuaacuugu......................................................................    5       1    seq
    ...................ucccugagacccuCacuugu......................................................................    1       1    seq
    ...................ucccugagacccuaacuugC......................................................................    409     1    seq
    ...................ucccugagCcccuaacuugu......................................................................    1       1    seq
```

```
                  Mature                                      Star
agagagacugccccccaaaucccugagacccuaacuugugaugugcuuuuaucaaaucacacagguugguaucucaggaauuuggugggcgugucaugccaacgaucuc
...................ucccugagGcccuaacuugu.....................................................................	15	1	seq
...................ucccugGgacccuaacuugu.....................................................................	16	1	seq
...................ucccugagacccuaacuugA.....................................................................	117	1	seq
...................uAccugagacccuaacuugu.....................................................................	1	1	seq
...................uUccugagacccuaacuugu.....................................................................	7	1	seq
...................ucccugagacccuaacuuUu.....................................................................	7	1	seq
...................ucccugagacccuaacuGgu.....................................................................	2	1	seq
...................ucAcugagacccuaacuugu.....................................................................	1	1	seq
...................ucccuUagacccuaacuugu.....................................................................	1	1	seq
...................ucccugagacccuaacuAgu.....................................................................	1	1	seq
...................ucccugagacccuaCcuugu.....................................................................	1	1	seq
...................ucGcugagacccuaacuugu.....................................................................	1	1	seq
...................ucccugagUcccuaacuugu.....................................................................	2	1	seq
...................ucccugCgacccuaacuugu.....................................................................	2	1	seq
...................ucccugagacccuaGcuugu.....................................................................	10	1	seq
...................ucccGgagacccuaacuugu.....................................................................	1	1	seq
...................ucccugagacccuGacuugu.....................................................................	10	1	seq
...................ucccugagaUccuaacuugu.....................................................................	7	1	seq
...................ucUcugagacccuaacuugu.....................................................................	17	1	seq
...................ucccAgagacccuaacuugu.....................................................................	3	1	seq
...................ucccugagacccGaacuugu.....................................................................	1	1	seq
...................ucccugagacccuaaGuugu.....................................................................	2	1	seq
...................uccUugagacccuaacuugu.....................................................................	12	1	seq
...................ucccugagaccUuaacuugu.....................................................................	10	1	seq
...................ucccugagacccuaacuuAu.....................................................................	4	1	seq
...................ucccugagacccuaacAugu.....................................................................	1	1	seq
...................ucccugaUacccuaacuugug...................................................................	2	1	seq
...................ucccuUagacccuaacuugug...................................................................	1	1	seq
...................ucccugagacccuCacuugug...................................................................	3	1	seq
...................ucccugagacccuaaUuugug...................................................................	7	1	seq
...................ucccugCgacccuaacuugug...................................................................	6	1	seq
...................ucccugagacccuaacuugAg...................................................................	11	1	seq
...................ucccugagacccuaCcuugug...................................................................	2	1	seq
...................ucccugagacGcuaacuugug...................................................................	2	1	seq
...................ucccugagacccGaacuugug...................................................................	1	1	seq
...................ucccugagaccUuaacuugug...................................................................	18	1	seq
...................ucccAgagacccuaacuugug...................................................................	10	1	seq
...................ucccugagacccuaacuuCug...................................................................	1	1	seq
...................ucccugagacccuaacuugCg...................................................................	91	1	seq
...................ucccugagaccGuaacuugug...................................................................	3	1	seq
...................uccAugagacccuaacuugug...................................................................	5	1	seq
...................ucccugagaGccuaacuugug...................................................................	1	1	seq
...................uccUugagacccuaacuugug...................................................................	26	1	seq
...................ucccugagacccuUacuugug...................................................................	5	1	seq
...................ucccugaAacccuaacuugug...................................................................	12	1	seq
...................ucccugagacccuaacuAgug...................................................................	2	1	seq
...................ucccugagacccuaaGuugug...................................................................	4	1	seq
...................uAccugagacccuaacuugug...................................................................	2	1	seq
...................ucccugagCcccuaacuugug...................................................................	4	1	seq
...................ucccugagacccAaacuugug...................................................................	9	1	seq
...................uUccugagacccuaacuugug...................................................................	9	1	seq
...................ucccCgagacccuaacuugug...................................................................	40	1	seq
...................ucccugagacccuGacuugug...................................................................	27	1	seq
...................ucccugagacccuaacCugug...................................................................	35	1	seq
...................ucccugagacccuaGcuugug...................................................................	44	1	seq
...................ucccugGgacccuaacuugug...................................................................	51	1	seq
...................ucccugagacccuaacAugug...................................................................	3	1	seq
...................ucccugagUcccuaacuugug...................................................................	8	1	seq
...................ucccugagacccuaacuugGg...................................................................	28	1	seq
...................ucccugagacAcuaacuugug...................................................................	4	1	seq
...................uGccugagacccuaacuugug...................................................................	9	1	seq
...................ucccugaCacccuaacuugug...................................................................	1	1	seq
...................ucccugagaccAuaacuugug...................................................................	1	1	seq
...................ucccugagacccuaacuuUug...................................................................	19	1	seq
...................ucccugagacccuaacGugug...................................................................	2	1	seq
...................ucUcugagacccuaacuugug...................................................................	50	1	seq
...................ucccugagacUcuaacuugug...................................................................	44	1	seq
...................ucccugagacccuaacuGgug...................................................................	11	1	seq
...................ucccugagaUccuaacuugug...................................................................	7	1	seq
...................ucccugagacccuaacuuAug...................................................................	22	1	seq
```

Mature Star

agagagacugcccccaaaucccugagacccuaacuugugaugugcuuuuaucaaaucacacagguugguaucucaggaauuuggugggcgugucaugccaacgaucuc

..................ucccuAagacccuaacuugug...	14	1	seq
..................ucccGgagacccuaacuugug...	4	1	seq
..................uccGugagacccuaacuugug...	16	1	seq
..................ucccugagacccuaUcuugug...	3	1	seq
..................ucccugagGcccuaacuugug...	59	1	seq
..................ucccugUgacccuaacuugug...	9	1	seq
..................ucccugagacccCaacuugug...	22	1	seq
..................ucGcugagacccuaacuugug...	1	1	seq
..................ucccugagacccuaaAuugug...	1	1	seq
..................ucAcugagacccuaacuugug...	3	1	seq
..................ucccugGgacccuaacuuguga..	45	1	seq
..................ucccugagacccAaacuuguga..	6	1	seq
..................ucccAgagacccuaacuuguga..	10	1	seq
..................ucccugagaUccuaacuuguga..	12	1	seq
..................ucccugagacccuaCcuuguga..	1	1	seq
..................ucccugagacccGaacuuguga..	4	1	seq
..................ucccGgagacccuaacuuguga..	3	1	seq
..................uccGugagacccuaacuuguga..	10	1	seq
..................ucccugagacccuaacuugGga..	13	1	seq
..................ucccugagacccuaacAuguga..	8	1	seq
..................ucccugagacGcuaacuuguga..	3	1	seq
..................ucccugagacccuaacuugCga..	37	1	seq
..................uccAugagacccuaacuuguga..	2	1	seq
..................ucccuAagacccuaacuuguga..	3	1	seq
..................ucccugagCcccuaacuuguga..	6	1	seq
..................ucccugCgacccuaacuuguga..	3	1	seq
..................ucccugagGcccuaacuuguga..	46	1	seq
..................ucccugagaccUuaacuuguga..	15	1	seq
..................ucccugagacccuaaGuuguga..	1	1	seq
..................ucccugagacccuUacuuguga..	2	1	seq
..................ucccugagacccuaacuuAuga..	8	1	seq
..................ucccugagacccuaacuAguga..	6	1	seq
..................ucccugagacUcuaacuuguga..	66	1	seq
..................ucccCgagacccuaacuuguga..	25	1	seq
..................ucccugagacccuaacuugAga..	9	1	seq
..................ucccugagacAcuaacuuguga..	3	1	seq
..................ucUcugagacccuaacuuguga..	37	1	seq
..................ucccugagacccuGacuuguga..	33	1	seq
..................ucccugUgacccuaacuuguga..	3	1	seq
..................ucAcugagacccuaacuuguga..	3	1	seq
..................ucccuUagacccuaacuuguga..	2	1	seq
..................ucccugagaccAuaacuuguga..	2	1	seq
..................ucccugagacccCaacuuguga..	16	1	seq
..................ucccugagaAccuaacuuguga..	2	1	seq
..................ucccugagacccuaacuGguga..	5	1	seq
..................ucccugagaGccuaacuuguga..	1	1	seq
..................ucccugagacccuaGcuuguga..	30	1	seq
..................ucGcugagacccuaacuuguga..	1	1	seq
..................ucccugagacccuaaAuuguga..	1	1	seq
..................ucccugaAacccuaacuuguga..	9	1	seq
..................ucccugagacccuaacuuUuga..	9	1	seq
..................ucccugagUcccuaacuuguga..	5	1	seq
..................ucccugagacccuaacuuCuga..	1	1	seq
..................ucccugagacccuaUcuuguga..	2	1	seq
..................ucccugagacccuaacCuguga..	28	1	seq
..................ucccuCagacccuaacuuguga..	2	1	seq
..................ucccugagacccuaaUuuguga..	13	1	seq
..................ucccugagacccuCacuuguga..	3	1	seq
..................uccUugagacccuaacuuguga..	28	1	seq
..................ucccCgagacccuaacuugugau...	4	1	seq
..................ucccugagacccuaacuGgugau...	1	1	seq
..................ucccugagacccuaacuAgugau...	1	1	seq
..................ucccugGgacccuaacuugugau...	3	1	seq
..................ucccAgagacccuaacuugugau...	2	1	seq
..................ucUcugagacccuaacuugugau...	6	1	seq
..................ucccugagacccuaGcuugugau...	3	1	seq
..................ucccuAagacccuaacuugugau...	3	1	seq
..................uccUugagacccuaacuugugau...	2	1	seq
..................ucccugagUcccuaacuugugau...	1	1	seq
..................ucccugagacUcuaacuugugau...	2	1	seq

```
                  Mature                                      Star
agagagacugcccccaaaucccugagacccuaacuugugaugugcuuuuaucaaaucacacagguugguaucucaggaauuuggugggcgugucaugccaacgaucuc
..................ucccugagacccuaacuugCgau...................................................................	4	1	seq
..................ucccugagacccCaacuugugau...................................................................	5	1	seq
..................ucccugagacccuaacCugugau...................................................................	1	1	seq
..................ucccugagacccuGacuugugau...................................................................	2	1	seq
..................ucccugagaccUuaacuugugau...................................................................	1	1	seq
..................ucccugagacGcuaacuugugau...................................................................	1	1	seq
..................ucccugUgacccuaacuugugau...................................................................	2	1	seq
..................ucccugagacAcuaacuugugau...................................................................	1	1	seq
..................ucccugagGcccuaacuugugau...................................................................	10	1	seq
..................ucccugaUacccuaacuugugau...................................................................	1	1	seq
..................ucccGgagacccuaacuugugau...................................................................	1	1	seq
..................ucccugCgacccuaacuugugau...................................................................	2	1	seq
...................cccugagacccuaacuugu......................................................................	4	0	seq
...................cccugagacccuaacuugC......................................................................	2	1	seq
...................Uccugagacccuaacuugu......................................................................	1	1	seq
...................cccGgagacccuaacuugug.....................................................................	1	1	seq
...................cccugGgacccuaacuugug.....................................................................	2	1	seq
...................cccugagacccuaacuuguU.....................................................................	2	1	seq
...................Uccugagacccuaacuugug.....................................................................	4	1	seq
...................cccugagacccuaaGuuguga....................................................................	1	1	seq
...................cccugagacccuaacuuguUa....................................................................	1	1	seq
...................cccugagacccuaacuugCga....................................................................	1	1	seq
...................cccugGgacccuaacuuguga....................................................................	1	1	seq
...................cccugagaUccuaacuuguga....................................................................	1	1	seq
...................cccCgagacccuaacuuguga....................................................................	1	1	seq
...................cccugagacUcuaacuuguga....................................................................	1	1	seq
...................cccugagacccuaacCuguga....................................................................	1	1	seq
...................cccugagacccuaacuuUuga....................................................................	1	1	seq
....................ccugagacccuaacuugug.....................................................................	2	0	seq
.....................cugagacccuaacuuguga....................................................................	6	0	seq
```
