# Supplementary material for: Identification and Characterization of MicroRNAs from Longitudinal Muscle and Respiratory Tree in Sea Cucumber (Apostichopus japonicus) Using High-Throughput Sequencing
Source: PLoS One. 2015 Aug 5;10(8):e0134899. doi: 10.1371/journal.pone.0134899 (PMC4526669; doi:10.1371/journal.pone.0134899)
Supplement: S1 File — (ZIP) [file pone.0134899.s002.zip › S1 File/The secondary structures of the novel miRNAs in LTM/Scaffold22_17.pdf]

Provisional ID	:	Scaffold22_17
Score total	:	2619.6
Score for star read(s)	:	3.9
Score for read counts	:	2612.5
Score for mfe	:	1.7
Score for randfold	:	1.6
Score for cons. seed	:	
Total read count	:	5136
Mature read count	:	5083
Loop read count	:	0
Star read count	:	53

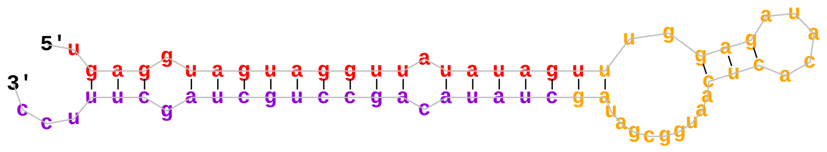

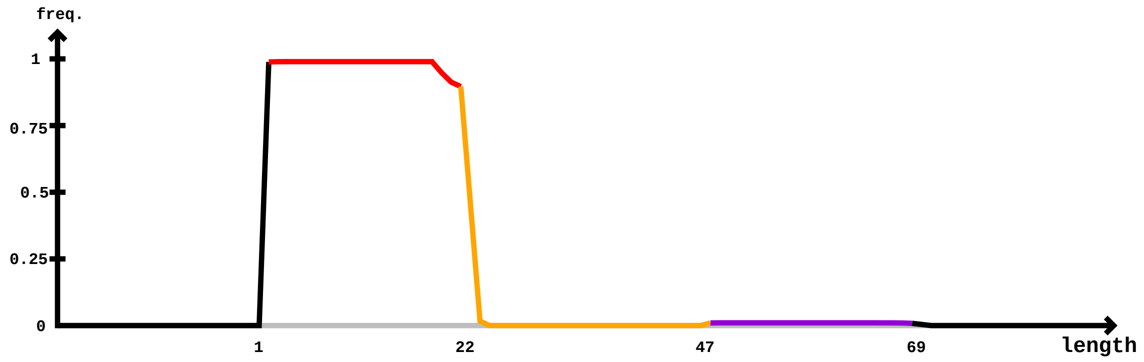

Mature Star

```
5'- guacaugaaugguccuuacgugagguaguagguuauauaguuuggagauacacucaauggcgauagcuauacagccugcuagcuuuccuugggccauucacaaaucuu  -3'   obs
    guacaugaaugguccuuacgugagguaguagguuauauaguuuggagauacacucaauggcgauagcuauacagccugcuagcuuuccuugggccauucacaaaucuu         exp
    .....(((((((((......(((.(((((((((.((((((..(((.....)))........)))))).))))))))).))).....)))))))))........         reads   mm   sample
    ....................ugagguaAuagguuauau......................................................................    1       1    seq
    ....................ugagguaguaggCuauau......................................................................    1       1    seq
    ....................ugaUguaguagguuauau......................................................................    1       1    seq
    ....................ugaggAaguagguuauau......................................................................    1       1    seq
    ....................ugagguaguagguuGuau......................................................................    2       1    seq
    ....................ugagguaguaUguuauau......................................................................    1       1    seq
    ....................ugagguaguagguuauau......................................................................    182     0    seq
    ....................ugagguaguagguuauaC......................................................................    21      1    seq
    ....................uAagguaguagguuauau......................................................................    1       1    seq
    ....................ugagguaguagguuauaG......................................................................    3       1    seq
    ....................ugagguaguagguuauauC.....................................................................    4       1    seq
    ....................ugagguaguagguuauGua.....................................................................    1       1    seq
    ....................ugagguaguagguuauauG.....................................................................    2       1    seq
    ....................uUagguaguagguuauaua.....................................................................    1       1    seq
    ....................ugaUguaguagguuauaua.....................................................................    1       1    seq
    ....................ugaggAaguagguuauaua.....................................................................    1       1    seq
    ....................ugagguaguagguuauauU.....................................................................    76      1    seq
    ....................ugagguaguGgguuauaua.....................................................................    1       1    seq
    ....................ugagguaguagguuGuaua.....................................................................    3       1    seq
    ....................ugaggCaguagguuauaua.....................................................................    1       1    seq
    ....................ugagguaguagguuauaua.....................................................................    89      0    seq
    ....................ugagguaguaggCuauaua.....................................................................    1       1    seq
    ....................Cgagguaguagguuauauag....................................................................    2       1    seq
    ....................ugagguaguagguuauauaA....................................................................    44      1    seq
    ....................Ggagguaguagguuauauag....................................................................    1       1    seq
    ....................ugagguGguagguuauauag....................................................................    1       1    seq
    ....................ugagguagGagguuauauag....................................................................    1       1    seq
    ....................uUagguaguagguuauauag....................................................................    1       1    seq
    ....................ugUgguaguagguuauauag....................................................................    3       1    seq
    ....................ugaggCaguagguuauauag....................................................................    2       1    seq
    ....................ugagguaguagguuauauaU....................................................................    14      1    seq
    ....................ugagguaguagguuauauaC....................................................................    1       1    seq
    ....................ugaAguaguagguuauauag....................................................................    1       1    seq
```

```
                   Mature                                       Star
guacaugaauggucuuacgugagguaguagguuauauaguuuggagauacacucaaggcgauagcuauacagccugcuagcuuuccuugggccauucacaaaucuu
...................ugaggGaguagguuauauag...................................................................  1    1  seq
...................ugagguaguagguCauauag...................................................................  3    1  seq
...................ugagguaguaAguuauauag...................................................................  2    1  seq
...................ugagguaguagguuauGuag...................................................................  1    1  seq
...................ugagguaguGgguuauauag...................................................................  1    1  seq
...................ugagguaguaggGuauauag...................................................................  1    1  seq
...................ugaUguaguagguuauauag...................................................................  4    1  seq
...................ugGgguaguagguuauauag...................................................................  1    1  seq
...................ugagguaguagguuUuauagu..................................................................  5    1  seq
...................ugagguaguagguuaGauagu..................................................................  10   1  seq
...................ugagguaAuagguuauauagu..................................................................  24   1  seq
...................ugGgguaguagguuauauagu..................................................................  200  1  seq
...................ugagguaguagguuaAauagu..................................................................  8    1  seq
...................ugagguaguagguuauauaAu..................................................................  21   1  seq
...................ugaUguaguagguuauauagu..................................................................  352  1  seq
...................ugagguaguagguuauUuagu..................................................................  5    1  seq
...................ugagguaguagguuauauaCu..................................................................  5    1  seq
...................ugagguaguagguuauauaUu..................................................................  12   1  seq
...................ugagguaguaUguuauauagu..................................................................  8    1  seq
...................ugaAguaguagguuauauagu..................................................................  69   1  seq
...................ugagguaCuagguuauauagu..................................................................  2    1  seq
...................ugUgguaguagguuauauagu..................................................................  226  1  seq
...................ugaggGaguagguuauauagu..................................................................  81   1  seq
...................ugaggCaguagguuauauagu..................................................................  148  1  seq
...................ugagguGguagguuauauagu..................................................................  121  1  seq
...................ugagguaguagguCauauagu..................................................................  65   1  seq
...................ugagguaguagguuauaCagu..................................................................  53   1  seq
...................ugagguaguagguGauauagu..................................................................  3    1  seq
...................ugagguaguUgguuauauagu..................................................................  17   1  seq
...................ugagguaguagUuuauauagu..................................................................  3    1  seq
...................ugagguaguaggCuauauagu..................................................................  138  1  seq
...................ugagguagGagguuauauagu..................................................................  18   1  seq
...................ugCgguaguagguuauauagu..................................................................  8    1  seq
...................ugagguaguagguuauauCgu..................................................................  8    1  seq
...................ugagUuaguagguuauauagu..................................................................  1    1  seq
...................uUagguaguagguuauauagu..................................................................  70   1  seq
...................ugagguCguagguuauauagu..................................................................  5    1  seq
...................ugagguaguagguAauauagu..................................................................  4    1  seq
...................ugagguaguagCuuauauagu..................................................................  3    1  seq
...................ugagguaguagguuauaGagu..................................................................  13   1  seq
...................ugagguaguagguuauauGgu..................................................................  97   1  seq
...................ugagguaguagguuauauUgu..................................................................  30   1  seq
...................ugaggAaguagguuauauagu..................................................................  70   1  seq
...................uCagguaguagguuauauagu..................................................................  2    1  seq
...................ugagguaguagguuauaAagu..................................................................  6    1  seq
...................ugagguaguagguuCuauagu..................................................................  2    1  seq
...................ugagguaguagguuauGuagu..................................................................  94   1  seq
...................ugagguUguagguuauauagu..................................................................  21   1  seq
...................ugagguaguGgguuauauagu..................................................................  107  1  seq
...................ugagguaguagguuaCauagu..................................................................  74   1  seq
...................ugagguagAagguuauauagu..................................................................  45   1  seq
...................ugagguaguaggGuauauagu..................................................................  29   1  seq
...................ugagguaguagAuuauauagu..................................................................  21   1  seq
...................ugagguaguaggAuauauagu..................................................................  34   1  seq
...................uAagguaguagguuauauagu..................................................................  45   1  seq
...................ugagCuaguagguuauauagu..................................................................  3    1  seq
...................ugagAuaguagguuauauagu..................................................................  19   1  seq
...................ugagguaguCgguuauauagu..................................................................  3    1  seq
...................ugagguagCagguuauauagu..................................................................  86   1  seq
...................ugaCguaguagguuauauagu..................................................................  16   1  seq
...................ugagguaguaAguuauauagu..................................................................  36   1  seq
...................ugagguaUuagguuauauagu..................................................................  5    1  seq
...................ugaCguaguagguuauauaguu.................................................................  13   1  seq
...................ugaggAaguagguuauauaguu.................................................................  71   1  seq
...................ugaggCaguagguuauauaguu.................................................................  103  1  seq
...................ugagguaguagguuCuauaguu.................................................................  3    1  seq
...................ugagguaguaCguuauauaguu.................................................................  1    1  seq
...................ugagguaguagguCauauaguu.................................................................  51   1  seq
...................ugagguaguagguuauUuaguu.................................................................  3    1  seq
...................ugagguCguagguuauauaguu.................................................................  2    1  seq
```

```
                    Mature                                       Star
guacaugaaugguccuuacgugagguaguagguuauauaguuuggagauacacucaaggcgauagcuauacagccugcuagcuuuccuugggccauucacaaaucuu
....................ugagguaguaUguuauauaguu.................................................................  5    1  seq
....................ugagguaguagAuuauauaguu.................................................................  23   1  seq
....................ugUgguaguagguuauauaguu.................................................................  157  1  seq
....................ugagguagGagguuauauaguu.................................................................  19   1  seq
....................ugagUuaguagguuauauaguu.................................................................  1    1  seq
....................ugCgguaguagguuauauaguu.................................................................  3    1  seq
....................ugagguagCagguuauauaguu.................................................................  57   1  seq
....................ugagguaguagguuauauaCuu.................................................................  1    1  seq
....................ugagguaguagguuauaCaguu.................................................................  54   1  seq
....................ugagAuaguagguuauauaguu.................................................................  17   1  seq
....................ugaUguaguagguuauauaguu.................................................................  306  1  seq
....................ugGgguaguagguuauauaguu.................................................................  159  1  seq
....................ugagguaguGgguuauauaguu.................................................................  94   1  seq
....................ugagguaguaAguuauauaguu.................................................................  26   1  seq
....................ugagguaguagCuuauauaguu.................................................................  1    1  seq
....................ugagguaguaggAuauauaguu.................................................................  39   1  seq
....................ugagguaguagguuauauUguu.................................................................  21   1  seq
....................ugagguaguagUuuauauaguu.................................................................  3    1  seq
....................ugagguaguagguAauauaguu.................................................................  5    1  seq
....................ugaAguaguagguuauauaguu.................................................................  32   1  seq
....................ugagguaguagguuauauGguu.................................................................  106  1  seq
....................ugaggGaguagguuauauaguu.................................................................  38   1  seq
....................ugagguUguagguuauauaguu.................................................................  20   1  seq
....................ugagguaguaggGuauauaguu.................................................................  18   1  seq
....................ugagguaguagguuauGuaguu.................................................................  81   1  seq
....................ugagguaguagguuauauaUuu.................................................................  13   1  seq
....................ugagguaUuagguuauauaguu.................................................................  4    1  seq
....................ugagguGguagguuauauaguu.................................................................  81   1  seq
....................ugagguaguagguuauaAaguu.................................................................  2    1  seq
....................ugagCuaguagguuauauaguu.................................................................  2    1  seq
....................ugagguagAagguuauauaguu.................................................................  27   1  seq
....................ugagguaCuagguuauauaguu.................................................................  1    1  seq
....................ugagguaguUgguuauauaguu.................................................................  15   1  seq
....................ugagguaguagguuaAauaguu.................................................................  6    1  seq
....................ugagguaguagguuauauCguu.................................................................  4    1  seq
....................ugagguaguagguuaGauaguu.................................................................  8    1  seq
....................ugagguaguagguuaCauaguu.................................................................  57   1  seq
....................ugagguaguagguGauauaguu.................................................................  4    1  seq
....................ugagguaguagguUuauaguu..................................................................  10   1  seq
....................ugagguaguagguuauaGaguu.................................................................  5    1  seq
....................ugagguaguaggCuauauaguu.................................................................  140  1  seq
....................ugagguaguagguuauCuaguu.................................................................  1    1  seq
....................ugagguaAuagguuauauaguu.................................................................  27   1  seq
....................ugagguaguagguuauauaAuu.................................................................  16   1  seq
....................ugagguaguCgguuauauaguu.................................................................  4    1  seq
....................ugGgguaguagguuauauaguuu................................................................  8    1  seq
....................ugaggAaguagguuauauaguuu................................................................  1    1  seq
....................ugaggCaguagguuauauaguuu................................................................  3    1  seq
....................ugagguaguagguuauauaAuuu................................................................  2    1  seq
....................ugaAguaguagguuauauaguuu................................................................  2    1  seq
....................ugagguaguagguuauauGguuu................................................................  4    1  seq
....................ugagguaAuagguuauauaguuu................................................................  1    1  seq
....................ugagguUguagguuauauaguuu................................................................  1    1  seq
....................ugagguaguagguuaCauaguuu................................................................  5    1  seq
....................ugagguaguaggCuauauaguuu................................................................  8    1  seq
....................ugagguagAagguuauauaguuu................................................................  2    1  seq
....................ugagguaguGgguuauauaguuu................................................................  4    1  seq
....................ugaUguaguagguuauauaguuu................................................................  15   1  seq
....................ugagguaguaggAuauauaguuu................................................................  2    1  seq
....................ugagguCguagguuauauaguuu................................................................  1    1  seq
....................ugagguaguUgguuauauaguuu................................................................  2    1  seq
....................ugagguagCagguuauauaguuu................................................................  2    1  seq
....................ugUgguaguagguuauauaguuu................................................................  8    1  seq
....................ugaggGaguagguuauauaguuu................................................................  2    1  seq
....................ugagguagGagguuauauaguuu................................................................  1    1  seq
....................ugagguaguagguuauauUguuu................................................................  1    1  seq
....................ugagguaguaAguuauauaguuu................................................................  1    1  seq
....................ugagguGguagguuauauaguuu................................................................  3    1  seq
....................ugagguaguagguuauaCaguuu................................................................  7    1  seq
.....................gagguaguagguuauauagC..................................................................  2    1  seq
```

```
                    Mature                                       Star
guacaugaauggucc uuacgugagguaguagguuauauaguuuggagauacacucaauggcgauagcuauacagccugcuagcuuuccuugggccauucacaaaucuu
.....................Aagguaguagguuauauagu...................................................................   1    1    seq
.....................gaggCaguagguuauauaguu..................................................................   1    1    seq
.....................gGgguaguagguuauauaguu..................................................................   1    1    seq
......................agguaguagguuauauagu...................................................................   1    0    seq
..................................................................cuauacaAccugcuagcu........................   1    1    seq
..................................................................cuauacaAccugcuagcuuu......................   2    1    seq
..................................................................cuauacaAccugcuagcuuuc.....................   7    1    seq
..................................................................cuauacaAccugcuagcuuucc....................   41   1    seq
...................................................................uauacaAccugcuagcuuuccu...................   2    1    seq
```
