# Supplementary material for: Identification and Characterization of MicroRNAs from Longitudinal Muscle and Respiratory Tree in Sea Cucumber (Apostichopus japonicus) Using High-Throughput Sequencing
Source: PLoS One. 2015 Aug 5;10(8):e0134899. doi: 10.1371/journal.pone.0134899 (PMC4526669; doi:10.1371/journal.pone.0134899)
Supplement: S1 File — (ZIP) [file pone.0134899.s002.zip › S1 File/The secondary structures of the novel miRNAs in LTM/Scaffold280_281.pdf]

Provisional ID : Scaffold280_281
Score total : 1.4
Score for star read(s) : -1.3
Score for read counts : 0
Score for mfe : 1.1
Score for randfold : 1.6
Score for cons. seed :
Total read count : 15927
Mature read count : 15927
Loop read count : 0
Star read count : 0

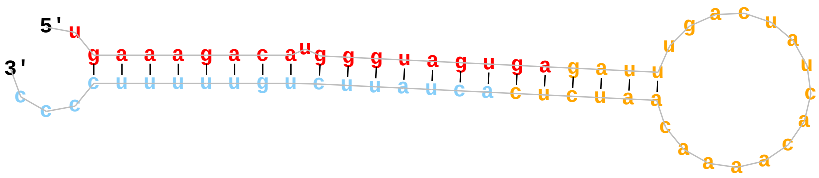

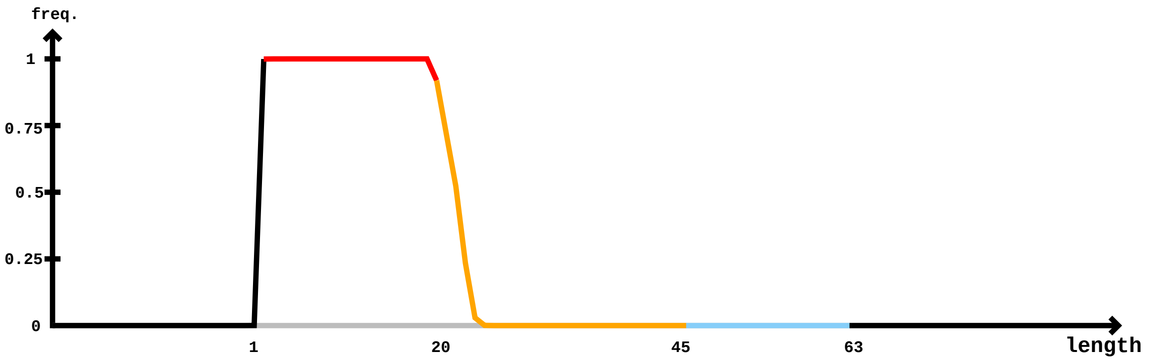

Mature **Star**

```
5'- cagaaucgguguucccugagugaaagacauggguagugagauuugacuaucacaaaacaaucucacuauucuguuuuuccccugguggauacuuauuacaucuacuacc   -3'    exp
    ...(((.(((((((((.(.(.((((((((.((((((((((((..............))))))))))))))))))))).).)).))))))).)))...............    reads    mm    sample
    ...................Augaaagacauggguaguga......................................................................    1    1    seq
    ...................Cugaaagacauggguaguga......................................................................    2    1    seq
    ....................ugGaagacauggguagug.......................................................................    5    1    seq
    ....................ugaaagacaGgguagug........................................................................    2    1    seq
    ....................ugaaagacaugggAagug.......................................................................    5    1    seq
    ....................ugaaagacauggguagCg.......................................................................    6    1    seq
    ....................ugUaagacauggguagug.......................................................................    3    1    seq
    ....................ugaaagacauggguaguA.......................................................................    131    1    seq
    ....................ugaaagacaugggugGug.......................................................................    3    1    seq
    ....................ugaaagacauggAuagug.......................................................................    1    1    seq
    ....................ugaaagacUuggguagug.......................................................................    1    1    seq
    ....................ugaaagacauAgguagug.......................................................................    4    1    seq
    ....................ugaaagacaugAguagug.......................................................................    2    1    seq
    ....................ugaaCgacauggguagug.......................................................................    2    1    seq
    ....................uUaaagacauggguagug.......................................................................    3    1    seq
    ....................ugaaagacauggguagGg.......................................................................    2    1    seq
    ....................ugaGagacauggguagug.......................................................................    2    1    seq
    ....................ugaaaAacauggguagug.......................................................................    1    1    seq
    ....................ugaaagGcauggguagug.......................................................................    3    1    seq
    ....................Agaaagacauggguagug.......................................................................    1    1    seq
    ....................uCaaagacauggguagug.......................................................................    1    1    seq
    ....................ugaaGgacauggguagug.......................................................................    5    1    seq
    ....................ugaaagacaCggguagug.......................................................................    2    1    seq
    ....................ugaaagacaugUguagug.......................................................................    1    1    seq
    ....................ugaaagacaugggGagug.......................................................................    1    1    seq
    ....................ugaaagacauggguaUug.......................................................................    1    1    seq
    ....................ugaaagacaugggCagug.......................................................................    3    1    seq
    ....................Cgaaagacauggguagug.......................................................................    4    1    seq
    ....................ugaaagacauggguaguC.......................................................................    6    1    seq
    ....................Ggaaagacauggguagug.......................................................................    3    1    seq
    ....................ugaaagUcauggguagug.......................................................................    1    1    seq
    ....................ugaaagacauggguaguU.......................................................................    14    1    seq
    ....................uAaaagacauggguagug.......................................................................    1    1    seq
    ....................ugaaagacauggguagug.......................................................................    1068    0    seq
```

Mature			
cagaaucgguguucccugagugaaagacauggguagugagauuugacuaucacaaaacaaucucacuauucuguuuuuccccugguggauacuuauuacaucuacuacc			
....................ugaaagacaCggguaguga..	15	1	seq
....................ugaaagacauggguagugC..	27	1	seq
....................ugaaagacauggguagGga..	7	1	seq
....................Ggaaagacauggguaguga..	14	1	seq
....................uUaaagacauggguaguga..	17	1	seq
....................ugaGagacauggguaguga..	23	1	seq
....................ugaaagaAauggguaguga..	1	1	seq
....................ugaaagacaAggguaguga..	4	1	seq
....................ugaaagacauggguagugG..	136	1	seq
....................ugaaagaUauggguaguga..	7	1	seq
....................ugGaagacauggguaguga..	22	1	seq
....................ugUaagacauggguaguga..	18	1	seq
....................ugaUagacauggguaguga..	4	1	seq
....................ugaaagacaugUguaguga..	2	1	seq
....................ugaaagacauUgguaguga..	1	1	seq
....................ugaaagacGuggguaguga..	20	1	seq
....................ugaaaAacauggguaguga..	1	1	seq
....................ugaaagacauggguGguga..	18	1	seq
....................ugaaagacauggguUguga..	2	1	seq
....................ugaaagacUuggguaguga..	4	1	seq
....................ugaaagacaugggGaguga..	5	1	seq
....................uAaaagacauggguaguga..	4	1	seq
....................ugaaagacauggguaUuga..	1	1	seq
....................ugaaagacaugggCaguga..	10	1	seq
....................ugaaagGcauggguaguga..	24	1	seq
....................uCaaagacauggguaguga..	4	1	seq
....................ugaaagacauggguagugU..	341	1	seq
....................Cgaaagacauggguaguga..	15	1	seq
....................ugaaagacauggguagCga..	13	1	seq
....................ugaaagacauggguaguUa..	6	1	seq
....................ugaaagacaGggguaguga..	1	1	seq
....................ugaaagCcauggguaguga..	1	1	seq
....................ugaaagacauggguaAuga..	3	1	seq
....................ugaaagacauggguagAga..	10	1	seq
....................ugaaUgacauggguaguga..	5	1	seq
....................Agaaagacauggguaguga..	2	1	seq
....................ugaaagacCuggguaguga..	2	1	seq
....................ugaaagacauggguaguga..	4323	0	seq
....................ugaaagacauAgguaguga..	8	1	seq
....................ugaaagaGauggguaguga..	1	1	seq
....................ugaaagacaugAguaguga..	11	1	seq
....................ugaaagacaugggAaguga..	9	1	seq
....................ugaaagacauggAuaguga..	4	1	seq
....................ugaaagacauggUuaguga..	1	1	seq
....................ugaaagacauggguaguAa..	5	1	seq
....................ugaaGgacauggguaguga..	27	1	seq
....................ugaaagUcauggguaguga..	2	1	seq
....................ugaaagacauggguaguCag...	1	1	seq
....................ugaaaCacauggguagugag...	1	1	seq
....................ugaCagacauggguagugag...	2	1	seq
....................ugaaagacauggguaguAag...	1	1	seq
....................ugaaagacauggUuagugag...	2	1	seq
....................ugaaagacauggAuagugag...	3	1	seq
....................ugaaagacUuggguagugag...	5	1	seq
....................ugaaagGcauggguagugag...	23	1	seq
....................Ggaaagacauggguagugag...	10	1	seq
....................ugaaUgacauggguagugag...	1	1	seq
....................ugaaagacauggguagAgag...	5	1	seq
....................ugaaagacauggguagGgag...	3	1	seq
....................ugaaagacauggguagugaC...	39	1	seq
....................ugUaagacauggguagugag...	8	1	seq
....................ugaaagacaugggCagugag...	8	1	seq
....................ugaaagacaugAguagugag...	4	1	seq
....................ugaaagacauggguagugGg...	27	1	seq
....................ugaaagacauggguagugaU...	349	1	seq
....................ugaaagaUauggguagugag...	4	1	seq
....................ugaGagacauggguagugag...	23	1	seq
....................ugGaagacauggguagugag...	19	1	seq
....................ugaaagacauggguaAugag...	4	1	seq
....................ugaaagacauggguGgugag...	10	1	seq

```
                    Mature                   Star
cagaaucggguguucccugagugaaagacauggguagugagauuugacuaucacaaaacaaucucacuauucuguuuuuccccugguggauacuuauuacaucuacuacc
....................ugaUagacauggguagugag.....................................................................  5    1  seq
....................uUaaagacauggguagugag.....................................................................  19   1  seq
....................ugaaGgacauggguagugag.....................................................................  26   1  seq
....................Cgaaagacauggguagugag.....................................................................  13   1  seq
....................ugaaagacGuggguagugag.....................................................................  20   1  seq
....................ugaaagacauggguagugUg.....................................................................  2    1  seq
....................ugaaagacaugUguagugag.....................................................................  2    1  seq
....................ugaaagacauggguagCgag.....................................................................  10   1  seq
....................uAaaagacauggguagugag.....................................................................  3    1  seq
....................ugaaagacauggCuagugag.....................................................................  1    1  seq
....................ugaaagacauggguagugCg.....................................................................  2    1  seq
....................ugaaagacauAgguagugag.....................................................................  6    1  seq
....................ugaaagacaugggAagugag.....................................................................  11   1  seq
....................ugaaagacauggguagugaA.....................................................................  446  1  seq
....................ugaaagacauggguaguUag.....................................................................  4    1  seq
....................ugaaagacauggguUgugag.....................................................................  4    1  seq
....................ugaaagacaugggGagugag.....................................................................  2    1  seq
....................ugaaagacauggAuagugaga....................................................................  48   1  seq
....................ugaUagacauggguagugaga....................................................................  75   1  seq
....................ugaaagaUauggguagugaga....................................................................  45   1  seq
....................ugaCagacauggguagugaga....................................................................  5    1  seq
....................ugaaagacaugUguagugaga....................................................................  14   1  seq
....................ugaaagacauggCuagugaga....................................................................  8    1  seq
....................ugaaagacauggguagugUga....................................................................  78   1  seq
....................ugaaagacaugAguagugaga....................................................................  86   1  seq
....................ugaaagacauggguagGgaga....................................................................  34   1  seq
....................ugaaagacauCgguagugaga....................................................................  4    1  seq
....................ugaaaUacauggguagugaga....................................................................  9    1  seq
....................ugaaagacaugggAagugaga....................................................................  304  1  seq
....................uAaaagacauggguagugaga....................................................................  58   1  seq
....................ugaaagacaGggguagugaga....................................................................  21   1  seq
....................ugaaagacauggguaUugaga....................................................................  7    1  seq
....................ugaaagacauggguagugaCa....................................................................  11   1  seq
....................ugaaGgacauggguagugaga....................................................................  379  1  seq
....................ugGaagacauggguagugaga....................................................................  297  1  seq
....................ugaaagacauggguaguCaga....................................................................  9    1  seq
....................ugaaagacauggguagugCga....................................................................  14   1  seq
....................ugaaagacGuggguagugaga....................................................................  278  1  seq
....................ugaaagacauggguaguUaga....................................................................  64   1  seq
....................ugaaagacauggguagugGga....................................................................  423  1  seq
....................ugaaagacauggguCgugaga....................................................................  3    1  seq
....................ugaaagacauAgguagugaga....................................................................  82   1  seq
....................ugaaagacUuggguagugaga....................................................................  43   1  seq
....................ugaaagacCuggguagugaga....................................................................  22   1  seq
....................ugaaagacauggguagAgaga....................................................................  39   1  seq
....................ugaaUgacauggguagugaga....................................................................  44   1  seq
....................ugaGagacauggguagugaga....................................................................  344  1  seq
....................uCaaagacauggguagugaga....................................................................  39   1  seq
....................ugaaaAacauggguagugaga....................................................................  26   1  seq
....................ugaaagUcauggguagugaga....................................................................  27   1  seq
....................ugaaagacauggguaCugaga....................................................................  4    1  seq
....................ugaaagacauggguagugaUa....................................................................  31   1  seq
....................ugUaagacauggguagugaga....................................................................  232  1  seq
....................ugaaagacauggguUgugaga....................................................................  30   1  seq
....................ugaaagacauUgguagugaga....................................................................  5    1  seq
....................ugaaagacauggguagugaAa....................................................................  41   1  seq
....................uUaaagacauggguagugaga....................................................................  263  1  seq
....................ugaaagacauggguGgugaga....................................................................  196  1  seq
....................ugaaagacaugCguagugaga....................................................................  2    1  seq
....................ugaaagacaugggGagugaga....................................................................  83   1  seq
....................ugaaagacauggguaguAaga....................................................................  34   1  seq
....................ugCaagacauggguagugaga....................................................................  18   1  seq
....................ugaaagacauggguaAugaga....................................................................  52   1  seq
....................ugaaagCcauggguagugaga....................................................................  4    1  seq
....................ugaaaCacauggguagugaga....................................................................  1    1  seq
....................ugaaCgacauggguagugaga....................................................................  8    1  seq
....................ugaaagacauggUuagugaga....................................................................  11   1  seq
....................ugaaagaGauggguagugaga....................................................................  9    1  seq
....................ugaaagacauggguagCgaga....................................................................  152  1  seq
....................ugaaagacaugggCagugaga....................................................................  172  1  seq
```

```
                    Mature                      Star
cagaaucgguguucccugagugaaagacauggguagugagauuugacuaucacaaaacaaucucacuauucuguuuuuccccugguggauacuuauuacaucuacuacc
....................ugaaagGcauggguagugaga....................................................................    292    1    seq
....................ugaaagaAauggguagugaga....................................................................    10     1    seq
....................ugaaagacCuggguagugagau...................................................................    15     1    seq
....................ugaaagacauggguaCugagau...................................................................    4      1    seq
....................ugaaagacauAgguagugagau...................................................................    53     1    seq
....................ugaaagacauCgguagugagau...................................................................    2      1    seq
....................ugaaagacauggguaguUagau...................................................................    64     1    seq
....................ugaaagacaugCguagugagau...................................................................    1      1    seq
....................ugUaagacauggguagugagau...................................................................    184    1    seq
....................ugaaagacauggguUgugagau...................................................................    22     1    seq
....................ugaaagacaugAguagugagau...................................................................    59     1    seq
....................ugaaagGcauggguagugagau...................................................................    246    1    seq
....................ugaaagacauggCuagugagau...................................................................    4      1    seq
....................ugaaagacaugggCagugagau...................................................................    131    1    seq
....................ugaaGgacauggguagugagau...................................................................    347    1    seq
....................ugaaagCcauggguagugagau...................................................................    12     1    seq
....................ugaaagacauggguagugaAau...................................................................    25     1    seq
....................ugaaagUcauggguagugagau...................................................................    20     1    seq
....................ugaaagacauggguagGgagau...................................................................    45     1    seq
....................ugaaagacauggguaAugagau...................................................................    49     1    seq
....................ugaaagacauggguagugCgau...................................................................    4      1    seq
....................ugaaagacauggguaguAagau...................................................................    34     1    seq
....................ugaaagaUauggguagugagau...................................................................    37     1    seq
....................ugCaagacauggguagugagau...................................................................    10     1    seq
....................ugaUagacauggguagugagau...................................................................    55     1    seq
....................ugaGagacauggguagugagau...................................................................    250    1    seq
....................ugaaagacauggUuagugagau...................................................................    9      1    seq
....................ugaaagacauggAuagugagau...................................................................    47     1    seq
....................ugaaaAacauggguagugagau...................................................................    27     1    seq
....................ugaaagacauggguagCgagau...................................................................    148    1    seq
....................ugGaagacauggguagugagau...................................................................    198    1    seq
....................ugaaagacaGggguagugagau...................................................................    15     1    seq
....................ugaaagacaugggAagugagau...................................................................    163    1    seq
....................ugaaagacauggguagugUgau...................................................................    64     1    seq
....................ugaaagacauggguCgugagau...................................................................    10     1    seq
....................ugaaagacauggguaUugagau...................................................................    6      1    seq
....................ugaaCgacauggguagugagau...................................................................    7      1    seq
....................ugaaagaGauggguagugagau...................................................................    5      1    seq
....................ugaaagacauggguaguCagau...................................................................    6      1    seq
....................ugaaaUacauggguagugagau...................................................................    12     1    seq
....................ugaaagaAauggguagugagau...................................................................    5      1    seq
....................ugaaagacauggguagugaUau...................................................................    26     1    seq
....................ugaaUgacauggguagugagau...................................................................    25     1    seq
....................ugaaagacauUgguagugagau...................................................................    16     1    seq
....................ugaaaCacauggguagugagau...................................................................    1      1    seq
....................ugaaagacauggguagAgagau...................................................................    70     1    seq
....................ugaaagacauggguGgugagau...................................................................    162    1    seq
....................ugaCagacauggguagugagau...................................................................    7      1    seq
....................ugaaagacUuggguagugagau...................................................................    28     1    seq
....................ugaaagacauggguagugaCau...................................................................    4      1    seq
....................ugaaagacaugggGagugagau...................................................................    34     1    seq
....................ugaaagacGuggguagugagau...................................................................    240    1    seq
....................ugaaagacaugUguagugagau...................................................................    6      1    seq
....................ugaaagacauggguagugGgau...................................................................    249    1    seq
....................ugaaagacauUgguagugagauu..................................................................    4      1    seq
....................ugaaagaGauggguagugagauu..................................................................    1      1    seq
....................ugaaagacauggguCgugagauu..................................................................    2      1    seq
....................ugaaagacauAgguagugagauu..................................................................    19     1    seq
....................ugaaagacaGggguagugagauu..................................................................    3      1    seq
....................ugaaagacaugAguagugagauu..................................................................    16     1    seq
....................ugaaagaUauggguagugagauu..................................................................    9      1    seq
....................ugaaagCcauggguagugagauu..................................................................    1      1    seq
....................ugaaagacauggguagGgagauu..................................................................    6      1    seq
....................ugaaagUcauggguagugagauu..................................................................    3      1    seq
....................ugaaagacaugggAagugagauu..................................................................    38     1    seq
....................ugaaagacaugggGagugagauu..................................................................    10     1    seq
....................ugaaagacauggguaUugagauu..................................................................    2      1    seq
....................ugaaagacUuggguagugagauu..................................................................    13     1    seq
....................ugaaagacauggCuagugagauu..................................................................    2      1    seq
....................ugaaagacaugggCagugagauu..................................................................    46     1    seq
```

```
                    Mature                                       Star
cagaaucgguguucccugagugaaagacauggguagugagauuugacuaucacaaaacaaucucacuauucuguuuuuccccugguggauacuuauuacaucuacuacc
....................ugaaagacauggguGgugagauu..................................................................  49  1  seq
....................ugaaagacauggguagAgagauu..................................................................  17  1  seq
....................ugaaagacauggguagCgagauu..................................................................  45  1  seq
....................ugaaagGcauggguagugagauu..................................................................  72  1  seq
....................ugaaagacGuggguagugagauu..................................................................  57  1  seq
....................ugaaagaAauggguagugagauu..................................................................  1   1  seq
....................ugaaagacauggguUgugagauu..................................................................  6   1  seq
....................ugaaagacCuggguagugagauu..................................................................  1   1  seq
....................ugaaagacauggAuagugagauu..................................................................  8   1  seq
....................ugaaagacaugUguagugagauu..................................................................  5   1  seq
....................ugaaagacauggguaAugagauu..................................................................  14  1  seq
....................ugaaagacauggUuagugagauu..................................................................  3   1  seq
....................ugaaagacaugCguagugagauu..................................................................  2   1  seq
....................ugaaagacGuggguagugagauuu.................................................................  1   1  seq
....................ugaaagGcauggguagugagauuu.................................................................  2   1  seq
....................ugaaagacauggguGgugagauuu.................................................................  1   1  seq
....................ugaaagacUuggguagugagauuu.................................................................  1   1  seq
....................ugaaagacauggAuagugagauuu.................................................................  1   1  seq
....................ugaaagacaugggAagugagauuu.................................................................  2   1  seq
....................ugaaagacaugAguagugagauuu.................................................................  2   1  seq
.....................gaaagacauggguagugagU....................................................................  1   1  seq
.....................Uaaagacauggguagugaga....................................................................  6   1  seq
.....................gaaagacauggguaUugagau...................................................................  1   1  seq
.......................aagacauggguagugaga....................................................................  1   0  seq
```
