# Supplementary material for: Identification and Characterization of MicroRNAs from Longitudinal Muscle and Respiratory Tree in Sea Cucumber (Apostichopus japonicus) Using High-Throughput Sequencing
Source: PLoS One. 2015 Aug 5;10(8):e0134899. doi: 10.1371/journal.pone.0134899 (PMC4526669; doi:10.1371/journal.pone.0134899)
Supplement: S1 File — (ZIP) [file pone.0134899.s002.zip › S1 File/The secondary structures of the novel miRNAs in LTM/Scaffold360_346.pdf]

Provisional ID : Scaffold360_346
Score total : 2.4
Score for star read(s) : -1.3
Score for read counts : 0
Score for mfe : 2.1
Score for randfold : 1.6
Score for cons. seed :
Total read count : 203
Mature read count : 203
Loop read count : 0
Star read count : 0

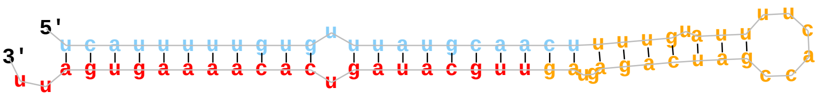

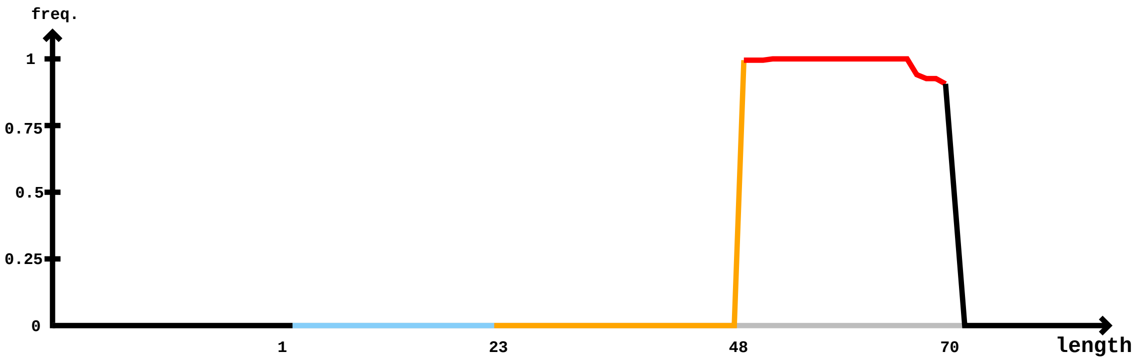


```
                         Star                                       Mature
5'- auccaucauucccgccagcccgaucauuuuuguguuuaugcaacuuuuguauuuucaccgaucagaguaguugcauagucacaaaagugauugggaggguugggcucu   -3'    exp
    ..........((((((..((((((((((((((((.((((((((((((((.(((......)))))))..)))))))))).))))))))))))))))..)).))))....   reads   mm   sample
    ......................................................................uugcauagucacaaaagC....................   1       1    seq
    ......................................................................uugcGuagucacaaaagu....................   1       1    seq
    ......................................................................uugcauagucacaaaagu....................   10      0    seq
    ......................................................................uugcauagucacaaaagug...................   3       0    seq
    ......................................................................uugUauagucacaaaagugau.................   1       1    seq
    ......................................................................uugcauagucacaCaagugau.................   1       1    seq
    ......................................................................uugcGuagucacaaaagugau.................   1       1    seq
    ......................................................................uugcauagucacaaaGgugau.................   1       1    seq
    ......................................................................uugcUuagucacaaaagugauu................   1       1    seq
    ......................................................................uugcauaguAacaaaagugauu................   2       1    seq
    ......................................................................uugcGuagucacaaaagugauu................   10      1    seq
    ......................................................................uugcauagucacUaaagugauu................   1       1    seq
    ......................................................................uugcauagucacaaaagAgauu................   7       1    seq
    ......................................................................uugcauagucGcaaaagugauu................   10      1    seq
    ......................................................................uugcauCgucacaaaagugauu................   1       1    seq
    ......................................................................uugcauagucacaaaaguAauu................   1       1    seq
    ......................................................................uugcauagucacaaUagugauu................   1       1    seq
    ......................................................................uugcauaguUacaaaagugauu................   4       1    seq
    ......................................................................uugcauagucacaGaagugauu................   13      1    seq
    ......................................................................uugcauagucacaaaagugGuu................   5       1    seq
    ......................................................................uugcauagucacaaaGgugauu................   5       1    seq
    ......................................................................uuCcauagucacaaaagugauu................   1       1    seq
    ......................................................................uugcauagucacaaaagugUuu................   2       1    seq
    ......................................................................uugcauagucacGaaagugauu................   5       1    seq
    ......................................................................uugcauagucacCaaagugauu................   1       1    seq
    ......................................................................uugcauGgucacaaaagugauu................   27      1    seq
    ......................................................................uugUauagucacaaaagugauu................   5       1    seq
    ......................................................................uugcauagAcacaaaagugauu................   7       1    seq
    ......................................................................uugcauagucacaCaagugauu................   1       1    seq
    ......................................................................uugcauagucacaaaaguUauu................   2       1    seq
    ......................................................................uugcaCagucacaaaagugauu................   5       1    seq
    ......................................................................uugGauagucacaaaagugauu................   4       1    seq
    ......................................................................uuUcauagucacaaaagugauu................   7       1    seq
    ......................................................................uugcauagucaUaaaagugauu................   3       1    seq
```

```
                       Star                                        Mature
auccaucauucccgccagcccgaucauuuuuguguuuaugcaacuuuuguauuuucaccgaucagaguaguugcauagucacaaaagugauugggaggguugggcucu
......................................................................uugcauagucacaaaagGgauu................   5   1   seq
......................................................................uugcauagucacaaaagCgauu................   17  1   seq
......................................................................uugcauaAucacaaaagugauu................   1   1   seq
......................................................................uugcauagucacaaGagugauu................   15  1   seq
......................................................................uugcauagucacaUaagugauu................   1   1   seq
......................................................................uugcauagCcacaaaagugauu................   10  1   seq
......................................................................uuAcauagucacaaaagugauu................   3   1   seq
.........................................................................cauagucacaaaagugauu................   1   0   seq
```
