# Supplementary material for: Identification and Characterization of MicroRNAs from Longitudinal Muscle and Respiratory Tree in Sea Cucumber (Apostichopus japonicus) Using High-Throughput Sequencing
Source: PLoS One. 2015 Aug 5;10(8):e0134899. doi: 10.1371/journal.pone.0134899 (PMC4526669; doi:10.1371/journal.pone.0134899)
Supplement: S1 File — (ZIP) [file pone.0134899.s002.zip › S1 File/The secondary structures of the novel miRNAs in LTM/Scaffold365_353.pdf]

| | | |
|---|---|---|
| Provisional ID | : | Scaffold365_353 |
| Score total | : | 1.7 |
| Score for star read(s) | : | -1.3 |
| Score for read counts | : | 0 |
| Score for mfe | : | 1.4 |
| Score for randfold | : | 1.6 |
| Score for cons. seed | : | |
| Total read count | : | 152 |
| Mature read count | : | 152 |
| Loop read count | : | 0 |
| Star read count | : | 0 |

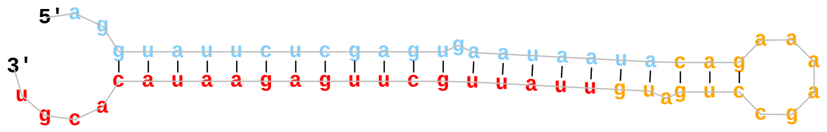


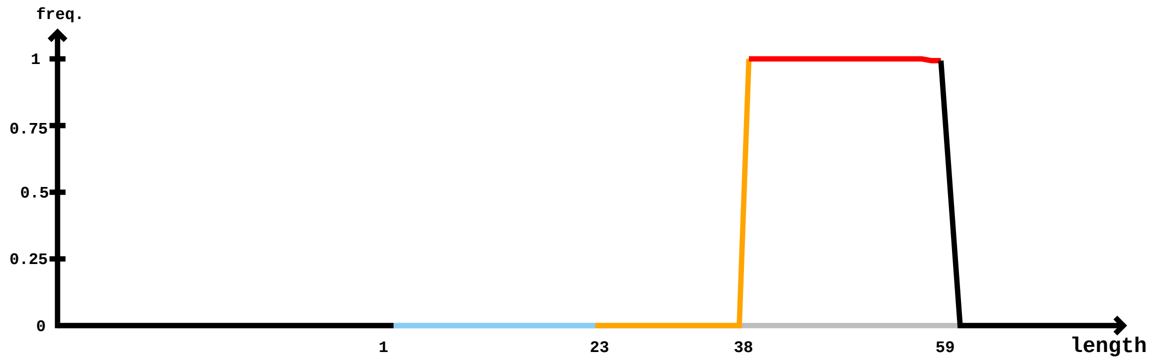


Star Mature

```
5'- uccuagucuucacuauccagaccucaugauuacagguauucucgagugaauaaauacagaaaagccugauguuauugcuugagaauacacguaguauacgggucacucac   -3'    exp
    ...((((....))))....(((((....(((((..((((((((((((.(((((((((((......))).)))))))))))))))))))..)))))....))))))......   reads      mm         sample
    .......................................................................uuauugcuugagaauacac....................   1          0          seq
    .......................................................................uuauugcuugGgaauacacgu..................   3          1          seq
    .......................................................................uuauugcuugagaaAacacgu..................   1          1          seq
    .......................................................................uCauugcuugagaauacacgu..................   5          1          seq
    .......................................................................uuauugcuugagUauacacgu..................   1          1          seq
    .......................................................................uuauugcuugagaauacaUgu..................   3          1          seq
    .......................................................................uuauugcuugagaGuacacgu..................   4          1          seq
    .......................................................................uuGuugcuugagaauacacgu..................   4          1          seq
    .......................................................................uuauugcuugCgaauacacgu..................   1          1          seq
    .......................................................................uuauugcuugagaauGcacgu..................   6          1          seq
    .......................................................................uuauugcuugaUaauacacgu..................   2          1          seq
    .......................................................................uuauugcuugagaauaGacgu..................   2          1          seq
    .......................................................................uuauugcuugagaauaUacgu..................   3          1          seq
    .......................................................................uuauCgcuugagaauacacgu..................   6          1          seq
    .......................................................................uuauugcuugagaauacaAgu..................   1          1          seq
    .......................................................................uuauuAcuugagaauacacgu..................   4          1          seq
    .......................................................................uuaAugcuugagaauacacgu..................   3          1          seq
    .......................................................................uuUuugcuugagaauacacgu..................   2          1          seq
    .......................................................................uuauAgcuugagaauacacgu..................   1          1          seq
    .......................................................................uuauugcuugagaaCacacgu..................   3          1          seq
    .......................................................................uGauugcuugagaauacacgu..................   3          1          seq
    .......................................................................uuauugcuugagGauacacgu..................   8          1          seq
    .......................................................................uuaCugcuugagaauacacgu..................   11         1          seq
    .......................................................................uuauugcuugagaauacacUu..................   1          1          seq
    .......................................................................uuauugcuugagaauacacAu..................   1          1          seq
    .......................................................................uuauugcGugagaauacacgu..................   1          1          seq
    .......................................................................uuauGgcuugagaauacacgu..................   1          1          seq
    .......................................................................uuauugcuugUgaauacacgu..................   2          1          seq
    .......................................................................uuauuUcuugagaauacacgu..................   1          1          seq
    .......................................................................uuauugcuugagaauacUcgu..................   1          1          seq
    .......................................................................uuauugcuugaAaauacacgu..................   1          1          seq
    .......................................................................uuaGugcuugagaauacacgu..................   7          1          seq
    .......................................................................uuauugcuCgagaauacacgu..................   3          1          seq
    .......................................................................uuauugcCugagaauacacgu..................   2          1          seq
```

```
                                Star                                Mature
uccuagucuucacuauccagaccucaugauuacagguauucucgagugaauaauacagaaaagccugauguuauugcuugagaauacacguaguauacgggucacucac
......................................................................uuauugcuugagaauacacAua.................     2     1     seq
......................................................................uuauugUuugagaauacacgua.................     1     1     seq
......................................................................uuauugcuugagaauacacUua.................     1     1     seq
......................................................................uuauugcuugGgaauacacgua.................     3     1     seq
......................................................................uuauCgcuugagaauacacgua.................     4     1     seq
......................................................................uuauugcuugagaauaAacgua.................     2     1     seq
......................................................................uuaGugcuugagaauacacgua.................     5     1     seq
......................................................................uuaCugcuugagaauacacgua.................     3     1     seq
......................................................................uuauugcuugagaauacaGgua.................     1     1     seq
......................................................................uuauugcuugCgaauacacgua.................     1     1     seq
......................................................................uuauugcuugagaGuacacgua.................     3     1     seq
......................................................................uuUuugcuugagaauacacgua.................     3     1     seq
......................................................................uuGuugcuugagaauacacgua.................     2     1     seq
......................................................................uuauuAcuugagaauacacgua.................     1     1     seq
......................................................................uuauugcuugagGauacacgua.................     5     1     seq
......................................................................uuauugcuugagaaCacacgua.................     2     1     seq
......................................................................uuaAugcuugagaauacacgua.................     2     1     seq
......................................................................uuauugcuugagaauacCcgua.................     1     1     seq
......................................................................uuauugcuCgagaauacacgua.................     2     1     seq
......................................................................uuauugcCugagaauacacgua.................     4     1     seq
......................................................................uuauuUcuugagaauacacgua.................     1     1     seq
......................................................................uuauugcuugagaauGcacgua.................     2     1     seq
......................................................................uuauugcuugagaauacUcgua.................     2     1     seq
```
