# Supplementary material for: Identification and Characterization of MicroRNAs from Longitudinal Muscle and Respiratory Tree in Sea Cucumber (Apostichopus japonicus) Using High-Throughput Sequencing
Source: PLoS One. 2015 Aug 5;10(8):e0134899. doi: 10.1371/journal.pone.0134899 (PMC4526669; doi:10.1371/journal.pone.0134899)
Supplement: S1 File — (ZIP) [file pone.0134899.s002.zip › S1 File/The secondary structures of the novel miRNAs in LTM/Scaffold3711_1014.pdf]

Provisional ID	:	Scaffold3711_1014
Score total	:	103.7
Score for star read(s)	:	3.9
Score for read counts	:	96.5
Score for mfe	:	1.7
Score for randfold	:	1.6
Score for cons. seed	:	
Total read count	:	201
Mature read count	:	163
Loop read count	:	0
Star read count	:	38

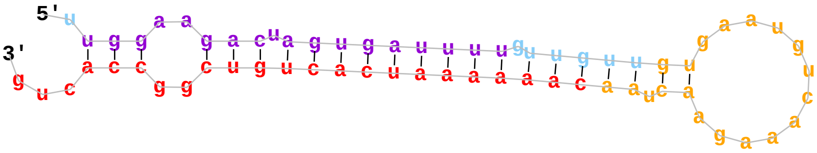

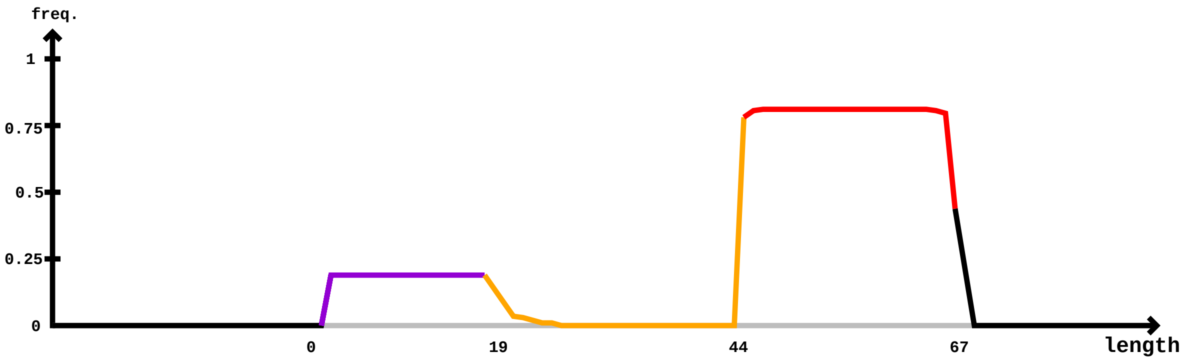


```
                                      Star                                          Mature
5'- caaaaaagucacauaccucuggcccuuuggaagacuagugauuuuguuguugugaaugucaaagaacuaacaaaaaaucacugucggccacuggggacagagguccauucauc  -3'  obs
    caaaaaagucacauaccucuggcccuuuggaagacuagugauuuuguuguugugaaugucaaagaacuaacaaaaaaucacugucggccacuggggacagagguccauucauc       exp
    ..............(((((((.((((.(((..(((.(((((((((.(((((((............)).)))))))))))))))))..)))..)))).)))))))......... reads   mm   sample
    ...........................uggaagacuGgugauuuu.................................................................... 31      1    seq
    ...........................uggaagacuagugauuuuUu.................................................................. 1       1    seq
    ...........................uggaaAacuagugauuuuguu................................................................. 1       1    seq
    ...........................uggaagGcuagugauuuuguu................................................................. 1       1    seq
    ...........................uggaagacuagGgauuuuguug................................................................ 1       1    seq
    ...........................uggaGgacuagugauuuuguug................................................................ 1       1    seq
    ...........................uggaagacuagugaCuuuguuguu.............................................................. 1       1    seq
    ...........................uggaagaGuagugauuuuguuguu.............................................................. 1       1    seq
    ......................................................................caaaaaaucacAgucggcca....................... 1       1    seq
    ......................................................................caaaaaaucacAgucggccacu..................... 70      1    seq
    ......................................................................caaaaaaucacugucggccacug.................... 1       0    seq
    ......................................................................caaaaaaucacGgucggccacug.................... 1       1    seq
    ......................................................................caaaaaaucacAgucggccacug.................... 84      1    seq
    .......................................................................aaaaaaucacAgucggccac...................... 2       1    seq
    .......................................................................aaaaaaucacAgucggccacu..................... 1       1    seq
    .......................................................................aaaaaaucacAgucggccacug.................... 2       1    seq
    ........................................................................aaaaaucacAgucggccacu..................... 1       1    seq
```
