# Supplementary material for: Identification and Characterization of MicroRNAs from Longitudinal Muscle and Respiratory Tree in Sea Cucumber (Apostichopus japonicus) Using High-Throughput Sequencing
Source: PLoS One. 2015 Aug 5;10(8):e0134899. doi: 10.1371/journal.pone.0134899 (PMC4526669; doi:10.1371/journal.pone.0134899)
Supplement: S1 File — (ZIP) [file pone.0134899.s002.zip › S1 File/The secondary structures of the novel miRNAs in LTM/Scaffold391_371.pdf]

Provisional ID	:	Scaffold391_371
Score total	:	2.2
Score for star read(s)	:	-1.3
Score for read counts	:	0
Score for mfe	:	1.9
Score for randfold	:	1.6
Score for cons. seed	:	
Total read count	:	3404
Mature read count	:	3404
Loop read count	:	0
Star read count	:	0

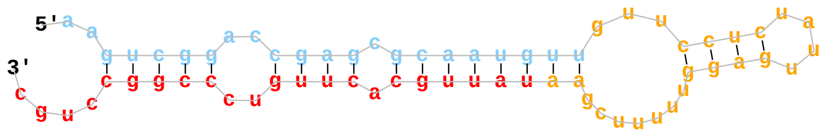

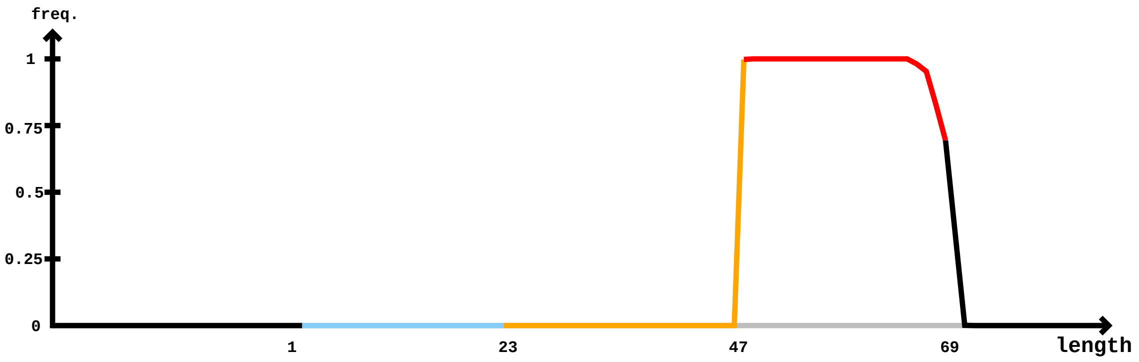

Star   Mature

```
5'- ucaucaccauggugauugauggucaagucggaccgagcgcaauguuguuccucuauugagguuuuucgaauauugcacuugucccggccugcucaucaacaccaaaau  -3'   exp
    .........(((((.(((((((.((.(((((..((((.((((((((...((((....)))).......)))))))).))))..))))).)).))))))))))))....   reads   mm   sample
    ......................................................................uauugcGcuugucccggc....................   1       1    seq
    ......................................................................uauugcacuugucccggc....................   32      0    seq
    ......................................................................uauugcacuuUucccggc....................   6       1    seq
    ......................................................................uauugcacuugAcccggc....................   5       1    seq
    ......................................................................uauugcacuugucccggA....................   1       1    seq
    ......................................................................uauugcacuugucccggU....................   20      1    seq
    ......................................................................uauugcacuugucccggcU...................   25      1    seq
    ......................................................................uauugcacuugucccggcG...................   1       1    seq
    ......................................................................uauugcacuugucccggcA...................   8       1    seq
    ......................................................................uauugcacuugucccggcc...................   40      0    seq
    ......................................................................uauugcacuuUucccggcc...................   8       1    seq
    ......................................................................uaAugcacuugucccggcc...................   1       1    seq
    ......................................................................uauugUacuugucccggcc...................   1       1    seq
    ......................................................................uauugcacuugAcccggcc...................   11      1    seq
    ......................................................................uauugcacuugucccggccA..................   43      1    seq
    ......................................................................Gauugcacuugucccggccu..................   4       1    seq
    ......................................................................uauugcacuugAcccggccu..................   143     1    seq
    ......................................................................uGuugcacuugucccggccu..................   1       1    seq
    ......................................................................uauugcacuugGcccggccu..................   3       1    seq
    ......................................................................uauugcacuuUucccggccu..................   85      1    seq
    ......................................................................uaCugcacuugucccggccu..................   2       1    seq
    ......................................................................uauugcacuugCcccggccu..................   2       1    seq
    ......................................................................uauugcacuugucccggccG..................   85      1    seq
    ......................................................................uauugcacuuguccGggccu..................   1       1    seq
    ......................................................................uauGgcacuugucccggccu..................   1       1    seq
    ......................................................................uauugcacuugucccggccC..................   41      1    seq
    ......................................................................uauugcacuuCucccggccu..................   2       1    seq
    ......................................................................uauugcaUuugucccggccu..................   1       1    seq
    ......................................................................uauugcacuAgucccggccu..................   2       1    seq
    ......................................................................uaGugcacuugucccggccu..................   3       1    seq
    ......................................................................uauugcacCugucccggccu..................   2       1    seq
    ......................................................................uauugcacuuguccUggccu..................   1       1    seq
    ......................................................................uauCgcacuugucccggccug.................   1       1    seq
    ......................................................................uauugcacuugucccggccGg.................   326     1    seq
```

Star Mature

```
ucaucaccauggugauugauggucaagucggaccgagcgcaauguuguuccucuauugagguuuuucgaauauugcacuugucccggccugcucaucaacaccaaaau
......................................................................uauugcacuuCucccggccug.................   1     1   seq
......................................................................uauugcacuugucccggccCg.................   3     1   seq
......................................................................uauugcacuugucccggccAg.................   2     1   seq
......................................................................uaGugcacuugucccggccug.................   1     1   seq
......................................................................uauuAcacuugucccggccug.................   2     1   seq
......................................................................uauGgcacuugucccggccug.................   1     1   seq
......................................................................uauugcacuugAcccggccug.................   48    1   seq
......................................................................uauugcacuugGcccggccug.................   1     1   seq
......................................................................uauugcacuuUucccggccug.................   69    1   seq
......................................................................uauugcGcuugucccggccug.................   1     1   seq
......................................................................uauugcacGugucccggccugc................   1     1   seq
......................................................................uauugcaUuugucccggccugc................   4     1   seq
......................................................................uauugcacuugucccggcGugc................   1     1   seq
......................................................................uauugcacuugucccgAccugc................   1     1   seq
......................................................................uauugcacuugAcccggccugc................   934   1   seq
......................................................................uauAgcacuugucccggccugc................   1     1   seq
......................................................................uauugcacuuguccUggccugc................   5     1   seq
......................................................................uauugcacCugucccggccugc................   3     1   seq
......................................................................uauGgcacuugucccggccugc................   11    1   seq
......................................................................uauugcacuugucccggccGgc................   300   1   seq
......................................................................uauugcacuugucUcggccugc................   3     1   seq
......................................................................uauugcacuGgucccggccugc................   2     1   seq
......................................................................uauuUcacuugucccggccugc................   1     1   seq
......................................................................uauugcacuuguccGggccugc................   3     1   seq
......................................................................uauugcacuugucccggccAgc................   1     1   seq
......................................................................uauugcacuugucccgUccugc................   1     1   seq
......................................................................uaGugcacuugucccggccugc................   5     1   seq
......................................................................uauugcacuuCucccggccugc................   5     1   seq
......................................................................uauugUacuugucccggccugc................   3     1   seq
......................................................................uauugcacuuAucccggccugc................   1     1   seq
......................................................................uauugcacuugucccggccCgc................   2     1   seq
......................................................................uauugcacuuguUccggccugc................   3     1   seq
......................................................................uauugcacuuUucccggccugc................   766   1   seq
......................................................................uauugcUcuugucccggccugc................   2     1   seq
......................................................................uauCgcacuugucccggccugc................   11    1   seq
......................................................................uauuAcacuugucccggccugc................   5     1   seq
......................................................................uauugAacuugucccggccugc................   3     1   seq
......................................................................uauugcGcuugucccggccugc................   6     1   seq
......................................................................uaAugcacuugucccggccugc................   3     1   seq
......................................................................uauugcacuugCcccggccugc................   7     1   seq
......................................................................uaCugcacuugucccggccugc................   12    1   seq
......................................................................uauugcacuuAucccggccugcu...............   1     1   seq
......................................................................uauugcacuugAcccggccugcu...............   172   1   seq
......................................................................uauugcacuuguccGggccugcu...............   1     1   seq
......................................................................uauugcUcuugucccggccugcu...............   1     1   seq
......................................................................uauugcacuuUucccggccugcu...............   58    1   seq
......................................................................uauugcaUuugucccggccugcu...............   1     1   seq
......................................................................uauugcacuugucccggGcugcu...............   1     1   seq
......................................................................uauugcacuugucccggccCgcu...............   3     1   seq
......................................................................uauugcacuuguUccggccugcu...............   1     1   seq
......................................................................uauGgcacuugucccggccugcu...............   3     1   seq
......................................................................uauuAcacuugucccggccugcu...............   1     1   seq
......................................................................uauCgcacuugucccggccugcu...............   1     1   seq
......................................................................uauugcacuugGcccggccugcu...............   1     1   seq
......................................................................uauugcacuugCcccggccugcu...............   2     1   seq
......................................................................uauugcacuugucccggccGgcu...............   1     1   seq
......................................................................uauugcacCugucccggccugcu...............   1     1   seq
......................................................................uauugcacuuguccUggccugcu...............   1     1   seq
......................................................................uauugcacuugAcccggccugcuc..............   3     1   seq
.......................................................................auugcacuugAcccggccu..................   2     1   seq
.......................................................................auugcacuuUucccggccug.................   1     1   seq
.......................................................................auugcacuuUucccggccugc................   2     1   seq
.......................................................................auugcacuugAcccggccugc................   1     1   seq
.......................................................................auugcacuugucccggccGgc................   1     1   seq
```
