# Supplementary material for: Identification and Characterization of MicroRNAs from Longitudinal Muscle and Respiratory Tree in Sea Cucumber (Apostichopus japonicus) Using High-Throughput Sequencing
Source: PLoS One. 2015 Aug 5;10(8):e0134899. doi: 10.1371/journal.pone.0134899 (PMC4526669; doi:10.1371/journal.pone.0134899)
Supplement: S1 File — (ZIP) [file pone.0134899.s002.zip › S1 File/The secondary structures of the novel miRNAs in LTM/Scaffold391_373.pdf]

Provisional ID	:	Scaffold391_373
Score total	:	2.4
Score for star read(s)	:	-1.3
Score for read counts	:	0
Score for mfe	:	2.1
Score for randfold	:	1.6
Score for cons. seed	:	
Total read count	:	380
Mature read count	:	380
Loop read count	:	0
Star read count	:	0

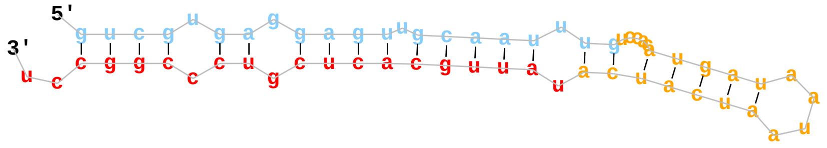

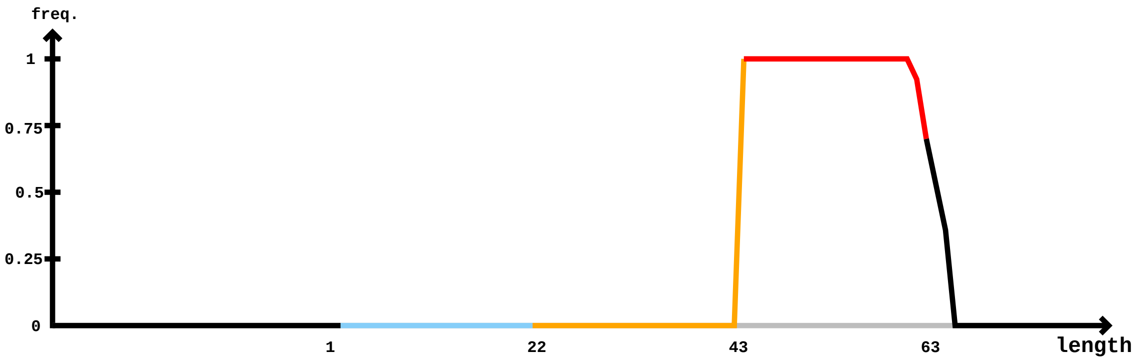

Star Mature

```
5'- uccaucaucaguagagggcagcaagcuggucgugaggaguugcaauuuguccacaugauaauaaucaucauauugcacucgucccggccugccugcuugcccucaauc   -3'   exp
    ............(((((((((.((.(((((.((.((((.(((((.((.....(((((....))))))).))))))))).)).))))).)).))).)))))))....   reads   mm   sample
    ......................................................................uauugcacucgucccggc....................   16      0    seq
    ......................................................................uauugcacucgucccggU....................   12      1    seq
    ......................................................................uauugcacucgucccggA....................   1       1    seq
    ......................................................................uauugcacucgucccggcc...................   31      0    seq
    ......................................................................uauugcacucgucccggcU...................   41      1    seq
    ......................................................................uauugcacucgucccggcA...................   9       1    seq
    ......................................................................uauugcacucgucccggcG...................   3       1    seq
    ......................................................................uauugcacAcgucccggcc...................   1       1    seq
    ......................................................................uauugcacucgucccUgccu..................   1       1    seq
    ......................................................................uauugcacucgucccggccG..................   13      1    seq
    ......................................................................uauugcacucguUccggccu..................   1       1    seq
    ......................................................................uauAgcacucgucccggccu..................   1       1    seq
    ......................................................................Gauugcacucgucccggccu..................   2       1    seq
    ......................................................................uauugcGcucgucccggccu..................   2       1    seq
    ......................................................................uauugcacucgucccggcUu..................   2       1    seq
    ......................................................................uauugcacucAucccggccu..................   1       1    seq
    ......................................................................uauugcacucgucccggUcu..................   2       1    seq
    ......................................................................uauugcacucCucccggccu..................   1       1    seq
    ......................................................................uauugcacucgucUcggccu..................   2       1    seq
    ......................................................................uGuugcacucgucccggccu..................   1       1    seq
    ......................................................................uauugcacuAgucccggccu..................   2       1    seq
    ......................................................................uauGgcacucgucccggccu..................   6       1    seq
    ......................................................................uauugcacucgucccggccC..................   67      1    seq
    ......................................................................uauugcacCgucccggccu..................   3       1    seq
    ......................................................................uaAugcacucgucccggccu..................   1       1    seq
    ......................................................................uauugcacucUucccggccu..................   1       1    seq
    ......................................................................uauugcacucgAcccggccu..................   2       1    seq
    ......................................................................uauCgcacucgucccggccu..................   1       1    seq
    ......................................................................uaGugcacucgucccggccu..................   1       1    seq
    ......................................................................uauugcacucguccUggccu..................   3       1    seq
    ......................................................................uauugcacucgucccggcUug.................   1       1    seq
    ......................................................................uauugcacucgucccggccCg.................   2       1    seq
    ......................................................................uauugcacucgucccggccGg.................   2       1    seq
    ......................................................................uauugcacucgucccAgccug.................   2       1    seq
```

Star **Mature**

```
uccaucaucaguagagggcagcaagcuggucgugaggaguugcaauuuguccacaugauaauaaucaucauauugcacucgucccggccugccugcuugcccucaauc
......................................................................uauugcacAcgucccggccug.................   1    1    seq
......................................................................uauugcacucUucccggccug.................   1    1    seq
......................................................................uauugcacucgucccUgccug.................   1    1    seq
......................................................................uaAugcacucgucccggccug.................   3    1    seq
......................................................................uauugcacucguccAggccug.................   1    1    seq
......................................................................uauugcacuGgucccggccugc................   2    1    seq
......................................................................uauugcacAcgucccggccugc................   2    1    seq
......................................................................uauugcacucAucccggccugc................   6    1    seq
......................................................................uaAugcacucgucccggccugc................   6    1    seq
......................................................................uauugcacucgucAcggccugc................   1    1    seq
......................................................................uauugcacucguUccggccugc................   2    1    seq
......................................................................uauCgcacucgucccggccugc................   11   1    seq
......................................................................uauugcGcucgucccggccugc................   16   1    seq
......................................................................uauugcacucgucccggcUugc................   2    1    seq
......................................................................uauugcacucgAcccggccugc................   5    1    seq
......................................................................uauugAacucgucccggccugc................   2    1    seq
......................................................................uauugcacucgucccAgccugc................   7    1    seq
......................................................................uauugcacucgucccggccAgc................   3    1    seq
......................................................................uaGugcacucgucccggccugc................   8    1    seq
......................................................................uauGgcacucgucccggccugc................   16   1    seq
......................................................................uauugcacucgucUcggccugc................   4    1    seq
......................................................................uauugcacucgucccgUccugc................   2    1    seq
......................................................................uauugcacucguccUggccugc................   4    1    seq
......................................................................uauugcacucgucccUgccugc................   1    1    seq
......................................................................uauugcacucgucccgAccugc................   2    1    seq
......................................................................uauugcUcucgucccggccugc................   1    1    seq
......................................................................uauugcaUucgucccggccugc................   4    1    seq
......................................................................uauugcacucgucccggccGgc................   1    1    seq
......................................................................uauAgcacucgucccggccugc................   1    1    seq
......................................................................uauugcacucguccGggccugc................   5    1    seq
......................................................................uauugcacucgucccggccCgc................   12   1    seq
......................................................................uauugcacucgucccggcGugc................   1    1    seq
......................................................................uauugcacucgucGcggccugc................   1    1    seq
......................................................................uauugcacucgucccggUcugc................   3    1    seq
......................................................................uauugcacucgucccggcAugc................   1    1    seq
......................................................................uauugcaGucgucccggccugc................   1    1    seq
......................................................................uauugcacucgucccggGcugc................   1    1    seq
......................................................................uauugcacucgGcccggccugc................   2    1    seq
```
