# Supplementary material for: Identification and Characterization of MicroRNAs from Longitudinal Muscle and Respiratory Tree in Sea Cucumber (Apostichopus japonicus) Using High-Throughput Sequencing
Source: PLoS One. 2015 Aug 5;10(8):e0134899. doi: 10.1371/journal.pone.0134899 (PMC4526669; doi:10.1371/journal.pone.0134899)
Supplement: S1 File — (ZIP) [file pone.0134899.s002.zip › S1 File/The secondary structures of the novel miRNAs in LTM/Scaffold391_375.pdf]

Provisional ID	:	Scaffold391_375
Score total	:	1.9
Score for star read(s)	:	-1.3
Score for read counts	:	0
Score for mfe	:	1.6
Score for randfold	:	1.6
Score for cons. seed	:	
Total read count	:	684
Mature read count	:	684
Loop read count	:	0
Star read count	:	0

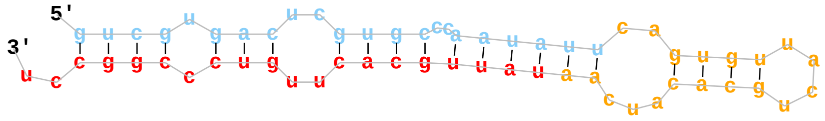

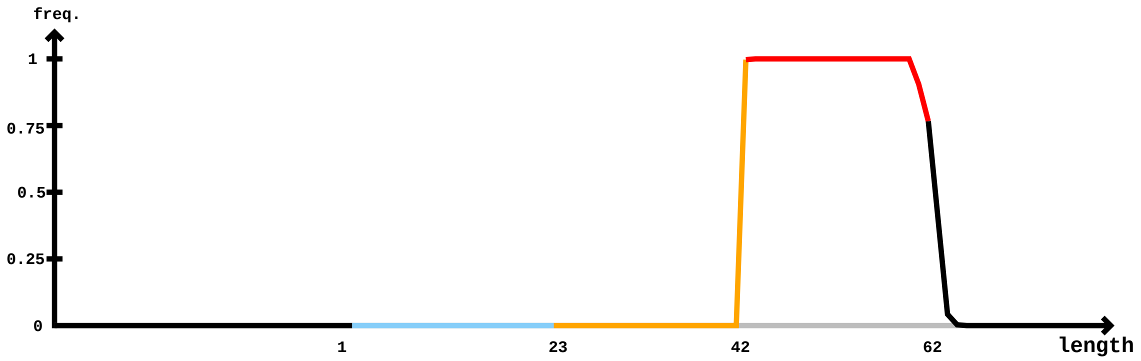


```
                                    Star                                        Mature
5'- agcuuuugucaacgaaucuugucuuguaggucgugacucgugcccaauauucaguguuacugcacaucaauauugcacuugucccggccuacuggauagguucuaauc   -3'   exp
    .............((((((.((((.((((((((.(((..((((..((((((..((((....))))...))))))))))..))).)))))))).)))))))))).....   reads   mm   sample
    ......................................................................uauugcacuugucccggc....................   32      0    seq
    ......................................................................uauugcGcuugucccggc....................   1       1    seq
    ......................................................................uauugcacuuUucccggc....................   6       1    seq
    ......................................................................uauugcacuugAcccggc....................   5       1    seq
    ......................................................................uauugcacuugucccggA....................   1       1    seq
    ......................................................................uauugcacuugucccggU....................   20      1    seq
    ......................................................................uaAugcacuugucccggcc...................   1       1    seq
    ......................................................................uauugcacuugucccggcA...................   8       1    seq
    ......................................................................uauugcacuugucccggcc...................   40      0    seq
    ......................................................................uauugUacuugucccggcc...................   1       1    seq
    ......................................................................uauugcacuugucccggcU...................   25      1    seq
    ......................................................................uauugcacuugAcccggcc...................   11      1    seq
    ......................................................................uauugcacuuUucccggcc...................   8       1    seq
    ......................................................................uauugcacuugucccggcG...................   1       1    seq
    ......................................................................uaCugcacuugucccggccu..................   2       1    seq
    ......................................................................uauugcacuuguccUggccu..................   1       1    seq
    ......................................................................uauugcaUuugucccggccu..................   1       1    seq
    ......................................................................uaGugcacuugucccggccu..................   3       1    seq
    ......................................................................uGuugcacuugucccggccu..................   1       1    seq
    ......................................................................uauugcacuuUucccggccu..................   85      1    seq
    ......................................................................uauugcacuugucccggccA..................   43      1    seq
    ......................................................................uauugcacCugucccggccu..................   2       1    seq
    ......................................................................uauugcacuuCucccggccu..................   2       1    seq
    ......................................................................uauugcacuugCcccggccu..................   2       1    seq
    ......................................................................Gauugcacuugucccggccu..................   4       1    seq
    ......................................................................uauugcacuugAcccggccu..................   143     1    seq
    ......................................................................uauGgcacuugucccggccu..................   1       1    seq
    ......................................................................uauugcacuugucccggccC..................   41      1    seq
    ......................................................................uauugcacuugucccggccG..................   85      1    seq
    ......................................................................uauugcacuuguccGggccu..................   1       1    seq
    ......................................................................uauugcacuugGcccggccu..................   3       1    seq
    ......................................................................uauugcacuAgucccggccu..................   2       1    seq
    ......................................................................uauugcacuugucUcggccua.................   1       1    seq
    ......................................................................uauCgcacuugucccggccua.................   1       1    seq
```

```
                             Star                                         Mature
agcuuuugucaacgaaucuugucuuguaggucgugacucgugcccaauauucaguguuacugcacaucaauauugcacuugucccggccuacuggauagguucuaauc
......................................................................uauugcacuuCucccggccua.................   1    1    seq
......................................................................uauugcacuuguccAggccua.................   1    1    seq
......................................................................uauugcacuugAcccggccua.................   9    1    seq
......................................................................uauugcCcuugucccggccua.................   1    1    seq
......................................................................uauugcacuuUucccggccua.................   10   1    seq
......................................................................uaGugcacuugucccggccua.................   1    1    seq
......................................................................uauAgcacuugucccggccua.................   1    1    seq
......................................................................uauugcacuugucccggccCa.................   1    1    seq
......................................................................uauugcacuugucccggccGa.................   43   1    seq
......................................................................uauGgcacuugucccggccua.................   1    1    seq
......................................................................uauCgcacuugucccggccuac................   4    1    seq
......................................................................uauGgcacuugucccggccuac................   4    1    seq
......................................................................uauugAacuugucccggccuac................   1    1    seq
......................................................................uaCugcacuugucccggccuac................   2    1    seq
......................................................................uaGugcacuugucccggccuac................   3    1    seq
......................................................................uauugcacuuUucccggccuac................   3    1    seq
......................................................................uauugcacuugAcccggccuac................   5    1    seq
......................................................................uauugcGcuugucccggccuac................   2    1    seq
......................................................................uauugcacuugucUcggccuac................   1    1    seq
......................................................................uauuAcacuugucccggccuac................   1    1    seq
......................................................................uauugcacuugucccggccCac................   1    1    seq
......................................................................uauugcaUuugucccggccuacu...............   1    1    seq
......................................................................uauugcacuugAcccggccuacu...............   1    1    seq
.......................................................................auugcacuugAcccggccu..................   2    1    seq
```
