# Supplementary material for: Identification and Characterization of MicroRNAs from Longitudinal Muscle and Respiratory Tree in Sea Cucumber (Apostichopus japonicus) Using High-Throughput Sequencing
Source: PLoS One. 2015 Aug 5;10(8):e0134899. doi: 10.1371/journal.pone.0134899 (PMC4526669; doi:10.1371/journal.pone.0134899)
Supplement: S1 File — (ZIP) [file pone.0134899.s002.zip › S1 File/The secondary structures of the novel miRNAs in LTM/Scaffold39_50.pdf]

Provisional ID	:	Scaffold39_50
Score total	:	1.4
Score for star read(s)	:	-1.3
Score for read counts	:	0
Score for mfe	:	1.1
Score for randfold	:	1.6
Score for cons. seed	:	
Total read count	:	319
Mature read count	:	319
Loop read count	:	0
Star read count	:	0

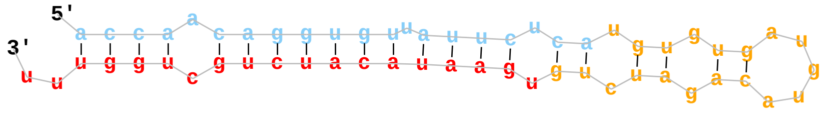

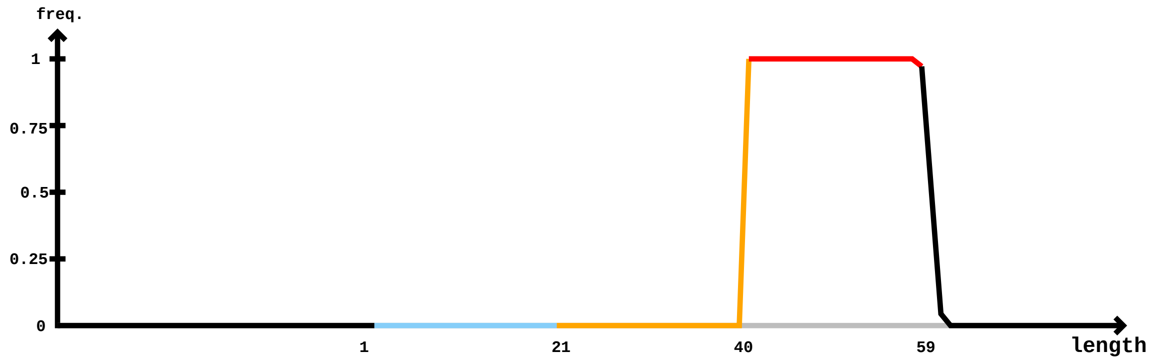

Star Mature

```
5'- guauggaccuguuauucaucagacugauaagaccaacagguguuauucucaugugugauguacagaucugugaauacaucugcugguuuuuauuaggcucgaugauaga   -3'   exp
    ..............((((((.(((((((((((.(((((((.((((.((.((.((.....)).)).)).))))))))))))).))).))))))))).)).)))))....   reads   mm   sample
    ......................................................................ugaauacaucugcugguA.....................   1       1    seq
    ......................................................................ugaauacaucugcugguC.....................   8       1    seq
    ......................................................................ugaauacaucugcugguuu....................   2       0    seq
    ......................................................................ugaauacaucugcugguCu....................   266     1    seq
    ......................................................................ugaauacaucugcugguCuu...................   28      1    seq
    ......................................................................ugaauacaucugcugguuuAu..................   1       1    seq
    ......................................................................ugaauacaucugcugguCuuu..................   13      1    seq
```
