# Supplementary material for: Identification and Characterization of MicroRNAs from Longitudinal Muscle and Respiratory Tree in Sea Cucumber (Apostichopus japonicus) Using High-Throughput Sequencing
Source: PLoS One. 2015 Aug 5;10(8):e0134899. doi: 10.1371/journal.pone.0134899 (PMC4526669; doi:10.1371/journal.pone.0134899)
Supplement: S1 File — (ZIP) [file pone.0134899.s002.zip › S1 File/The secondary structures of the novel miRNAs in LTM/Scaffold554_533.pdf]

Provisional ID	: Scaffold554_533
Score total	: 39.7
Score for star read(s)	: 3.9
Score for read counts	: 32.2
Score for mfe	: 2
Score for randfold	: 1.6
Score for cons. seed	:
Total read count	: 75
Mature read count	: 74
Loop read count	: 0
Star read count	: 1

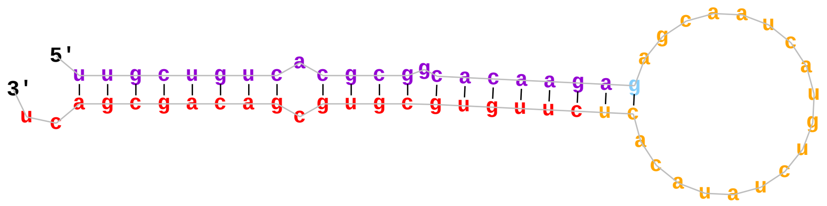

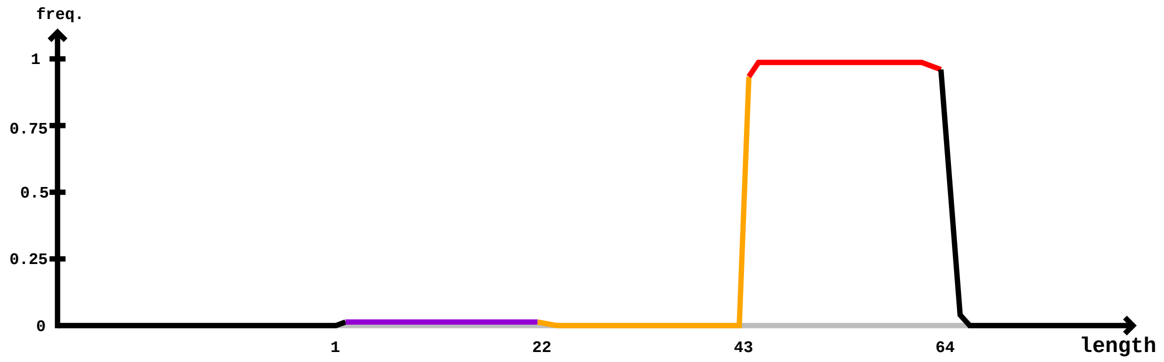


```
                              Star                            Mature
5'- uuaucaacauacccagcucucucguuaguugcugucacgcggcacaagagagcaaucaugucuauacacucuugugcgugcgacagcgacugauacagggcucccuuuca  -3'   obs
    uuaucaacauacccagcucucucguuaguugcugucacgcggcacaagagagcaaucaugucuauacacucuugugcgugcgacagcgacugauacagggcucccuuuca         exp
    ..............((((((...(((((((((((((.((((.((((((((..................)))))))))))).)))))))))))))..))))))........   reads   mm   sample
    ............................uugcugucCcgcggcacaaga.............................................................   1       1    seq
    ......................................................................cuugugcgugcgacagcga.....................   1       0    seq
    ......................................................................cuugugcgugcgacagcgac....................   1       0    seq
    ......................................................................cuugCgcgugcgacagcgacu...................   1       1    seq
    ......................................................................cuuUugcgugcgacagcgacu...................   6       1    seq
    ......................................................................cuugugcgCgcgacagcgacu...................   1       1    seq
    ......................................................................cuugugcgugcgacagcgacG...................   10      1    seq
    ......................................................................cuuguAcgugcgacagcgacu...................   1       1    seq
    ......................................................................cuugugcgugUgacagcgacu...................   1       1    seq
    ......................................................................cuGgugcgugcgacagcgacu...................   4       1    seq
    ......................................................................cuugugcCugcgacagcgacu...................   1       1    seq
    ......................................................................cuugugcgugcgacagcgacC...................   33      1    seq
    ......................................................................cuugugcgugcgacagcgacA...................   8       1    seq
    ......................................................................cuugugcgugcgacGgcgacu...................   1       1    seq
    ......................................................................cuugugUgugcgacagcgacuga.................   1       1    seq
    .......................................................................uugugcgugcgacagcgacC...................   2       1    seq
    .......................................................................uugGgcgugcgacagcgacuga.................   2       1    seq
```
