# Supplementary material for: Identification and Characterization of MicroRNAs from Longitudinal Muscle and Respiratory Tree in Sea Cucumber (Apostichopus japonicus) Using High-Throughput Sequencing
Source: PLoS One. 2015 Aug 5;10(8):e0134899. doi: 10.1371/journal.pone.0134899 (PMC4526669; doi:10.1371/journal.pone.0134899)
Supplement: S1 File — (ZIP) [file pone.0134899.s002.zip › S1 File/The secondary structures of the novel miRNAs in LTM/Scaffold652_587.pdf]

Provisional ID	:	Scaffold652_587
Score total	:	2.5
Score for star read(s)	:	-1.3
Score for read counts	:	0
Score for mfe	:	2.2
Score for randfold	:	1.6
Score for cons. seed	:	
Total read count	:	3210
Mature read count	:	3210
Loop read count	:	0
Star read count	:	0

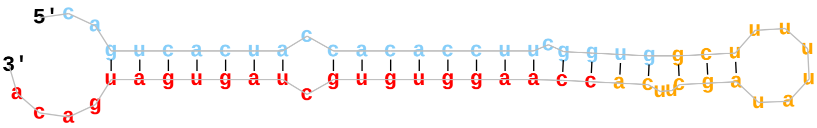

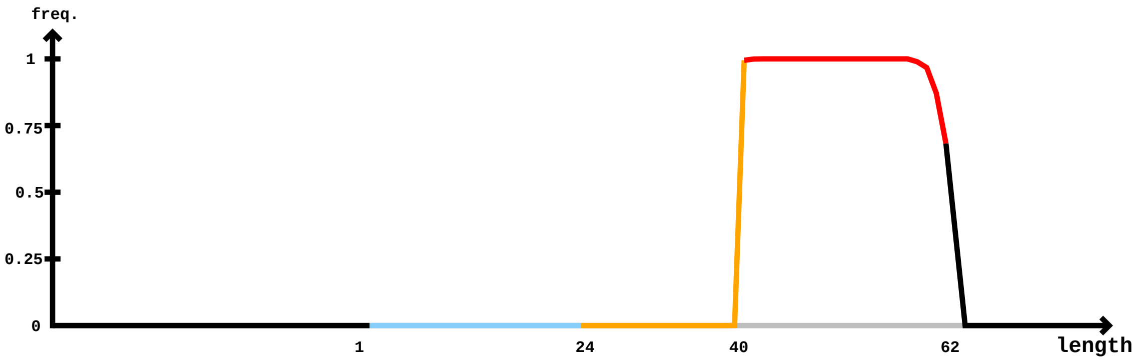

Star Mature

```
5'- gaagacgaucgcaccgugccuagucucccugcagucacuaccacaccuucggug gcuuuuuauagcuucaccaaggugugcuagugaugacagggggaugggaacaau   -3'    exp
    ...............((.((((.((((((((..(((((((.((((((((.(((((((......)))..)))))))))))).)))))))..)))))))))))).))...   reads   mm   sample
    .....................................................................accaaggugAgcuagugaugaca................   1       1    seq
    ......................................................................ccaaggugugcuagCgau....................   1       1    seq
    ......................................................................ccaaggugugcuagugaC....................   8       1    seq
    ......................................................................ccaaggugugcuagugau....................   23      0    seq
    ......................................................................ccGaggugugcuagugau....................   2       1    seq
    ......................................................................ccaaggugugcuagugaCg...................   39      1    seq
    ......................................................................ccaaggugugcuagugauU...................   8       1    seq
    ......................................................................ccaaggugugcuagugauA...................   2       1    seq
    ......................................................................ccaaggugCgcuagugaug...................   1       1    seq
    ......................................................................ccaaggugugcuagugaug...................   21      0    seq
    ......................................................................ccaaggugugcCagugauga..................   1       1    seq
    ......................................................................ccaaggugugcuGgugauga..................   3       1    seq
    ......................................................................ccaaggugugcuagugauUa..................   1       1    seq
    ......................................................................ccaaggGgugcuagugauga..................   1       1    seq
    ......................................................................ccaaggugAgcuagugauga..................   1       1    seq
    ......................................................................Ucaaggugugcuagugauga..................   1       1    seq
    ......................................................................ccaaggugugcuagugaugG..................   6       1    seq
    ......................................................................ccUaggugugcuagugauga..................   3       1    seq
    ......................................................................ccaaggugugcuagugaCga..................   270     1    seq
    ......................................................................ccaaggugugcuagCgauga..................   1       1    seq
    ......................................................................ccaaggugugcuCgugauga..................   1       1    seq
    ......................................................................ccGaggugugcuagugauga..................   2       1    seq
    ......................................................................ccaaggugugcuagugUuga..................   1       1    seq
    ......................................................................ccaaggugCgcuagugauga..................   3       1    seq
    ......................................................................ccaaAgugugcuagugauga..................   1       1    seq
    ......................................................................Gcaaggugugcuagugauga..................   1       1    seq
    ......................................................................ccaaggugugcuagugaugU..................   4       1    seq
    ......................................................................ccaaggugugcuagugaugC..................   2       1    seq
    ......................................................................ccaaggugugcuagugGuga..................   2       1    seq
    ......................................................................ccaaggugugcuagugaGga..................   1       1    seq
    ......................................................................ccUaggugugcuagugaugac.................   2       1    seq
    ......................................................................ccaaggugugcuagugGugac.................   3       1    seq
    ......................................................................ccaaggugugcuGgugaugac.................   4       1    seq
    ......................................................................ccaaUgugugcuagugaugac.................   1       1    seq
```

```
                              Star                                              Mature
gaagacgaucgcaccgugccuagucuccugcagucacuaccacaccuucggugggcuuuuuauagcuucaccaaggugugcuagugaugacagggggaugggaacaau
.....................................................................ccaaggugugcCagugaugac................. 3    1    seq
.....................................................................ccaaggugugcuUgugaugac................. 1    1    seq
.....................................................................ccaagguguAcuagugaugac................. 1    1    seq
.....................................................................ccaaggugugcuagugauCac................. 1    1    seq
.....................................................................cGaaggugugcuagugaugac................. 2    1    seq
.....................................................................ccaaggugugcuagugaAgac................. 1    1    seq
.....................................................................ccaUggugugcuagugaugac................. 3    1    seq
.....................................................................ccaaggugugGuagugaugac................. 1    1    seq
.....................................................................ccaaggugugcAagugaugac................. 1    1    seq
.....................................................................ccCaggugugcuagugaugac................. 1    1    seq
.....................................................................ccaaggugugcuagugaugUc................. 1    1    seq
.....................................................................ccaGggugugcuagugaugac................. 5    1    seq
.....................................................................ccaagguAugcuagugaugac................. 1    1    seq
.....................................................................ccaaggugugcuaguAaugac................. 1    1    seq
.....................................................................ccaaggugugcuagGgaugac................. 2    1    seq
.....................................................................cUaaggugugcuagugaugac................. 2    1    seq
.....................................................................ccaaggugugcGagugaugac................. 1    1    seq
.....................................................................ccaaggugGgcuagugaugac................. 1    1    seq
.....................................................................ccaaggugugcuagugaGgac................. 1    1    seq
.....................................................................ccaaggCgugcuagugaugac................. 5    1    seq
.....................................................................ccaaggugugcuaUugaugac................. 1    1    seq
.....................................................................ccaaggugugcuagugaugGc................. 2    1    seq
.....................................................................ccaaggAgugcuagugaugac................. 2    1    seq
.....................................................................ccaaggugugcuagugaCgac................. 530  1    seq
.....................................................................ccGaggugugcuagugaugac................. 4    1    seq
.....................................................................ccaagguUugcuagugaugac................. 1    1    seq
.....................................................................ccaaggGgugcuagugaugac................. 5    1    seq
.....................................................................ccaaggugCgcuagugaugac................. 5    1    seq
.....................................................................ccaaAgugugcuagugaugac................. 3    1    seq
.....................................................................ccaaggugugcuagCgaugac................. 3    1    seq
.....................................................................ccaaggugugcuagugauAac................. 1    1    seq
.....................................................................ccaaggugugcuaguCaugaca................ 1    1    seq
.....................................................................ccaaggugugcuCgugaugaca................ 1    1    seq
.....................................................................ccaaggugugcuagugaugCca................ 8    1    seq
.....................................................................ccaaggugugcuagugauUaca................ 8    1    seq
.....................................................................ccaagguUugcuagugaugaca................ 1    1    seq
.....................................................................ccaaggugugcuagugGugaca................ 41   1    seq
.....................................................................ccaCggugugcuagugaugaca................ 2    1    seq
.....................................................................ccaaggugugcuGgugaugaca................ 21   1    seq
.....................................................................ccaaUgugugcuagugaugaca................ 2    1    seq
.....................................................................ccaagAugugcuagugaugaca................ 2    1    seq
.....................................................................cAaaggugugcuagugaugaca................ 3    1    seq
.....................................................................ccaaggugAgcuagugaugaca................ 18   1    seq
.....................................................................ccaaggGgugcuagugaugaca................ 49   1    seq
.....................................................................ccaaggugugcuagugauCaca................ 1    1    seq
.....................................................................ccaaggugCgcuagugaugaca................ 27   1    seq
.....................................................................ccaaggugugGuagugaugaca................ 1    1    seq
.....................................................................ccaaggugugcuagugaGgaca................ 6    1    seq
.....................................................................ccUaggugugcuagugaugaca................ 27   1    seq
.....................................................................ccaaggugugcuaUugaugaca................ 1    1    seq
.....................................................................ccCaggugugcuagugaugaca................ 5    1    seq
.....................................................................ccaaggugugcuagugaugUca................ 7    1    seq
.....................................................................ccaaggugugcuaAugaugaca................ 7    1    seq
.....................................................................ccaaggAgugcuagugaugaca................ 23   1    seq
.....................................................................ccaagguAugcuagugaugaca................ 4    1    seq
.....................................................................ccaaggugugcuaCugaugaca................ 1    1    seq
.....................................................................ccaaAgugugcuagugaugaca................ 8    1    seq
.....................................................................ccaaggugugcuagAgaugaca................ 3    1    seq
.....................................................................ccaaggugugcuagCgaugaca................ 24   1    seq
.....................................................................ccaaggugugcuagugUugaca................ 7    1    seq
.....................................................................cGaaggugugcuagugaugaca................ 3    1    seq
.....................................................................ccaUggugugcuagugaugaca................ 19   1    seq
.....................................................................ccaGggugugcuagugaugaca................ 34   1    seq
.....................................................................ccaaCgugugcuagugaugaca................ 1    1    seq
.....................................................................ccaaggugugcuagugCugaca................ 1    1    seq
.....................................................................ccaaggugugcuagGgaugaca................ 20   1    seq
.....................................................................cUaaggugugcuagugaugaca................ 21   1    seq
.....................................................................ccaaggugugcuaguUaugaca................ 2    1    seq
.....................................................................ccGaggugugcuagugaugaca................ 26   1    seq
.....................................................................ccaagguguCcuagugaugaca................ 1    1    seq
```

Star **Mature**

```
gaagacgaucgcaccgugccuagucucccugcagucacuaccacaccuucggugggcuuuuuauagcuucaccaaggugugcuagugaugacagggggaugggaacaau
......................................................................ccaagUugugcuagugaugaca................    1      1      seq
......................................................................ccaaggugugcGagugaugaca................    1      1      seq
......................................................................ccaaggCgugcuagugaugaca................    36     1      seq
......................................................................ccaaggugugcuagugaugGca................    51     1      seq
......................................................................ccaagguguUcuagugaugaca................    1      1      seq
......................................................................ccaaggugugcCagugaugaca................    19     1      seq
......................................................................ccaaggugugcuaguAaugaca................    15     1      seq
......................................................................ccaaggugugcuagugaCgaca................    1566   1      seq
......................................................................ccaagguguAcuagugaugaca................    15     1      seq
......................................................................ccaaggugGgcuagugaugaca................    9      1      seq
......................................................................ccaaggugugcAagugaugaca................    6      1      seq
......................................................................ccaaggugugcuagugauAaca................    16     1      seq
......................................................................ccaaggugugcuUgugaugaca................    6      1      seq
.......................................................................caaggugugcuagugaCga..................    3      1      seq
.......................................................................caaggugugcuagugaCgac.................    2      1      seq
.......................................................................cCaggugugcuagugaugaca................    3      1      seq
.......................................................................caaUgugugcuagugaugaca................    3      1      seq
.......................................................................caaggugugcuagugaCgaca................    4      1      seq
........................................................................aaggugugcuagugaCgac.................    1      1      seq
........................................................................aaggugugcuagugaCgaca................    1      1      seq
```
