# Supplementary material for: Identification and Characterization of MicroRNAs from Longitudinal Muscle and Respiratory Tree in Sea Cucumber (Apostichopus japonicus) Using High-Throughput Sequencing
Source: PLoS One. 2015 Aug 5;10(8):e0134899. doi: 10.1371/journal.pone.0134899 (PMC4526669; doi:10.1371/journal.pone.0134899)
Supplement: S1 File — (ZIP) [file pone.0134899.s002.zip › S1 File/The secondary structures of the novel miRNAs in LTM/Scaffold759_623.pdf]

Provisional ID	:	Scaffold759_623
Score total	:	2551.8
Score for star read(s)	:	3.9
Score for read counts	:	2544.7
Score for mfe	:	1.6
Score for randfold	:	1.6
Score for cons. seed	:	
Total read count	:	5003
Mature read count	:	2127
Loop read count	:	0
Star read count	:	2876

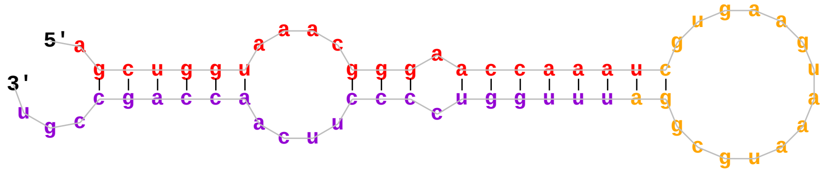

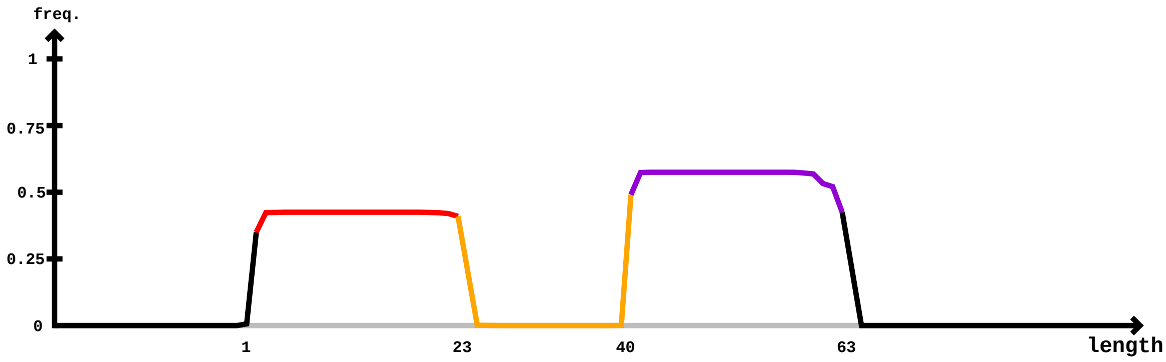

Mature **Star**

```
5'- ccggaaaauguagcgcuuuagcugguaaacgggaaccaaaucgugaaguaaaugcggauuugguccccuucaaccagccguagcacugcauucuagacaacucaccuacag   -3'   obs
    ccggaaaauguagcgcuuuagcugguaaacgggaaccaaaucgugaaguaaaugcggauuugguccccuucaaccagccguagcacugcauucuagacaacucaccuacag         exp
    ......((((((.(((...((((((....(((.((((((((..............)))))))).)))....))))))...))).)))))).....................   reads   mm   sample
    ..................uagcugguaaacgggaacca.........................................................................   3       0    seq
    ..................uagcugguaaacgggaaccaaa.......................................................................   3       0    seq
    ..................uagcugguaaacgggaaUcaaa.......................................................................   1       1    seq
    ..................Cagcugguaaacgggaaccaaau......................................................................   5       1    seq
    ..................uagcugguaaacgggaacGaaau......................................................................   1       1    seq
    ..................uagcugguaaacgggaaccaaau......................................................................   13      0    seq
    ..................uagcugguaaacgggaaccaaaC......................................................................   2       1    seq
    ..................Aagcugguaaacgggaaccaaau......................................................................   3       1    seq
    ..................uagcugguaaacgggaaccaaauU.....................................................................   1       1    seq
    ...................agcugguaaacgggaacc..........................................................................   5       0    seq
    ...................agcugguaaacgggaacca.........................................................................   2       0    seq
    ...................agcGgguaaacgggaaccaa........................................................................   1       1    seq
    ...................agcugguaaacgggaaccaa........................................................................   14      0    seq
    ...................agcGgguaaacgggaaccaaa.......................................................................   1       1    seq
    ...................agcugguaaacgggaaccaaC.......................................................................   1       1    seq
    ...................agcugguaaacgggaGccaaa.......................................................................   1       1    seq
    ...................agcugguaaacgggaaccaaa.......................................................................   38      0    seq
    ...................agcugguaaacgggaaccaGa.......................................................................   1       1    seq
    ...................agcugguaaacgggaaccaaU.......................................................................   4       1    seq
    ...................agcugguaaacgggaaccaaG.......................................................................   1       1    seq
    ...................agcugguaaacgggaaccaaau......................................................................   1303    0    seq
    ...................agUugguaaacgggaaccaaau......................................................................   4       1    seq
    ...................agcuAguaaacgggaaccaaau......................................................................   2       1    seq
    ...................agcCgguaaacgggaaccaaau......................................................................   6       1    seq
    ...................agcugguaaacgggaaccaaUu......................................................................   1       1    seq
    ...................agcugguaGacgggaaccaaau......................................................................   3       1    seq
    ...................agcugguaaacgggaacGaaau......................................................................   1       1    seq
    ...................agcugguaaacgggaaccaaaG......................................................................   10      1    seq
    ...................aCcugguaaacgggaaccaaau......................................................................   2       1    seq
    ...................agcuCguaaacgggaaccaaau......................................................................   1       1    seq
    ...................agcugguaaacgggaGccaaau......................................................................   6       1    seq
    ...................agcugguaaacgggaaccaaaC......................................................................   84      1    seq
    ...................Ggcugguaaacgggaaccaaau......................................................................   5       1    seq
```

```
                   Mature                                  Star
ccggaaaauguagcgcuuuagcugguaaacgggaaccaaaucgugaaguaaaugcggauuugguccccuucaaccagccguagcacugcauucuagacaacucaccuacag
...................agcAgguaaacgggaaccaaau......................................................................   3    1   seq
...................agcuggCaaacgggaaccaaau......................................................................   4    1   seq
...................agcugguaaacgggaaUcaaau......................................................................   4    1   seq
...................Ugcugguaaacgggaaccaaau......................................................................   3    1   seq
...................agcugguaaUcgggaaccaaau......................................................................   1    1   seq
...................agcuggGaaacgggaaccaaau......................................................................   2    1   seq
...................agcugguaaacgggaaccGaau......................................................................   6    1   seq
...................agcGgguaaacgggaaccaaau......................................................................   8    1   seq
...................agcugguaaacgggaaccaaGu......................................................................   3    1   seq
...................agcugguaaacgggaaccaUau......................................................................   1    1   seq
...................agcugguaaacgAgaaccaaau......................................................................   2    1   seq
...................agcugguaaacgggaacUaaau......................................................................   3    1   seq
...................aAcugguaaacgggaaccaaau......................................................................   5    1   seq
...................agcugguaaGcgggaaccaaau......................................................................   10   1   seq
...................agcugguaaacgggaaccaaaA......................................................................   4    1   seq
...................agcugguaaacAggaaccaaau......................................................................   3    1   seq
...................agcugguaaacgggaUccaaau......................................................................   1    1   seq
...................agcugguUaacgggaaccaaau......................................................................   1    1   seq
...................agcugguaaaUgggaaccaaau......................................................................   1    1   seq
...................aUcugguaaacgggaaccaaau......................................................................   5    1   seq
...................agcugguaaacgggaaccUaau......................................................................   1    1   seq
...................agcugguaUacgggaaccaaau......................................................................   3    1   seq
...................agcugguaaacgUgaaccaaau......................................................................   1    1   seq
...................agcugguGaacgggaaccaaau......................................................................   5    1   seq
...................agcugAuaaacgggaaccaaau......................................................................   1    1   seq
...................agcugguaaacgggGaccaaau......................................................................   6    1   seq
...................agcugguaaacgggaaccaGau......................................................................   6    1   seq
...................agcuggGaaacgggaaccaaauc.....................................................................   1    1   seq
...................agcugguaaacgggaaccaaauA.....................................................................   3    1   seq
...................agcugguaaGcgggaaccaaauc.....................................................................   1    1   seq
...................agcuAguaaacgggaaccaaauc.....................................................................   1    1   seq
...................agcugguaaacgggaaccaaGuc.....................................................................   1    1   seq
...................agcugguaCacgggaaccaaauc.....................................................................   1    1   seq
...................agcugguaaacgggaaccaaaCc.....................................................................   1    1   seq
...................agcugguaaacgggaacUaaauc.....................................................................   1    1   seq
...................agcugguaaacgggaaccaaauU.....................................................................   31   1   seq
...................agcugguaaacgggaGccaaauc.....................................................................   1    1   seq
...................agcugguaaacgggaaccaGauc.....................................................................   1    1   seq
...................agcugguaaacgggaaccaaauc.....................................................................   84   0   seq
...................agcugguaaacgggaaccaaaucU....................................................................   1    1   seq
....................gcugguaaacgggaaccaa........................................................................   1    0   seq
....................gcugguaaacgggaaccaaa.......................................................................   1    0   seq
....................gcugguaaacgggaaccaGau......................................................................   1    1   seq
....................gcugguaaacgggaUccaaau......................................................................   1    1   seq
....................gcugguaGacgggaaccaaau......................................................................   1    1   seq
....................gcugguaaacgggGaccaaau......................................................................   1    1   seq
....................gcugguaaGcgggaaccaaau......................................................................   1    1   seq
....................gcugguaaacgggaaccaaau......................................................................   125  0   seq
....................gcugguGaacgggaaccaaau......................................................................   1    1   seq
....................gcugguaaacgggaaccaaGu......................................................................   1    1   seq
....................gcugguaaacgggaaccaaaG......................................................................   2    1   seq
....................gcugguaaacgggaaccaaaC......................................................................   10   1   seq
....................gcuUguaaacgggaaccaaau......................................................................   4    1   seq
....................gcugguaaacgggaaccGaauc.....................................................................   1    1   seq
....................Ccugguaaacgggaaccaaauc.....................................................................   1    1   seq
....................gcGgguaaacgggaaccaaauc.....................................................................   1    1   seq
....................gcugguaaacgggaaccaGauc.....................................................................   1    1   seq
....................gcugguaaacgggaacAaaauc.....................................................................   1    1   seq
....................gcugguaaacgggaaccaaauU.....................................................................   15   1   seq
....................gcugguaaacgggaCccaaauc.....................................................................   1    1   seq
....................gcugguaaacgggaaccaaauc.....................................................................   181  0   seq
....................gcAgguaaacgggaaccaaauc.....................................................................   2    1   seq
....................gcugguaaGcgggaaccaaauc.....................................................................   1    1   seq
....................gcugguaaacgggaaccaaaCc.....................................................................   1    1   seq
....................gcuAguaaacgggaaccaaauc.....................................................................   1    1   seq
....................gcugguaaacgggaaccaaauA.....................................................................   3    1   seq
....................Ucugguaaacgggaaccaaauc.....................................................................   1    1   seq
....................gcugguaaacgggaaccaaGuc.....................................................................   3    1   seq
....................gcugguaaacgggGaccaaauc.....................................................................   1    1   seq
....................gcugguaaacgggaaccaaaucU....................................................................   4    1   seq
```

Mature / Star

```
                   Mature                               Star
ccggaaaauguagcgcuuuagcugguaaacgggaaccaaaucgugaaguaaaugcggauuugguccccuucaaccagccguagcacugcauucuagacaacucaccuacag
....................gcugguaaacgggaaccaaaucC....................................................................  1    1    seq
.....................cugguaaacgggaaccaaauc.....................................................................  1    0    seq
.....................cugguaaacgggaaccaaauU.....................................................................  1    1    seq
......................ugguaaacgggaaccaaau......................................................................  2    0    seq
......................ugguaaacgggaaccaGaucgu...................................................................  1    1    seq
......................ugguaaacgggaaccaaaucgu...................................................................  1    0    seq
......................ugguaaacgggaaccaaaucguU..................................................................  1    1    seq
.........................................................auuugguccccuucaacc....................................  1    0    seq
.........................................................auuugguccccuucaaccag..................................  1    0    seq
.........................................................auuugguccccuucaaccagU.................................  1    1    seq
..........................................................uuuCguccccuucaacca...................................  1    1    seq
..........................................................uuugguccccuucaaccG...................................  1    1    seq
..........................................................uuugguccccuucaacca...................................  11   0    seq
..........................................................uuugguccccuucaaccag..................................  13   0    seq
..........................................................uCugguccccuucaaccag..................................  1    1    seq
..........................................................uuugguccccuucaaccaU..................................  1    1    seq
..........................................................uuugguccccuucaaccaA..................................  2    1    seq
..........................................................uuugguccccuucGaccagc.................................  1    1    seq
..........................................................uuugguccccuucaaUcagc.................................  1    1    seq
..........................................................uuuggucUccuucaaccagc.................................  1    1    seq
..........................................................uuugguccccuCcaaccagc.................................  1    1    seq
..........................................................uuCgguccccuucaaccagc.................................  1    1    seq
..........................................................uuugUuccccuucaaccagc.................................  1    1    seq
..........................................................uuuggAccccuucaaccagc.................................  2    1    seq
..........................................................uuuUguccccuucaaccagc.................................  5    1    seq
..........................................................uuugguccccuucaaccagU.................................  115  1    seq
..........................................................uuugguccccuucaaccaAc.................................  1    1    seq
..........................................................uuuAguccccuucaaccagc.................................  1    1    seq
..........................................................Guugguccccuucaaccagc.................................  3    1    seq
..........................................................uuugguccccuucaaccagA.................................  13   1    seq
..........................................................uuuggCccccuucaaccagc.................................  1    1    seq
..........................................................uCugguccccuucaaccagc.................................  1    1    seq
..........................................................uuuggCccccuucaaccagcc................................  4    1    seq
..........................................................uuuggGccccuucaaccagcc................................  2    1    seq
..........................................................uuugUuccccuucaaccagcc................................  1    1    seq
..........................................................uuugguccUcuucaaccagcc................................  2    1    seq
..........................................................uuGgguccccuucaaccagcc................................  1    1    seq
..........................................................uuugguccccuucaaccUgcc................................  2    1    seq
..........................................................uuugguccccuucCaccagcc................................  1    1    seq
..........................................................uuugguccccuucaaccaAcc................................  1    1    seq
..........................................................uuugguccccuAcaaccagcc................................  1    1    seq
..........................................................uuuUguccccuucaaccagcc................................  10   1    seq
..........................................................uuugguccccuucaaUcagcc................................  4    1    seq
..........................................................uuugguccccuucaacUagcc................................  1    1    seq
..........................................................uuugguccccCucaaccagcc................................  2    1    seq
..........................................................uuugguccccuucaaccagAc................................  1    1    seq
..........................................................uuugguccccuucaGccagcc................................  1    1    seq
..........................................................uuuAguccccuucaaccagcc................................  3    1    seq
..........................................................uuugguccccuCcaaccagcc................................  3    1    seq
..........................................................uCugguccccuucaaccagcc................................  3    1    seq
..........................................................uuugguccccuucaaccaUcc................................  1    1    seq
..........................................................uuCgguccccuucaaccagcc................................  2    1    seq
..........................................................uuuggucUccuucaaccagcc................................  1    1    seq
..........................................................uuugguccccuucGaccagcc................................  2    1    seq
..........................................................uuugguccccGucaaccagcc................................  1    1    seq
..........................................................uuuCguccccuucaaccagcc................................  1    1    seq
..........................................................uuAgguccccuucaaccagcc................................  2    1    seq
..........................................................uuuUguccccuucaaccagccg...............................  85   1    seq
..........................................................uuugguccccAucaaccagccg...............................  1    1    seq
..........................................................uuugAuccccuucaaccagccg...............................  10   1    seq
..........................................................uuugguccccuucaaccagAcg...............................  5    1    seq
..........................................................uuugguccccuucCaccagccg...............................  1    1    seq
..........................................................uuugguccccuucaaccagUcg...............................  10   1    seq
..........................................................uuugguccccuucaUccagccg...............................  3    1    seq
..........................................................uuugguAcccuucaaccagccg...............................  4    1    seq
..........................................................uuugguccccuucaaUcagccg...............................  8    1    seq
..........................................................uuugguccccuucaaccaUccg...............................  4    1    seq
..........................................................uuuAguccccuucaaccagccg...............................  9    1    seq
..........................................................uuugguccccuucaaccUgccg...............................  3    1    seq
..........................................................uuugguccccuGcaaccagccg...............................  2    1    seq
```

```
                   Mature                                   Star
ccggaaaauguagcgcuuuagcugguaaacgggaaccaaaucgugaaguaaaugcggauuugguccccuucaaccagccguagcacugcauucuagacaacucaccuacag
..........................................................uuuggCccccuucaaccagccg...............................    23    1    seq
..........................................................uuuggucccAuucaaccagccg...............................    3     1    seq
..........................................................uuugguccccuucaaGcagccg...............................    1     1    seq
..........................................................uuugguccccuucaaccagGcg...............................    5     1    seq
..........................................................uuugguccccuucaCccagccg...............................    2     1    seq
..........................................................uuugguccccuucaacGagccg...............................    1     1    seq
..........................................................uuugguccccCucaaccagccg...............................    19    1    seq
..........................................................uuuggGccccuucaaccagccg...............................    6     1    seq
..........................................................uuAgguccccuucaaccagccg...............................    16    1    seq
..........................................................uuugguccccuucaaccCgccg...............................    2     1    seq
..........................................................uuugguccccuucaacUagccg...............................    12    1    seq
..........................................................uuuggucAccuucaaccagccg...............................    3     1    seq
..........................................................uuGgguccccuucaaccagccg...............................    26    1    seq
..........................................................uuugguccccuAcaaccagccg...............................    2     1    seq
..........................................................uuugguccccuuUaaccagccg...............................    4     1    seq
..........................................................uuugguccccuuAaaccagccg...............................    2     1    seq
..........................................................uuuggucUccuucaaccagccg...............................    12    1    seq
..........................................................uuuCguccccuucaaccagccg...............................    7     1    seq
..........................................................uuugguccUcuucaaccagccg...............................    13    1    seq
..........................................................uuCgguccccuucaaccagccg...............................    37    1    seq
..........................................................uuuggucccGuucaaccagccg...............................    2     1    seq
..........................................................uuugguUcccuucaaccagccg...............................    11    1    seq
..........................................................uuugguccccuucaGccagccg...............................    23    1    seq
..........................................................uuugCuccccuucaaccagccg...............................    2     1    seq
..........................................................uuugguccccuucaaccaAccg...............................    14    1    seq
..........................................................uuugguccGcuucaaccagccg...............................    2     1    seq
..........................................................uuugguccccuucGaccagccg...............................    26    1    seq
..........................................................uuuggAccccuucaaccagccg...............................    21    1    seq
..........................................................uuuggucGccuucaaccagccg...............................    2     1    seq
..........................................................uuugguccccuucUaccagccg...............................    1     1    seq
..........................................................uuugguccccuCcaaccagccg...............................    29    1    seq
..........................................................uuugguGcccuucaaccagccg...............................    2     1    seq
..........................................................uuugguccccuucaaUcagccgu..............................    67    1    seq
..........................................................uuugguAcccuucaaccagccgu..............................    21    1    seq
..........................................................uuugguccccuGcaaccagccgu..............................    17    1    seq
..........................................................uuugguccccuuUaaccagccgu..............................    51    1    seq
..........................................................uuugguccccuCcaaccagccgu..............................    274   1    seq
..........................................................uuugguccccuuAaaccagccgu..............................    8     1    seq
..........................................................uuugguccccuucCaccagccgu..............................    16    1    seq
..........................................................uuugguccccCucaaccagccgu..............................    243   1    seq
..........................................................uuugguccccuucaUccagccgu..............................    8     1    seq
..........................................................uuugguccccuucaaGcagccgu..............................    15    1    seq
..........................................................uuuggucccGuucaaccagccgu..............................    7     1    seq
..........................................................uuugguccccAucaaccagccgu..............................    24    1    seq
..........................................................uuugguccccuucaCccagccgu..............................    3     1    seq
..........................................................uuugguccccGucaaccagccgu..............................    8     1    seq
..........................................................uuugguccAcuucaaccagccgu..............................    17    1    seq
..........................................................uuugguccccuucGaccagccgu..............................    229   1    seq
..........................................................uuugguccUcuucaaccagccgu..............................    108   1    seq
..........................................................uuugguccccuucaacGagccgu..............................    5     1    seq
..........................................................uuuggucUccuucaaccagccgu..............................    106   1    seq
..........................................................uuugguccccuucaacAagccgu..............................    28    1    seq
..........................................................uuugguccccuucaaAcagccgu..............................    12    1    seq
..........................................................uuugguccccuAcaaccagccgu..............................    23    1    seq
..........................................................uuuggucAccuucaaccagccgu..............................    12    1    seq
..........................................................uuugguccccuucaacUagccgu..............................    88    1    seq
..........................................................uuuggucGccuucaaccagccgu..............................    5     1    seq
..........................................................uuugguccccuucaGccagccgu..............................    233   1    seq
..........................................................uuuggucccAuucaaccagccgu..............................    7     1    seq
..........................................................uuugguccccuucUaccagccgu..............................    15    1    seq
..........................................................uuugguccccuuGaaccagccgu..............................    7     1    seq
..........................................................uuugguGcccuucaaccagccgu..............................    15    1    seq
..........................................................uuugguccGcuucaaccagccgu..............................    13    1    seq
..........................................................uuugguUcccuucaaccagccgu..............................    59    1    seq
...........................................................uugguccccuucaaccagU.................................    9     1    seq
...........................................................uugguccccuCcaaccagc.................................    2     1    seq
...........................................................uugguccccuucaaccagc.................................    19    0    seq
...........................................................uugguccccuucaaccagA.................................    1     1    seq
...........................................................uugguccccuucaCccagc.................................    1     1    seq
...........................................................uuggCccccuucaaccagcc................................    1     1    seq
```

Mature **Star**

```
ccggaaaauguagcgcuuuagcugguaaacgggaaccaaaucgugaaguaaaugcggauuugguccccuucaaccagccguagcacugcauucuagacaacucaccuacag
...........................................................uugguccccuucaaccagGc................................ 1 1 seq
...........................................................uugguccccuucaaccagcA................................ 1 1 seq
...........................................................uuggucccGuucaaccagccg............................... 1 1 seq
...........................................................uugguccccuucUaccagccg............................... 1 1 seq
...........................................................uCgguccccuucaaccagccg............................... 1 1 seq
...........................................................uuggAccccuucaaccagccg............................... 1 1 seq
...........................................................uugguccccuucaacUagccg............................... 1 1 seq
...........................................................uugUuccccuucaaccagccg............................... 1 1 seq
...........................................................uugAuccccuucaaccagccg............................... 1 1 seq
...........................................................uuUguccccuucaaccagccg............................... 1 1 seq
...........................................................uugguccccuGcaaccagccgu.............................. 2 1 seq
...........................................................uugguccccuucaaccagcAgu.............................. 3 1 seq
...........................................................uuCguccccuucaaccagccgu.............................. 1 1 seq
...........................................................uuggGccccuucaaccagccgu.............................. 7 1 seq
...........................................................uuggCccccuucaaccagccgu.............................. 21 1 seq
...........................................................uugguccccuucaaccCgccgu.............................. 1 1 seq
...........................................................uugguUcccuucaaccagccgu.............................. 8 1 seq
...........................................................uugguccccuucaaccaUccgu.............................. 5 1 seq
...........................................................uugguccccuucaacAagccgu.............................. 4 1 seq
...........................................................uugguccccuucaUccagccgu.............................. 1 1 seq
...........................................................uuggAccccuucaaccagccgu.............................. 18 1 seq
...........................................................uugCuccccuucaaccagccgu.............................. 6 1 seq
...........................................................uugguccccuucaaccGgccgu.............................. 15 1 seq
...........................................................uugguccccuucaacUagccgu.............................. 8 1 seq
...........................................................uugguccccuucaaccaAccgu.............................. 4 1 seq
...........................................................uugguccccuucaaccUgccgu.............................. 6 1 seq
...........................................................uugguAcccuucaaccagccgu.............................. 3 1 seq
...........................................................uugguccccuucaacGagccgu.............................. 1 1 seq
...........................................................uugguccccuucaaccagcGgu.............................. 2 1 seq
...........................................................uuggucUccuucaaccagccgu.............................. 11 1 seq
...........................................................uugguccccuucaaAcagccgu.............................. 2 1 seq
...........................................................uugguccccuucGaccagccgu.............................. 24 1 seq
...........................................................uugguccccuucaaccaCccgu.............................. 1 1 seq
...........................................................uuggucccUuucaaccagccgu.............................. 13 1 seq
...........................................................uugguccccuuAaaccagccgu.............................. 1 1 seq
...........................................................uugguccccuucUaccagccgu.............................. 6 1 seq
...........................................................uugguccccuucaaGcagccgu.............................. 2 1 seq
...........................................................uuAguccccuucaaccagccgu.............................. 12 1 seq
...........................................................uugguccccuuUaaccagccgu.............................. 1 1 seq
...........................................................uugguccUcuucaaccagccgu.............................. 9 1 seq
...........................................................uugguccccuucaaccagUcgu.............................. 12 1 seq
...........................................................uugguccccuucaaUcagccgu.............................. 6 1 seq
...........................................................uugguccccGucaaccagccgu.............................. 1 1 seq
...........................................................uugguccccuAcaaccagccgu.............................. 3 1 seq
...........................................................uugAuccccuucaaccagccgu.............................. 6 1 seq
...........................................................uugUuccccuucaaccagccgu.............................. 55 1 seq
...........................................................uuggucccGuucaaccagccgu.............................. 1 1 seq
...........................................................uugguccccuucaaccagAcgu.............................. 3 1 seq
...........................................................uugguccccuucaCccagccgu.............................. 1 1 seq
...........................................................uuUguccccuucaaccagccgu.............................. 16 1 seq
...........................................................uugguccccuucaaccagGcgu.............................. 3 1 seq
...........................................................uugguccccAucaaccagccgu.............................. 4 1 seq
...........................................................uugguccccuCcaaccagccgu.............................. 22 1 seq
...........................................................uugguccccuucaGccagccgu.............................. 15 1 seq
...........................................................uugguccAcuucaaccagccgu.............................. 1 1 seq
...........................................................uugguccccCucaaccagccgu.............................. 24 1 seq
...........................................................uugguGcccuucaaccagccgu.............................. 1 1 seq
...........................................................uugUuccccuucaaccagccgua............................. 1 1 seq
............................................................ugguccccuucaaccagccAu.............................. 1 1 seq
............................................................ugCuccccuucaaccagccgu.............................. 1 1 seq
............................................................uggCccccuucaaccagccgu.............................. 1 1 seq
............................................................uggGccccuucaaccagccgu.............................. 2 1 seq
............................................................ugguccccuuUaaccagccgu.............................. 1 1 seq
```
