# Supplementary material for: Identification and Characterization of MicroRNAs from Longitudinal Muscle and Respiratory Tree in Sea Cucumber (Apostichopus japonicus) Using High-Throughput Sequencing
Source: PLoS One. 2015 Aug 5;10(8):e0134899. doi: 10.1371/journal.pone.0134899 (PMC4526669; doi:10.1371/journal.pone.0134899)
Supplement: S1 File — (ZIP) [file pone.0134899.s002.zip › S1 File/The secondary structures of the novel miRNAs in LTM/Scaffold759_626.pdf]

Provisional ID	:	Scaffold759_626
Score total	:	1.1
Score for star read(s)	:	-1.3
Score for read counts	:	0
Score for mfe	:	0.8
Score for randfold	:	1.6
Score for cons. seed	:	
Total read count	:	9549
Mature read count	:	9549
Loop read count	:	0
Star read count	:	0

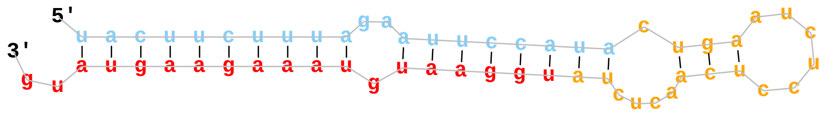

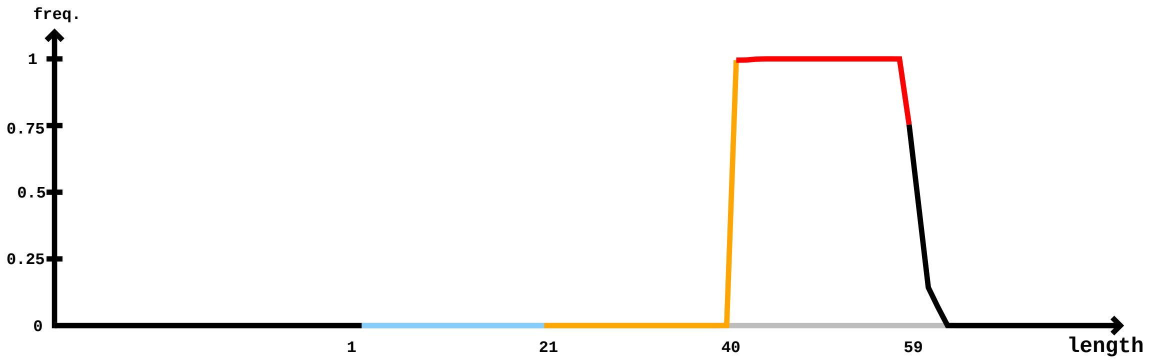

Star **Mature**

```
5'- accaucuucaccauaucccaugccauaacauacuucuuuagaauuccauacugaaucuccucaacucuauggaauguaaagaaguauguauugaaacugggaccucaca   -3'   exp
    ...............(((((...((..(((((((((((((..((((((((.(((......)))....)))))))).)))))))))))))..))....)))))....... reads  mm  sample
    ....................................................................Cuggaauguaaagaagua....................... 2      1   seq
    ....................................................................Cuggaauguaaagaaguau...................... 1      1   seq
    .....................................................................uggaauguaaagCaguau...................... 1      1   seq
    .....................................................................uggaauguaaagaaguUu...................... 3      1   seq
    .....................................................................uggaauguaaGgaaguau...................... 27     1   seq
    .....................................................................uggaauguaUagaaguau...................... 2      1   seq
    .....................................................................Aggaauguaaagaaguau...................... 1      1   seq
    .....................................................................ugAaauguaaagaaguau...................... 3      1   seq
    .....................................................................uggaauguaaagaaguaG...................... 22     1   seq
    .....................................................................uggaauguaaagaagCau...................... 7      1   seq
    .....................................................................Gggaauguaaagaaguau...................... 4      1   seq
    .....................................................................uggaauguGaagaaguau...................... 6      1   seq
    .....................................................................uggaauguaaagaaguau...................... 1981   0   seq
    .....................................................................uggaauguaaagaagGau...................... 1      1   seq
    .....................................................................ugUaauguaaagaaguau...................... 5      1   seq
    .....................................................................uUgaauguaaagaaguau...................... 13     1   seq
    .....................................................................uggaaugGaaagaaguau...................... 1      1   seq
    .....................................................................uggaauguaGagaaguau...................... 3      1   seq
    .....................................................................uggaauguaaUgaaguau...................... 2      1   seq
    .....................................................................uggaauAuaaagaaguau...................... 1      1   seq
    .....................................................................uggaauguUaagaaguau...................... 2      1   seq
    .....................................................................uggaauguaaagaaguaC...................... 162    1   seq
    .....................................................................uggaauguaaagaagAau...................... 3      1   seq
    .....................................................................uggaauguaaagaaAuau...................... 3      1   seq
    .....................................................................uggaauguaaagaaguaA...................... 25     1   seq
    .....................................................................uggaauguaaagaaUuau...................... 1      1   seq
    .....................................................................uggGauguaaagaaguau...................... 12     1   seq
    .....................................................................uggUauguaaagaaguau...................... 3      1   seq
    .....................................................................uggaGuguaaagaaguau...................... 11     1   seq
    .....................................................................uggaauguaaagaaguGu...................... 15     1   seq
    .....................................................................uggCauguaaagaaguau...................... 2      1   seq
    .....................................................................uggaauguaaagGaguau...................... 9      1   seq
    .....................................................................Cggaauguaaagaaguau...................... 10     1   seq
    .....................................................................uggaaCguaaagaaguau...................... 6      1   seq
```

Star Mature

```
accaucuucaccauaucccaugccauaacauacuucuuuagaauuccauacugaaucuccucaacucuauggaauguaaagaaguauguauugaaacugggaccucaca
.....................................................................uggaaugCaaagaaguau......................    6      1    seq
.....................................................................uggaauguaUagaaguaug.....................    1      1    seq
.....................................................................Gggaauguaaagaaguaug.....................    6      1    seq
.....................................................................uggaauguaaagaagAaug.....................    4      1    seq
.....................................................................uggaauguaaGgaaguaug.....................    24     1    seq
.....................................................................uggaGuguaaagaaguaug.....................    13     1    seq
.....................................................................uggaauguaaagaaguaAg.....................    3      1    seq
.....................................................................uggaaugCaaagaaguaug.....................    6      1    seq
.....................................................................uAgaauguaaagaaguaug.....................    5      1    seq
.....................................................................uggaUuguaaagaaguaug.....................    1      1    seq
.....................................................................uggaauAuaaagaaguaug.....................    1      1    seq
.....................................................................uggUauguaaagaaguaug.....................    5      1    seq
.....................................................................uggaauguaGagaaguaug.....................    14     1    seq
.....................................................................ugUaauguaaagaaguaug.....................    10     1    seq
.....................................................................uUgaauguaaagaaguaug.....................    9      1    seq
.....................................................................uggaauguaaagaaguauA.....................    231    1    seq
.....................................................................uggaauguaaagaaguGug.....................    9      1    seq
.....................................................................uggaaugAaaagaaguaug.....................    2      1    seq
.....................................................................uggGauguaaagaaguaug.....................    24     1    seq
.....................................................................ugAaauguaaagaaguaug.....................    5      1    seq
.....................................................................uggaauguaaagGaguaug.....................    10     1    seq
.....................................................................uggaauguaaagaaguauU.....................    75     1    seq
.....................................................................uggaauguaaagaaguaug.....................    2486   0    seq
.....................................................................uggaauguaaagaaAuaug.....................    3      1    seq
.....................................................................uggaauguaaagaCguaug.....................    1      1    seq
.....................................................................uggaauguaaagaaguaCg.....................    14     1    seq
.....................................................................Aggaauguaaagaaguaug.....................    6      1    seq
.....................................................................uggaauguaaagaagCaug.....................    6      1    seq
.....................................................................uggaauguaaagaaguauC.....................    9      1    seq
.....................................................................uggaauguCaagaaguaug.....................    1      1    seq
.....................................................................uggaauguGaagaaguaug.....................    5      1    seq
.....................................................................uggaaAguaaagaaguaug.....................    1      1    seq
.....................................................................uggaauguaaagaaguaGg.....................    2      1    seq
.....................................................................Cggaauguaaagaaguaug.....................    10     1    seq
.....................................................................uggaauguaaaAaaguaug.....................    2      1    seq
.....................................................................uggaauguaaagaGguaug.....................    15     1    seq
.....................................................................uggaaCguaaagaaguaug.....................    2      1    seq
.....................................................................uggaauguaaagCaguaugu....................    7      1    seq
.....................................................................uggaCuguaaagaaguaugu....................    6      1    seq
.....................................................................Aggaauguaaagaaguaugu....................    23     1    seq
.....................................................................uggaauguaGagaaguaugu....................    149    1    seq
.....................................................................uggaaugGaaagaaguaugu....................    5      1    seq
.....................................................................uggaauguaaagUaguaugu....................    12     1    seq
.....................................................................uggUauguaaagaaguaugu....................    85     1    seq
.....................................................................uggaauguaUagaaguaugu....................    19     1    seq
.....................................................................uggaauguaaagGaguaugu....................    125    1    seq
.....................................................................uggaauguaaagaaguaugA....................    476    1    seq
.....................................................................uggaauguaaagaaguauAu....................    36     1    seq
.....................................................................uggaauguaaagaaUuaugu....................    9      1    seq
.....................................................................uggaauguaaagaCguaugu....................    4      1    seq
.....................................................................uggaauguaaCgaaguaugu....................    8      1    seq
.....................................................................uggaauguaaagaaAuaugu....................    29     1    seq
.....................................................................uggGauguaaagaaguaugu....................    221    1    seq
.....................................................................uggaaugAaaagaaguaugu....................    5      1    seq
.....................................................................uggaaugCaaagaaguaugu....................    58     1    seq
.....................................................................uggaauguaaagaaguaGgu....................    9      1    seq
.....................................................................uggaauguaaagaUguaugu....................    11     1    seq
.....................................................................uggaauAuaaagaaguaugu....................    33     1    seq
.....................................................................Gggaauguaaagaaguaugu....................    94     1    seq
.....................................................................uggaauguaaaAaaguaugu....................    10     1    seq
.....................................................................uggCauguaaagaaguaugu....................    5      1    seq
.....................................................................uggaauguaaagaagAaugu....................    43     1    seq
.....................................................................ugCaauguaaagaaguaugu....................    4      1    seq
.....................................................................uggaauCuaaagaaguaugu....................    1      1    seq
.....................................................................uggaauguaaaUaaguaugu....................    6      1    seq
.....................................................................uggaauguaaagaaguaAgu....................    3      1    seq
.....................................................................uggaaAguaaagaaguaugu....................    7      1    seq
.....................................................................uggaauguaaagaaCuaugu....................    4      1    seq
.....................................................................uggaauguaaagaGguaugu....................    157    1    seq
.....................................................................uggaauguaaagaaguUugu....................    5      1    seq
```

```
                              Star                                       Mature
accaucuucaccauaucccaugccauaacauacuucuuuagaauuccauacugaaucuccucaacucuauggaauguaaagaaguaugauauugaaacugggaccucaca
.....................................................................ugAaauguaaagaaguaugu....................   24    1    seq
.....................................................................uggaauguaaGgaaguaugu....................   309   1    seq
.....................................................................uggaaGguaaagaaguaugu....................   9     1    seq
.....................................................................uUgaauguaaagaaguaugu....................   100   1    seq
.....................................................................uggaauguaaagaaguCugu....................   2     1    seq
.....................................................................uAgaauguaaagaaguaugu....................   39    1    seq
.....................................................................uggaauguGaagaaguaugu....................   67    1    seq
.....................................................................uggaauguaCagaaguaugu....................   2     1    seq
.....................................................................uggaauguaaagaaguauUu....................   12    1    seq
.....................................................................uggaauguaaagaagCaugu....................   69    1    seq
.....................................................................uggaauguaaaCaaguaugu....................   2     1    seq
.....................................................................uggaauguaaagaagGaugu....................   21    1    seq
.....................................................................uggaauguCaagaaguaugu....................   5     1    seq
.....................................................................uCgaauguaaagaaguaugu....................   9     1    seq
.....................................................................uggaUuguaaagaaguaugu....................   6     1    seq
.....................................................................uggaauUuaaagaaguaugu....................   9     1    seq
.....................................................................uggaaCguaaagaaguaugu....................   86    1    seq
.....................................................................uggaGuguaaagaaguaugu....................   151   1    seq
.....................................................................uggaauguaaagaaguauCu....................   4     1    seq
.....................................................................ugUaauguaaagaaguaugu....................   79    1    seq
.....................................................................Cggaauguaaagaaguaugu....................   130   1    seq
.....................................................................uggCauguaaagaaguaugua...................   2     1    seq
.....................................................................uggaauguaUagaaguaugua...................   12    1    seq
.....................................................................uggaaugAaaagaaguaugua...................   3     1    seq
.....................................................................uggaauguaaagaUguaugua...................   4     1    seq
.....................................................................uggaauguaaagaCguaugua...................   2     1    seq
.....................................................................uggaaGguaaagaaguaugua...................   2     1    seq
.....................................................................uggaauguaaagaGguaugua...................   49    1    seq
.....................................................................uggaauguaaagaagCaugua...................   27    1    seq
.....................................................................uggaauguGaagaaguaugua...................   38    1    seq
.....................................................................uggaUuguaaagaaguaugua...................   2     1    seq
.....................................................................uggaauguaaagaaguaGgua...................   3     1    seq
.....................................................................uggUauguaaagaaguaugua...................   27    1    seq
.....................................................................uggaauguaaagaaguauUua...................   4     1    seq
.....................................................................uggaaCguaaagaaguaugua...................   25    1    seq
.....................................................................uggaaugCaaagaaguaugua...................   16    1    seq
.....................................................................uggaauguaaagaaUuaugua...................   2     1    seq
.....................................................................uggaauCuaaagaaguaugua...................   1     1    seq
.....................................................................ugUaauguaaagaaguaugua...................   23    1    seq
.....................................................................uggaauguaaagaagAaugua...................   18    1    seq
.....................................................................uggaauAuaaagaaguaugua...................   7     1    seq
.....................................................................uggaauguaaagGaguaugua...................   56    1    seq
.....................................................................uggaauguaaagUaguaugua...................   3     1    seq
.....................................................................ugAaauguaaagaaguaugua...................   11    1    seq
.....................................................................uggaauguaaagaaAuaugua...................   3     1    seq
.....................................................................uggaGuguaaagaaguaugua...................   44    1    seq
.....................................................................uggaauguaaaAaaguaugua...................   6     1    seq
.....................................................................uggGauguaaagaaguaugua...................   75    1    seq
.....................................................................uggaauguaaagaaCuaugua...................   2     1    seq
.....................................................................uggaauguaCagaaguaugua...................   2     1    seq
.....................................................................uggaauguaaagaaguUugua...................   4     1    seq
.....................................................................uggaauguaaagaaguaAgua...................   1     1    seq
.....................................................................uggaauguaaagCaguaugua...................   1     1    seq
.....................................................................uggaauguaaagaaguaugAa...................   4     1    seq
.....................................................................uggaauguaGagaaguaugua...................   40    1    seq
.....................................................................uUgaauguaaagaaguaugua...................   44    1    seq
.....................................................................ugCaauguaaagaaguaugua...................   1     1    seq
.....................................................................uggaauUuaaagaaguaugua...................   3     1    seq
.....................................................................uggaauguCaagaaguaugua...................   2     1    seq
.....................................................................uggaauguaaagaaguauAua...................   10    1    seq
.....................................................................uggaauguaaagaagGaugua...................   6     1    seq
.....................................................................uggaaAguaaagaaguaugua...................   3     1    seq
.....................................................................uAgaauguaaagaaguaugua...................   11    1    seq
.....................................................................uggaauguaaCgaaguaugua...................   2     1    seq
.....................................................................uggaauguaaGgaaguaugua...................   103   1    seq
.....................................................................uggaauUuaaagaaguauguau..................   2     1    seq
.....................................................................uggaauguaaGgaaguauguau..................   88    1    seq
.....................................................................uggaGuguaaagaaguauguau..................   44    1    seq
.....................................................................uggaauAuaaagaaguauguau..................   7     1    seq
.....................................................................ugAaauguaaagaaguauguau..................   5     1    seq
```

```
                              Star                                   Mature
accaucuucaccauaucccaugccauaacauacuucuuuagaauuccauacugaaucuccucaacucuauggaauguaaagaaguauguauugaaacugggaccucaca
.....................................................................uggaauguaaagGaguauguau..................   36   1   seq
.....................................................................ugUaauguaaagaaguauguau..................   26   1   seq
.....................................................................uggaauguaaagaaguauCuau..................   3    1   seq
.....................................................................uggaauguaaagCaguauguau..................   2    1   seq
.....................................................................uggaauguGaagaaguauguau..................   20   1   seq
.....................................................................uggaUuguaaagaaguauguau..................   1    1   seq
.....................................................................uggaaugGaaagaaguauguau..................   2    1   seq
.....................................................................ugCaauguaaagaaguauguau..................   2    1   seq
.....................................................................uggaauguaaagaagGauguau..................   88   1   seq
.....................................................................uggaauguaaagaaguaugAau..................   7    1   seq
.....................................................................uggUauguaaagaaguauguau..................   14   1   seq
.....................................................................uggaauguaaagaagCauguau..................   19   1   seq
.....................................................................uggaauguaaaUaaguauguau..................   1    1   seq
.....................................................................uggaauguaaagaCguauguau..................   1    1   seq
.....................................................................uggaauguaaagaUguauguau..................   1    1   seq
.....................................................................uggaauguaaagaaguauUuau..................   3    1   seq
.....................................................................uggaaAguaaagaaguauguau..................   2    1   seq
.....................................................................uggaauguaaagaGguauguau..................   28   1   seq
.....................................................................uggGauguaaagaaguauguau..................   54   1   seq
.....................................................................uggaaugAaaagaaguauguau..................   2    1   seq
.....................................................................uggaauguaaagaaAuauguau..................   6    1   seq
.....................................................................uggaauguaGagaaguauguau..................   36   1   seq
.....................................................................uggaauguaaagaagAauguau..................   36   1   seq
.....................................................................uggaauguaaagaaguauAuau..................   11   1   seq
.....................................................................uggaauguaUagaaguauguau..................   3    1   seq
.....................................................................uggCauguaaagaaguauguau..................   3    1   seq
.....................................................................uggaauguaaCgaaguauguau..................   4    1   seq
.....................................................................uggaaugCaaagaaguauguau..................   17   1   seq
.....................................................................uggaauguaaagUaguauguau..................   3    1   seq
.....................................................................uggaauguaaaAaaguauguau..................   2    1   seq
.....................................................................uggaauguaaagaaguaAguau..................   1    1   seq
.....................................................................uggaaGguaaagaaguauguau..................   1    1   seq
.....................................................................uggaaCguaaagaaguauguau..................   30   1   seq
.....................................................................uggaauguaCagaaguauguau..................   3    1   seq
.....................................................................uggaauguaaagaaguaGguau..................   3    1   seq
.....................................................................uggaauguaaagaaUuauguau..................   1    1   seq
......................................................................Ugaauguaaagaaguaug.....................   1    1   seq
......................................................................Ugaauguaaagaaguaugu....................   3    1   seq
......................................................................Ugaauguaaagaaguaugua...................   1    1   seq
.......................................................................gaauguaaagaaguauguG...................   1    1   seq
.......................................................................gaauguaaagaaguauguGu..................   27   1   seq
.......................................................................gaauguaaagaaguauguGuu.................   2    1   seq
........................................................................aauguaaagaaguauguGu..................   11   1   seq
```
