# Supplementary material for: Identification and Characterization of MicroRNAs from Longitudinal Muscle and Respiratory Tree in Sea Cucumber (Apostichopus japonicus) Using High-Throughput Sequencing
Source: PLoS One. 2015 Aug 5;10(8):e0134899. doi: 10.1371/journal.pone.0134899 (PMC4526669; doi:10.1371/journal.pone.0134899)
Supplement: S1 File — (ZIP) [file pone.0134899.s002.zip › S1 File/The secondary structures of the novel miRNAs in LTM/Scaffold762_627.pdf]

Provisional ID : Scaffold762_627
Score total : 2.1
Score for star read(s) : -1.3
Score for read counts : 0
Score for mfe : 1.8
Score for randfold : 1.6
Score for cons. seed :
Total read count : 1855
Mature read count : 1855
Loop read count : 0
Star read count : 0

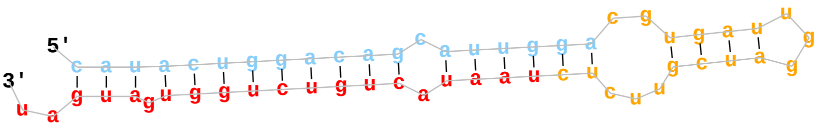

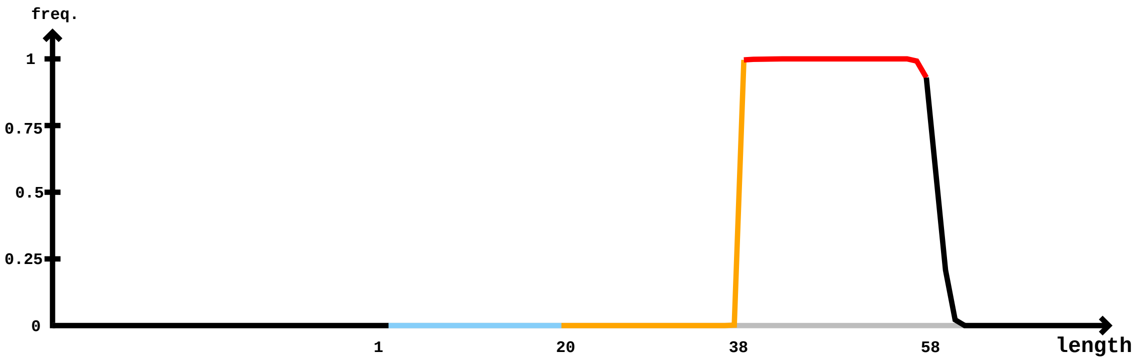


```
                                          Star                      Mature
5'- guguuugcgauuuguugagccuaguggugacaucauacuggacagcauuggacgugauuggaucguucucuaauacugucuggugaugauguugacacuggcuuuguu   -3'   exp
    ................(((((.((((..(((((((((((((((((.((((((..((((...))))...)))))).)))))))))..))))))))..)))))))))....   reads   mm    sample
    .....................................................................cuaauacugucuggugaugaC..................   1       1     seq
    .....................................................................cuaauacugucuggugaugGu..................   2       1     seq
    ......................................................................uaauacugucuggugaug....................   9       0     seq
    ......................................................................uaauacugucugAugaug....................   1       1     seq
    ......................................................................uaauacugucuggugauA....................   3       1     seq
    ......................................................................uaauacugucuggugauU....................   1       1     seq
    ......................................................................uaauGcugucuggugaug....................   1       1     seq
    ......................................................................uaauGcugucuggugauga...................   5       1     seq
    ......................................................................uaauacugucuggugaugG...................   10      1     seq
    ......................................................................uaauacugucuggugaugU...................   2       1     seq
    ......................................................................uaauacugucuggCgauga...................   1       1     seq
    ......................................................................uaauacugucuggugauga...................   90      0     seq
    ......................................................................uaauacugucAggugauga...................   1       1     seq
    ......................................................................uaUuacugucuggugauga...................   1       1     seq
    ......................................................................uaGuacugucuggugauga...................   1       1     seq
    ......................................................................uaauacugucuggAgauga...................   1       1     seq
    ......................................................................uaauacugCcuggugauga...................   1       1     seq
    ......................................................................uaauacugucuAgugauga...................   1       1     seq
    ......................................................................uaaGacugucuggugauga...................   1       1     seq
    ......................................................................uCauacugucuggugaugau..................   1       1     seq
    ......................................................................Gaauacugucuggugaugau..................   11      1     seq
    ......................................................................uaaGacugucuggugaugau..................   20      1     seq
    ......................................................................uaauacugucuAgugaugau..................   4       1     seq
    ......................................................................uaGuacugucuggugaugau..................   8       1     seq
    ......................................................................uaauacugucGggugaugau..................   1       1     seq
    ......................................................................uaUuacugucuggugaugau..................   10      1     seq
    ......................................................................uaauacugucuggAgaugau..................   6       1     seq
    ......................................................................uaauacugucuggugaugaC..................   317     1     seq
    ......................................................................uaauUcugucuggugaugau..................   2       1     seq
    ......................................................................uaauGcugucuggugaugau..................   35      1     seq
    ......................................................................uaauacAgucuggugaugau..................   1       1     seq
    ......................................................................uaaCacugucuggugaugau..................   7       1     seq
    ......................................................................uaauacugucuggugaugGu..................   14      1     seq
    ......................................................................uaauaUugucuggugaugau..................   2       1     seq
```

```
                                                Star                               Mature
guguuugcgauuuguugagccuaguggugacaucauacuggacagcauuggacgugauuggaucguucucuaauacugucuggugaugauguugacacuggcuuuguu
......................................................................uaauacugucugguUaugau..................  1   1   seq
......................................................................uaauacugucuggugGugau..................  12  1   seq
......................................................................uaauacugucuUgugaugau..................  2   1   seq
......................................................................uaauacuAucuggugaugau..................  2   1   seq
......................................................................uaauacugAcuggugaugau..................  2   1   seq
......................................................................uaauacugucuggugauUau..................  3   1   seq
......................................................................uaauacuguUuggugaugau..................  1   1   seq
......................................................................uaauacugucuggugaCgau..................  8   1   seq
......................................................................uaauacugucuggugaugaG..................  43  1   seq
......................................................................uaauacugCcuggugaugau..................  13  1   seq
......................................................................uaauacugucuggCgaugau..................  10  1   seq
......................................................................uaauacugucuggugaugCu..................  1   1   seq
......................................................................uaauacugucCggugaugau..................  7   1   seq
......................................................................uaauacugucugAugaugau..................  2   1   seq
......................................................................uaaAacugucuggugaugau..................  6   1   seq
......................................................................uaauacCgucuggugaugau..................  12  1   seq
......................................................................uaauacugucAggugaugau..................  4   1   seq
......................................................................uaauaGugucuggugaugau..................  1   1   seq
......................................................................uaauacuguAuggugaugau..................  1   1   seq
......................................................................uaauacugucuggGgaugau..................  8   1   seq
......................................................................Aaauacugucuggugaugau..................  2   1   seq
......................................................................uaauacugucuggugaugaA..................  34  1   seq
......................................................................uaauacugucuggugaugUu..................  3   1   seq
......................................................................uUauacugucuggugaugau..................  3   1   seq
......................................................................uGauacugucuggugaugau..................  11  1   seq
......................................................................uaauCcugucuggugaugau..................  1   1   seq
......................................................................uaauacugucuggugauAau..................  4   1   seq
......................................................................Caauacugucuggugaugau..................  7   1   seq
......................................................................uaauacuguAuggugaugaug.................  1   1   seq
......................................................................uaauUcugucuggugaugaug.................  3   1   seq
......................................................................uaauacugucuggugauAaug.................  8   1   seq
......................................................................uaauacugucuggAgaugaug.................  15  1   seq
......................................................................uaauacugCcuggugaugaug.................  26  1   seq
......................................................................uaauacugucuggugaugCug.................  1   1   seq
......................................................................uaauacugucuggugaugUug.................  8   1   seq
......................................................................uaauacugucGggugaugaug.................  3   1   seq
......................................................................uaauacugucuAgugaugaug.................  9   1   seq
......................................................................uaauacugucCggugaugaug.................  24  1   seq
......................................................................uaauacugucuUgugaugaug.................  2   1   seq
......................................................................uaauacuguUuggugaugaug.................  3   1   seq
......................................................................uaUuacugucuggugaugaug.................  37  1   seq
......................................................................uaauacuUucuggugaugaug.................  1   1   seq
......................................................................uaauacugucugUugaugaug.................  2   1   seq
......................................................................uGauacugucuggugaugaug.................  34  1   seq
......................................................................uaauaUugucuggugaugaug.................  3   1   seq
......................................................................uaauacugucuggGgaugaug.................  7   1   seq
......................................................................uaauacugucuggugauUaug.................  4   1   seq
......................................................................uaauaGugucuggugaugaug.................  1   1   seq
......................................................................uaauacugucuggugaugaAg.................  7   1   seq
......................................................................uaauaAugucuggugaugaug.................  2   1   seq
......................................................................uaaGacugucuggugaugaug.................  44  1   seq
......................................................................uaauacugucuggugaAgaug.................  2   1   seq
......................................................................uUauacugucuggugaugaug.................  1   1   seq
......................................................................uaauacugucuggugaugGug.................  57  1   seq
......................................................................uaauacugucuggugaGgaug.................  1   1   seq
......................................................................uaauacugucuggugGugaug.................  33  1   seq
......................................................................uaauacAgucuggugaugaug.................  6   1   seq
......................................................................uaaCacugucuggugaugaug.................  22  1   seq
......................................................................uaaAacugucuggugaugaug.................  11  1   seq
......................................................................uaGuacugucuggugaugaug.................  30  1   seq
......................................................................uaauacGgucuggugaugaug.................  8   1   seq
......................................................................uaauCcugucuggugaugaug.................  2   1   seq
......................................................................uaauacugucugguUaugaug.................  3   1   seq
......................................................................uaauacugucuggugaCgaug.................  27  1   seq
......................................................................uaauacugucuggugaugaGg.................  6   1   seq
......................................................................uaauacugucuCgugaugaug.................  2   1   seq
......................................................................uaauacugucuggugCugaug.................  2   1   seq
......................................................................uaauacugucAggugaugaug.................  5   1   seq
......................................................................uaauacugucuggugUugaug.................  6   1   seq
......................................................................uaauacugucugAugaugaug.................  10  1   seq
```

```
                                 Star                                 Mature
guguuugcgauuuguugagccuaguggugacaucauacuggacagcauuggacgugauuggaucguucucuaauacugucuggugaugauguugacacuggcuuuguu
......................................................................uaauacugGcuggugaugaug.................  4    1  seq
......................................................................uaauacugucuggugaugaCg.................  52   1  seq
......................................................................uaauacugucuggCgaugaug.................  29   1  seq
......................................................................uaauacugAcuggugaugaug.................  6    1  seq
......................................................................uaauacCgucuggugaugaug.................  35   1  seq
......................................................................uaauacuAucuggugaugaug.................  5    1  seq
......................................................................uaauGcugucuggugaugaug.................  78   1  seq
......................................................................uCauacugucuggugaugaug.................  1    1  seq
......................................................................uaCuacugucuggugaugaug.................  1    1  seq
......................................................................uaauacugucuggugGugaugu................  15   1  seq
......................................................................uaauGcugucuggugaugaugu................  144  1  seq
......................................................................uaaGacugucuggugaugaugu................  19   1  seq
......................................................................uaauacugucuggugauAaugu................  4    1  seq
......................................................................uaauacugucuggugUugaugu................  1    1  seq
......................................................................uaauacugucugguUaugaugu................  1    1  seq
......................................................................uaaCacugucuggugaugaugu................  13   1  seq
......................................................................uaauacugucuggugauUaugu................  5    1  seq
......................................................................uaauacugAcuggugaugaugu................  3    1  seq
......................................................................uaauacugucuggAgaugaugu................  6    1  seq
......................................................................uaauacugucuggCgaugaugu................  9    1  seq
......................................................................uaauacugucuAgugaugaugu................  5    1  seq
......................................................................uaauacugucuUgugaugaugu................  1    1  seq
......................................................................uaauCcugucuggugaugaugu................  2    1  seq
......................................................................uaauacugucugguCaugaugu................  1    1  seq
......................................................................uaauacugucuggugaugGugu................  20   1  seq
......................................................................uaauacugucuggugaugaCgu................  9    1  seq
......................................................................uaauacugucuggugaugaGgu................  1    1  seq
......................................................................uaGuacugucuggugaugaugu................  9    1  seq
......................................................................uaauacuAucuggugaugaugu................  1    1  seq
......................................................................uaauacugCcuggugaugaugu................  12   1  seq
......................................................................uaauacugucGggugaugaugu................  1    1  seq
......................................................................uaauacugucuggugaugCugu................  2    1  seq
......................................................................uaauacGgucuggugaugaugu................  4    1  seq
......................................................................uaauacugucCggugaugaugu................  5    1  seq
......................................................................uaauaUugucuggugaugaugu................  3    1  seq
......................................................................uaauacugucuggugaugUugu................  1    1  seq
......................................................................uaauacuguGuggugaugaugu................  1    1  seq
......................................................................uaauacuguAuggugaugaugu................  1    1  seq
......................................................................uaauacugucugAugaugaugu................  1    1  seq
......................................................................uaauacugGcuggugaugaugu................  2    1  seq
......................................................................uaauacCgucuggugaugaugu................  9    1  seq
......................................................................uaauacugucuggugaCgaugu................  8    1  seq
......................................................................uaUuacugucuggugaugaugu................  18   1  seq
......................................................................uaauacuguUuggugaugaugu................  3    1  seq
......................................................................uaauacugucuggGgaugaugu................  2    1  seq
......................................................................uaauUcugucuggugaugaugu................  3    1  seq
......................................................................uaauacugucuggugUugauguu...............  2    1  seq
......................................................................uaauacugucuCgugaugauguu...............  1    1  seq
......................................................................uaauacugCcuggugaugauguu...............  7    1  seq
......................................................................uaauacugucuggugaAgauguu...............  2    1  seq
......................................................................uaauacugucCggugaugauguu...............  2    1  seq
......................................................................uaauacugucugAugaugauguu...............  1    1  seq
......................................................................uaauacugucuggAgaugauguu...............  1    1  seq
......................................................................uaauacugucuggugGugauguu...............  4    1  seq
......................................................................uaauacugAcuggugaugauguu...............  3    1  seq
......................................................................uaauacCgucuggugaugauguu...............  4    1  seq
......................................................................uaauacugucuggugaCgauguu...............  3    1  seq
......................................................................uaauacugucuggCgaugauguu...............  1    1  seq
......................................................................uaauacugucuggGgaugauguu...............  1    1  seq
......................................................................uaauacugucAggugaugauguu...............  1    1  seq
......................................................................uaauacGgucuggugaugauguu...............  1    1  seq
......................................................................uaauacugucuAgugaugauguu...............  2    1  seq
......................................................................uaauacugucuUgugaugauguu...............  1    1  seq
.......................................................................aauacugucuggugaugau..................  1    0  seq
.......................................................................Uauacugucuggugaugaug.................  1    1  seq
.......................................................................aauGcugucuggugaugauguu...............  2    1  seq
........................................................................auGcugucuggugaugauguu...............  1    1  seq
.........................................................................uacugucuggugaugaug.................  1    0  seq
..........................................................................Gcugucuggugaugaugu................  1    1  seq
```
