# Supplementary material for: Identification and Characterization of MicroRNAs from Longitudinal Muscle and Respiratory Tree in Sea Cucumber (Apostichopus japonicus) Using High-Throughput Sequencing
Source: PLoS One. 2015 Aug 5;10(8):e0134899. doi: 10.1371/journal.pone.0134899 (PMC4526669; doi:10.1371/journal.pone.0134899)
Supplement: S1 File — (ZIP) [file pone.0134899.s002.zip › S1 File/The secondary structures of the novel miRNAs in LTM/Scaffold823_655.pdf]

Provisional ID	:	Scaffold823_655
Score total	:	2.4
Score for star read(s)	:	-1.3
Score for read counts	:	0
Score for mfe	:	2.1
Score for randfold	:	1.6
Score for cons. seed	:	
Total read count	:	72
Mature read count	:	72
Loop read count	:	0
Star read count	:	0

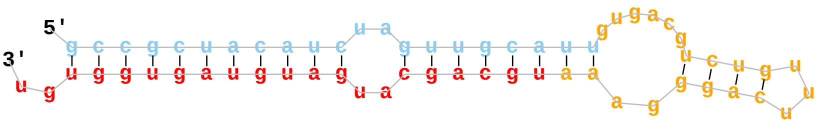

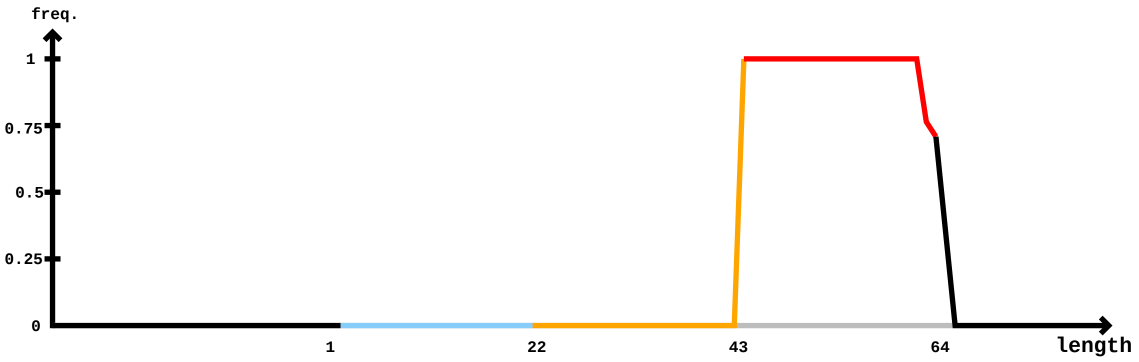

Star　　　　Mature

```
5'- aagcugucauugaugucuacuguucuacgccgcuacaucuaguugcauugugacgucuguuucagggaaaugcagcaugauguaguggugu agcucaguaagaccauu -3'   exp
    ..............((((((((..(((((((((((((((..((((((((......((((...))))..))))))))..)))))))))))))))..)))).))))....   reads   mm   sample
    ......................................................................ugcagcaugauguaguggu...................   13      0    seq
    ......................................................................ugcagcaugauguaguggC...................   3       1    seq
    ......................................................................ugcagcaugauguaguggG...................   1       1    seq
    ......................................................................ugcagcaugauguagugguA..................   2       1    seq
    ......................................................................ugcagcaugauguagCggug..................   1       1    seq
    ......................................................................ugcagcaugauguagugguU..................   1       1    seq
    ......................................................................ugcagcGugauguaguggugu.................   2       1    seq
    ......................................................................ugcagcaugauguagCggugu.................   1       1    seq
    ......................................................................ugcagcaugauguGguggugu.................   1       1    seq
    ......................................................................ugcGgcaugauguaguggugu.................   46      1    seq
    ......................................................................ugcagcaugauguagAggugu.................   1       1    seq
```
