# Supplementary material for: Identification and Characterization of MicroRNAs from Longitudinal Muscle and Respiratory Tree in Sea Cucumber (Apostichopus japonicus) Using High-Throughput Sequencing
Source: PLoS One. 2015 Aug 5;10(8):e0134899. doi: 10.1371/journal.pone.0134899 (PMC4526669; doi:10.1371/journal.pone.0134899)
Supplement: S1 File — (ZIP) [file pone.0134899.s002.zip › S1 File/The secondary structures of the novel miRNAs in LTM/Scaffold838_659.pdf]

Provisional ID	:	Scaffold838_659
Score total	:	1.2
Score for star read(s)	:	-1.3
Score for read counts	:	0
Score for mfe	:	0.9
Score for randfold	:	1.6
Score for cons. seed	:	
Total read count	:	3461
Mature read count	:	3461
Loop read count	:	0
Star read count	:	0

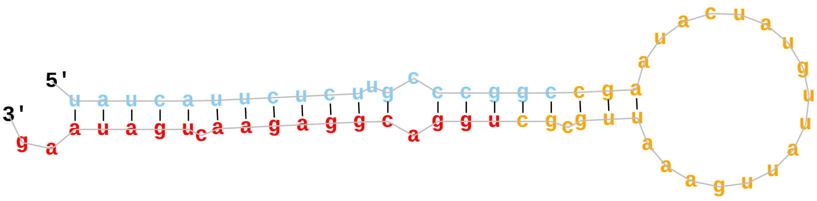

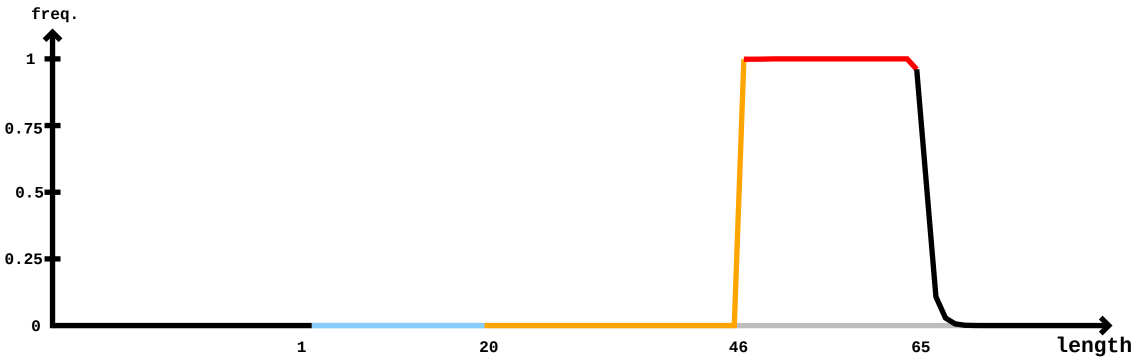

Star   Mature

```
5'- aucauauuugucuuuuaucuuuccuuaucauucucuugcccggccgaauacuauguuauugaaauugcgcuggacggagaacugauaagggcuuauauaagacacgug   -3'    exp
    ....(((.((((((.(((...(((((((((((((((.(.((((((((.................))).))))).))))))).)))))))))...))).)))))).)))    reads    mm    sample
    ......................................................................uggacggagaaUugauaa....................    1        1     seq
    ......................................................................uggacggaAaacugauaa....................    1        1     seq
    ......................................................................uggacggagaacugauaU....................    22       1     seq
    ......................................................................uggacggagaacugauaG....................    12       1     seq
    ......................................................................uggacggagaacugauaC....................    4        1     seq
    ......................................................................uggacggagaacugauaa....................    95       0     seq
    ......................................................................ugUacggagaacugauaa....................    1        1     seq
    ......................................................................uggacgUagaacugauaag...................    1        1     seq
    ......................................................................Gggacggagaacugauaag...................    3        1     seq
    ......................................................................uggacggaAaacugauaag...................    1        1     seq
    ......................................................................uggacggagaacugGuaag...................    3        1     seq
    ......................................................................uggacggagaacugaCaag...................    1        1     seq
    ......................................................................uggacggagCacugauaag...................    2        1     seq
    ......................................................................uggacggagaacugauaaC...................    5        1     seq
    ......................................................................uggaGggagaacugauaag...................    1        1     seq
    ......................................................................uggacggagaacugauaaU...................    46       1     seq
    ......................................................................ugAacggagaacugauaag...................    1        1     seq
    ......................................................................uggGcggagaacugauaag...................    4        1     seq
    ......................................................................uggacggGgaacugauaag...................    7        1     seq
    ......................................................................uggacggagaacugauaGg...................    6        1     seq
    ......................................................................uggacggagaacugauUag...................    2        1     seq
    ......................................................................Aggacggagaacugauaag...................    1        1     seq
    ......................................................................uggacggagaGcugauaag...................    1        1     seq
    ......................................................................uggaAggagaacugauaag...................    1        1     seq
    ......................................................................uggacggagGacugauaag...................    2        1     seq
    ......................................................................uggacggagaacugauaag...................    1130     0     seq
    ......................................................................uggacAgagaacugauaag...................    2        1     seq
    ......................................................................uAgacggagaacugauaag...................    4        1     seq
    ......................................................................uUgacggagaacugauaag...................    7        1     seq
    ......................................................................uggacggagaacugauGag...................    3        1     seq
    ......................................................................uggacggaCaacugauaag...................    1        1     seq
    ......................................................................uggacggagUacugauaag...................    1        1     seq
    ......................................................................uggacgCagaacugauaag...................    1        1     seq
    ......................................................................Cggacggagaacugauaag...................    3        1     seq
```

```
                              Star                                          Mature
aucauauuugucuuuuaucuuuccuuaucauucucuugcccggccgaauacuauguuauugaaauugcgcuggacggagaacugauaagggcuuauauaagacacgug
......................................................................uggacggagaaUugauaag...................   2     1     seq
......................................................................uggacggagaacCgauaag...................   3     1     seq
......................................................................ugUacggagaacugauaag...................   5     1     seq
......................................................................uggacggagaacugauaaA...................   85    1     seq
......................................................................uggacggagaacugUuaag...................   1     1     seq
......................................................................uggaUggagaacugauaag...................   1     1     seq
......................................................................uggacggagaUcugauaag...................   2     1     seq
......................................................................uggacggagaaAugauaagg..................   2     1     seq
......................................................................uggacggagaacugauUagg..................   3     1     seq
......................................................................uggacggagaacugauGagg..................   27    1     seq
......................................................................uggacggaAaacugauaagg..................   7     1     seq
......................................................................Cggacggagaacugauaagg..................   50    1     seq
......................................................................uggacUgagaacugauaagg..................   3     1     seq
......................................................................uggacAgagaacugauaagg..................   16    1     seq
......................................................................uggacggaUaacugauaagg..................   1     1     seq
......................................................................uUgacggagaacugauaagg..................   25    1     seq
......................................................................uggGcggagaacugauaagg..................   46    1     seq
......................................................................uggacggagaacugauaagC..................   34    1     seq
......................................................................uggacggagaacugaAaagg..................   2     1     seq
......................................................................uggacggagCacugauaagg..................   1     1     seq
......................................................................uggacggagaacGgauaagg..................   1     1     seq
......................................................................uggacggagaacuAauaagg..................   5     1     seq
......................................................................uggacggagaacugauaagA..................   940   1     seq
......................................................................uggacggagaacugauaaCg..................   7     1     seq
......................................................................uggacggagUacugauaagg..................   2     1     seq
......................................................................ugUacggagaacugauaagg..................   27    1     seq
......................................................................uggaAggagaacugauaagg..................   1     1     seq
......................................................................uggacggagaacugauaaUg..................   4     1     seq
......................................................................uggacCgagaacugauaagg..................   2     1     seq
......................................................................uggacgAagaacugauaagg..................   4     1     seq
......................................................................uggacggCgaacugauaagg..................   1     1     seq
......................................................................ugAacggagaacugauaagg..................   8     1     seq
......................................................................uggUcggagaacugauaagg..................   12    1     seq
......................................................................uggaUggagaacugauaagg..................   11    1     seq
......................................................................Aggacggagaacugauaagg..................   11    1     seq
......................................................................uggacggagaacugUuaagg..................   8     1     seq
......................................................................uggCcggagaacugauaagg..................   4     1     seq
......................................................................uggacggagaacugauaagU..................   78    1     seq
......................................................................uggacggagaacCgauaagg..................   28    1     seq
......................................................................uggacggagaacAgauaagg..................   7     1     seq
......................................................................uggacggagaaGugauaagg..................   1     1     seq
......................................................................uggacggagaacugauaUgg..................   7     1     seq
......................................................................uggacggUgaacugauaagg..................   7     1     seq
......................................................................uggacggagaacugCuaagg..................   2     1     seq
......................................................................uggacggagaGcugauaagg..................   36    1     seq
......................................................................Gggacggagaacugauaagg..................   26    1     seq
......................................................................uggacggagaacugaCaagg..................   10    1     seq
......................................................................uCgacggagaacugauaagg..................   6     1     seq
......................................................................uAgacggagaacugauaagg..................   17    1     seq
......................................................................uggacggagGacugauaagg..................   47    1     seq
......................................................................uggacggagaacuUauaagg..................   1     1     seq
......................................................................uggacgUagaacugauaagg..................   5     1     seq
......................................................................uggacggagaacugGuaagg..................   34    1     seq
......................................................................uggacggagaUcugauaagg..................   1     1     seq
......................................................................uggacggGgaacugauaagg..................   35    1     seq
......................................................................uggacggagaacugaCaaggg.................   5     1     seq
......................................................................uggCcggagaacugauaaggg.................   1     1     seq
......................................................................uggaUggagaacugauaaggg.................   4     1     seq
......................................................................uggacggagaacCgauaaggg.................   22    1     seq
......................................................................uggGcggagaacugauaaggg.................   28    1     seq
......................................................................uggacggagaaAugauaaggg.................   1     1     seq
......................................................................uggaAggagaacugauaaggg.................   3     1     seq
......................................................................uggacggagaaGugauaaggg.................   2     1     seq
......................................................................uggacggagaacugauaagAg.................   5     1     seq
......................................................................uggacgUagaacugauaaggg.................   5     1     seq
......................................................................uggacggagaacGgauaaggg.................   2     1     seq
......................................................................uggacggagaacugauaagCg.................   3     1     seq
......................................................................uggacggagaCcugauaaggg.................   1     1     seq
......................................................................uggacggagaacuUauaaggg.................   1     1     seq
......................................................................uggacggagaacugauaaUgg.................   1     1     seq
```

```
                         Star                                          Mature
aucauauuugucuuuuaucuuuccuuaucauucucuugcccggccgaauacuauguuauugaaauugcgcuggacggagaacugauaagggcuuauauaagacacgug
......................................................................ugUacggagaacugauaaggg.................   9   1   seq
......................................................................uggacggagaacugCuaaggg.................   2   1   seq
......................................................................uAgacggagaacugauaaggg.................   3   1   seq
......................................................................uggaGggagaacugauaaggg.................   2   1   seq
......................................................................uggacggagaacuAauaaggg.................   6   1   seq
......................................................................uggacggagaacAgauaaggg.................   5   1   seq
......................................................................uggUcggagaacugauaaggg.................   5   1   seq
......................................................................uggacggagaacugauaagUg.................   2   1   seq
......................................................................uggacggagUacugauaaggg.................   3   1   seq
......................................................................uggacggagaacugGuaaggg.................   13  1   seq
......................................................................uggacUgagaacugauaaggg.................   4   1   seq
......................................................................uggacggagaacugauUaggg.................   2   1   seq
......................................................................uggacggagaacugauGaggg.................   28  1   seq
......................................................................uggacggUgaacugauaaggg.................   6   1   seq
......................................................................ugAacggagaacugauaaggg.................   2   1   seq
......................................................................uCgacggagaacugauaaggg.................   4   1   seq
......................................................................uggacgAagaacugauaaggg.................   6   1   seq
......................................................................uggacggagaacugauaUggg.................   1   1   seq
......................................................................uggacggaAaacugauaaggg.................   5   1   seq
......................................................................uggacAgagaacugauaaggg.................   8   1   seq
......................................................................uggacggagaGcugauaaggg.................   16  1   seq
......................................................................uggacggagCacugauaaggg.................   1   1   seq
......................................................................uUgacggagaacugauaaggg.................   16  1   seq
......................................................................uggacggagGacugauaaggg.................   22  1   seq
......................................................................uggacggagaacugUuaaggg.................   3   1   seq
......................................................................uggacggGgaacugauaaggg.................   17  1   seq
......................................................................uggacggagaacuAauaagggc................   2   1   seq
......................................................................uggacggagaacCgauaagggc................   2   1   seq
......................................................................uggacggagaacugauaUgggc................   1   1   seq
......................................................................uggacggagGacugauaagggc................   4   1   seq
......................................................................ugAacggagaacugauaagggc................   3   1   seq
......................................................................uggaUggagaacugauaagggc................   1   1   seq
......................................................................uggacggagaacAgauaagggc................   1   1   seq
......................................................................uggacggagaacugauGagggc................   4   1   seq
......................................................................uggacggagCacugauaagggc................   1   1   seq
......................................................................uggacggagaacugGuaagggc................   8   1   seq
......................................................................uggacggagaUcugauaagggc................   1   1   seq
......................................................................uggacggagaacugaCaagggc................   3   1   seq
......................................................................uggacggagaacugauaagAgc................   1   1   seq
......................................................................uggacggagUacugauaagggc................   1   1   seq
......................................................................uggUcggagaacugauaagggc................   2   1   seq
......................................................................uggacggUgaacugauaagggc................   2   1   seq
......................................................................uggacggagaacugaAaagggc................   2   1   seq
......................................................................uggacAgagaacugauaagggc................   5   1   seq
......................................................................uggacggGgaacugauaagggc................   9   1   seq
......................................................................ugUacggagaacugauaagggc................   5   1   seq
......................................................................uggacggagaGcugauaagggc................   5   1   seq
......................................................................uggGcggagaacugauaagggc................   9   1   seq
......................................................................uggacgAagaacugauaagggc................   1   1   seq
......................................................................uggCcggagaacugauaagggc................   2   1   seq
......................................................................uggacggaAaacugauaagggc................   1   1   seq
......................................................................uggacggagaacugaAaagggcu...............   1   1   seq
......................................................................uggUcggagaacugauaagggcu...............   1   1   seq
......................................................................uggGcggagaacugauaagggcu...............   1   1   seq
......................................................................uggacggagaacCgauaagggcu...............   1   1   seq
......................................................................uggacggagaacugauaUgggcu...............   1   1   seq
......................................................................uggacUgagaacugauaagggcu...............   1   1   seq
......................................................................uggacggagaGcugauaagggcu...............   1   1   seq
......................................................................uggCcggagaacugauaagggcu...............   3   1   seq
......................................................................uggacAgagaacugauaagggcu...............   1   1   seq
......................................................................uggacggagaacugGuaagggcu...............   2   1   seq
......................................................................uggacggagaacugaCaagggcu...............   1   1   seq
......................................................................uggacggagaacugauGagggcu...............   2   1   seq
......................................................................uggacggagaacuAauaagggcuu..............   1   1   seq
......................................................................uggacggagaGcugauaagggcuu..............   1   1   seq
......................................................................uggacggagaacugaAaagggcuu..............   1   1   seq
........................................................................gacggagaacugauGagggcu...............   1   1   seq
.........................................................................acggagaacugauaaggg.................   1   0   seq
.........................................................................acggagaacugauaagggcu...............   1   0   seq
.........................................................................acggagaacugauaagggcuuG.............   1   1   seq
```

Star Mature

aucauauuugucuuuuaucuuuccuuaucauucucuugcccggccgaauacuauguuauugaaauugcgcuggacggagaacugauaagggcuuauauaagacacgug
