# Supplementary material for: Identification and Characterization of MicroRNAs from Longitudinal Muscle and Respiratory Tree in Sea Cucumber (Apostichopus japonicus) Using High-Throughput Sequencing
Source: PLoS One. 2015 Aug 5;10(8):e0134899. doi: 10.1371/journal.pone.0134899 (PMC4526669; doi:10.1371/journal.pone.0134899)
Supplement: S1 File — (ZIP) [file pone.0134899.s002.zip › S1 File/The secondary structures of the novel miRNAs in LTM/Scaffold912_690.pdf]

Provisional ID	:	Scaffold912_690
Score total	:	1799.8
Score for star read(s)	:	3.9
Score for read counts	:	1792.2
Score for mfe	:	2.1
Score for randfold	:	1.6
Score for cons. seed	:	
Total read count	:	3527
Mature read count	:	3438
Loop read count	:	0
Star read count	:	89

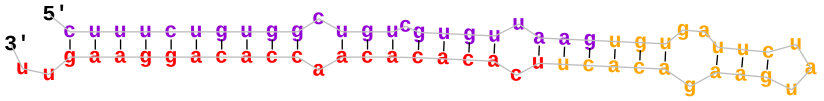

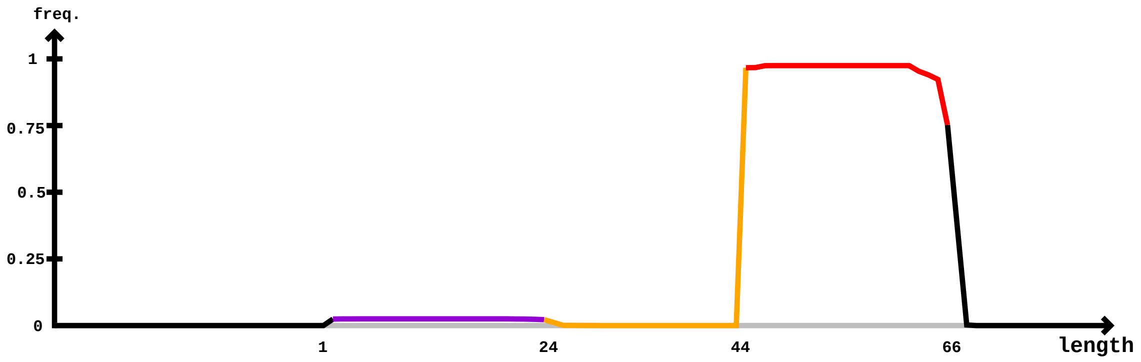

Star   Mature

```
5'- gauagagaagagaggcuacuugcggaccuuucuguggcugucguguuaagugugauucuaugaagacacuucacacacaaccacaggaaguuuugcacgguagccaug   -3'   obs
    gauagagaagagaggcuacuugcggaccuuucuguggcugucguguuaagugugauucuaugaagacacuucacacacaaccacaggaaguuuugcacgguagccaug         exp
    .............(((((((((((((.((((((((((.(((.((((.((((((..(((...))).)))))).))))))).)))))))))).)))))).)))))))...   reads   mm   sample
    ...........................cuuucuguggcugucgugu..............................................................   2       0    seq
    ...........................cuuucuguggcugucguguu.............................................................   1       0    seq
    ...........................cuuuUuguggcugucguguua............................................................   1       1    seq
    ...........................cuuucuguggcugucguguua............................................................   2       0    seq
    ...........................cuuucuguggcugucguguuaa...........................................................   4       0    seq
    ...........................cuuuUuguggcugucguguuaag..........................................................   3       1    seq
    ...........................cuuucuguggcugucguguuaaA..........................................................   2       1    seq
    ...........................cuuucuguggUugucguguuaag..........................................................   4       1    seq
    ...........................cuuucuguggcugucgAguuaag..........................................................   2       1    seq
    ...........................cuuCcuguggcugucguguuaag..........................................................   1       1    seq
    ...........................cAuucuguggcugucguguuaag..........................................................   1       1    seq
    ...........................cuuucuguggcugucguguuaag..........................................................   59      0    seq
    ...........................cuuucuguggcugucguguuaagu.........................................................   3       0    seq
    ...........................cuuucuguggcuAucguguuaaguguga.....................................................   1       1    seq
    ............................uuucuguggUugucguguuaa...........................................................   1       1    seq
    ............................uuucuguggcugucguguuaag..........................................................   1       0    seq
    ..............................ucuguggcugucguguuaagugu.......................................................   1       0    seq
    ......................................................................ucacacacaaccacaggG....................   2       1    seq
    ......................................................................Gcacacacaaccacagga....................   1       1    seq
    ......................................................................ucacacacaaccacagga....................   68      0    seq
    ......................................................................ucacacGcaaccacagga....................   1       1    seq
    ......................................................................ucacacacaacUacagga....................   1       1    seq
    ......................................................................ucacacacaaccacGgga....................   1       1    seq
    ......................................................................ucacacacaaccacaggaG...................   2       1    seq
    ......................................................................ucacacacaaccacaggaa...................   43      0    seq
    ......................................................................ucacacacaacAacaggaa...................   1       1    seq
    ......................................................................ucacacUcaaccacaggaa...................   1       1    seq
    ......................................................................ucacacacaaccacaggaU...................   1       1    seq
    ......................................................................ucacacacGaccacaggaag..................   1       1    seq
    ......................................................................ucacaGacaaccacaggaag..................   1       1    seq
    ......................................................................ucacacacaaccacaggaaU..................   30      1    seq
    ......................................................................ucUcacacaaccacaggaag..................   1       1    seq
    ......................................................................ucacacacaaccacGggaag..................   1       1    seq
```

Star / Mature			
gauagagaagagaggcuacuugcggaccuuucuguggcugucguguuaagugugauucuaugaagacacuucacacacaaccacaggaaguuuugcacgguagccaug			
....ucacacacaaccacaggaaA..................	18	1	seq
....Ccacacacaaccacaggaag..................	1	1	seq
....ucacGcacaaccacaggaag..................	1	1	seq
....ucGcacacaaccacaggaag..................	1	1	seq
....ucacacacaaccacaggaaC..................	3	1	seq
....ucacacGcaaccacaggaag..................	1	1	seq
....ucacacacaUccacaggaagu.................	8	1	seq
....ucacacacaaccacaggaCgu.................	4	1	seq
....ucacacacaaccacagUaagu.................	1	1	seq
....ucacacacaaccacaggaUgu.................	6	1	seq
....ucacacUcaaccacaggaagu.................	11	1	seq
....ucacacacGaccacaggaagu.................	43	1	seq
....ucacacacaaccaUaggaagu.................	9	1	seq
....ucacaUacaaccacaggaagu.................	13	1	seq
....ucacacacaaccacaCgaagu.................	1	1	seq
....ucacacacaaccacaAgaagu.................	9	1	seq
....ucacacacaaccaAaggaagu.................	1	1	seq
....ucacacacaaccacaggGagu.................	37	1	seq
....ucacacacaaccacUggaagu.................	6	1	seq
....ucacacacaaGcacaggaagu.................	2	1	seq
....ucacaAacaaccacaggaagu.................	1	1	seq
....ucacacacaaccacGggaagu.................	52	1	seq
....ucacacacaaccacaggCagu.................	1	1	seq
....ucUcacacaaccacaggaagu.................	40	1	seq
....ucacacacaCccacaggaagu.................	1	1	seq
....ucacacacaaccacCggaagu.................	1	1	seq
....ucacacGcaaccacaggaagu.................	38	1	seq
....ucacacacaaccUcaggaagu.................	7	1	seq
....ucacacCcaaccacaggaagu.................	1	1	seq
....ucacacacaaccacagAaagu.................	8	1	seq
....uUacacacaaccacaggaagu.................	10	1	seq
....ucacUcacaaccacaggaagu.................	8	1	seq
....ucaGacacaaccacaggaagu.................	10	1	seq
....uGacacacaaccacaggaagu.................	7	1	seq
....ucacacacaaccacaggaaUu.................	6	1	seq
....ucGcacacaaccacaggaagu.................	36	1	seq
....ucacacacaaccCcaggaagu.................	3	1	seq
....ucaAacacaaccacaggaagu.................	1	1	seq
....ucaUacacaaccacaggaagu.................	26	1	seq
....ucCcacacaaccacaggaagu.................	2	1	seq
....ucacacacaGccacaggaagu.................	42	1	seq
....ucacacacaacUacaggaagu.................	13	1	seq
....ucacacacaaUcacaggaagu.................	7	1	seq
....ucacacacaaccacaUgaagu.................	1	1	seq
....ucacacacCaccacaggaagu.................	2	1	seq
....ucacacacaaccacaggaGgu.................	36	1	seq
....ucacacacaacAacaggaagu.................	2	1	seq
....ucacacacaaccacaggUagu.................	3	1	seq
....ucacaGacaaccacaggaagu.................	1	1	seq
....ucacacaUaaccacaggaagu.................	15	1	seq
....ucacacacUaccacaggaagu.................	5	1	seq
....ucacacacaaccacaggaaAu.................	3	1	seq
....ucacGcacaaccacaggaagu.................	54	1	seq
....ucacacacaaccacaggaaCu.................	3	1	seq
....ucacacacaaccacaUgaaguu................	8	1	seq
....ucacacacaaccacaggCaguu................	9	1	seq
....ucacacacaaccacCggaaguu................	14	1	seq
....ucaGacacaaccacaggaaguu................	41	1	seq
....ucacacacaaccacaggaGguu................	161	1	seq
....ucacacGcaaccacaggaaguu................	191	1	seq
....ucacacacaaccCcaggaaguu................	16	1	seq
....ucacacacaaccaGaggaaguu................	1	1	seq
....ucacacacaaccacaggaaUuu................	41	1	seq
....ucaAacacaaccacaggaaguu................	1	1	seq
....ucacacacaaccacaggGaguu................	174	1	seq
....ucacacaGaaccacaggaaguu................	10	1	seq
....ucacaGacaaccacaggaaguu................	6	1	seq
....ucacacacaaccacagAaaguu................	28	1	seq
....ucacacacUaccacaggaaguu................	27	1	seq
....ucacCcacaaccacaggaaguu................	6	1	seq

Star Mature

```
gauagagaagagaggcuacuugcggaccuuucuguggcugucguguuaagugugauucuaugaagacacuucacacacaaccacaggaaguuuugcacgguagccaug
......................................................................ucacacacaaccacaggaCguu................	3	1	seq
......................................................................ucacacacaaccGcaggaaguu................	107	1	seq
......................................................................ucCcacacaaccacaggaaguu................	7	1	seq
......................................................................ucacacacaacGacaggaaguu................	3	1	seq
......................................................................ucacaAacaaccacaggaaguu................	10	1	seq
......................................................................ucacacacaaccacUggaaguu................	41	1	seq
......................................................................ucacacacaaAcacaggaaguu................	8	1	seq
......................................................................ucacacaUaaccacaggaaguu................	47	1	seq
......................................................................ucacacacaaccacagUaaguu................	12	1	seq
......................................................................ucacacacGaccacaggaaguu................	153	1	seq
......................................................................ucacacacaaccacaggaUguu................	17	1	seq
......................................................................ucacacacaaccacaggaaCuu................	2	1	seq
......................................................................ucacacacaaccacaCgaaguu................	2	1	seq
......................................................................ucacacacaaccUcaggaaguu................	14	1	seq
......................................................................ucacacCcaaccacaggaaguu................	7	1	seq
......................................................................ucacacacaaccaUaggaaguu................	56	1	seq
......................................................................ucacacUcaaccacaggaaguu................	30	1	seq
......................................................................ucacGcacaaccacaggaaguu................	197	1	seq
......................................................................ucacacacaaccacaAgaaguu................	59	1	seq
......................................................................ucacacacaaccaAaggaaguu................	11	1	seq
......................................................................ucacacacaaccacGggaaguu................	164	1	seq
......................................................................ucacacacaaccacagCaaguu................	2	1	seq
......................................................................ucacacaAaaccacaggaaguu................	3	1	seq
......................................................................ucacacacaaUcacaggaaguu................	36	1	seq
......................................................................ucacaUacaaccacaggaaguu................	37	1	seq
......................................................................ucacUcacaaccacaggaaguu................	38	1	seq
......................................................................ucacacacaacAacaggaaguu................	2	1	seq
......................................................................ucGcacacaaccacaggaaguu................	165	1	seq
......................................................................ucacacacaUccacaggaaguu................	18	1	seq
......................................................................ucacacacaaccacaggaaAuu................	32	1	seq
......................................................................ucUcacacaaccacaggaaguu................	170	1	seq
......................................................................ucacacacaaccacaggUaguu................	17	1	seq
......................................................................ucacacacaaGcacaggaaguu................	2	1	seq
......................................................................ucacacacaacUacaggaaguu................	54	1	seq
......................................................................ucacacacaCccacaggaaguu................	3	1	seq
......................................................................ucacacacaGccacaggaaguu................	174	1	seq
......................................................................ucaUacacaaccacaggaaguu................	93	1	seq
......................................................................ucacacacCaccacaggaaguu................	16	1	seq
......................................................................ucacaAacaaccacaggaaguuu...............	1	1	seq
......................................................................ucacacacCaccacaggaaguuu...............	1	1	seq
......................................................................ucacacacaaccacUggaaguuu...............	1	1	seq
......................................................................ucacacacaaUcacaggaaguuu...............	3	1	seq
......................................................................ucacacacaaGcacaggaaguuu...............	1	1	seq
......................................................................ucaUacacaaccacaggaaguuu...............	4	1	seq
......................................................................ucacGcacaaccacaggaaguuu...............	7	1	seq
......................................................................ucacacacaaccacaggUaguuu...............	1	1	seq
......................................................................ucacacacUaccacaggaaguuu...............	2	1	seq
......................................................................ucacacacaGccacaggaaguuu...............	7	1	seq
......................................................................ucacacacaaccCcaggaaguuu...............	2	1	seq
......................................................................ucacacCcaaccacaggaaguuu...............	1	1	seq
......................................................................ucacacaUaaccacaggaaguuu...............	2	1	seq
......................................................................ucacacacaaccacaAgaaguuu...............	3	1	seq
......................................................................ucacacacaaccGcaggaaguuu...............	2	1	seq
......................................................................ucacacacaaccacGggaaguuu...............	9	1	seq
......................................................................ucGcacacaaccacaggaaguuu...............	5	1	seq
......................................................................ucacacGcaaccacaggaaguuu...............	3	1	seq
......................................................................ucacacacaaccacaggaaUuuu...............	1	1	seq
......................................................................ucaGacacaaccacaggaaguuu...............	1	1	seq
......................................................................ucUcacacaaccacaggaaguuu...............	3	1	seq
......................................................................ucacacacaaccacaggaGguuu...............	5	1	seq
......................................................................ucacUcacaaccacaggaaguuu...............	3	1	seq
......................................................................ucacacacGaccacaggaaguuu...............	3	1	seq
......................................................................ucacacacaaccacaggGaguuu...............	3	1	seq
......................................................................ucacacacaaccaUaggaaguuu...............	4	1	seq
......................................................................ucacacacGaccacaggaaguuuu..............	3	1	seq
......................................................................ucacacacaaUcacaggaaguuuu..............	1	1	seq
......................................................................ucaUacacaaccacaggaaguuuu..............	1	1	seq
......................................................................ucUcacacaaccacaggaaguuuu..............	2	1	seq
......................................................................ucacacacaaccacaggaaUuuuu..............	1	1	seq
.......................................................................cacacacaaccacaggaagC.................	1	1	seq
```

Star **Mature**

```
gauagagaagagaggcuacuugcggaccuuucuguggcugucguguuaagugugauucuaugaagacacuucacacacaaccacaggaaguuuugcacgguagccaug
.......................................................................cGcacacaaccacaggaaguu................	1	1	seq
........................................................................acacacaaccacaggaag..................	1	0	seq
........................................................................acacacaaccacaggaaC..................	1	1	seq
........................................................................Ucacacaaccacaggaagu.................	3	1	seq
........................................................................acacacaaccacaggaagu.................	1	0	seq
........................................................................acacacaaccacaggaagC.................	1	1	seq
........................................................................Ucacacaaccacaggaaguu................	17	1	seq
........................................................................Ucacacaaccacaggaaguuu...............	1	1	seq
.........................................................................cacacaaccacaggaaguu................	1	0	seq
```
