# Supplementary material for: Identification and Characterization of MicroRNAs from Longitudinal Muscle and Respiratory Tree in Sea Cucumber (Apostichopus japonicus) Using High-Throughput Sequencing
Source: PLoS One. 2015 Aug 5;10(8):e0134899. doi: 10.1371/journal.pone.0134899 (PMC4526669; doi:10.1371/journal.pone.0134899)
Supplement: S2 File — (ZIP) [file pone.0134899.s003.zip › S2 File/The secondary structures of the novel miRNAs in RPT/Scaffold1117_2260.pdf]

Provisional ID	:	Scaffold1117_2260
Score total	:	61.5
Score for star read(s)	:	3.9
Score for read counts	:	59.8
Score for mfe	:	0
Score for randfold	:	-2.2
Score for cons. seed	:	
Total read count	:	129
Mature read count	:	121
Loop read count	:	0
Star read count	:	8

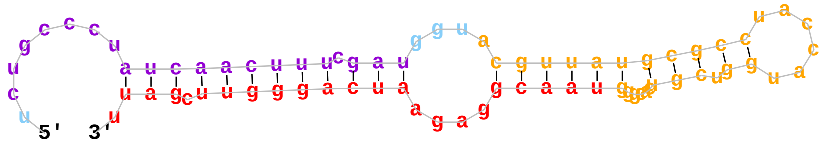

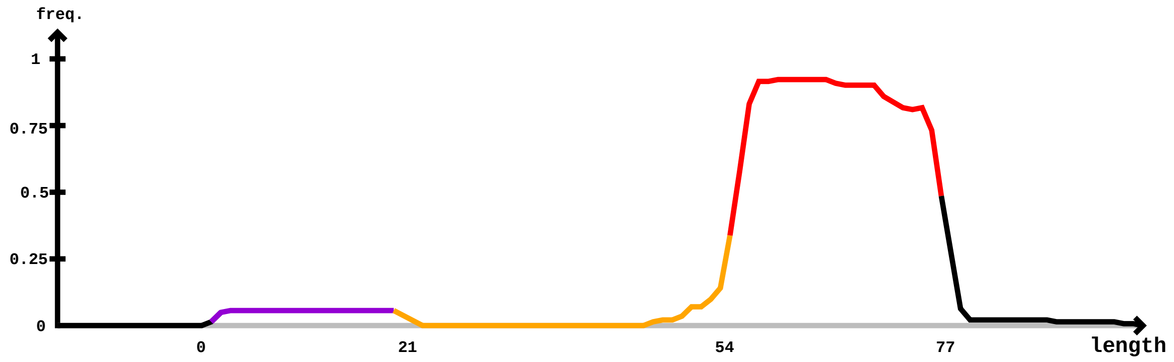

Star **Mature**

```
5'- ggauccuucgaaugucugcccuaucaacuuucgaugguacguuaugcgccuaccauggucgucacggguaacggagaaucaggguucgauuccggagagggagccugagaa   -3'   obs
    ggauccuucgaaugucugcccuaucaacuuucgaugguacguuaugcgccuaccauggucgucacggguaacggagaaucaggguucgauuccggagagggagccugagaa         exp
    ((.((((((....(((.(((((......(((((.((...(((.(((.(((......)))))).)))..)).)))))....)))))..)))......)))))).))......   reads   mm   sample
    ..............ucugcUcuaucaacuuucgau............................................................................   2   1   seq
    ...............cugcUcuaucaacuuucgau............................................................................   3   1   seq
    ...............cugcUcuaucaacuuucgaug...........................................................................   2   1   seq
    ................ugcUcuaucaacuuucgau............................................................................   1   1   seq
    ............................................................guGacggguaacggagaau................................   1   1   seq
    ............................................................guUacggguaacggagaau................................   1   1   seq
    .............................................................uGacggguaacggagaauc...............................   1   1   seq
    ...............................................................acggguaacggagaaucagg............................   1   0   seq
    ...............................................................acgUguaacggagaaucagggu..........................   1   1   seq
    ................................................................cggguaacggagaaucaggA...........................   1   1   seq
    ................................................................cggguaacggagaaucaggg...........................   3   0   seq
    ................................................................cggUuaacggagaaucaggguucga......................   1   1   seq
    ..................................................................gguaacggagaaucaggg...........................   2   0   seq
    ..................................................................gguaacggGgaaucagggu..........................   2   1   seq
    ...................................................................guaacggagaaucaggguu.........................   1   0   seq
    ...................................................................guaacggagaaucaggguucgau.....................   2   0   seq
    ...................................................................gGaacggagaaucaggguucgauu....................   1   1   seq
    ...................................................................guaacggagaaucaggguucgauC...................   2   1   seq
    ....................................................................uaacggagaaucaggguu.........................   2   0   seq
    ....................................................................uaacggagaaucaggguuc........................   1   0   seq
    ....................................................................uaacggagaaucaggguucgau.....................   3   0   seq
    ....................................................................uGacggagaaucaggguucgauu....................   1   1   seq
    ....................................................................uaUcggagaaucaggguucgauu....................   1   1   seq
    ....................................................................uaacggagaaucaggguucgauu....................   20   0   seq
    .....................................................................aacUgagaaucaggguucga......................   1   1   seq
    .....................................................................aacggagaaucaggguucga......................   8   0   seq
    .....................................................................aacggagaaucaggguucgau.....................   12   0   seq
    .....................................................................aacggagaaucagggCucgau.....................   1   1   seq
    .....................................................................aacggagaGucaggguucgau.....................   1   1   seq
    .....................................................................aacUgagaaucaggguucgauu....................   1   1   seq
    .....................................................................aacggagaaucaggguucgauu....................   10   0   seq
    ......................................................................acggagaaucaggguucga......................   2   0   seq
    ......................................................................acggagaaucaggguucgau.....................   8   0   seq
```

```
                    Star                                              Mature
ggauccuucgaaugucugcccuaucaacuuucgaugguacguuaugcgccuaccauggucgucacggguaacggagaaucaggguucgauuccggagagggagccugagaa
......................................................................acgUagaaucaggguucgau.....................    2    1    seq
......................................................................acggagaaucaggguucgauC....................    2    1    seq
......................................................................acgUagaaucaggguucgauu....................    2    1    seq
......................................................................acggagaaucaggguucgauu....................    14   0    seq
......................................................................acgUagaaucaggguucgauucc..................    2    1    seq
......................................................................acggagaaucaggguucgauucc..................    4    0    seq
.......................................................................cggagaaucaggguucgau.....................    5    0    seq
.......................................................................cggagaaucaggguuUgau.....................    1    1    seq
.......................................................................cgUagaaucaggguucgau.....................    1    1    seq
.......................................................................cggagaaucaggguucgauu....................    5    0    seq
.........................................................................gagaaucaggguucgauu....................    1    0    seq
...................................................................................guucgauuccggagaggga.........    1    0    seq
........................................................................................auuccggagaUggagccugagaa    1    1    seq
..........................................................................................uccggagagggagccugag..    1    0    seq
```
