# Supplementary material for: Identification and Characterization of MicroRNAs from Longitudinal Muscle and Respiratory Tree in Sea Cucumber (Apostichopus japonicus) Using High-Throughput Sequencing
Source: PLoS One. 2015 Aug 5;10(8):e0134899. doi: 10.1371/journal.pone.0134899 (PMC4526669; doi:10.1371/journal.pone.0134899)
Supplement: S2 File — (ZIP) [file pone.0134899.s003.zip › S2 File/The secondary structures of the novel miRNAs in RPT/Scaffold111_460.pdf]

Provisional ID	:	Scaffold111_460
Score total	:	2.1
Score for star read(s)	:	-1.3
Score for read counts	:	0
Score for mfe	:	1.8
Score for randfold	:	1.6
Score for cons. seed	:	
Total read count	:	10733
Mature read count	:	10733
Loop read count	:	0
Star read count	:	0

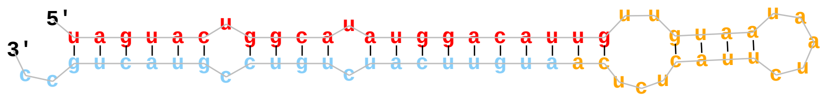

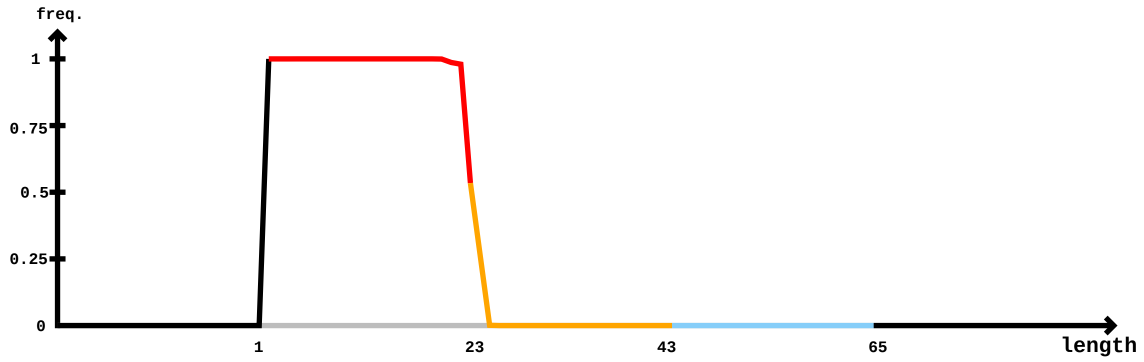


```
                         Mature                                     Star
5'- acacauugcuggguucuugauaguacuggcauauggacauuguuguaauaaucuuacucucaauguucaucuguccguacugccaagugccuggccuuacauucauuc   -3'   exp
    .......(((((((.((((.((((((.((((.((((((((((..((((.....))))...))))))))))).)))).)))))).)))).)))))))...............   reads      mm         sample
    ....................uUguacuggcauauggac......................................................................   1          1          seq
    ....................uaguacuggcauauggac......................................................................   4          0          seq
    ....................uaguacuggcauauggaU......................................................................   1          1          seq
    ....................uaguacuggcauauggacG.....................................................................   1          1          seq
    ....................uaguacuggcauauggaca.....................................................................   123        0          seq
    ....................Gaguacuggcauauggaca.....................................................................   1          1          seq
    ....................uaguacuggUauauggaca.....................................................................   1          1          seq
    ....................uagGacuggcauauggaca.....................................................................   5          1          seq
    ....................uaguacuggcauauggGca.....................................................................   1          1          seq
    ....................uaguacuggcauauggacU.....................................................................   3          1          seq
    ....................uaUuacuggcauauggaca.....................................................................   4          1          seq
    ....................uaguacuggcauauggacaG....................................................................   3          1          seq
    ....................uUguacuggcauauggacau....................................................................   5          1          seq
    ....................uaCuacuggcauauggacau....................................................................   1          1          seq
    ....................uaguacGggcauauggacau....................................................................   1          1          seq
    ....................uaguacuggUauauggacau....................................................................   1          1          seq
    ....................uaguacuggcauauUgacau....................................................................   1          1          seq
    ....................uaguaUuggcauauggacau....................................................................   1          1          seq
    ....................uaguacuggcauauggGcau....................................................................   1          1          seq
    ....................uaguacuggcauauggacaA....................................................................   1          1          seq
    ....................uaguacuggcauauAgacau....................................................................   1          1          seq
    ....................uaguGcuggcauauggacau....................................................................   2          1          seq
    ....................Gaguacuggcauauggacau....................................................................   1          1          seq
    ....................uaguacuggcGuauggacau....................................................................   1          1          seq
    ....................uGguacuggcauauggacau....................................................................   5          1          seq
    ....................uaguacuggcauauggacaC....................................................................   13         1          seq
    ....................uaUuacuggcauauggacau....................................................................   7          1          seq
    ....................uaguacuggcauauggacGu....................................................................   3          1          seq
    ....................uagCacuggcauauggacau....................................................................   1          1          seq
    ....................Caguacuggcauauggacau....................................................................   1          1          seq
    ....................uaguacuggcauauggacUu....................................................................   1          1          seq
    ....................uagGacuggcauauggacau....................................................................   19         1          seq
    ....................uaguacuggcauauggacaAu...................................................................   13         1          seq
    ....................uaguacuggcauauggacGuu...................................................................   173        1          seq
```

```
                   Mature                                        Star
acacauugcuggguucuugauaguacuggcauauggacauuguuguaauaaucuuacucucaauguucaucuguccguacugccaagugccuggccuuacauucauuc
....................uaguacuUgcauauggacauu...................................................................   35     1     seq
....................uaguacuggcauauCgacauu...................................................................   1      1     seq
....................uaguaAuggcauauggacauu...................................................................   7      1     seq
....................uaguacugAcauauggacauu...................................................................   53     1     seq
....................uaguacuggcaCauggacauu...................................................................   82     1     seq
....................uaguacuggcauaGggacauu...................................................................   14     1     seq
....................uaguacuggcauauAgacauu...................................................................   78     1     seq
....................uaguacuggcauauggacaCu...................................................................   68     1     seq
....................uagGacuggcauauggacauu...................................................................   1410   1     seq
....................uaguacuggcauauUgacauu...................................................................   49     1     seq
....................uaguacuggcGuauggacauu...................................................................   159    1     seq
....................uaAuacuggcauauggacauu...................................................................   45     1     seq
....................uaguacuAgcauauggacauu...................................................................   46     1     seq
....................uaCuacuggcauauggacauu...................................................................   42     1     seq
....................uaguGcuggcauauggacauu...................................................................   101    1     seq
....................uaguacuggAauauggacauu...................................................................   13     1     seq
....................uaguacuggcauauggaUauu...................................................................   36     1     seq
....................uaguUcuggcauauggacauu...................................................................   5      1     seq
....................uaguacuggcauauggUcauu...................................................................   14     1     seq
....................uaguacGggcauauggacauu...................................................................   54     1     seq
....................uaguacuggcaAauggacauu...................................................................   5      1     seq
....................uUguacuggcauauggacauu...................................................................   342    1     seq
....................uaguacuggcUuauggacauu...................................................................   4      1     seq
....................uagAacuggcauauggacauu...................................................................   25     1     seq
....................uaguacuCgcauauggacauu...................................................................   4      1     seq
....................uaguacuggcauaugCacauu...................................................................   8      1     seq
....................uaguacuggGauauggacauu...................................................................   13     1     seq
....................uaguaUuggcauauggacauu...................................................................   44     1     seq
....................uaguacuggcauaAggacauu...................................................................   22     1     seq
....................uaUuacuggcauauggacauu...................................................................   585    1     seq
....................uaguCcuggcauauggacauu...................................................................   22     1     seq
....................uagCacuggcauauggacauu...................................................................   122    1     seq
....................uaguacuggcauauggaAauu...................................................................   3      1     seq
....................uaguacuggcauaugAacauu...................................................................   31     1     seq
....................uaguacuggcauGuggacauu...................................................................   84     1     seq
....................uaguacuggcauauggacaGu...................................................................   34     1     seq
....................uaguacuggcauauggCcauu...................................................................   9      1     seq
....................uaguacAggcauauggacauu...................................................................   32     1     seq
....................uaguacuggcauauggGcauu...................................................................   239    1     seq
....................uGguacuggcauauggacauu...................................................................   236    1     seq
....................uaguacuggcauCuggacauu...................................................................   6      1     seq
....................uaguacCggcauauggacauu...................................................................   135    1     seq
....................uaguacugUcauauggacauu...................................................................   16     1     seq
....................uaguacuggcauauggacCuu...................................................................   1      1     seq
....................uaguacugCcauauggacauu...................................................................   7      1     seq
....................uaguacuggUauauggacauu...................................................................   55     1     seq
....................uaguacuggcCuauggacauu...................................................................   11     1     seq
....................uaguacuggcauUuggacauu...................................................................   5      1     seq
....................uaguacuggcaGauggacauu...................................................................   28     1     seq
....................uaguacuggcauauggacUuu...................................................................   11     1     seq
....................uCguacuggcauauggacauu...................................................................   4      1     seq
....................uaguacuggcauaCggacauu...................................................................   113    1     seq
....................uaguacuggcauauggaGauu...................................................................   5      1     seq
....................uaguaGuggcauauggacauu...................................................................   5      1     seq
....................uaguacuggcauaugUacauu...................................................................   19     1     seq
....................uaguacuggcauaugCacauug..................................................................   4      1     seq
....................uaguacuggcauaugUacauug..................................................................   26     1     seq
....................uaguGcuggcauauggacauug..................................................................   128    1     seq
....................uaguacuggcauauAgacauug..................................................................   99     1     seq
....................uaguacAggcauauggacauug..................................................................   45     1     seq
....................uaguacuggcauauggaUauug..................................................................   46     1     seq
....................uaguaUuggcauauggacauug..................................................................   62     1     seq
....................uaguacuggcauauggacUuug..................................................................   9      1     seq
....................uaguacuggGauauggacauug..................................................................   17     1     seq
....................uaguaAuggcauauggacauug..................................................................   4      1     seq
....................uaguacuggcauUuggacauug..................................................................   8      1     seq
....................uaguacugUcauauggacauug..................................................................   33     1     seq
....................uaguacuggcUuauggacauug..................................................................   13     1     seq
....................uaguacuggcaCauggacauug..................................................................   103    1     seq
....................uaguacuggcauauggacGuug..................................................................   167    1     seq
```

Mature **Star**

acacauugcuggguucuugauaguacuggcauauggacauuguuguaauaaucuuacucucaauguucaucuguccguacugccaagugccuggccuuacauucauuc			
....................uaguacuggcauauggacCuug..	7	1	seq
....................uaguacuggcauaugAacauug..	55	1	seq
....................uaCuacuggcauauggacauug..	51	1	seq
....................uaguacuggAauauggacauug..	10	1	seq
....................uaguacuggcGuauggacauug..	211	1	seq
....................uaguacuggcauauUgacauug..	70	1	seq
....................uaguacuggUauauggacauug..	91	1	seq
....................uaguacuggcauaGggacauug..	15	1	seq
....................uagAacuggcauauggacauug..	29	1	seq
....................uagCacuggcauauggacauug..	142	1	seq
....................uaguacuggcauauggacaAug..	12	1	seq
....................uaguacuggcauauggGcauug..	272	1	seq
....................uaguacuUgcauauggacauug..	50	1	seq
....................uaguacuggcauauggacaCug..	83	1	seq
....................uaguaGuggcauauggacauug..	9	1	seq
....................uaguacuAgcauauggacauug..	57	1	seq
....................uaguacuggcauauggacaGug..	32	1	seq
....................uaguacuggcauaCggacauug..	176	1	seq
....................uaguacuggcauauggUcauug..	22	1	seq
....................uaguacuggcauauggCcauug..	11	1	seq
....................uaguacuggcaAauggacauug..	5	1	seq
....................uaguacuggcauCuggacauug..	6	1	seq
....................uaguUcuggcauauggacauug..	15	1	seq
....................uaguacugAcauauggacauug..	67	1	seq
....................uagGacuggcauauggacauug..	1716	1	seq
....................uaguacCggcauauggacauug..	148	1	seq
....................uaguCcuggcauauggacauug..	28	1	seq
....................uaguacuggcauGuggacauug..	112	1	seq
....................uaAuacuggcauauggacauug..	55	1	seq
....................uaguacuggcCuauggacauug..	5	1	seq
....................uaguacuggcauauggaAauug..	3	1	seq
....................uaguacuggcaGauggacauug..	37	1	seq
....................uaguacuggcauauCgacauug..	7	1	seq
....................uaguacuggcauaAggacauug..	22	1	seq
....................uaguacGggcauauggacauug..	85	1	seq
....................uaUuacuggcauauggacauug..	826	1	seq
....................uaguacuCgcauauggacauug..	2	1	seq
....................uaguacugCcauauggacauug..	3	1	seq
....................uaguacuggcauauggaGauug..	3	1	seq
....................uaguacuggcaAauggacauugu...	1	1	seq
....................uaguacuggcauauggacaCugu...	3	1	seq
....................uaguacGggcauauggacauugu...	4	1	seq
....................uaguacuggcauauggacGuugu...	17	1	seq
....................uaguacuggcauaAggacauugu...	1	1	seq
....................uaUuacuggcauauggacauugu...	48	1	seq
....................uaguacuggcauauUgacauugu...	5	1	seq
....................uaguacCggcauauggacauugu...	11	1	seq
....................uaguacuggcauauggacaGugu...	5	1	seq
....................uaguacuggcauGuggacauugu...	8	1	seq
....................uaguacuggcauauggUcauugu...	1	1	seq
....................uaguacugAcauauggacauugu...	8	1	seq
....................uaguaUuggcauauggacauugu...	2	1	seq
....................uagAacuggcauauggacauugu...	3	1	seq
....................uaguacuggcauauggaUauugu...	1	1	seq
....................uaCuacuggcauauggacauugu...	4	1	seq
....................uaguacuggcauauAgacauugu...	11	1	seq
....................uaguacuggcCuauggacauugu...	1	1	seq
....................uaguacugUcauauggacauugu...	2	1	seq
....................uaguacuggAauauggacauugu...	1	1	seq
....................uagGacuggcauauggacauugu...	140	1	seq
....................uaguacuggcauUuggacauugu...	1	1	seq
....................uaguacuggcauauggaGauugu...	1	1	seq
....................uaguacuUgcauauggacauugu...	5	1	seq
....................uaguacuggcauaCggacauugu...	10	1	seq
....................uaguacuggcGuauggacauugu...	11	1	seq
....................uaguacuggcaGauggacauugu...	3	1	seq
....................uagCacuggcauauggacauugu...	18	1	seq
....................uaguacuggcauaugAacauugu...	6	1	seq
....................uaguGcuggcauauggacauugu...	12	1	seq
....................uaguacuggUauauggacauugu...	6	1	seq

Mature Star

```
acacauugcuggguucuugauaguacuggcauauggacauuguuguaauaaucuuacucucaauguucaucuguccguacugccaagugccuggccuuacauucauuc
....................uaguacAggcauauggacauugu.................................................................	3	1	seq
....................uaguacuggcauaGggacauugu.................................................................	2	1	seq
....................uaguacuggcauauCgacauugu.................................................................	1	1	seq
....................uaguacuAgcauauggacauugu.................................................................	4	1	seq
....................uaguacuggcaCauggacauugu.................................................................	9	1	seq
....................uaguacuggcauaugUacauugu.................................................................	3	1	seq
....................uaAuacuggcauauggacauugu.................................................................	5	1	seq
....................uaguacuggcauauggGcauugu.................................................................	27	1	seq
....................uaguCcuggcauauggacauugu.................................................................	1	1	seq
....................uaguacuggcauauggacUuugu.................................................................	3	1	seq
....................uaguacuggcauauAgacauuguu................................................................	1	1	seq
....................uaguacuggcaGauggacauuguu................................................................	1	1	seq
....................uaguacugAcauauggacauuguu................................................................	1	1	seq
....................uaguacuggcGuauggacauuguu................................................................	1	1	seq
....................uagGacuggcauauggacauuguu................................................................	4	1	seq
....................uaguacuggcauauggacGuuguu................................................................	1	1	seq
....................uaguacuggcauauUgacauuguu................................................................	1	1	seq
....................uaguacuggcauauggGcauuguu................................................................	1	1	seq
....................uaUuacuggcauauggacauuguu................................................................	1	1	seq
.......................uacuggcauauggacauug..................................................................	1	0	seq
```
