# Supplementary material for: Identification and Characterization of MicroRNAs from Longitudinal Muscle and Respiratory Tree in Sea Cucumber (Apostichopus japonicus) Using High-Throughput Sequencing
Source: PLoS One. 2015 Aug 5;10(8):e0134899. doi: 10.1371/journal.pone.0134899 (PMC4526669; doi:10.1371/journal.pone.0134899)
Supplement: S2 File — (ZIP) [file pone.0134899.s003.zip › S2 File/The secondary structures of the novel miRNAs in RPT/Scaffold1239_2353.pdf]

| | | |
|---|---|---|
| Provisional ID | : | Scaffold1239_2353 |
| Score total | : | 3.1 |
| Score for star read(s) | : | -1.3 |
| Score for read counts | : | 0 |
| Score for mfe | : | 2.8 |
| Score for randfold | : | 1.6 |
| Score for cons. seed | : | |
| Total read count | : | 22 |
| Mature read count | : | 22 |
| Loop read count | : | 0 |
| Star read count | : | 0 |

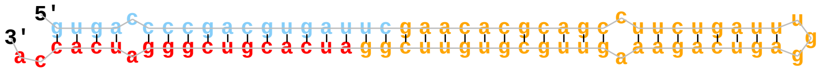


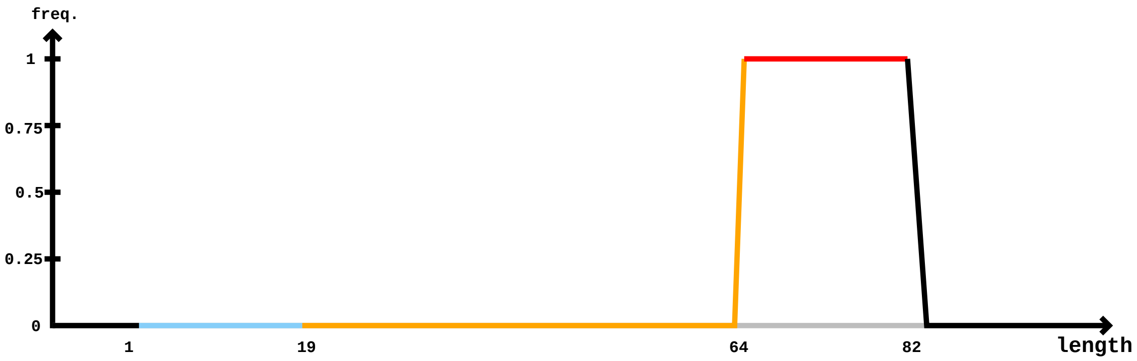


Star Mature

```
5'- ugcuauggugaccccgacgugauucgaacacgcagccuucugauuuggagucagaaaguugcguguucggaucacgucgggaucaccagguaaugugaccaaucacag   -3'    exp
    ((((.((((((.((((((((((((((((((((((((.((((((((...)))))))).))))))))))))))))))))))))).)))))))))).(((((....))))).     reads      mm         sample
    ...............................................................aucacgucgggaucacca...........................     11         0          seq
    ...............................................................aucacgucgggGucacca...........................     10         1          seq
    ...............................................................aGcacgucgggaucacca...........................     1          1          seq
```
