# Supplementary material for: Identification and Characterization of MicroRNAs from Longitudinal Muscle and Respiratory Tree in Sea Cucumber (Apostichopus japonicus) Using High-Throughput Sequencing
Source: PLoS One. 2015 Aug 5;10(8):e0134899. doi: 10.1371/journal.pone.0134899 (PMC4526669; doi:10.1371/journal.pone.0134899)
Supplement: S2 File — (ZIP) [file pone.0134899.s003.zip › S2 File/The secondary structures of the novel miRNAs in RPT/Scaffold1492_2593.pdf]

Provisional ID	:	Scaffold1492_2593
Score total	:	248.9
Score for star read(s)	:	3.9
Score for read counts	:	241.3
Score for mfe	:	2.1
Score for randfold	:	1.6
Score for cons. seed	:	
Total read count	:	485
Mature read count	:	484
Loop read count	:	0
Star read count	:	1

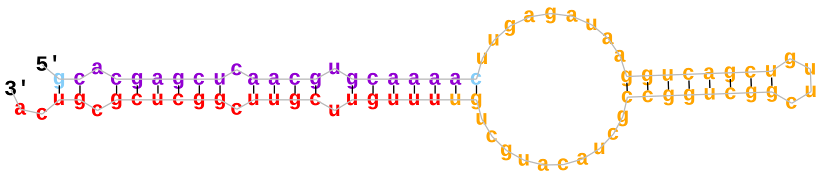

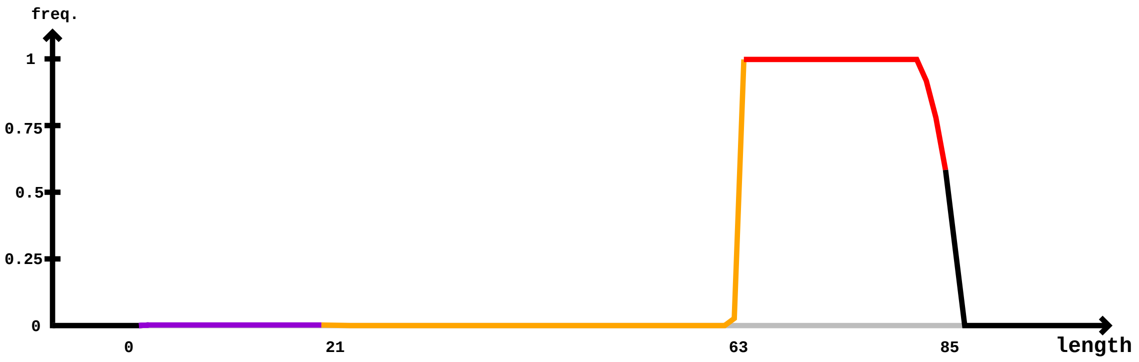

Star **Mature**

```
5'- caguuuggcacgagcucaacgugcaaaacuugagauaaggucagcuguucggcuggccgcuacaugcuguuuuguucguucggcucgcgucaaauugcagucacacua   -3'   obs
    caguuuggcacgagcucaacgugcaaaacuugagauaaggucagcuguucggcuggccgcuacaugcuguuuuguucguucggcucgcgucaaauugcagucacacua         exp
    (((((((((.((((((.((((.(((((((.........((((((((....))))))))..........))))))).)))).)))))).))))))))).........   reads   mm   sample
    ........Aacgagcucaacgugcaaaa................................................................................   1   1   seq
    .....................................................................uuuGguucguucggcucgcg...................   1   1   seq
    .....................................................................uuuuguucguucggcucgcA...................   3   1   seq
    .....................................................................Guuuguucguucggcucgcg...................   1   1   seq
    .....................................................................uuuuguucguucggcucgcC...................   1   1   seq
    .....................................................................uCuuguucguucggcucgcg...................   2   1   seq
    .....................................................................uuuuguucguucggcAcgcg...................   1   1   seq
    .....................................................................uuuuguucguucggcucAcgu..................   1   1   seq
    .....................................................................uuuGguucguucggcucgcgu..................   1   1   seq
    .....................................................................uuGuguucguucggcucgcguc.................   1   1   seq
    .....................................................................uuuuguucguucggcucgcUuca................   1   1   seq
    ......................................................................uuuUuucguucggcucgcg...................   6   1   seq
    ......................................................................uuuguucguucggcucgcg...................   23   0   seq
    ......................................................................uuuguucguucggcucgcA...................   1   1   seq
    ......................................................................uuugCucguucggcucgcgu..................   1   1   seq
    ......................................................................uuuguucguucggcCcgcgu..................   1   1   seq
    ......................................................................uuuguucguuAggcucgcgu..................   2   1   seq
    ......................................................................uCuguucguucggcucgcgu..................   4   1   seq
    ......................................................................uuuguucgAucggcucgcgu..................   2   1   seq
    ......................................................................Cuuguucguucggcucgcgu..................   1   1   seq
    ......................................................................uuGguucguucggcucgcgu..................   1   1   seq
    ......................................................................uuuguucguucggcucgcgG..................   3   1   seq
    ......................................................................uuuguucguucggcuUgcgu..................   1   1   seq
    ......................................................................uuCguucguucggcucgcgu..................   1   1   seq
    ......................................................................uGuguucguucggcucgcgu..................   5   1   seq
    ......................................................................uuuUuucguucggcucgcgu..................   35   1   seq
    ......................................................................Auuguucguucggcucgcgu..................   1   1   seq
    ......................................................................uuuguucguucggcucAcgu..................   3   1   seq
    ......................................................................uuuguucguucggcucgcgA..................   4   1   seq
    ......................................................................uGuguucguucggcucgcguc.................   12   1   seq
    ......................................................................uuuguucguucggcAcgcguc.................   1   1   seq
    ......................................................................uCuguucguucggcucgcguc.................   3   1   seq
    ......................................................................uuGguucguucggcucgcguc.................   1   1   seq
```

Star **Mature**

```
caguuuggcacgagcucaacgugcaaaacuugagauaaggucagcuguucggcuggccgcuacaugcuguuuuguucguucggcucgcgucaaauugcagucacacua
......................................................................uuuguucguucggcucgUguc.................	2	1	seq
......................................................................uuuguucguucggcucgcgGc.................	2	1	seq
......................................................................uAuguucguucggcucgcguc.................	2	1	seq
......................................................................uuugCucguucggcucgcguc.................	1	1	seq
......................................................................uuuguucguucggGucgcguc.................	1	1	seq
......................................................................uuuguucgAucggcucgcguc.................	1	1	seq
......................................................................uuuguucguucggcCcgcguc.................	2	1	seq
......................................................................uuuguucgGucggcucgcguc.................	1	1	seq
......................................................................uuCguucguucggcucgcguc.................	2	1	seq
......................................................................uuuguucguuUggcucgcguc.................	1	1	seq
......................................................................uuuCuucguucggcucgcguc.................	1	1	seq
......................................................................uuuguCcguucggcucgcguc.................	3	1	seq
......................................................................uuuUuucguucggcucgcguc.................	57	1	seq
......................................................................uuuguucguuAggcucgcguc.................	1	1	seq
......................................................................uuuguucguucgUcucgcguca................	2	1	seq
......................................................................uuuguucguucAgcucgcguca................	4	1	seq
......................................................................uuuguucguucggcucgcgAca................	1	1	seq
......................................................................uuuguucguucggcucAcguca................	2	1	seq
......................................................................uuuguucgCucggcucgcguca................	7	1	seq
......................................................................uuuAuucguucggcucgcguca................	2	1	seq
......................................................................uuuguucguucggGucgcguca................	1	1	seq
......................................................................uuuguuUguucggcucgcguca................	1	1	seq
......................................................................uuuguucgAucggcucgcguca................	1	1	seq
......................................................................uuuguucguAcggcucgcguca................	1	1	seq
......................................................................uuugGucguucggcucgcguca................	5	1	seq
......................................................................uuuguucguuUggcucgcguca................	6	1	seq
......................................................................uuugAucguucggcucgcguca................	2	1	seq
......................................................................uuuguucguucggcuGgcguca................	1	1	seq
......................................................................uuuguucUuucggcucgcguca................	1	1	seq
......................................................................uuAguucguucggcucgcguca................	3	1	seq
......................................................................uuuguucguucggcAcgcguca................	2	1	seq
......................................................................uuuUuucguucggcucgcguca................	167	1	seq
......................................................................uuuguucguucggcGcgcguca................	3	1	seq
......................................................................uuuguucguucggcucgcgGca................	6	1	seq
......................................................................uuuguucguucggcuUgcguca................	2	1	seq
......................................................................uuuguucguucggcuAgcguca................	1	1	seq
......................................................................uuuguCcguucggcucgcguca................	7	1	seq
......................................................................uuuguucguucggcCcgcguca................	4	1	seq
......................................................................uuCguucguucggcucgcguca................	10	1	seq
......................................................................uuuguucguGcggcucgcguca................	2	1	seq
......................................................................uuuguucguucUgcucgcguca................	2	1	seq
......................................................................uuuguucguucgAcucgcguca................	3	1	seq
......................................................................uuugCucguucggcucgcguca................	11	1	seq
......................................................................uuuCuucguucggcucgcguca................	2	1	seq
......................................................................uuuguucAuucggcucgcguca................	4	1	seq
......................................................................uuuguucgGucggcucgcguca................	2	1	seq
......................................................................uuuguucguucggUucgcguca................	2	1	seq
......................................................................uuuguucguucggcucgUguca................	6	1	seq
......................................................................uuGguucguucggcucgcguca................	5	1	seq
......................................................................uuuguucguucggcucgcUuca................	1	1	seq
```
