# Supplementary material for: Identification and Characterization of MicroRNAs from Longitudinal Muscle and Respiratory Tree in Sea Cucumber (Apostichopus japonicus) Using High-Throughput Sequencing
Source: PLoS One. 2015 Aug 5;10(8):e0134899. doi: 10.1371/journal.pone.0134899 (PMC4526669; doi:10.1371/journal.pone.0134899)
Supplement: S2 File — (ZIP) [file pone.0134899.s003.zip › S2 File/The secondary structures of the novel miRNAs in RPT/Scaffold191_722.pdf]

Provisional ID	:	Scaffold191_722
Score total	:	2.1
Score for star read(s)	:	-1.3
Score for read counts	:	0
Score for mfe	:	1.8
Score for randfold	:	1.6
Score for cons. seed	:	
Total read count	:	3737
Mature read count	:	3737
Loop read count	:	0
Star read count	:	0

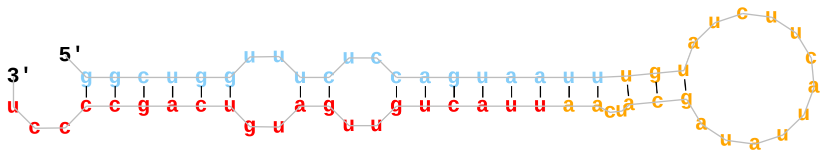

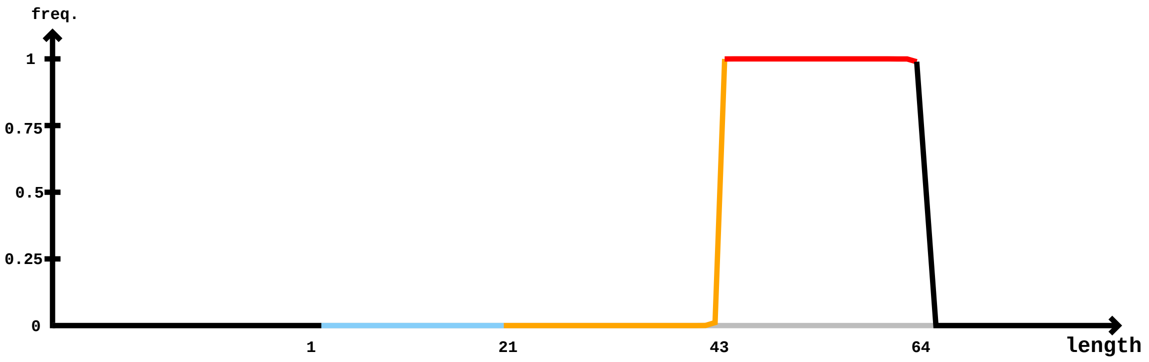

Star Mature

```
5'- ccaugcucucaugguacgccagcaagggcugguuucuccaguaauuuguaucuucauuauagcaucaauuacuguugaugucagccccuugcugguauacugcccucua   -3'    exp
    (((((....)))))...(((((((((((((((..((..(((((((((((............)))..))))))))..))..)))).))))))))))).............    reads      mm          sample
    ..................................................................aauuacuguugaugucagcc.......................    1          0           seq
    ...................................................................auuacuguugaugucagcccU.....................    3          1           seq
    ...................................................................auuacuguugaugucagcccUu....................    23         1           seq
    ...................................................................auuacuguugaugucagcccUuu...................    15         1           seq
    ....................................................................uuacuguugaugucagcccU.....................    35         1           seq
    ....................................................................uuacuguugaugucagcccA.....................    1          1           seq
    ....................................................................uCacuguugaugucagccccu....................    1          1           seq
    ....................................................................uuacuguugaugucagcccUu....................    2540       1           seq
    ....................................................................uuacuguugaugucagcccAu....................    1          1           seq
    ....................................................................uuaUuguugaugucagccccu....................    1          1           seq
    ....................................................................uuacuguCgaugucagccccu....................    1          1           seq
    ....................................................................uuacuguugaugucagcccGu....................    3          1           seq
    ....................................................................uuacuguugaugucagcccGuu...................    1          1           seq
    ....................................................................uuacuguugaugucagcccUuu...................    1110       1           seq
    .....................................................................uacuguugaugucagcccUuu...................    1          1           seq
```
