# Supplementary material for: Identification and Characterization of MicroRNAs from Longitudinal Muscle and Respiratory Tree in Sea Cucumber (Apostichopus japonicus) Using High-Throughput Sequencing
Source: PLoS One. 2015 Aug 5;10(8):e0134899. doi: 10.1371/journal.pone.0134899 (PMC4526669; doi:10.1371/journal.pone.0134899)
Supplement: S2 File — (ZIP) [file pone.0134899.s003.zip › S2 File/The secondary structures of the novel miRNAs in RPT/Scaffold198_750.pdf]

Provisional ID	:	Scaffold198_750
Score total	:	351.4
Score for star read(s)	:	3.9
Score for read counts	:	344.8
Score for mfe	:	1.1
Score for randfold	:	1.6
Score for cons. seed	:	
Total read count	:	688
Mature read count	:	567
Loop read count	:	0
Star read count	:	121

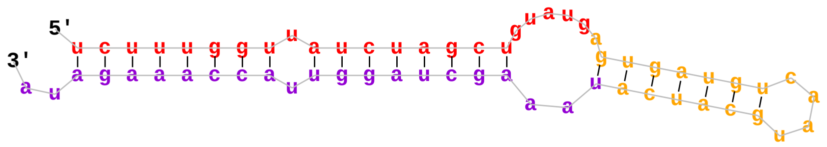

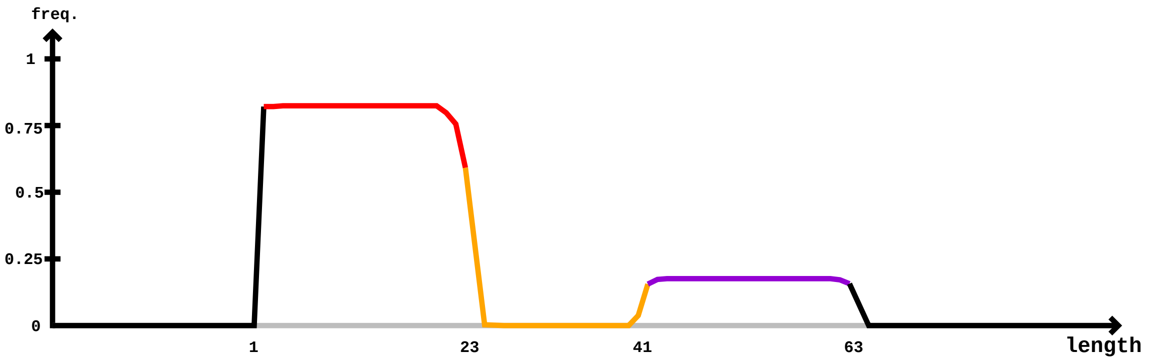

Mature **Star**

5'- ucauacugacucugcuuuucucuuugguuaucuagcuguaugagugaugucaaugcaucauaaagcuagguuaccaaagauaagugccuguugucacaugucgccccug -3'	obs		
ucauacugacucugcuuuucucuuugguuaucuagcuguaugagugaugucaaugcaucauaaagcuagguuaccaaagauaagugccuguugucacaugucgccccug	exp		
.(((..((((.(.((.(((.((((((((.((((((((......(((((((....)))))))..)))))))).)))))))).))).))..)..)))).))).........	reads	mm	sample
....................ucuGugguuaucuagcugu..	1	1	seq
....................ucuuugguuaucuagcugu..	16	0	seq
....................ucuuugguuaucuGgcugu..	1	1	seq
....................ucuuuggCuaucuagcugua...	1	1	seq
....................ucuuugAuuaucuagcugua...	1	1	seq
....................ucCuugguuaucuagcugua...	1	1	seq
....................Gcuuugguuaucuagcugua...	4	1	seq
....................ucuuugguuaucuagUugua...	1	1	seq
....................ucuuugguCaucuagcugua...	1	1	seq
....................ucuuugguuaucuCgcugua...	2	1	seq
....................ucuGugguuaucuagcugua...	2	1	seq
....................ucuuugguuaucuagcugCa...	1	1	seq
....................Ccuuugguuaucuagcugua...	1	1	seq
....................ucuugUuuaucuagcugua...	2	1	seq
....................ucAuugguuaucuagcugua...	1	1	seq
....................ucuuCgguuaucuagcugua...	1	1	seq
....................uUuuugguuaucuagcugua...	1	1	seq
....................ucuuugguuaucuGgcugua...	1	1	seq
....................ucuuugguuaucCagcugua...	1	1	seq
....................ucGuugguuaucuagcugua...	4	1	seq
....................ucuuugguuaucuagcuguC...	1	1	seq
....................ucuuugguuaucuagcuguU...	2	1	seq
....................ucuuugguuaucuaAcuguau..	1	1	seq
....................ucuuugguuaucuagcugCau..	4	1	seq
....................ucuAugguuaucuagcuguau..	1	1	seq
....................ucuGugguuaucuagcuguau..	21	1	seq
....................ucuuugguuaAcuagcuguau..	1	1	seq
....................ucuuugUuuaucuagcuguau..	3	1	seq
....................ucuuuUguuaucuagcuguau..	1	1	seq
....................uUuuugguuaucuagcuguau..	6	1	seq
....................ucuCugguuaucuagcuguau..	6	1	seq
....................ucuuugguuaucuagcGguau..	2	1	seq
....................ucCuugguuaucuagcuguau..	10	1	seq

```
                    Mature                              Star
ucauacugacucugcuuuucucuuugguuaucuagcuguaugagugaugucaaugcaucauaaagcuagguuaccaaagauaagugccuguugucacaugucgccccug
....................ucuuuggCuaucuagcuguau....................................................................  2   1   seq
....................ucuuugguCaucuagcuguau....................................................................  3   1   seq
....................ucuuugguuaucuagUuguau....................................................................  1   1   seq
....................ucuuugguuaucCagcuguau....................................................................  6   1   seq
....................ucuuugguuaucuagcugGau....................................................................  1   1   seq
....................ucuuCgguuaucuagcuguau....................................................................  4   1   seq
....................ucuuugguuaucuCgcuguau....................................................................  1   1   seq
....................ucuuugguuaucuagcAguau....................................................................  2   1   seq
....................uGuuugguuaucuagcuguau....................................................................  5   1   seq
....................ucuuugguuaGcuagcuguau....................................................................  1   1   seq
....................ucuuuggGuaucuagcuguau....................................................................  3   1   seq
....................ucuuugguuaucuagAuguau....................................................................  2   1   seq
....................ucuuugguuaucuagcCguau....................................................................  3   1   seq
....................ucuuugAuuaucuagcuguau....................................................................  2   1   seq
....................ucuuugguuGucuagcuguau....................................................................  5   1   seq
....................ucAuugguuaucuagcuguau....................................................................  4   1   seq
....................ucuuugguuaucuGgcuguau....................................................................  5   1   seq
....................ucGuugguuaucuagcuguau....................................................................  5   1   seq
....................ucuuugguuauUuagcuguau....................................................................  1   1   seq
....................ucuuugguuaucuaCcuguaug...................................................................  2   1   seq
....................ucuuugguuaucuagcugCaug...................................................................  3   1   seq
....................ucCuugguuaucuagcuguaug...................................................................  18  1   seq
....................ucGuugguuaucuagcuguaug...................................................................  9   1   seq
....................ucuuugguuaucuagcuguCug...................................................................  1   1   seq
....................ucuuugguGaucuagcuguaug...................................................................  2   1   seq
....................ucuuugguAaucuagcuguaug...................................................................  1   1   seq
....................ucuuuggAuaucuagcuguaug...................................................................  1   1   seq
....................ucuuCgguuaucuagcuguaug...................................................................  8   1   seq
....................ucuuugguuaucuUgcuguaug...................................................................  2   1   seq
....................ucuuugguuaGcuagcuguaug...................................................................  1   1   seq
....................ucuCugguuaucuagcuguaug...................................................................  8   1   seq
....................ucuuugAuuaucuagcuguaug...................................................................  2   1   seq
....................ucuuugguuaucuagcGguaug...................................................................  1   1   seq
....................ucuuuggGuaucuagcuguaug...................................................................  4   1   seq
....................ucuuugguuaucuagcuAuaug...................................................................  3   1   seq
....................ucuuGgguuaucuagcuguaug...................................................................  1   1   seq
....................ucuuugguuaucuagcuUuaug...................................................................  2   1   seq
....................ucuuugguuaucCagcuguaug...................................................................  8   1   seq
....................ucuGugguuaucuagcuguaug...................................................................  32  1   seq
....................ucuuuAguuaucuagcuguaug...................................................................  4   1   seq
....................ucuuugguuaucuagcugAaug...................................................................  1   1   seq
....................ucuuugguuaucuaAcuguaug...................................................................  1   1   seq
....................ucuuugguuaucuagUuguaug...................................................................  6   1   seq
....................ucuuugguuaucuagcugGaug...................................................................  6   1   seq
....................ucuuugguCaucuagcuguaug...................................................................  3   1   seq
....................ucuuugguuaucuagcCguaug...................................................................  7   1   seq
....................ucuuugguuGucuagcuguaug...................................................................  8   1   seq
....................ucAuugguuaucuagcuguaug...................................................................  8   1   seq
....................ucuuugguuaucuCgcuguaug...................................................................  9   1   seq
....................ucuuugguuaucAagcuguaug...................................................................  1   1   seq
....................ucuuuggCuaucuagcuguaug...................................................................  9   1   seq
....................ucuuugguuaucuaUcuguaug...................................................................  3   1   seq
....................ucuuugUuuaucuagcuguaug...................................................................  14  1   seq
....................ucuAugguuaucuagcuguaug...................................................................  1   1   seq
....................ucuuugguuaucuagAuguauga..................................................................  5   1   seq
....................ucuuugguuaAcuagcuguauga..................................................................  1   1   seq
....................ucuuugguuaucuaAcuguauga..................................................................  6   1   seq
....................ucuuugguuaucuagcAguauga..................................................................  2   1   seq
....................ucuuugguuaucuaUcuguauga..................................................................  4   1   seq
....................ucuuuggGuaucuagcuguauga..................................................................  21  1   seq
....................ucuuugguuauUuagcuguauga..................................................................  4   1   seq
....................ucuuuggCuaucuagcuguauga..................................................................  16  1   seq
....................ucuuugguuaGcuagcuguauga..................................................................  5   1   seq
....................ucuuuggAuaucuagcuguauga..................................................................  1   1   seq
....................ucuuugguuaucCagcuguauga..................................................................  14  1   seq
....................ucuuugguuauGuagcuguauga..................................................................  1   1   seq
....................ucuuugguuaucuagcGguauga..................................................................  2   1   seq
....................ucuuugguuaucGagcuguauga..................................................................  1   1   seq
....................ucuuugAuuaucuagcuguauga..................................................................  9   1   seq
....................ucuuugguuaucuCgcuguauga..................................................................  18  1   seq
```

Mature / Star

```
                    Mature                                      Star
ucauacugacucugcuuuucucuuugguuaucuagcuguaugagugaugucaaugcaucauaaagcuagguuaccaaagauaagugccuguugucacaugucgccccug
....................ucuuugguAaucuagcuguauga..................................................................   2   1   seq
....................ucuuugguuaucAagcuguauga..................................................................   2   1   seq
....................ucuuugguuaucuagcCguauga..................................................................   28  1   seq
....................ucuuugguuaucuagGuguauga..................................................................   2   1   seq
....................ucuuuggGaucuagcuguauga..................................................................   2   1   seq
....................ucuuugguuaucuagUuguauga..................................................................   9   1   seq
....................ucuuugUuuaucuagcuguauga..................................................................   28  1   seq
....................ucuuugguCaucuagcuguauga..................................................................   13  1   seq
....................ucuuugguuaucuUgcuguauga..................................................................   2   1   seq
....................ucuuugguuGucuagcuguauga..................................................................   16  1   seq
....................ucuuugguCaucuagcuguaugag.................................................................   1   1   seq
....................ucuuugguuauUuagcuguaugagu................................................................   1   1   seq
......................uuuUguuaucuagcuguau....................................................................   1   1   seq
......................uuuUguuaucuagcuguauga..................................................................   1   1   seq
...........................................................aAaaagcuagguuaccaaaga.............................   1   1   seq
...........................................................auaaagcuCgguuaccaaaga.............................   1   1   seq
...........................................................auaaagUuagguuaccaaaga.............................   1   1   seq
...........................................................auaaagcuagguuGccaaagau............................   1   1   seq
...........................................................auaaagcuGgguuaccaaagau............................   1   1   seq
...........................................................auaCagcuagguuaccaaagau............................   1   1   seq
...........................................................auaaGgcuagguuaccaaagaua...........................   2   1   seq
...........................................................auGaagcuagguuaccaaagaua...........................   1   1   seq
...........................................................auaaagcuCgguuaccaaagaua...........................   1   1   seq
...........................................................auaaagcuagguuaccaaGgaua...........................   1   1   seq
...........................................................auaaagcuagguuacAaaagaua...........................   1   1   seq
...........................................................auaaagcAagguuaccaaagaua...........................   1   1   seq
...........................................................auaaagcuagguuaAcaaagaua...........................   1   1   seq
...........................................................auaaagcuaggCuaccaaagaua...........................   1   1   seq
...........................................................auaGagcuagguuaccaaagaua...........................   4   1   seq
...........................................................auaaagcuagguuaUcaaagaua...........................   1   1   seq
...........................................................auaaagcuagguuaccaaaCaua...........................   1   1   seq
...........................................................auaaagcuGgguuaccaaagaua...........................   1   1   seq
...........................................................auaaagcuagguuaccaaaUaua...........................   1   1   seq
...........................................................auaaagcuaggGuaccaaagaua...........................   3   1   seq
............................................................uaaagcuagguuaccaaGgau............................   2   1   seq
............................................................uaaagcuagguuGccaaagau............................   1   1   seq
............................................................uGaagcuagguuaccaaagau............................   1   1   seq
............................................................uaaGgcuagguuaccaaagau............................   1   1   seq
............................................................uaaagcuagguuaccaUagau............................   1   1   seq
............................................................uaaagcuagguuacUaaagau............................   1   1   seq
............................................................uaaagcuagguuaccGaagaua...........................   1   1   seq
............................................................uaaagcuagguuaccaCagaua...........................   1   1   seq
............................................................uaaGgcuagguuaccaaagaua...........................   11  1   seq
............................................................uaUagcuagguuaccaaagaua...........................   2   1   seq
............................................................uGaagcuagguuaccaaagaua...........................   4   1   seq
............................................................uaaagcuGgguuaccaaagaua...........................   1   1   seq
............................................................uaaagcuagguCaccaaagaua...........................   1   1   seq
............................................................uaaagcuaggCuaccaaagaua...........................   2   1   seq
............................................................uaGagcuagguuaccaaagaua...........................   11  1   seq
............................................................uaaagcuagguuGccaaagaua...........................   3   1   seq
............................................................uaaagcuaggAuaccaaagaua...........................   2   1   seq
............................................................uaaagcuagguuaccaaGgaua...........................   2   1   seq
............................................................uaaagcuagguuaccaaaUaua...........................   1   1   seq
............................................................uUaagcuagguuaccaaagaua...........................   12  1   seq
............................................................uaaagAuagguuaccaaagaua...........................   1   1   seq
............................................................uaaagcuagAuuaccaaagaua...........................   1   1   seq
............................................................uaaagcuaAguuaccaaagaua...........................   1   1   seq
............................................................uaaagcCagguuaccaaagaua...........................   3   1   seq
............................................................uaaagcuagguAaccaaagaua...........................   1   1   seq
............................................................uaaagcuagguuaccCaagaua...........................   1   1   seq
............................................................uaaaAcuagguuaccaaagaua...........................   3   1   seq
............................................................uaaagcuagguuaccaGagaua...........................   5   1   seq
............................................................uaaagcuagguuacUaaagaua...........................   1   1   seq
............................................................uaaagcuaggGuaccaaagaua...........................   1   1   seq
............................................................uaaagcuagguuaUcaaagaua...........................   2   1   seq
.............................................................aaaUcuagguuaccaaagaua...........................   7   1   seq
.............................................................aaaAcuagguuaccaaagaua...........................   1   1   seq
.............................................................aUagcuagguuaccaaagaua...........................   1   1   seq
.............................................................aaagcuGgguuaccaaagaua...........................   1   1   seq
.............................................................aaagcCagguuaccaaagaua...........................   1   1   seq
```

Mature Star

```
ucauacugacucugcuuuucucuuugguuaucuagcuguaugagugaugucaaugcaucauaaagcuagguuaccaaagauaagugccuguugucacaugucgccccug
.............................................................aGagcuagguuaccaaagaua...........................	1	1	seq
..............................................................aagcuagguuaccaaagaua...........................	2	0	seq
```
