# Supplementary material for: Identification and Characterization of MicroRNAs from Longitudinal Muscle and Respiratory Tree in Sea Cucumber (Apostichopus japonicus) Using High-Throughput Sequencing
Source: PLoS One. 2015 Aug 5;10(8):e0134899. doi: 10.1371/journal.pone.0134899 (PMC4526669; doi:10.1371/journal.pone.0134899)
Supplement: S2 File — (ZIP) [file pone.0134899.s003.zip › S2 File/The secondary structures of the novel miRNAs in RPT/Scaffold22_115.pdf]

Provisional ID	:	Scaffold22_115
Score total	:	2.3
Score for star read(s)	:	-1.3
Score for read counts	:	0
Score for mfe	:	2
Score for randfold	:	1.6
Score for cons. seed	:	
Total read count	:	1016
Mature read count	:	1016
Loop read count	:	0
Star read count	:	0

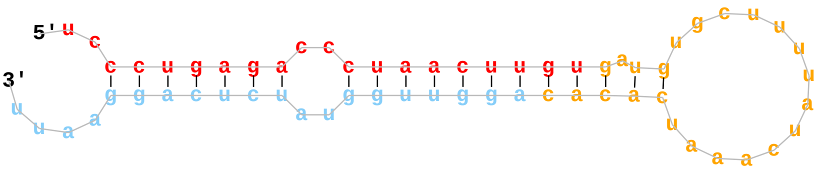

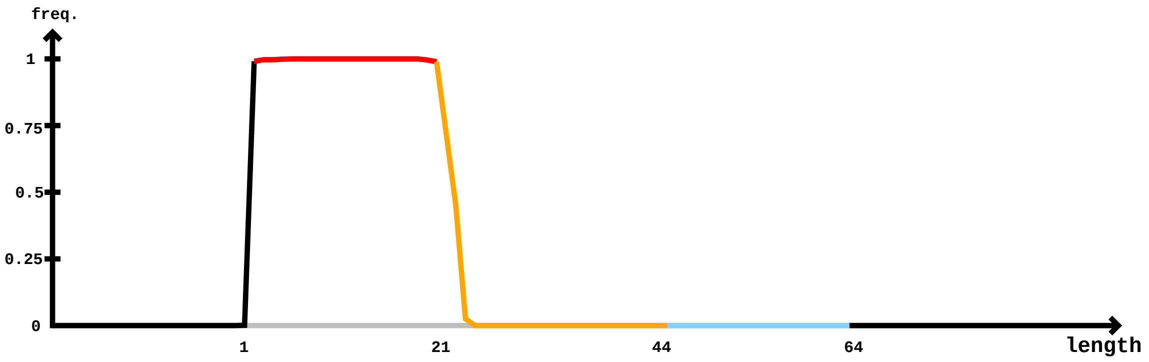

Mature **Star**

```
5'- agagagacugccccccaaaucccugagacccuaacuugugaugugcuuuuaucaaaucacacagguugguaucucaggaauuuggugggcgugucaugccaacgaucuc   -3'   exp
    .((((((((((((.((((((.(((((((..((((((((((.((..............))))))))))))..))))))).)))))).))))).)))..........))))    reads    mm    sample
    ..................aucccGgagacccuaacuuguga....................................................................    1        1     seq
    ...................ucccugagacccuaacuu........................................................................    3        0     seq
    ...................uccGugagacccuaacuu........................................................................    1        1     seq
    ...................ucccugagacccuaacuug.......................................................................    7        0     seq
    ...................ucccugagUcccuaacuugu......................................................................    1        1     seq
    ...................ucccugagacccuaacuuUu......................................................................    1        1     seq
    ...................ucccugagacccuaGcuugu......................................................................    3        1     seq
    ...................ucccugagacccuaacuugG......................................................................    6        1     seq
    ...................uccGugagacccuaacuugu......................................................................    11       1     seq
    ...................ucUcugagacccuaacuugu......................................................................    3        1     seq
    ...................ucccugagacccuGacuugu......................................................................    3        1     seq
    ...................ucccugagacccuaacCugu......................................................................    2        1     seq
    ...................ucccugagacccCaacuugu......................................................................    3        1     seq
    ...................ucccugGgacccuaacuugu......................................................................    3        1     seq
    ...................ucccugagacccuaacuGgu......................................................................    1        1     seq
    ...................ucccugaUacccuaacuugu......................................................................    1        1     seq
    ...................uUccugagacccuaacuugu......................................................................    7        1     seq
    ...................ucccugagacccuCacuugu......................................................................    1        1     seq
    ...................ucccugagacUcuaacuugu......................................................................    1        1     seq
    ...................uGccugagacccuaacuugu......................................................................    4        1     seq
    ...................ucccugagacccuaacAugu......................................................................    1        1     seq
    ...................ucccugagacccuaacuugA......................................................................    12       1     seq
    ...................ucGcugagacccuaacuugu......................................................................    1        1     seq
    ...................Acccugagacccuaacuugu......................................................................    2        1     seq
    ...................uccAugagacccuaacuugu......................................................................    1        1     seq
    ...................ucccugagacccuaacuugC......................................................................    62       1     seq
    ...................ucccugagaccUuaacuugu......................................................................    1        1     seq
    ...................ucccugagGcccuaacuugu......................................................................    7        1     seq
    ...................ucccugaCacccuaacuugu......................................................................    1        1     seq
    ...................ucccugagCcccuaacuugu......................................................................    6        1     seq
    ...................Gcccugagacccuaacuugu......................................................................    9        1     seq
    ...................ucccugagacUcuaacuugug.....................................................................    12       1     seq
    ...................ucccugagaccAuaacuugug.....................................................................    1        1     seq
    ...................ucccugagacccAaacuugug.....................................................................    1        1     seq
```

```
                  Mature                                    Star
agagagacugcccccaaaucccugagacccuaacuugugaugugcuuuuaucaaaucacacagguugguaucucaggaauuuggugggcgugucaugccaacgaucuc
..................ucccAgagacccuaacuugug.....................................................................  2   1  seq
..................ucccuCagacccuaacuugug.....................................................................  1   1  seq
..................ucccugagacccCaacuugug.....................................................................  11  1  seq
..................ucccugagaccUuaacuugug.....................................................................  5   1  seq
..................ucccCgagacccuaacuugug.....................................................................  13  1  seq
..................ucccugagacccuaacGugug.....................................................................  1   1  seq
..................ucccugagacccuaaAuugug.....................................................................  3   1  seq
..................ucccugagacccuaacuuAug.....................................................................  5   1  seq
..................ucccugagacccuaacuuUug.....................................................................  5   1  seq
..................ucccugagacccuaacuGgug.....................................................................  2   1  seq
..................ucccugagaccGuaacuugug.....................................................................  1   1  seq
..................ucccugagCcccuaacuugug.....................................................................  29  1  seq
..................ucccugagacccuaaUuugug.....................................................................  2   1  seq
..................ucccuAagacccuaacuugug.....................................................................  9   1  seq
..................ucccugaUacccuaacuugug.....................................................................  1   1  seq
..................uccUugagacccuaacuugug.....................................................................  1   1  seq
..................uccGugagacccuaacuugug.....................................................................  38  1  seq
..................uGccugagacccuaacuugug.....................................................................  19  1  seq
..................ucccugagacccuaacuugGg.....................................................................  16  1  seq
..................ucccugagacccuaacuuCug.....................................................................  1   1  seq
..................ucccugagacccuaacuugCg.....................................................................  18  1  seq
..................ucccugagacccuaacAugug.....................................................................  1   1  seq
..................ucccugagacccuaGcuugug.....................................................................  18  1  seq
..................uUccugagacccuaacuugug.....................................................................  46  1  seq
..................ucUcugagacccuaacuugug.....................................................................  12  1  seq
..................ucccugagacccuCacuugug.....................................................................  5   1  seq
..................ucccugUgacccuaacuugug.....................................................................  2   1  seq
..................ucccugagUcccuaacuugug.....................................................................  1   1  seq
..................ucccugagacccuaacuAgug.....................................................................  1   1  seq
..................ucccugagacccuaacCugug.....................................................................  18  1  seq
..................ucccugagaAccuaacuugug.....................................................................  1   1  seq
..................ucccugagacccuaacuugAg.....................................................................  5   1  seq
..................ucccugagaGccuaacuugug.....................................................................  2   1  seq
..................ucccugaAacccuaacuugug.....................................................................  2   1  seq
..................ucccugagacccuaUcuugug.....................................................................  1   1  seq
..................ucccGgagacccuaacuugug.....................................................................  3   1  seq
..................ucGcugagacccuaacuugug.....................................................................  1   1  seq
..................ucccugagaUccuaacuugug.....................................................................  7   1  seq
..................ucccuUagacccuaacuugug.....................................................................  2   1  seq
..................ucccugagGcccuaacuugug.....................................................................  27  1  seq
..................ucccugagacccuGacuugug.....................................................................  13  1  seq
..................ucccugagacccuaaGuugug.....................................................................  1   1  seq
..................ucccugGgacccuaacuugug.....................................................................  23  1  seq
..................ucccugagacAcuaacuugug.....................................................................  2   1  seq
..................uccAugagacccuaacuugug.....................................................................  2   1  seq
..................ucccugaUacccuaacuuguga....................................................................  5   1  seq
..................ucccugagacccCaacuuguga....................................................................  15  1  seq
..................ucccugagacccuaaUuuguga....................................................................  3   1  seq
..................ucccugaAacccuaacuuguga....................................................................  3   1  seq
..................ucccugagacccuaaAuuguga....................................................................  5   1  seq
..................ucccugGgacccuaacuuguga....................................................................  26  1  seq
..................ucccugagCcccuaacuuguga....................................................................  31  1  seq
..................ucccuUagacccuaacuuguga....................................................................  1   1  seq
..................ucccugCgacccuaacuuguga....................................................................  2   1  seq
..................ucccugagacccuGacuuguga....................................................................  25  1  seq
..................ucccugagacccuaaGuuguga....................................................................  2   1  seq
..................ucccuAagacccuaacuuguga....................................................................  3   1  seq
..................ucccugagacccuaacuugAga....................................................................  1   1  seq
..................ucccugagacGcuaacuuguga....................................................................  1   1  seq
..................uccGugagacccuaacuuguga....................................................................  48  1  seq
..................ucccugagacccGaacuuguga....................................................................  2   1  seq
..................ucccugagaAccuaacuuguga....................................................................  2   1  seq
..................ucccugagGcccuaacuuguga....................................................................  32  1  seq
..................ucccugagacccuaUcuuguga....................................................................  1   1  seq
..................ucccugagaccGuaacuuguga....................................................................  2   1  seq
..................ucccugagacccuaacuuCuga....................................................................  4   1  seq
..................ucccugagacccuaCcuuguga....................................................................  2   1  seq
..................ucccGgagacccuaacuuguga....................................................................  2   1  seq
..................ucAcugagacccuaacuuguga....................................................................  1   1  seq
..................ucUcugagacccuaacuuguga....................................................................  28  1  seq
```

```
                   Mature                                       Star
agagagacugccccccaaaucccugagacccuaacuugugaugugcuuuuaucaaaucacacagguugguaucucaggaauuuggugggcgugucaugccaacgaucuc
...................ucccugagacccuaacuugGga....................................................................  8    1    seq
...................ucccugagacccuaacAuguga....................................................................  2    1    seq
...................ucccuCagacccuaacuuguga....................................................................  1    1    seq
...................uccUugagacccuaacuuguga....................................................................  8    1    seq
...................ucccugagacccuaGcuuguga....................................................................  17   1    seq
...................ucccugUgacccuaacuuguga....................................................................  5    1    seq
...................ucccugagacccuaacGuguga....................................................................  2    1    seq
...................ucccugagacccAaacuuguga....................................................................  2    1    seq
...................ucccCgagacccuaacuuguga....................................................................  15   1    seq
...................ucccugagacccuUacuuguga....................................................................  1    1    seq
...................ucGcugagacccuaacuuguga....................................................................  3    1    seq
...................ucccugagacccuaacuAguga....................................................................  1    1    seq
...................ucccugagacccuaacuuAuga....................................................................  3    1    seq
...................ucccugagaccAuaacuuguga....................................................................  1    1    seq
...................ucccugagaccUuaacuuguga....................................................................  11   1    seq
...................ucccugagUcccuaacuuguga....................................................................  4    1    seq
...................ucccugagaUccuaacuuguga....................................................................  2    1    seq
...................ucccugagacccuaacuugCga....................................................................  19   1    seq
...................ucccugagacccuCacuuguga....................................................................  1    1    seq
...................ucccugagacccuaacCuguga....................................................................  25   1    seq
...................ucccugagacccuaacuuUuga....................................................................  7    1    seq
...................ucccugagacUcuaacuuguga....................................................................  26   1    seq
...................uccAugagacccuaacuuguga....................................................................  1    1    seq
...................ucccAgagacccuaacuuguga....................................................................  4    1    seq
...................ucccugagacccuaacuGguga....................................................................  5    1    seq
...................ucccugagaGccuaacuuguga....................................................................  1    1    seq
...................ucccugagacccuaacuugAgau...................................................................  1    1    seq
...................ucccugaUacccuaacuugugau...................................................................  1    1    seq
...................ucccugagacccuaacuugCgau...................................................................  1    1    seq
...................ucUcugagacccuaacuugugau...................................................................  2    1    seq
...................ucccCgagacccuaacuugugau...................................................................  1    1    seq
...................uccAugagacccuaacuugugau...................................................................  1    1    seq
...................ucccugagGcccuaacuugugau...................................................................  2    1    seq
...................ucccugagCcccuaacuugugau...................................................................  5    1    seq
...................ucccugagacccuGacuugugau...................................................................  1    1    seq
...................ucccugagacccuaacuugGgau...................................................................  1    1    seq
...................ucccugGgacccuaacuugugau...................................................................  4    1    seq
...................ucccugagaUccuaacuugugau...................................................................  1    1    seq
...................ucccGgagacccuaacuugugau...................................................................  1    1    seq
...................ucccugUgacccuaacuugugau...................................................................  1    1    seq
...................uccGugagacccuaacuugugau...................................................................  3    1    seq
....................ccUugagacccuaacuuguga....................................................................  1    1    seq
....................cUcugagacccuaacuuguga....................................................................  1    1    seq
....................cccugagacccuGacuuguga....................................................................  1    1    seq
....................cccGgagacccuaacuuguga....................................................................  1    1    seq
....................cccugagaUccuaacuuguga....................................................................  1    1    seq
....................cccugagacccAaacuuguga....................................................................  1    1    seq
......................cugagacccuaacuuguga....................................................................  1    0    seq
......................cugGgacccuaacuuguga....................................................................  1    1    seq
.......................ugagacccuaacuuguga....................................................................  1    0    seq
```
