# Supplementary material for: Identification and Characterization of MicroRNAs from Longitudinal Muscle and Respiratory Tree in Sea Cucumber (Apostichopus japonicus) Using High-Throughput Sequencing
Source: PLoS One. 2015 Aug 5;10(8):e0134899. doi: 10.1371/journal.pone.0134899 (PMC4526669; doi:10.1371/journal.pone.0134899)
Supplement: S2 File — (ZIP) [file pone.0134899.s003.zip › S2 File/The secondary structures of the novel miRNAs in RPT/Scaffold22_117.pdf]

Provisional ID	:	Scaffold22_117
Score total	:	6033.4
Score for star read(s)	:	3.9
Score for read counts	:	6026.3
Score for mfe	:	1.7
Score for randfold	:	1.6
Score for cons. seed	:	
Total read count	:	11832
Mature read count	:	11708
Loop read count	:	0
Star read count	:	124

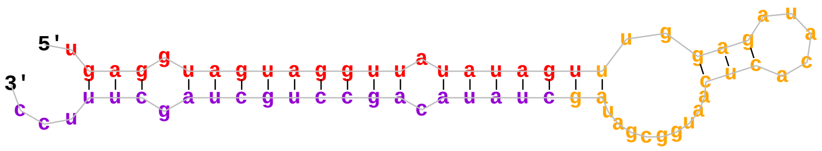

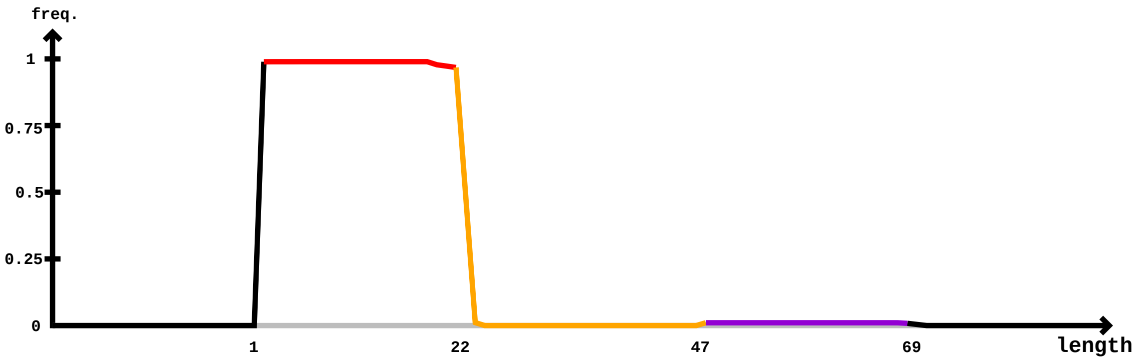

Mature Star

5'- guacaugaauggucCuuacgugagguaguagguuauauaguuuggagauacacucaauggcgauagcuauacagccugcuagcuuccuugggccauucacaaaucuu -3' obs

guacaugaauggucCuuacgugagguaguagguuauauaguuuggagauacacucaauggcgauagcuauacagccugcuagcuuccuugggccauucacaaaucuu exp

.....((((((((((......(((.(((((((((.(((((((..(((.....))).........))))))).))))))))).)))....))))))))))........	reads	mm	sample
....................ugagguaAuagguuauau...	1	1	seq
....................ugagguaguagguuauaC...	6	1	seq
....................ugagguagAagguuauau...	1	1	seq
....................ugaUguaguagguuauau...	14	1	seq
....................uUagguaguagguuauau...	1	1	seq
....................ugCgguaguagguuauau...	1	1	seq
....................ugagguGguagguuauau...	1	1	seq
....................ugagguaguagguuauau...	105	0	seq
....................ugagguaguagguuauaG...	1	1	seq
....................ugagguaguagguCauau...	1	1	seq
....................ugGgguaguagguuauau...	1	1	seq
....................ugagguaguagguuGuau...	1	1	seq
....................ugaggAaguagguuauau...	1	1	seq
....................ugagguagCagguuauau...	1	1	seq
....................ugagguaguagguuauauU..	4	1	seq
....................uUagguaguagguuauaua..	5	1	seq
....................ugagguaguagguuauaua..	42	0	seq
....................ugaUguaguagguuauaua..	6	1	seq
....................ugaggCaguagguuauaua..	2	1	seq
....................ugagguaguagguuauauaA...	9	1	seq
....................ugagguagCagguuauauag...	1	1	seq
....................ugagguaguaggGuauauag...	1	1	seq
....................ugagguaguaAguuauauag...	2	1	seq
....................ugaggAaguagguuauauag...	1	1	seq
....................ugUgguaguagguuauauag...	1	1	seq
....................ugagguaguagguuaCauag...	1	1	seq
....................ugagguagGagguuauauag...	1	1	seq
....................Ggagguaguagguuauauag...	1	1	seq
....................ugagguaguagguuauauUg...	1	1	seq
....................ugagguaguaggCuauauag...	2	1	seq
....................ugagguaguagguuauauaU...	3	1	seq
....................ugagguaguaUguuauauag...	1	1	seq
....................Cgagguaguagguuauauag...	2	1	seq

Mature Star

```
guacaugaaugguccuuacgugagguaguagguuauauaguuuggagauacacucaauggcgauagcuauacagccugcuagcuuuccuugggccauucacaaaucuu
....................ugagguaguagguuUuauag.................................................................... 1 1 seq
....................uUagguaguagguuauauag.................................................................... 4 1 seq
....................ugaggCaguagguuauauag.................................................................... 1 1 seq
....................ugaUguaguagguuauauag.................................................................... 25 1 seq
....................ugGgguaguagguuauauag.................................................................... 4 1 seq
....................ugaggCaguagguuauauagu................................................................... 215 1 seq
....................ugagUuaguagguuauauagu................................................................... 17 1 seq
....................ugagguaguagguuauUuagu................................................................... 5 1 seq
....................ugagguUguagguuauauagu................................................................... 14 1 seq
....................uAagguaguagguuauauagu................................................................... 53 1 seq
....................ugagguGguagguuauauagu................................................................... 131 1 seq
....................ugaUguaguagguuauauagu................................................................... 3125 1 seq
....................ugagguagAagguuauauagu................................................................... 50 1 seq
....................ugaggAaguagguuauauagu................................................................... 146 1 seq
....................ugagguaguagguuauGuagu................................................................... 98 1 seq
....................ugagguaguagguuauauaUu................................................................... 40 1 seq
....................ugagguaguagguuaAauagu................................................................... 3 1 seq
....................ugagguaguagguuCuauagu................................................................... 2 1 seq
....................ugagguCguagguuauauagu................................................................... 4 1 seq
....................ugagguaCuagguuauauagu................................................................... 2 1 seq
....................ugagguaguaAguuauauagu................................................................... 32 1 seq
....................ugagCuaguagguuauauagu................................................................... 2 1 seq
....................uCagguaguagguuauauagu................................................................... 75 1 seq
....................ugagguagGagguuauauagu................................................................... 35 1 seq
....................ugCgguaguagguuauauagu................................................................... 18 1 seq
....................ugagguaguagguuauCuagu................................................................... 3 1 seq
....................ugagguaguUgguuauauagu................................................................... 21 1 seq
....................ugagguaguagguuauaGagu................................................................... 5 1 seq
....................ugagguaguaggCuauauagu................................................................... 187 1 seq
....................ugagguaguagguuauauGgu................................................................... 93 1 seq
....................ugUgguaguagguuauauagu................................................................... 290 1 seq
....................ugagguaguaCguuauauagu................................................................... 1 1 seq
....................ugagguaguagguuauauCgu................................................................... 3 1 seq
....................ugagguaguagguuaCauagu................................................................... 60 1 seq
....................ugaggGaguagguuauauagu................................................................... 135 1 seq
....................ugaCguaguagguuauauagu................................................................... 23 1 seq
....................ugagguaguagguCauauagu................................................................... 78 1 seq
....................ugagguaguaggGuauauagu................................................................... 46 1 seq
....................ugagguagCagguuauauagu................................................................... 86 1 seq
....................ugagguaAuagguuauauagu................................................................... 17 1 seq
....................ugagguaguGgguuauauagu................................................................... 137 1 seq
....................ugagguaguagguGauauagu................................................................... 10 1 seq
....................ugagguaUuagguuauauagu................................................................... 20 1 seq
....................ugagguaguCgguuauauagu................................................................... 7 1 seq
....................ugagguaguagguAauauagu................................................................... 9 1 seq
....................ugagguaguagguuauauaAu................................................................... 18 1 seq
....................ugagguaguagguuauauaCu................................................................... 5 1 seq
....................ugagguaguagAuuauauagu................................................................... 18 1 seq
....................ugagguaguaggAuauauagu................................................................... 33 1 seq
....................ugGgguaguagguuauauagu................................................................... 247 1 seq
....................ugagAuaguagguuauauagu................................................................... 25 1 seq
....................ugagguaguagguuaGauagu................................................................... 10 1 seq
....................ugagguaguagUuuauauagu................................................................... 24 1 seq
....................ugagguaguagguuUuauagu................................................................... 5 1 seq
....................ugagguaguagCuuauauagu................................................................... 3 1 seq
....................ugagguaguagguuauaCagu................................................................... 45 1 seq
....................ugagguaguaUguuauauagu................................................................... 62 1 seq
....................ugaAguaguagguuauauagu................................................................... 40 1 seq
....................ugagguaguagguuauaAagu................................................................... 3 1 seq
....................ugagguaguagguuauauUgu................................................................... 15 1 seq
....................uUagguaguagguuauauagu................................................................... 1023 1 seq
....................ugagguaguagguuCuauaguu.................................................................. 1 1 seq
....................ugagguaAuagguuauauaguu.................................................................. 37 1 seq
....................ugagUuaguagguuauauaguu.................................................................. 10 1 seq
....................ugagguaguagguuauUuaguu.................................................................. 3 1 seq
....................ugaggAaguagguuauauaguu.................................................................. 84 1 seq
....................ugagguaguagguuauCuaguu.................................................................. 3 1 seq
....................ugagguaguaggCuauauaguu.................................................................. 143 1 seq
....................ugagguaguagguuauauaCuu.................................................................. 5 1 seq
....................ugagguagCagguuauauaguu.................................................................. 76 1 seq
```

```
                    Mature                                       Star
guacaugaauggucuuacgugagguaguagguuauauaguuuggagauacacucaauggcgauagcuauacagccugcuagcuuuccuugggccauucacaaaucuu
...................ugagguagAagguuauauaguu..................................................................   55     1     seq
...................ugagguaguagguuauauUguu..................................................................   13     1     seq
...................ugagguaguagCuuauauaguu..................................................................   1      1     seq
...................ugagguaguagguuauauaAuu..................................................................   14     1     seq
...................ugagguaguaggGuauauaguu..................................................................   38     1     seq
...................ugagguaguaggAuauauaguu..................................................................   29     1     seq
...................ugagguaguagguuauaAaguu..................................................................   4      1     seq
...................ugagguUguagguuauauaguu..................................................................   9      1     seq
...................ugaggCaguagguuauauaguu..................................................................   125    1     seq
...................ugaUguaguagguuauauaguu..................................................................   2511   1     seq
...................ugaAguaguagguuauauaguu..................................................................   36     1     seq
...................ugagguaguagguuUuauaguu..................................................................   4      1     seq
...................ugagguaguagguAauauaguu..................................................................   11     1     seq
...................ugagguGguagguuauauaguu..................................................................   84     1     seq
...................ugagguaguaCguuauauaguu..................................................................   3      1     seq
...................ugagguaguagguuauGuaguu..................................................................   61     1     seq
...................ugagguaguCgguuauauaguu..................................................................   4      1     seq
...................ugaggGaguagguuauauaguu..................................................................   100    1     seq
...................ugagguaUuagguuauauaguu..................................................................   15     1     seq
...................ugagguaguagguuauauCguu..................................................................   1      1     seq
...................ugagguaguagguuaGauaguu..................................................................   2      1     seq
...................ugagCuaguagguuauauaguu..................................................................   2      1     seq
...................ugagguaguagguuauauaUuu..................................................................   34     1     seq
...................ugagguaguagguuauauGguu..................................................................   65     1     seq
...................ugagguaguUgguuauauaguu..................................................................   6      1     seq
...................ugagguaCuagguuauauaguu..................................................................   7      1     seq
...................ugagguaguGgguuauauaguu..................................................................   104    1     seq
...................ugagguaguagguuaAauaguu..................................................................   3      1     seq
...................ugagguaguagguuaCauaguu..................................................................   55     1     seq
...................ugagguaguagguuauaCaguu..................................................................   34     1     seq
...................ugagguagGagguuauauaguu..................................................................   25     1     seq
...................ugagguaguagAuuauauaguu..................................................................   9      1     seq
...................ugGgguaguagguuauauaguu..................................................................   165    1     seq
...................ugagguaguaUguuauauaguu..................................................................   50     1     seq
...................ugaCguaguagguuauauaguu..................................................................   22     1     seq
...................ugCgguaguagguuauauaguu..................................................................   18     1     seq
...................ugagguaguaAguuauauaguu..................................................................   30     1     seq
...................ugagguCguagguuauauaguu..................................................................   3      1     seq
...................ugUgguaguagguuauauaguu..................................................................   217    1     seq
...................ugagguaguagguuauaGaguu..................................................................   3      1     seq
...................ugagguaguagguCauauaguu..................................................................   63     1     seq
...................ugagguaguagUuuauauaguu..................................................................   18     1     seq
...................ugagAuaguagguuauauaguu..................................................................   26     1     seq
...................ugagguaguagguGauauaguu..................................................................   1      1     seq
...................ugGgguaguagguuauauaguuu.................................................................   5      1     seq
...................ugagguaguagguuaGauaguuu.................................................................   2      1     seq
...................ugagguagGagguuauauaguuu.................................................................   1      1     seq
...................ugagguaguagguuauGuaguuu.................................................................   2      1     seq
...................ugagguaguagguuauauaUuuu.................................................................   4      1     seq
...................ugagguaguagguCauauaguuu.................................................................   2      1     seq
...................ugCgguaguagguuauauaguuu.................................................................   1      1     seq
...................ugagguaguaggAuauauaguuu.................................................................   2      1     seq
...................ugagguCguagguuauauaguuu.................................................................   1      1     seq
...................ugUgguaguagguuauauaguuu.................................................................   4      1     seq
...................ugaggCaguagguuauauaguuu.................................................................   9      1     seq
...................ugagguaguagguuaCauaguuu.................................................................   4      1     seq
...................ugaUguaguagguuauauaguuu.................................................................   74     1     seq
...................ugagguaguagguuauauGguuu.................................................................   2      1     seq
...................ugagguaguagguuauaCaguuu.................................................................   1      1     seq
...................ugagguaguaggCuauauaguuu.................................................................   3      1     seq
...................ugagguaAuagguuauauaguuu.................................................................   1      1     seq
...................ugagguaguGgguuauauaguuu.................................................................   2      1     seq
...................ugagguaguagguGauauaguuu.................................................................   1      1     seq
...................ugagguaguaggGuauauaguuu.................................................................   1      1     seq
...................ugagguaguUgguuauauaguuu.................................................................   1      1     seq
...................ugagguaguagguuauUuaguuu.................................................................   1      1     seq
...................ugagguagCagguuauauaguuu.................................................................   4      1     seq
...................ugaggGaguagguuauauaguuu.................................................................   2      1     seq
...................ugagguaguaCguuauauaguuu.................................................................   1      1     seq
....................gagUuaguagguuauauagu...................................................................   1      1     seq
```

```
                    Mature                                       Star
guacaugaaugguccuuacgugagguaguagguuauauaguuuggagauacacucaauggcgauagcuauacagccugcuagcuuuccuugggccauucacaaaucuu
.....................Uagguaguagguuauauagu...................................................................   1    1    seq
.....................gagUuaguagguuauauaguu..................................................................   1    1    seq
........................guaguagguuauauaguu..................................................................   1    0    seq
..................................................................cuauacaAccugcuagcuuu......................   1    1    seq
..................................................................cuauacaAccugcuagcuuuc.....................   25   1    seq
..................................................................cuauacaAccugcuagcuuucc....................   97   1    seq
..................................................................cuauacagccugcuagcuuuccA...................   1    1    seq
```
