# Supplementary material for: Identification and Characterization of MicroRNAs from Longitudinal Muscle and Respiratory Tree in Sea Cucumber (Apostichopus japonicus) Using High-Throughput Sequencing
Source: PLoS One. 2015 Aug 5;10(8):e0134899. doi: 10.1371/journal.pone.0134899 (PMC4526669; doi:10.1371/journal.pone.0134899)
Supplement: S2 File — (ZIP) [file pone.0134899.s003.zip › S2 File/The secondary structures of the novel miRNAs in RPT/Scaffold2335_2950.pdf]

Provisional ID	:	Scaffold2335_2950
Score total	:	1.8
Score for star read(s)	:	-1.3
Score for read counts	:	0
Score for mfe	:	1.5
Score for randfold	:	1.6
Score for cons. seed	:	
Total read count	:	71
Mature read count	:	71
Loop read count	:	0
Star read count	:	0

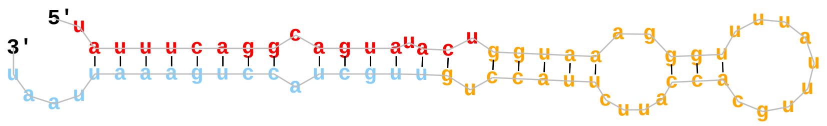

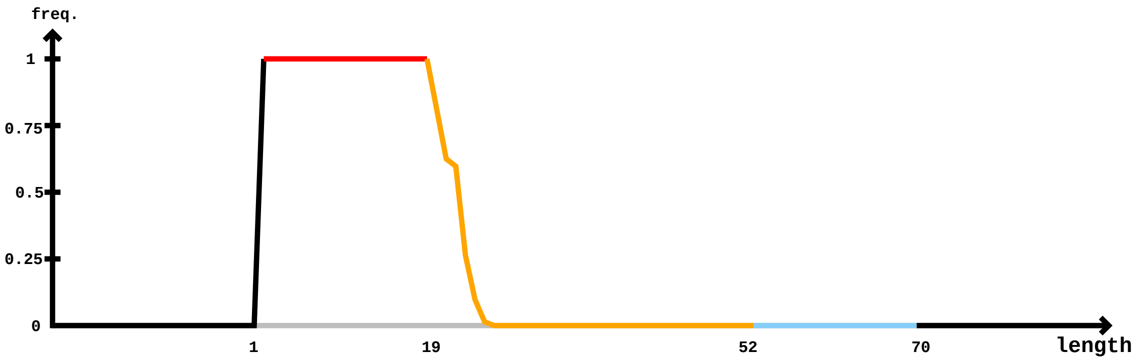

Mature **Star**

```
5'- guaucaucggcaucaugcauuauuucaggcaguauacugguaaaggguuuuauuugcaccauucuuaccuguugcuaccugaaauuaaugcaagaugugugaauaacc   -3'    exp
    ...((((..(((((.((((((((((((((.((((.((.(((((..(((.........)))....))))).)))))).))))))).))))))).)))))))))......    reads     mm       sample
    ....................uauuucaggcaguauacu......................................................................    12        0        seq
    ....................uauuucaggUaguauacu......................................................................    1         1        seq
    ....................uauuucaggcaguauacC......................................................................    1         1        seq
    ....................Gauuucaggcaguauacu......................................................................    1         1        seq
    ....................uauuucaggcaguGuacug.....................................................................    1         1        seq
    ....................uauuucaggcaguauacuU.....................................................................    8         1        seq
    ....................uauuucaggcaguauacug.....................................................................    3         0        seq
    ....................uUuuucaggcaguauacugg....................................................................    1         1        seq
    ....................uauuucaggcaguauacugU....................................................................    1         1        seq
    ....................uauuAcaggcaguauacuggu...................................................................    1         1        seq
    ....................uauuucaggcaUuauacuggu...................................................................    1         1        seq
    ....................uaCuucaggcaguauacuggu...................................................................    3         1        seq
    ....................uauuucaggcGguauacuggu...................................................................    1         1        seq
    ....................uauuucaAgcaguauacuggu...................................................................    1         1        seq
    ....................uauGucaggcaguauacuggu...................................................................    5         1        seq
    ....................uauuucGggcaguauacuggu...................................................................    3         1        seq
    ....................uauuuUaggcaguauacuggu...................................................................    2         1        seq
    ....................uauuucaggcaguauGcuggu...................................................................    2         1        seq
    ....................uUuuucaggcaguauacuggu...................................................................    1         1        seq
    ....................uauuCcaggcaguauacuggu...................................................................    2         1        seq
    ....................uauuucaggcaguGuacuggu...................................................................    2         1        seq
    ....................uauuucaggcGguauacuggua..................................................................    1         1        seq
    ....................uaGuucaggcaguauacuggua..................................................................    1         1        seq
    ....................uauuucaggcaguGuacuggua..................................................................    1         1        seq
    ....................uauuucaggcaguaCacuggua..................................................................    1         1        seq
    ....................uauuucagAcaguauacuggua..................................................................    1         1        seq
    ....................uauuCcaggcaguauacuggua..................................................................    1         1        seq
    ....................uauGucaggcaguauacuggua..................................................................    3         1        seq
    ....................uauuucaggcaAuauacuggua..................................................................    1         1        seq
    ....................uauuucaggcaUuauacuggua..................................................................    1         1        seq
    ....................uauuucGggcaguauacuggua..................................................................    1         1        seq
    ....................uauuucaggcaguGuacugguaa.................................................................    1         1        seq
    ....................uauuucaggGaguauacugguaa.................................................................    1         1        seq
    ....................uauuucaggUaguauacugguaa.................................................................    1         1        seq
```

```
                    Mature                                            Star
guaucaucggcaucaugcauuauuucaggcaguauacugguaaaggguuuuauuugcaccauucuuaccuguugcuaccugaaauuaaugcaagaugugugaauaacc
....................uauuucaggcaguCuacugguaa.................................................................	1	1	seq
....................uauuucaggcaguauaAugguaa.................................................................	1	1	seq
....................uauuucaggcagAauacugguaa.................................................................	1	1	seq
....................uauuucGggcaguauacugguaaa................................................................	1	1	seq
```
