# Supplementary material for: Identification and Characterization of MicroRNAs from Longitudinal Muscle and Respiratory Tree in Sea Cucumber (Apostichopus japonicus) Using High-Throughput Sequencing
Source: PLoS One. 2015 Aug 5;10(8):e0134899. doi: 10.1371/journal.pone.0134899 (PMC4526669; doi:10.1371/journal.pone.0134899)
Supplement: S2 File — (ZIP) [file pone.0134899.s003.zip › S2 File/The secondary structures of the novel miRNAs in RPT/Scaffold2497_3005.pdf]

Provisional ID : Scaffold2497_3005
Score total : 2930
Score for star read(s) : 3.9
Score for read counts : 2923
Score for mfe : 1.6
Score for randfold : 1.6
Score for cons. seed :
Total read count : 5745
Mature read count : 5589
Loop read count : 0
Star read count : 156

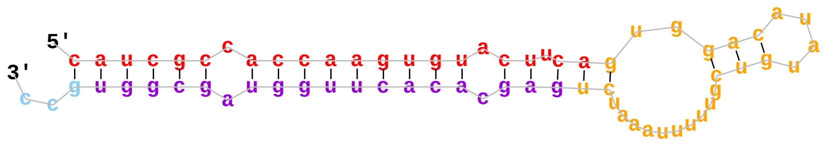

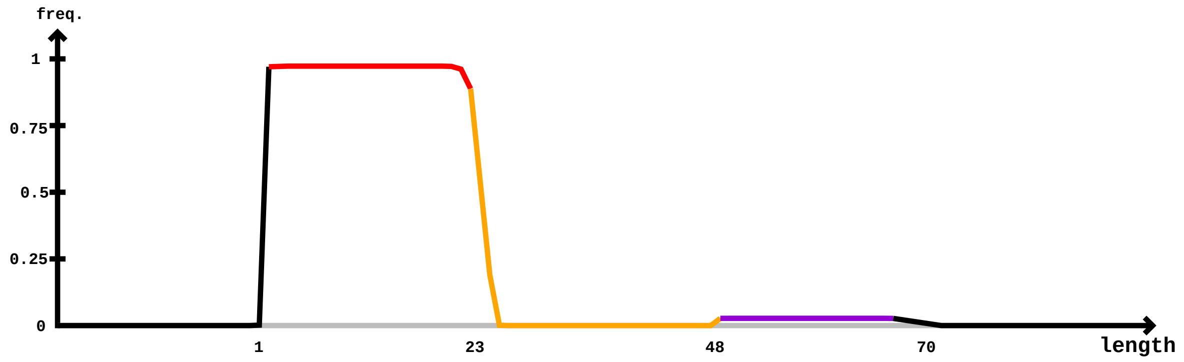

Mature **Star**

```
5'- cucaaggccguagcaugugccaucgccaccaaguguacuucaguggacauaugucguuuuuaaaucugagcacacuugguagcggugccgugagcuacucccauacaucgga -3'   obs
    cucaaggccguagcaugugccaucgccaccaaguguacuucaguggacauaugucguuuuuaaaucugagcacacuugguagcggugccgugagcuacucccauacaucgga        exp
    .....((..(((((.((((.((((((.(((((((((.((.(((..(((....)))..........))))).))))))))).)))))).)))).)))))..))..........   reads   mm   sample
    ...................ccaucgccaccaaguguacuuc.......................................................................   4   0   seq
    ...................ccaucgccaccaaguguacuuca......................................................................   4   0   seq
    ...................ccaucgccaccaaguguacuucagu....................................................................   1   0   seq
    ....................caucgccaccaaguguacu.........................................................................   5   0   seq
    ....................caucgccaccaaguguacC.........................................................................   1   1   seq
    ....................cUucgccaccaaguguacuu........................................................................   1   1   seq
    ....................caucgccaccaaguguacuC........................................................................   2   1   seq
    ....................caucgccaccaaguguacuu........................................................................   55   0   seq
    ....................caucgccGccaaguguacuu........................................................................   1   1   seq
    ....................cauUgccaccaaguguacuuc.......................................................................   1   1   seq
    ....................caucgccaccaagCguacuuc.......................................................................   2   1   seq
    ....................caCcgccaccaaguguacuuc.......................................................................   4   1   seq
    ....................cUucgccaccaaguguacuuc.......................................................................   1   1   seq
    ....................caucgccaccaaguguacuuA.......................................................................   1   1   seq
    ....................caucgccaccaGguguacuuc.......................................................................   2   1   seq
    ....................caucgccaccaagGguacuuc.......................................................................   1   1   seq
    ....................caucgccaccaaguguacuuU.......................................................................   7   1   seq
    ....................caucgccaccaagugCacuuc.......................................................................   1   1   seq
    ....................caucgccGccaaguguacuuc.......................................................................   1   1   seq
    ....................caucgccaccGaguguacuuc.......................................................................   1   1   seq
    ....................cauGgccaccaaguguacuuc.......................................................................   4   1   seq
    ....................caGcgccaccaaguguacuuc.......................................................................   1   1   seq
    ....................caucgccaccaaguguaUuuc.......................................................................   1   1   seq
    ....................caucgccaccaaguguacuuc.......................................................................   376   0   seq
    ....................Uaucgccaccaaguguacuuc.......................................................................   1   1   seq
    ....................cGucgccaccaaguguacuuc.......................................................................   1   1   seq
    ....................caucUccaccaaguguacuuc.......................................................................   1   1   seq
    ....................caAcgccaccaaguguacuuc.......................................................................   1   1   seq
    ....................caucgccaccaaguguacuCc.......................................................................   2   1   seq
    ....................caucgccaccaagAguacuuc.......................................................................   1   1   seq
    ....................caucgccacUaaguguacuuc.......................................................................   1   1   seq
    ....................caucgccaccaaguAuacuuc.......................................................................   2   1   seq
    ....................caucgccaccaaguguGcuuc.......................................................................   2   1   seq
```

```
                    Mature                                         Star
cucaaggccguagcaugugccaucgccaccaaguguacuucaguggacauaugucguuuuuaaaucugagcacacuugguagcgguggccgugagcuacucccauacaucgga
....................caucgccaccaagugCacuuca......................................................................    8      1    seq
....................caucgccaccaaguguacCuca......................................................................    9      1    seq
....................caGcgccaccaaguguacuuca......................................................................    23     1    seq
....................caucgccaccaaguguacGuca......................................................................    1      1    seq
....................caucgccaAcaaguguacuuca......................................................................    2      1    seq
....................caucAccaccaaguguacuuca......................................................................    5      1    seq
....................caucgccaccaaguguacuuca......................................................................    3050   0    seq
....................caucgccaccaaguguacuuGa......................................................................    2      1    seq
....................caucgccaccaagugGacuuca......................................................................    7      1    seq
....................cGucgccaccaaguguacuuca......................................................................    14     1    seq
....................caucgccaccaagAguacuuca......................................................................    10     1    seq
....................caucgccaccaaguguacuucU......................................................................    25     1    seq
....................caucgccaccCaguguacuuca......................................................................    3      1    seq
....................caucgccaccaaguguacuuUa......................................................................    2      1    seq
....................caucgccaccaagCguacuuca......................................................................    16     1    seq
....................caucgGcaccaaguguacuuca......................................................................    1      1    seq
....................caucgUcaccaaguguacuuca......................................................................    4      1    seq
....................caucgccaccaaguguacuGca......................................................................    1      1    seq
....................caucgccaccaaguguacuucC......................................................................    10     1    seq
....................cauUgccaccaaguguacuuca......................................................................    2      1    seq
....................caucgccacGaaguguacuuca......................................................................    2      1    seq
....................cUucgccaccaaguguacuuca......................................................................    13     1    seq
....................caucgccaccaaguguGcuuca......................................................................    8      1    seq
....................caucgccaccaaguAuacuuca......................................................................    4      1    seq
....................caucgccaUcaaguguacuuca......................................................................    3      1    seq
....................caucgccacUaaguguacuuca......................................................................    6      1    seq
....................caucgccaccaaguUuacuuca......................................................................    6      1    seq
....................Uaucgccaccaaguguacuuca......................................................................    2      1    seq
....................cCucgccaccaaguguacuuca......................................................................    1      1    seq
....................caucgccaccaaguguacuAca......................................................................    2      1    seq
....................caucgccaccaaguguaUuuca......................................................................    5      1    seq
....................caucgcUaccaaguguacuuca......................................................................    2      1    seq
....................caucgccaccaaguguUcuuca......................................................................    1      1    seq
....................cauAgccaccaaguguacuuca......................................................................    1      1    seq
....................caucgccGccaaguguacuuca......................................................................    14     1    seq
....................caucgccaccaGguguacuuca......................................................................    11     1    seq
....................caucgccaccGaguguacuuca......................................................................    14     1    seq
....................caucgccaccaaguguacuucG......................................................................    19     1    seq
....................Gaucgccaccaaguguacuuca......................................................................    1      1    seq
....................caucgccaccaaguguCcuuca......................................................................    1      1    seq
....................caucUccaccaaguguacuuca......................................................................    1      1    seq
....................caCcgccaccaaguguacuuca......................................................................    12     1    seq
....................caucgccaccaagGguacuuca......................................................................    11     1    seq
....................caucgccaccaaguguaGuuca......................................................................    1      1    seq
....................caucgccaccaaguguacuCca......................................................................    5      1    seq
....................caucgccaccaagugAacuuca......................................................................    5      1    seq
....................caAcgccaccaaguguacuuca......................................................................    7      1    seq
....................caucgccaccaaAuguacuuca......................................................................    1      1    seq
....................caucgAcaccaaguguacuuca......................................................................    1      1    seq
....................cauGgccaccaaguguacuuca......................................................................    25     1    seq
....................caucgccaccaaguguacAuca......................................................................    2      1    seq
....................caucgccGccaaguguacuucag.....................................................................    2      1    seq
....................caucgccaccaaguguacuucag.....................................................................    441    0    seq
....................cauGgccaccaaguguacuucag.....................................................................    2      1    seq
....................caucgccaccaaguguacuucCg.....................................................................    1      1    seq
....................cGucgccaccaaguguacuucag.....................................................................    4      1    seq
....................caucgccaccaaguAuacuucag.....................................................................    1      1    seq
....................caucgccaccaaguguGcuucag.....................................................................    1      1    seq
....................caucgccaccaaguguacCucag.....................................................................    1      1    seq
....................caucgccaccaagugCacuucag.....................................................................    3      1    seq
....................caucgccaccaaguguacuucaA.....................................................................    20     1    seq
....................caucgccaccaaguguacGucag.....................................................................    1      1    seq
....................cUucgccaccaaguguacuucag.....................................................................    2      1    seq
....................caucgccaccaaguguacuucGg.....................................................................    3      1    seq
....................caucAccaccaaguguacuucag.....................................................................    2      1    seq
....................caucgccaUcaaguguacuucag.....................................................................    2      1    seq
....................Gaucgccaccaaguguacuucag.....................................................................    1      1    seq
....................caucgccaccaaguguacuGcag.....................................................................    2      1    seq
....................caucgccaccaGguguacuucag.....................................................................    2      1    seq
....................caucgccaccaaguguacuCcag.....................................................................    1      1    seq
```

Mature **Star**

cucaaggccguagcaugugccaucgccaccaaguguacuucaguggacauaugucguuuuuaaaucugagcacacuugguagcggugccgugagcuacucccauacaucgga			
....................caGcgccaccaaguguacuucag...	5	1	seq
....................caucgccaccaaguguacuucaU...	101	1	seq
....................caucgccaccaaguguacuucaC...	14	1	seq
....................caucgccaccCaguguacuucag...	1	1	seq
....................caucgccaccaagAguacuucag...	1	1	seq
....................caCcgccaccaaguguacuucag...	7	1	seq
....................caucgccaccaaguAuacuucagu..	1	1	seq
....................caucgccaccaaguguGcuucagu..	5	1	seq
....................caucgccaccaGguguacuucagu..	1	1	seq
....................caucgccGccaaguguacuucagu..	3	1	seq
....................caucgccaccaaAuguacuucagu..	1	1	seq
....................caucgccaccaaguguacuucagu..	829	0	seq
....................caucgUcaccaaguguacuucagu..	1	1	seq
....................caucgccaccaaguguacuuAagu..	1	1	seq
....................caucgccaccaagGguacuucagu..	2	1	seq
....................caCcgccaccaaguguacuucagu..	5	1	seq
....................caucgccaccaaguUuacuucagu..	2	1	seq
....................caucgccaccaaguguacuucCgu..	2	1	seq
....................caAcgccaccaaguguacuucagu..	1	1	seq
....................caucgccaccaaguguacuucGgu..	7	1	seq
....................caGcgccaccaaguguacuucagu..	3	1	seq
....................caucgccaccaaguguacuGcagu..	2	1	seq
....................caucgccaccaaguguacCucagu..	1	1	seq
....................cUucgccaccaaguguacuucagu..	6	1	seq
....................Uaucgccaccaaguguacuucagu..	2	1	seq
....................caucgccaccaaguguacuucagA..	16	1	seq
....................cGucgccaccaaguguacuucagu..	2	1	seq
....................caucgccaccaaguguacuucagG..	2	1	seq
....................caucgccaccaaguguacuuUagu..	1	1	seq
....................caucgccaccaaguguacuuGagu..	1	1	seq
....................caucgccaccaagugGacuucagu..	3	1	seq
....................cauGgccaccaaguguacuucagu..	4	1	seq
....................caucgccaccaaguguacGucagu..	2	1	seq
....................caucgccaccaaguguacuucaCu..	5	1	seq
....................caucgccaccaaguguacuCcagu..	2	1	seq
....................caucgccaccaagCguacuucagu..	5	1	seq
....................caucgccaccaaguCuacuucagu..	1	1	seq
....................caucAccaccaaguguacuucagu..	1	1	seq
....................caucgcUaccaaguguacuucagu..	2	1	seq
....................caucgccaccaaguguacuucaUu..	93	1	seq
....................cauUgccaccaaguguacuucagu..	1	1	seq
....................caucgccaccGaguguacuucagu..	4	1	seq
....................caucgccaccaaguguacuucagC..	56	1	seq
....................caucgccaccaaguguacuucaguU...	6	1	seq
.....................aucgccaccaaguguacuuc...	1	0	seq
.....................aucgccaccaaguguacuuca..	4	0	seq
.....................aucgccaccaaguguacuucaU...	1	1	seq
.....................aucgccaccaaguguacuucagu..	1	0	seq
......................ucgccaccaaguguacuuA...	1	1	seq
......................ucgccaccaaguguacuucagu..	6	0	seq
....gagcGcacuugguagcgg............................	1	1	seq
....gagcacacuugguagcgg............................	1	0	seq
....gagcGcacuugguagcggu...........................	2	1	seq
....gagcacacuugguagcggC...........................	10	1	seq
....gagcacacuugguagcggu...........................	105	0	seq
....gGgcacacuugguagcggu...........................	1	1	seq
....gaUcacacuugguagcggu...........................	1	1	seq
....gagcacacuugguagcgAu...........................	1	1	seq
....gagGacacuugguagcggu...........................	1	1	seq
....gUgcacacuugguagcggu...........................	4	1	seq
....gaUcacacuugguagcggug..........................	2	1	seq
....gagcacacuugguagcggGg..........................	2	1	seq
....gagcacacuugguagcgCug..........................	1	1	seq
....gagcGcacuugguagcggug..........................	1	1	seq
....gagcacacuugguagcgguU..........................	1	1	seq
....gagcacacuCgguagcggug..........................	1	1	seq
....Cagcacacuugguagcggug..........................	1	1	seq
....gagcacacuugguagcgguA..........................	11	1	seq
....Uagcacacuugguagcggug..........................	1	1	seq
....gGgcacacuugguagcggug..........................	1	1	seq

```
                    Mature                                           Star
cucaaggccguagcaugugccaucgccaccaaguguacuucaguggacauaugucguuuuuaaaucugagcacacuugguagcggugccgugagcuacucccauacaucgga
...................................................................gaAcacacuugguagcggug.........................    1    1    seq
...................................................................Aagcacacuugguagcggug.........................    1    1    seq
...................................................................gagGacacuugguagcggug.........................    2    1    seq
...................................................................gagcacacuugguagcgguAc........................    1    1    seq
...................................................................gagcacacuugUuagcggugcc.......................    1    1    seq
....................................................................agcacacuugguagcggu..........................    1    0    seq
```
