# Supplementary material for: Identification and Characterization of MicroRNAs from Longitudinal Muscle and Respiratory Tree in Sea Cucumber (Apostichopus japonicus) Using High-Throughput Sequencing
Source: PLoS One. 2015 Aug 5;10(8):e0134899. doi: 10.1371/journal.pone.0134899 (PMC4526669; doi:10.1371/journal.pone.0134899)
Supplement: S2 File — (ZIP) [file pone.0134899.s003.zip › S2 File/The secondary structures of the novel miRNAs in RPT/Scaffold280_975.pdf]

Provisional ID	:	Scaffold280_975
Score total	:	1.4
Score for star read(s)	:	-1.3
Score for read counts	:	0
Score for mfe	:	1.1
Score for randfold	:	1.6
Score for cons. seed	:	
Total read count	:	15771
Mature read count	:	15771
Loop read count	:	0
Star read count	:	0

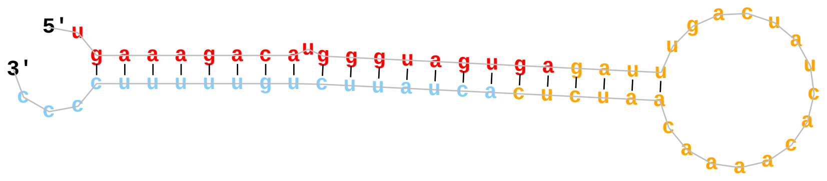

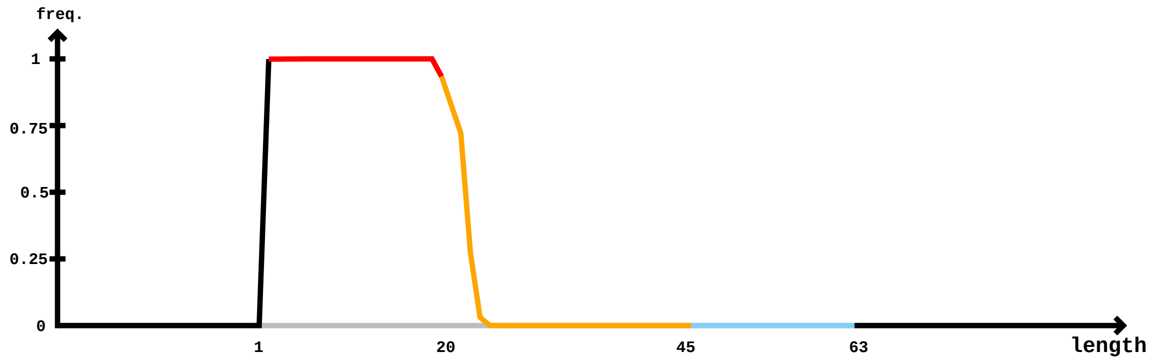

Mature **Star**

5'- cagaaucgguguucccugagugaaagacauggguagugagauuugacuaucacaaaacaaucucacuauucuguuuuuccccugguggauacuuauuacaucuacuacc -3' exp

...(((.((((((((.(.(.((((((((.((((((((((((..............)))))))))))))))))))))).).)).))))))).)))...........	reads	mm	sample
....................ugaaagacauUgguagug..	2	1	seq
....................ugaaagacaugggguGgug..	4	1	seq
....................ugaaagacauggguagCg..	3	1	seq
....................ugaaCgacauggguagug..	1	1	seq
....................ugaaagacaGggguagug..	1	1	seq
....................ugaaagacaugggAagug..	2	1	seq
....................ugaaagacaugggCagug..	4	1	seq
....................ugUaagacauggguagug..	3	1	seq
....................Ggaaagacauggguagug..	7	1	seq
....................ugaaagaUauggguagug..	2	1	seq
....................ugaaagacauggguagAg..	3	1	seq
....................ugaGagacauggguagug..	12	1	seq
....................ugaaagacauggguagGg..	1	1	seq
....................ugaaagacaAggguagug..	4	1	seq
....................ugaaagacauggguaguC..	1	1	seq
....................ugCaagacauggguagug..	1	1	seq
....................ugaaagacauggAuagug..	1	1	seq
....................ugaaGgacauggguagug..	5	1	seq
....................ugaaagacauggguaguA..	63	1	seq
....................Cgaaagacauggguagug..	4	1	seq
....................uCaaagacauggguagug..	2	1	seq
....................ugaaUgacauggguagug..	1	1	seq
....................uUaaagacauggguagug..	35	1	seq
....................ugaaagGcauggguagug..	1	1	seq
....................ugaaagacauggguaguU..	5	1	seq
....................uAaaagacauggguagug..	1	1	seq
....................ugGaagacauggguagug..	6	1	seq
....................ugaaagacauggguagug..	870	0	seq
....................ugaaagacauggguaAug..	1	1	seq
....................ugaaagacauggguaguAa...	3	1	seq
....................ugaGagacauggguaguga...	27	1	seq
....................ugaaagacauggguaguCa...	1	1	seq
....................ugaaagacauggguaguga...	2262	0	seq
....................ugaaagacauAgguaguga...	3	1	seq

```
                    Mature                                           Star
cagaaucgguguucccugagugaaagacauggguagugagauuugacuaucacaaaacaaucucacuauucuguuuuuccccugguggauacuuauuacaucuacc
....................ugaaagacaugAguaguga...................................................................   2     1   seq
....................ugaaagacauggguagugC...................................................................   6     1   seq
....................ugaaagacUuggguaguga...................................................................   1     1   seq
....................ugaaagacaAggguaguga...................................................................   1     1   seq
....................ugGaagacauggguaguga...................................................................   17    1   seq
....................ugaaagacauggguaguUa...................................................................   4     1   seq
....................ugaaagacauggguagugU...................................................................   74    1   seq
....................ugaUagacauggguaguga...................................................................   1     1   seq
....................ugaaagacaCgguaguga....................................................................   1     1   seq
....................ugaaagacGuggguaguga...................................................................   11    1   seq
....................ugaaagacaugggguUguga..................................................................   1     1   seq
....................ugaaagacaugggguGguga..................................................................   9     1   seq
....................uUaaagacauggguaguga...................................................................   109   1   seq
....................ugaaCgacauggguaguga...................................................................   1     1   seq
....................uAaaagacauggguaguga...................................................................   3     1   seq
....................ugaaagacaugggAaguga...................................................................   15    1   seq
....................ugaaagacaugggguaUuga..................................................................   2     1   seq
....................ugaaagacCuggguaguga...................................................................   14    1   seq
....................ugaaagacaugggCaguga...................................................................   6     1   seq
....................ugaaGgacauggguaguga...................................................................   19    1   seq
....................ugaaagacauggguagAga...................................................................   3     1   seq
....................ugaaagacauggguagGga...................................................................   5     1   seq
....................ugaaUgacauggguaguga...................................................................   2     1   seq
....................ugaCagacauggguaguga...................................................................   1     1   seq
....................ugaaagacaugUguaguga...................................................................   1     1   seq
....................ugaaagGcauggguaguga...................................................................   13    1   seq
....................Ggaaagacauggguaguga...................................................................   11    1   seq
....................ugaaagacaGgguaguga....................................................................   3     1   seq
....................ugaaagacauggguagugG...................................................................   15    1   seq
....................ugUaagacauggguaguga...................................................................   7     1   seq
....................uCaaagacauggguaguga...................................................................   8     1   seq
....................ugaaagacauggAuaguga...................................................................   2     1   seq
....................ugaaagacauggguagCga...................................................................   6     1   seq
....................Cgaaagacauggguaguga...................................................................   12    1   seq
....................ugaaagacauggguaguUag..................................................................   4     1   seq
....................ugaaagacaugggguaUugag.................................................................   1     1   seq
....................Cgaaagacauggguagugag..................................................................   17    1   seq
....................ugaaagaAauggguagugag..................................................................   3     1   seq
....................ugaaagacaugggAagugag..................................................................   21    1   seq
....................ugaaagacaugAguagugag..................................................................   3     1   seq
....................ugaaagacaugggCagugag..................................................................   23    1   seq
....................ugaaagacaugggguGgugag.................................................................   10    1   seq
....................ugaaagacCuggguagugag..................................................................   10    1   seq
....................ugaaagacauggguagGgag..................................................................   4     1   seq
....................ugaaagacaugggguUgugag.................................................................   2     1   seq
....................ugaaagacauggguagugaU..................................................................   75    1   seq
....................Ggaaagacauggguagugag..................................................................   13    1   seq
....................ugaaagacauggguagugUg..................................................................   5     1   seq
....................uAaaagacauggguagugag..................................................................   1     1   seq
....................ugaaagacauggguaguAag..................................................................   6     1   seq
....................ugaGagacauggguagugag..................................................................   55    1   seq
....................ugaaagacauggUuagugag..................................................................   1     1   seq
....................ugaaagacaugggGagugag..................................................................   6     1   seq
....................ugaaagacauggguagugCg..................................................................   1     1   seq
....................ugaaagacGuggguagugag..................................................................   15    1   seq
....................ugaaagacauggCuagugag..................................................................   1     1   seq
....................ugaaagacaugggguaAugag.................................................................   2     1   seq
....................ugaaagCcauggguagugag..................................................................   1     1   seq
....................ugaaagacauggguagugaC..................................................................   6     1   seq
....................ugaaagacauggguagCgag..................................................................   16    1   seq
....................ugaaagacauggguagugaA..................................................................   117   1   seq
....................ugaaagGcauggguagugag..................................................................   24    1   seq
....................uUaaagacauggguagugag..................................................................   106   1   seq
....................ugaaagaUauggguagugag..................................................................   3     1   seq
....................ugaaagacauggguaguCag..................................................................   1     1   seq
....................ugaaaUacauggguagugag..................................................................   1     1   seq
....................ugaaagacUuggguagugag..................................................................   2     1   seq
....................ugaaGgacauggguagugag..................................................................   22    1   seq
....................ugaaagacaGgguagugag...................................................................   2     1   seq
....................ugaaagacaugUguagugag..................................................................   5     1   seq
```

```
                   Mature                    Star
cagaaucgguguucccugagugaaagacauggguagugagauuugacuaucacaaaacaaucucacuauucuguuuuuccccugguggauacuuauuacaucuacuacc
....................ugUaagacauggguagugag.....................................................................	12	1	seq
....................ugGaagacauggguagugag.....................................................................	26	1	seq
....................ugaaagacauggguagugGg.....................................................................	18	1	seq
....................ugaUagacauggguagugag.....................................................................	2	1	seq
....................ugaaagacauggguagAgag.....................................................................	3	1	seq
....................ugaaagacauAgguagugag.....................................................................	2	1	seq
....................ugaaagacauggAuagugag.....................................................................	2	1	seq
....................uCaaagacauggguagugag.....................................................................	14	1	seq
....................ugaaagacauUgguagugag.....................................................................	4	1	seq
....................ugaaagacaugCguagugag.....................................................................	2	1	seq
....................ugaaUgacauggguagugag.....................................................................	1	1	seq
....................ugaaagaGauggguagugag.....................................................................	1	1	seq
....................ugaaUgacauggguagugaga....................................................................	33	1	seq
....................uUaaagacauggguagugaga....................................................................	1623	1	seq
....................ugaaagacauggguagGgaga....................................................................	30	1	seq
....................ugaaagacauggguaguAaga....................................................................	32	1	seq
....................ugaaagacauggUuagugaga....................................................................	13	1	seq
....................ugCaagacauggguagugaga....................................................................	6	1	seq
....................ugaaGgacauggguagugaga....................................................................	409	1	seq
....................ugaaaCacauggguagugaga....................................................................	5	1	seq
....................ugaaagacauggguaAugaga....................................................................	49	1	seq
....................ugaaaUacauggguagugaga....................................................................	23	1	seq
....................ugaaagacauggAuagugaga....................................................................	44	1	seq
....................ugaaagacauggguagAgaga....................................................................	25	1	seq
....................ugaaagacaugggu Ugugaga...................................................................	19	1	seq
....................ugaaagacaugggAagugaga....................................................................	366	1	seq
....................ugUaagacauggguagugaga....................................................................	247	1	seq
....................ugGaagacauggguagugaga....................................................................	291	1	seq
....................ugaaagGcauggguagugaga....................................................................	333	1	seq
....................ugaaagacauggguagugCga....................................................................	11	1	seq
....................ugaaagacauggguaguCaga....................................................................	8	1	seq
....................ugaUagacauggguagugaga....................................................................	61	1	seq
....................ugaaagacaGgguagugaga.....................................................................	38	1	seq
....................ugaaagacaugggu Ggugaga...................................................................	207	1	seq
....................ugaaagacauCgguagugaga....................................................................	7	1	seq
....................ugaaagacauggguaguUaga....................................................................	45	1	seq
....................ugaaagacauggguagugGga....................................................................	329	1	seq
....................ugaaagacaugggCagugaga....................................................................	310	1	seq
....................ugaaagacauggguagugaAa....................................................................	32	1	seq
....................ugaaagacauAgguagugaga....................................................................	93	1	seq
....................ugaaagacauUgguagugaga....................................................................	92	1	seq
....................ugaaagaAauggguagugaga....................................................................	5	1	seq
....................ugaaagacGuggguagugaga....................................................................	302	1	seq
....................ugaaagUcauggguagugaga....................................................................	12	1	seq
....................ugaaagacUuggguagugaga....................................................................	26	1	seq
....................ugaaaAacauggguagugaga....................................................................	16	1	seq
....................ugaaagacaugUguagugaga....................................................................	25	1	seq
....................ugaaagacauggguagugaUa....................................................................	53	1	seq
....................ugaaagaGauggguagugaga....................................................................	13	1	seq
....................ugaaagacauggguaUugaga....................................................................	20	1	seq
....................ugaaagacaugCguagugaga....................................................................	10	1	seq
....................ugaaagacCuggguagugaga....................................................................	185	1	seq
....................ugaGagacauggguagugaga....................................................................	801	1	seq
....................ugaaagacaugAguagugaga....................................................................	89	1	seq
....................ugaaagacauggguaCugaga....................................................................	5	1	seq
....................uCaaagacauggguagugaga....................................................................	156	1	seq
....................ugaCagacauggguagugaga....................................................................	8	1	seq
....................ugaaagacauggguagugaCa....................................................................	7	1	seq
....................ugaaagacauggguagCgaga....................................................................	164	1	seq
....................ugaaagaUauggguagugaga....................................................................	36	1	seq
....................ugaaagacauggCuagugaga....................................................................	6	1	seq
....................ugaaagCcauggguagugaga....................................................................	7	1	seq
....................ugaaagacauggguagugUga....................................................................	43	1	seq
....................uAaaagacauggguagugaga....................................................................	103	1	seq
....................ugaaagacaugggu Cgugaga...................................................................	7	1	seq
....................ugaaCgacauggguagugaga....................................................................	24	1	seq
....................ugaaagacaugggGagugaga....................................................................	150	1	seq
....................ugaaagacaugggu Ugugagau..................................................................	6	1	seq
....................ugaaUgacauggguagugagau...................................................................	20	1	seq
....................ugaaagacaGgguagugagau....................................................................	28	1	seq
```

Mature **Star**

cagaaucgguguucccugagugaaagacauggguagugagauuugacuaucacaaaacaaucucacuauucuguuuuuccccugguggauacuuauuacaucuacuacc			
....................ugaaagacauggAuagugagau...	30	1	seq
....................ugaaagacaugCguagugagau...	7	1	seq
....................ugaaaAacauggguagugagau...	17	1	seq
....................ugaaagGcauggguagugagau...	265	1	seq
....................ugaaagacauggUuagugagau...	7	1	seq
....................ugaaagacauUgguagugagau...	52	1	seq
....................ugaGagacauggguagugagau...	447	1	seq
....................ugaaaCacauggguagugagau...	9	1	seq
....................ugCaagacauggguagugagau...	3	1	seq
....................ugaaagacauCgguagugagau...	1	1	seq
....................ugaaagacauggguagugGgau...	242	1	seq
....................ugaaaUacauggguagugagau...	13	1	seq
....................ugaaagCcauggguagugagau...	9	1	seq
....................ugaaagacaugUguagugagau...	31	1	seq
....................ugaaagacauggCuagugagau...	6	1	seq
....................ugaaagacauggguagugaCau...	2	1	seq
....................ugaaagacauggguaguAagau...	29	1	seq
....................ugaaagacauggguGgugagau...	140	1	seq
....................ugGaagacauggguagugagau...	223	1	seq
....................ugaaagacaugAguagugagau...	67	1	seq
....................ugaaagacauggguagugaUau...	41	1	seq
....................ugaaagacauggguCgugagau...	7	1	seq
....................ugaaagacauggguagugCgau...	10	1	seq
....................ugaaagacauggguagCgagau...	140	1	seq
....................ugaaagacauAgguagugagau...	74	1	seq
....................ugaaagacauggguaUugagau...	8	1	seq
....................ugaaGgacauggguagugagau...	270	1	seq
....................ugaaagacCuggguagugagau...	141	1	seq
....................ugaaagacauggguagugaAau...	22	1	seq
....................ugaaagacauggguaCugagau...	1	1	seq
....................ugaUagacauggguagugagau...	31	1	seq
....................ugaaagacauggguaguCagau...	2	1	seq
....................ugaaCgacauggguagugagau...	13	1	seq
....................ugaaagacauggguaAugagau...	36	1	seq
....................ugaaagacaugggCagugagau...	255	1	seq
....................ugUaagacauggguagugagau...	136	1	seq
....................ugaaagaUauggguagugagau...	26	1	seq
....................ugaaagacauggguagGgagau...	78	1	seq
....................ugaaagacGuggguagugagau...	219	1	seq
....................ugaaagaGauggguagugagau...	14	1	seq
....................ugaaagaAauggguagugagau...	5	1	seq
....................ugaaagacaugggGagugagau...	174	1	seq
....................ugaaagacaugggAagugagau...	308	1	seq
....................ugaaagacUuggguagugagau...	24	1	seq
....................ugaaagacauggguagugUgau...	19	1	seq
....................ugaaagacauggguaguUagau...	35	1	seq
....................ugaCagacauggguagugagau...	6	1	seq
....................ugaaagacauggguagAgagau...	51	1	seq
....................ugaaagUcauggguagugagau...	14	1	seq
....................ugaaagGcauggguagugagauu..	50	1	seq
....................ugaaagacauggguaUugagauu..	5	1	seq
....................ugaaagacaugAguagugagauu..	13	1	seq
....................ugaaagacauAgguagugagauu..	11	1	seq
....................ugaaagacauggCuagugagauu..	1	1	seq
....................ugaaagacauggUuagugagauu..	1	1	seq
....................ugaaagacauggguaAugagauu..	8	1	seq
....................ugaaagaUauggguagugagauu..	6	1	seq
....................ugaaagUcauggguagugagauu..	1	1	seq
....................ugaaagaGauggguagugagauu..	1	1	seq
....................ugaaagacUuggguagugagauu..	3	1	seq
....................ugaaagacaugggAagugagauu..	72	1	seq
....................ugaaagacauggguGgugagauu..	37	1	seq
....................ugaaagacaugggCagugagauu..	52	1	seq
....................ugaaagacauggguUgugagauu..	7	1	seq
....................ugaaagacauggguagCgagauu..	35	1	seq
....................ugaaagacauUgguagugagauu..	15	1	seq
....................ugaaagCcauggguagugagauu..	2	1	seq
....................ugaaagacauggguCgugagauu..	3	1	seq
....................ugaaagacaugUguagugagauu..	7	1	seq
....................ugaaagacauggAuagugagauu..	8	1	seq

Mature Star

cagaaucgguguucccugagugaaagacauggguagugagauuugacuaucacaaaacaaucucacuauucuguuuuuccccugguggauacuuauuacaucuacuacc			
....................ugaaagacauggguagGgagauu..	13	1	seq
....................ugaaagacGuggguagugagauu..	39	1	seq
....................ugaaagacaugCguagugagauu..	1	1	seq
....................ugaaagaAauggguagugagauu..	1	1	seq
....................ugaaagacauggguagAgagauu..	5	1	seq
....................ugaaagacCuggguagugagauu..	32	1	seq
....................ugaaagacaGgguagugagauu..	3	1	seq
....................ugaaagacaugggGagugagauu..	46	1	seq
....................ugaaagacaugggCagugagauuu...	2	1	seq
....................ugaaagacCuggguagugagauuu...	1	1	seq
....................ugaaagacauUgguagugagauuu...	1	1	seq
....................ugaaagGcauggguagugagauuu...	1	1	seq
....................ugaaagacaugggAagugagauuu...	2	1	seq
....................ugaaagUcauggguagugagauuu...	1	1	seq
....................ugaaagacGuggguagugagauuu...	1	1	seq
....................ugaaagacauggguagCgagauuu...	1	1	seq
....................ugaaagacauggguagAgagauuu...	1	1	seq
.....................Uaaagacauggguagugaga..	1	1	seq
.....................gUaagacauggguagugagau...	1	1	seq
......................aaagacauggguagugaga..	2	0	seq
......................aaagacaugggAagugaga..	1	1	seq
......................aaaUacauggguagugagau...	2	1	seq
.......................aagacauggguagugaga..	3	0	seq
........................agacauggguagugagaC...	1	1	seq
........................agacauggguagugagau...	2	0	seq
........................agacauggguagugagauu..	1	0	seq
