# Supplementary material for: Identification and Characterization of MicroRNAs from Longitudinal Muscle and Respiratory Tree in Sea Cucumber (Apostichopus japonicus) Using High-Throughput Sequencing
Source: PLoS One. 2015 Aug 5;10(8):e0134899. doi: 10.1371/journal.pone.0134899 (PMC4526669; doi:10.1371/journal.pone.0134899)
Supplement: S2 File — (ZIP) [file pone.0134899.s003.zip › S2 File/The secondary structures of the novel miRNAs in RPT/Scaffold285_985.pdf]

Provisional ID	:	Scaffold285_985
Score total	:	1.4
Score for star read(s)	:	-1.3
Score for read counts	:	0
Score for mfe	:	1.1
Score for randfold	:	1.6
Score for cons. seed	:	
Total read count	:	27
Mature read count	:	27
Loop read count	:	0
Star read count	:	0

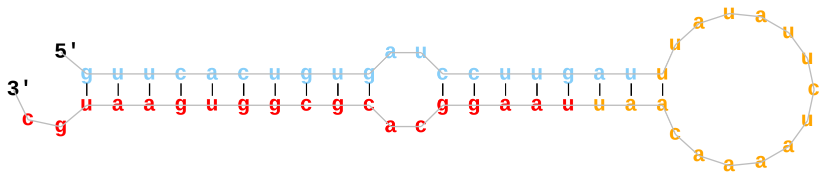

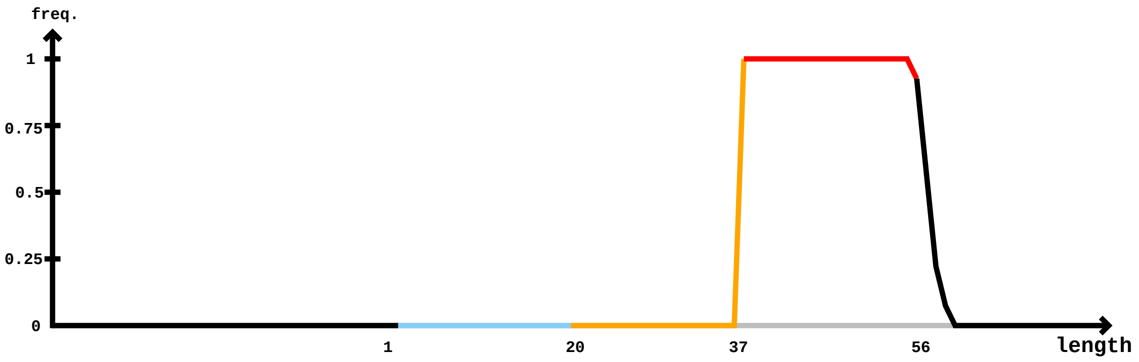


```
                                                Star                                        Mature
5'- uucgcugcuucuucuuauccaucccguucuuaguguucacugugauccuugauuuauauucuaaaacaauuaaggcacgcggugaaugccaaggcucgagggugaaau   -3'    exp
    ...................((((((.((((.(((((((((((((..((((((((.............))))))))..)))))))))))).))))..)).)))))....    reads    mm        sample
    ......................................................................uaaggcacgcggugaaug....................    2        0         seq
    ......................................................................uaaggcacgcggCgaaugc...................    1        1         seq
    ......................................................................uaaggcacgcggugaaugU...................    1        1         seq
    ......................................................................uaaggcacgcggugaaugc...................    13       0         seq
    ......................................................................uaaUgcacgcggugaaugc...................    1        1         seq
    ......................................................................Gaaggcacgcggugaaugcc..................    1        1         seq
    ......................................................................uaaUgcacgcggugaaugcc..................    1        1         seq
    ......................................................................uaaggcacgcggCgaaugcc..................    1        1         seq
    ......................................................................uaaUgcacgcggugaaugcca.................    3        1         seq
    ......................................................................uaaggcacgcggugGaugcca.................    1        1         seq
    ......................................................................uaaUgcacgcggugaaugccaa................    2        1         seq
```
