# Supplementary material for: Identification and Characterization of MicroRNAs from Longitudinal Muscle and Respiratory Tree in Sea Cucumber (Apostichopus japonicus) Using High-Throughput Sequencing
Source: PLoS One. 2015 Aug 5;10(8):e0134899. doi: 10.1371/journal.pone.0134899 (PMC4526669; doi:10.1371/journal.pone.0134899)
Supplement: S2 File — (ZIP) [file pone.0134899.s003.zip › S2 File/The secondary structures of the novel miRNAs in RPT/Scaffold299_1022.pdf]

Provisional ID : Scaffold299_1022
Score total : 2
Score for star read(s) : -1.3
Score for read counts : 0
Score for mfe : 1.7
Score for randfold : 1.6
Score for cons. seed :
Total read count : 181
Mature read count : 181
Loop read count : 0
Star read count : 0

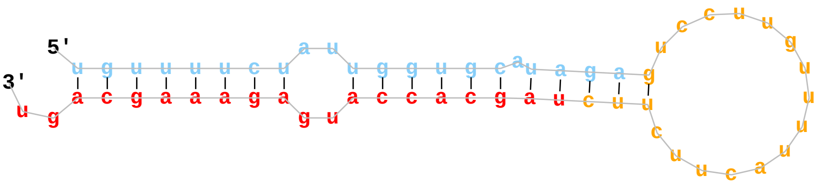

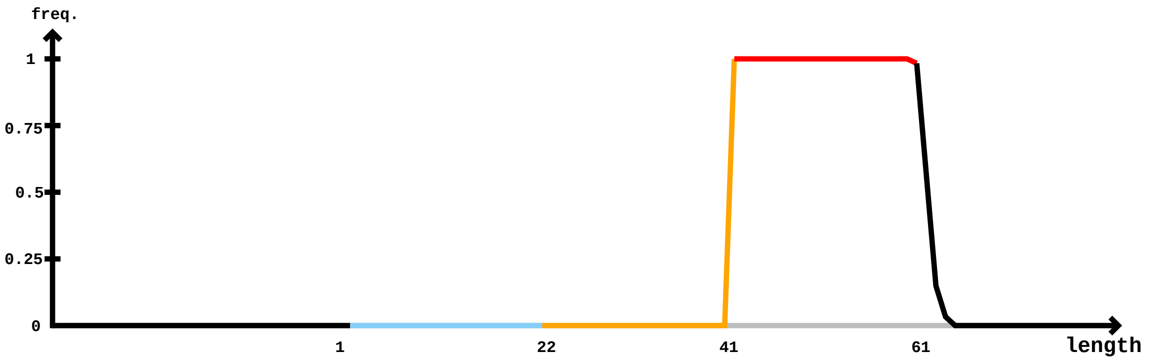

Star Mature

```
5'- ggcaagcaaagagugguaaucacaucuacuguuuucuauuggugcauagaguccuuguuuuacuucuucuagcaccaugagaaagcaguauuugugagacaccccaauc   -3'    exp
    ............(((....(((((..(((((((((((..((((((.(((((...............)))))))))))..)))))))))))..)))))..))).......     reads      mm      sample
    .....................................................................uagcaccaugagaaagcag.....................     3          0       seq
    .....................................................................uagcaccaugagGaagcagu....................     5          1       seq
    .....................................................................uagcaccaugagaaaUcagu....................     1          1       seq
    .....................................................................uagcaccauAagaaagcagu....................     2          1       seq
    .....................................................................uagcaccaugagaaagcagG....................     4          1       seq
    .....................................................................uagUaccaugagaaagcagu....................     2          1       seq
    .....................................................................uagcaccaugagaaUgcagu....................     1          1       seq
    .....................................................................uagcaccaCgagaaagcagu....................     1          1       seq
    .....................................................................Cagcaccaugagaaagcagu....................     2          1       seq
    .....................................................................uagcCccaugagaaagcagu....................     1          1       seq
    .....................................................................uaUcaccaugagaaagcagu....................     14         1       seq
    .....................................................................uagcGccaugagaaagcagu....................     1          1       seq
    .....................................................................uagcaccaugGgaaagcagu....................     4          1       seq
    .....................................................................uagcaccaugagaCagcagu....................     1          1       seq
    .....................................................................uagcacUaugagaaagcagu....................     2          1       seq
    .....................................................................uagcacGaugagaaagcagu....................     1          1       seq
    .....................................................................uagcaccaugagaaagcGgu....................     2          1       seq
    .....................................................................uagcaccaugagaaagcagC....................     36         1       seq
    .....................................................................uagGaccaugagaaagcagu....................     6          1       seq
    .....................................................................uagcaccaugagaaGgcagu....................     1          1       seq
    .....................................................................Gagcaccaugagaaagcagu....................     4          1       seq
    .....................................................................uagcaccaugagaaagcagA....................     1          1       seq
    .....................................................................uUgcaccaugagaaagcagu....................     2          1       seq
    .....................................................................uGgcaccaugagaaagcagu....................     3          1       seq
    .....................................................................uagcaccaugagaaaAcagu....................     1          1       seq
    .....................................................................uagcaAcaugagaaagcagu....................     1          1       seq
    .....................................................................uagcaccGugagaaagcagu....................     3          1       seq
    .....................................................................uagcaccaugagaaagcagGa...................     4          1       seq
    .....................................................................uagcaccaugagaaagcUgua...................     1          1       seq
    .....................................................................uaUcaccaugagaaagcagua...................     7          1       seq
    .....................................................................uagcaccaCgagaaagcagua...................     3          1       seq
    .....................................................................uagcaccaugagaaagcGgua...................     4          1       seq
    .....................................................................uagcaccCugagaaagcagua...................     1          1       seq
    .....................................................................uaAcaccaugagaaagcagua...................     3          1       seq
```

Star Mature

```
ggcaagcaaagagugguaaucacaucuacuguuuucuauuggugcauagaguccuuguuuuacuucuucuagcaccaugagaaagcaguauuugugagacaccccaauc
.....................................................................uagcaccGugagaaagcagua...................	1	1	seq
.....................................................................uagcaccaugagaaagcagCa...................	2	1	seq
.....................................................................uagGaccaugagaaagcagua...................	5	1	seq
.....................................................................uagcaccaugGgaaagcagua...................	1	1	seq
.....................................................................uagcaccaugagaGagcagua...................	1	1	seq
.....................................................................uagcaccaugagGaagcagua...................	2	1	seq
.....................................................................uagUaccaugagaaagcagua...................	4	1	seq
.....................................................................uagcacUaugagaaagcagua...................	1	1	seq
.....................................................................uUgcaccaugagaaagcagua...................	1	1	seq
.....................................................................uagcGccaugagaaagcagua...................	2	1	seq
.....................................................................uagcCccaugagaaagcagua...................	1	1	seq
.....................................................................uGgcaccaugagaaagcagua...................	2	1	seq
.....................................................................uagcaccaugagaaagcaUua...................	1	1	seq
.....................................................................uagcaccaugagaaGgcagua...................	2	1	seq
.....................................................................uagcaccaCgagaaagcaguau..................	3	1	seq
.....................................................................uagcaccaugagaaaCcaguau..................	1	1	seq
.....................................................................uagcaccaugagaaaUcaguau..................	1	1	seq
.....................................................................uagcaccaugagaaagcGguau..................	1	1	seq
.....................................................................uagcaccUugagaaagcaguau..................	2	1	seq
.....................................................................uagcaccaugagaaagcagCau..................	1	1	seq
.....................................................................uagcaccaugagaaGgcaguau..................	1	1	seq
.....................................................................uagcaccaAgagaaagcaguau..................	1	1	seq
.....................................................................uagcaccaugagaGagcaguau..................	1	1	seq
.....................................................................uagGaccaugagaaagcaguau..................	6	1	seq
.....................................................................uaUcaccaugagaaagcaguau..................	3	1	seq
.....................................................................uagcaccaugagGaagcaguauu.................	3	1	seq
.....................................................................uaUcaccaugagaaagcaguauu.................	2	1	seq
.....................................................................uagGaccaugagaaagcaguauu.................	1	1	seq
```
