# Supplementary material for: Identification and Characterization of MicroRNAs from Longitudinal Muscle and Respiratory Tree in Sea Cucumber (Apostichopus japonicus) Using High-Throughput Sequencing
Source: PLoS One. 2015 Aug 5;10(8):e0134899. doi: 10.1371/journal.pone.0134899 (PMC4526669; doi:10.1371/journal.pone.0134899)
Supplement: S2 File — (ZIP) [file pone.0134899.s003.zip › S2 File/The secondary structures of the novel miRNAs in RPT/Scaffold360_1160.pdf]

Provisional ID : Scaffold360_1160
Score total : 212.7
Score for star read(s) : 3.9
Score for read counts : 205.1
Score for mfe : 2.1
Score for randfold : 1.6
Score for cons. seed :
Total read count : 414
Mature read count : 412
Loop read count : 0
Star read count : 2

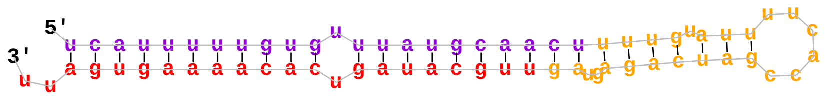

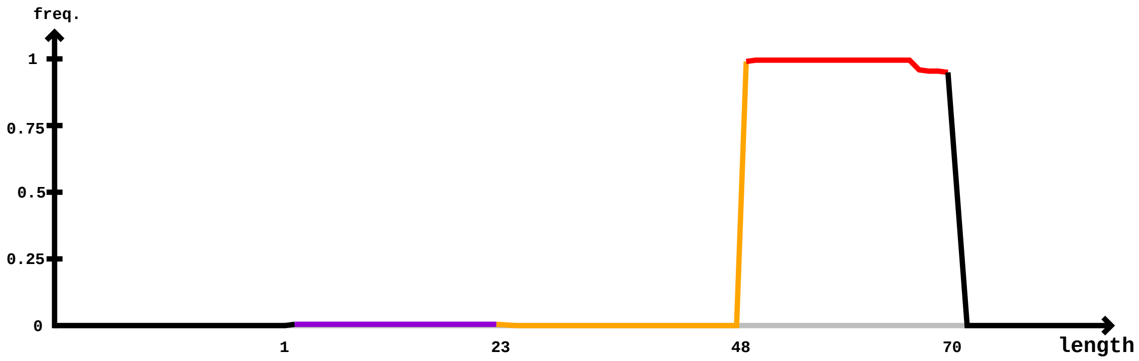

Star Mature

```
5'- auccaucauucccgccagcccgaucauuuuuguguuuaugcaacuuuuguauuuucaccgaucagaguaguugcauagucacaaaagugauugggaggguugggcucu   -3'   obs
    auccaucauucccgccagcccgaucauuuuuguguuuaugcaacuuuuguauuuucaccgaucagaguaguugcauagucacaaaagugauugggaggguugggcucu         exp
    ..........((((((..((((((((((((((((.((((((((((((((.(((......)))))))..)))))))))).))))))))))))))))..)).))))....   reads   mm   sample
    .......................ucauuuuuguguuuaGgcaacu...............................................................   2       1    seq
    ......................................................................uugcauagucacaaaagC....................   1       1    seq
    ......................................................................uugGauagucacaaaagu....................   1       1    seq
    ......................................................................uugcauagucacaaaagu....................   12      0    seq
    ......................................................................uCgcauagucacaaaagu....................   1       1    seq
    ......................................................................uugcauagucacaaaagug...................   2       0    seq
    ......................................................................uugcauaguUacaaaagugau.................   2       1    seq
    ......................................................................uuAcauagucacaaaagugauu................   8       1    seq
    ......................................................................uugcauagAcacaaaagugauu................   6       1    seq
    ......................................................................uugcauagucacaaaagCgauu................   21      1    seq
    ......................................................................uugcauagucacaaaagugGuu................   19      1    seq
    ......................................................................uugcauagucacaCaagugauu................   7       1    seq
    ......................................................................uugcaCagucacaaaagugauu................   5       1    seq
    ......................................................................uugcGuagucacaaaagugauu................   22      1    seq
    ......................................................................uugcauagucGcaaaagugauu................   23      1    seq
    ......................................................................uugcauagucacaaaCgugauu................   4       1    seq
    ......................................................................uugcauagucacUaaagugauu................   1       1    seq
    ......................................................................uugcauagucacGaaagugauu................   17      1    seq
    ......................................................................uugcauagucaUaaaagugauu................   3       1    seq
    ......................................................................uugcauagucacaaaagAgauu................   17      1    seq
    ......................................................................uugcauaUucacaaaagugauu................   1       1    seq
    ......................................................................uugcauUgucacaaaagugauu................   2       1    seq
    ......................................................................uuUcauagucacaaaagugauu................   49      1    seq
    ......................................................................uugcauagucCcaaaagugauu................   2       1    seq
    ......................................................................uugcauagucaAaaaagugauu................   2       1    seq
    ......................................................................uugcauagucacaaaGgugauu................   15      1    seq
    ......................................................................uugcauagGcacaaaagugauu................   2       1    seq
    ......................................................................uugcauagucacaaCagugauu................   1       1    seq
    ......................................................................uugcauaAucacaaaagugauu................   2       1    seq
    ......................................................................uugcauagucacaaaagugUuu................   3       1    seq
    ......................................................................uugGauagucacaaaagugauu................   24      1    seq
    ......................................................................uuCcauagucacaaaagugauu................   2       1    seq
    ......................................................................uugcauagucacaaaagGgauu................   17      1    seq
```

```
                       Star                                          Mature
auccaucauucccgccagcccgaucauuuuuguguuuaugcaacuuuuguauuuucaccgaucagaguaguugcauagucacaaaagugauugggaggguugggcucu
......................................................................uugcauaCucacaaaagugauu................    1    1    seq
......................................................................uugcauagucacaaaaguUauu................    2    1    seq
......................................................................uugcCuagucacaaaagugauu................    1    1    seq
......................................................................uugcauagucacaaUagugauu................    1    1    seq
......................................................................uugcauaguUacaaaagugauu................    10   1    seq
......................................................................uugcauGgucacaaaagugauu................    28   1    seq
......................................................................uugcauagucacaGaagugauu................    14   1    seq
......................................................................uugcauagCcacaaaagugauu................    22   1    seq
......................................................................uugcauaguAacaaaagugauu................    1    1    seq
......................................................................uugcauaguGacaaaagugauu................    2    1    seq
......................................................................uugcauagucacaaaUgugauu................    1    1    seq
......................................................................uugcaAagucacaaaagugauu................    2    1    seq
......................................................................uugUauagucacaaaagugauu................    7    1    seq
......................................................................uugcauagucacaaaaguAauu................    1    1    seq
......................................................................uugcauagucacaaaagugCuu................    3    1    seq
......................................................................uugcUuagucacaaaagugauu................    1    1    seq
......................................................................uugcauagucacaaGagugauu................    17   1    seq
......................................................................uugcauCgucacaaaagugauu................    2    1    seq
.......................................................................ugcauagucacaaaaguUauu................    1    1    seq
.......................................................................ugcGuagucacaaaagugauu................    1    1    seq
```
