# Supplementary material for: Identification and Characterization of MicroRNAs from Longitudinal Muscle and Respiratory Tree in Sea Cucumber (Apostichopus japonicus) Using High-Throughput Sequencing
Source: PLoS One. 2015 Aug 5;10(8):e0134899. doi: 10.1371/journal.pone.0134899 (PMC4526669; doi:10.1371/journal.pone.0134899)
Supplement: S2 File — (ZIP) [file pone.0134899.s003.zip › S2 File/The secondary structures of the novel miRNAs in RPT/Scaffold391_1235.pdf]

Provisional ID	:	Scaffold391_1235
Score total	:	2.2
Score for star read(s)	:	-1.3
Score for read counts	:	0
Score for mfe	:	1.9
Score for randfold	:	1.6
Score for cons. seed	:	
Total read count	:	5223
Mature read count	:	5223
Loop read count	:	0
Star read count	:	0

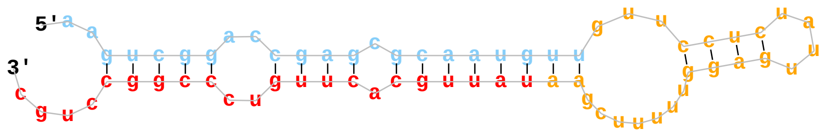

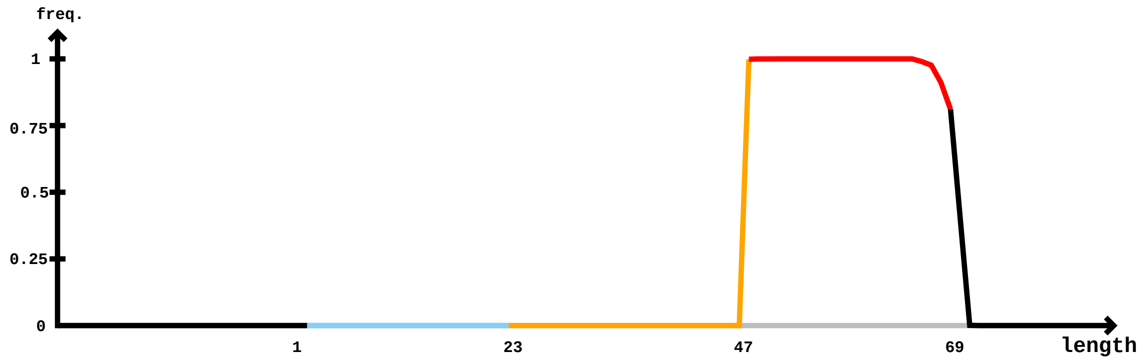

Star Mature

```
5'- ucaucaccauggugauugauggucaagucggaccgagcgcaauguuguuccucuauugagguuuuucgaauauugcacuugucccggccugcucaucaacaccaaaau   -3'    exp
    .........(((((.(((((((.((.(((((..((((.((((((((...((((....)))).......)))))))).))))..))))).)).))))))))))))....      reads   mm    sample
    ......................................................................uauugcacuugCcccggc....................      1       1     seq
    ......................................................................uauGgcacuugucccggc....................      2       1     seq
    ......................................................................uauugcacuugucccggU....................      3       1     seq
    ......................................................................uauugcacuugucccggc....................      29      0     seq
    ......................................................................uauugcacuugAcccggc....................      7       1     seq
    ......................................................................uauugcacuuUucccggc....................      6       1     seq
    ......................................................................uauCgcacuugucccggc....................      1       1     seq
    ......................................................................uauugcacuugucccggA....................      2       1     seq
    ......................................................................uaGugcacuugucccggc....................      1       1     seq
    ......................................................................uauugcacCugucccggcc...................      1       1     seq
    ......................................................................uauugcacuuUucccggcc...................      9       1     seq
    ......................................................................uauugcacuuCucccggcc...................      1       1     seq
    ......................................................................uauugcacuugCcccggcc...................      1       1     seq
    ......................................................................uauGgcacuugucccggcc...................      1       1     seq
    ......................................................................uauugcacuugucccAgcc...................      1       1     seq
    ......................................................................uauugcacuugucccggcU...................      12      1     seq
    ......................................................................uauugcacuugAcccggcc...................      12      1     seq
    ......................................................................uauugcacuugucccggcc...................      30      0     seq
    ......................................................................uauugcacuugucccggcA...................      5       1     seq
    ......................................................................uauugcacuuUucccggccu..................      91      1     seq
    ......................................................................uauugcacuuCucccggccu..................      1       1     seq
    ......................................................................uGuugcacuugucccggccu..................      1       1     seq
    ......................................................................uauugcacuuguUccggccu..................      1       1     seq
    ......................................................................uauugcacuugucccggccA..................      7       1     seq
    ......................................................................uauGgcacuugucccggccu..................      6       1     seq
    ......................................................................uUuugcacuugucccggccu..................      3       1     seq
    ......................................................................uaCugcacuugucccggccu..................      1       1     seq
    ......................................................................uauugcacuuguccAggccu..................      1       1     seq
    ......................................................................uauugcacuugCcccggccu..................      2       1     seq
    ......................................................................Gauugcacuugucccggccu..................      3       1     seq
    ......................................................................uaGugcacuugucccggccu..................      1       1     seq
    ......................................................................uauugcacuugucccggccG..................      37      1     seq
    ......................................................................uauugcacuugucccggcAu..................      1       1     seq
    ......................................................................uauugcacuugGcccggccu..................      1       1     seq
```

```
                        Star                                          Mature
ucaucaccauggugauugauggucaagucggaccgagcgcaauguuguucccucuauugagguuuuucgaauauugcacuugucccggccugcucaucaacaccaaaau
......................................................................uauugcacuugAcccggccu..................    165     1     seq
......................................................................uauugcacuugucUcggccu..................    2       1     seq
......................................................................uauugcacuugucccggccC..................    14      1     seq
......................................................................uaGugcacuugucccggccug.................    1       1     seq
......................................................................uauugcGcuugucccggccug.................    2       1     seq
......................................................................uauugcacuuUucccggccug.................    153     1     seq
......................................................................uauugcacuugCcccggccug.................    1       1     seq
......................................................................uauugcacuugucccggccGg.................    220     1     seq
......................................................................uauugcacuugGcccggccug.................    1       1     seq
......................................................................uauugcacuugAcccggccug.................    134     1     seq
......................................................................uauGgcacuugucccggccug.................    4       1     seq
......................................................................uaCugcacuugucccggccug.................    1       1     seq
......................................................................uauugcacuuAucccggccug.................    1       1     seq
......................................................................uauugcacuugucccggccAg.................    1       1     seq
......................................................................uauugcacuugucccggccCg.................    2       1     seq
......................................................................uUuugcacuugucccggccug.................    3       1     seq
......................................................................uauugcacuuguccAggccugc................    3       1     seq
......................................................................uaGugcacuugucccggccugc................    11      1     seq
......................................................................uauugcacuuAucccggccugc................    2       1     seq
......................................................................uauuAcacuugucccggccugc................    2       1     seq
......................................................................uauugcacuugCcccggccugc................    12      1     seq
......................................................................uauugcacuugGcccggccugc................    17      1     seq
......................................................................uauCgcacuugucccggccugc................    5       1     seq
......................................................................uauugcacuuCucccggccugc................    5       1     seq
......................................................................uauGgcacuugucccggccugc................    32      1     seq
......................................................................uauugcacuugucccggccCgc................    8       1     seq
......................................................................uauugcacuugAcccggccugc................    1588    1     seq
......................................................................uauugUacuugucccggccugc................    2       1     seq
......................................................................uauugcacuGgucccggccugc................    5       1     seq
......................................................................uauugcacuuUucccggccugc................    1938    1     seq
......................................................................uauugcGcuugucccggccugc................    9       1     seq
......................................................................uauugcacuugucccggGcugc................    1       1     seq
......................................................................uauugcacuugucUcggccugc................    7       1     seq
......................................................................uauugcacuugucccggccAgc................    2       1     seq
......................................................................uauugcacuuguccGggccugc................    3       1     seq
......................................................................uauugcacuuguGccggccugc................    1       1     seq
......................................................................uauugcacuugucccUgccugc................    1       1     seq
......................................................................uauugcacuugucccgAccugc................    1       1     seq
......................................................................uauAgcacuugucccggccugc................    1       1     seq
......................................................................uaAugcacuugucccggccugc................    6       1     seq
......................................................................uauugcaUuugucccggccugc................    4       1     seq
......................................................................uauugcacuuguAccggccugc................    2       1     seq
......................................................................uauUcacuugucccggccugc................    2       1     seq
......................................................................uauugcacuugucccggccGgc................    341     1     seq
......................................................................uauugcacuugucAcggccugc................    1       1     seq
......................................................................uauugcacAugucccggccugc................    1       1     seq
......................................................................uaCugcacuugucccggccugc................    6       1     seq
......................................................................uauugcacuuguccUggccugc................    2       1     seq
......................................................................uauugcacCugucccggccugc................    4       1     seq
......................................................................uauugcUcuugucccggccugc................    2       1     seq
......................................................................uauugcacuuUucccggccugcu...............    84      1     seq
......................................................................uauugcacuugucAcggccugcu...............    1       1     seq
......................................................................uauugcacuugucccggccGgcu...............    1       1     seq
......................................................................uauugcacuugAcccggccugcu...............    105     1     seq
......................................................................uauugcacuugGcccggccugcu...............    2       1     seq
......................................................................uauGgcacuugucccggccugcu...............    3       1     seq
......................................................................uauugcaUuugucccggccugcu...............    1       1     seq
......................................................................uauugcacuugCcccggccugcu...............    1       1     seq
......................................................................uauugcacuugAcccggccugcuc..............    3       1     seq
.......................................................................auugcacuuUucccggccugc................    2       1     seq
.......................................................................auugcacuugucccggccGgc................    1       1     seq
.......................................................................auugcacuugAcccggccugcu...............    2       1     seq
.......................................................................auCgcacuugucccggccugcu...............    1       1     seq
.......................................................................auuUcacuugucccggccugcuc..............    1       1     seq
.........................................................................ugcacuuUucccggccugc................    1       1     seq
```
