# Supplementary material for: Identification and Characterization of MicroRNAs from Longitudinal Muscle and Respiratory Tree in Sea Cucumber (Apostichopus japonicus) Using High-Throughput Sequencing
Source: PLoS One. 2015 Aug 5;10(8):e0134899. doi: 10.1371/journal.pone.0134899 (PMC4526669; doi:10.1371/journal.pone.0134899)
Supplement: S2 File — (ZIP) [file pone.0134899.s003.zip › S2 File/The secondary structures of the novel miRNAs in RPT/Scaffold391_1237.pdf]

Provisional ID	:	Scaffold391_1237
Score total	:	2.4
Score for star read(s)	:	-1.3
Score for read counts	:	0
Score for mfe	:	2.1
Score for randfold	:	1.6
Score for cons. seed	:	
Total read count	:	342
Mature read count	:	342
Loop read count	:	0
Star read count	:	0

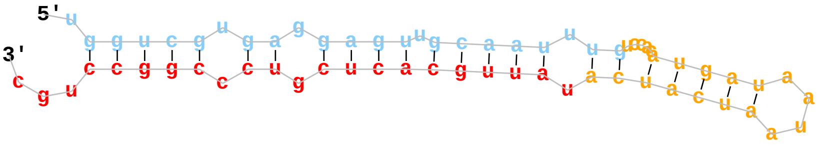

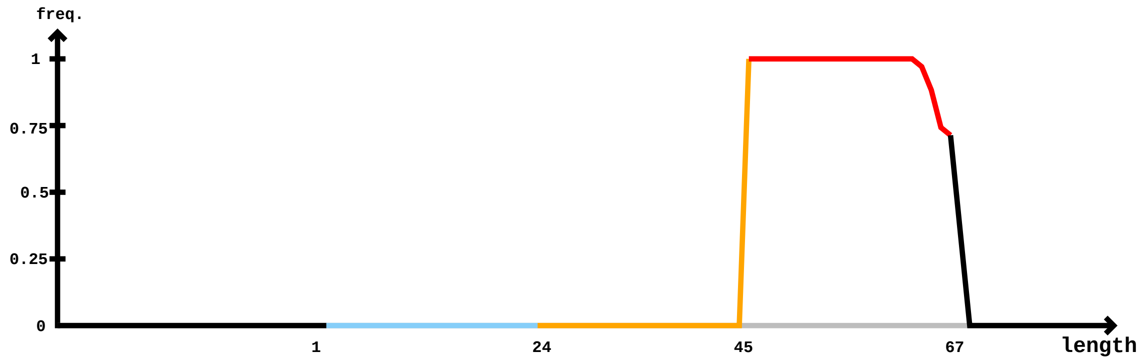

Star   Mature

```
5'- uccaucaucaguagagggcagcaagcuggucgugaggaguugcaauuuguccacaugauaauaaucaucauauugcacucgucccggccugccugcuugcccucaauc   -3'    exp
    ............((((((((((.((.(((((.((.((((.(((((.((.....(((((....))))))).)))))))))).)).))))).)).))).)))))))....   reads   mm   sample
    ......................................................................uauugcacucgucccggA....................   1       1    seq
    ......................................................................uauugcacucgucccggc....................   7       0    seq
    ......................................................................uauugcacucgucccggU....................   2       1    seq
    ......................................................................uauugcacucgucccggcA...................   1       1    seq
    ......................................................................uauGgcacucgucccggcc...................   1       1    seq
    ......................................................................uauugcacucUucccggcc...................   1       1    seq
    ......................................................................uauugcacucgucccggcU...................   9       1    seq
    ......................................................................uauugcacucgucccggcc...................   18      0    seq
    ......................................................................uauugcacucgucccggccG..................   1       1    seq
    ......................................................................uauGgcacucgucccggccu..................   5       1    seq
    ......................................................................uaGugcacucgucccggccu..................   4       1    seq
    ......................................................................uauugcGcucgucccggccu..................   1       1    seq
    ......................................................................uauugcacucgucccggccC..................   25      1    seq
    ......................................................................uauugcacucUucccggccu..................   1       1    seq
    ......................................................................Gauugcacucgucccggccu..................   1       1    seq
    ......................................................................uauugcacCcgucccggccu..................   1       1    seq
    ......................................................................uauugcacucgAcccggccu..................   1       1    seq
    ......................................................................uauugcacucgucUcggccu..................   1       1    seq
    ......................................................................uauugcacucgGcccggccu..................   1       1    seq
    ......................................................................uGuugcacucgucccggccu..................   4       1    seq
    ......................................................................uUuugcacucgucccggccu..................   2       1    seq
    ......................................................................uUuugcacucgucccggccug.................   2       1    seq
    ......................................................................uauGgcacucgucccggccug.................   1       1    seq
    ......................................................................uauCgcacucgucccggccug.................   2       1    seq
    ......................................................................uauugcacucguccGggccug.................   1       1    seq
    ......................................................................uauugcacucgucccggccGg.................   1       1    seq
    ......................................................................uauugcacucgucccggccCg.................   2       1    seq
    ......................................................................uGuugcacucgucccggccug.................   1       1    seq
    ......................................................................uauuCcacucgucccggccugc................   1       1    seq
    ......................................................................uauugcacucgucccAgccugc................   10      1    seq
    ......................................................................uauugcacucgucAcggccugc................   4       1    seq
    ......................................................................uauugUacucgucccggccugc................   6       1    seq
    ......................................................................uauugcacucgAcccggccugc................   8       1    seq
    ......................................................................uauugcacuGgucccggccugc................   5       1    seq
```

```
                               Star                                           Mature
uccaucaucaguagagggcagcaagcuggucgugaggaguugcaauuuguccacaugauaauaaucaucauauugcacucgucccggccugccugcuugcccucaauc
......................................................................uauugcacucgucccggGcugc................   1    1    seq
......................................................................uauugcacucgucccggAcugc................   1    1    seq
......................................................................uauugcacucgucccggccAgc................   2    1    seq
......................................................................uauugcacucgucccgCccugc................   1    1    seq
......................................................................uaAugcacucgucccggccugc................   10   1    seq
......................................................................uauugcacucgucccggUcugc................   7    1    seq
......................................................................uauugcacucguAccggccugc................   2    1    seq
......................................................................uauugcacucCucccggccugc................   1    1    seq
......................................................................uauugcaUucgucccggccugc................   7    1    seq
......................................................................uauugcacucgucccUgccugc................   1    1    seq
......................................................................uauugcacucguccUggccugc................   8    1    seq
......................................................................uauugcacucguccGggccugc................   9    1    seq
......................................................................uauugGacucgucccggccugc................   2    1    seq
......................................................................uauugcGcucgucccggccugc................   11   1    seq
......................................................................uauugcacucgucccggcUugc................   7    1    seq
......................................................................uauugcacAcgucccggccugc................   1    1    seq
......................................................................uauugcacucgucccgAccugc................   6    1    seq
......................................................................uaGugcacucgucccggccugc................   12   1    seq
......................................................................uauugcacucUucccggccugc................   14   1    seq
......................................................................uauugcacucgucccggccGgc................   4    1    seq
......................................................................uauugcacucgGcccggccugc................   9    1    seq
......................................................................uauuUcacucgucccggccugc................   1    1    seq
......................................................................uauugcacucgucccggccCgc................   13   1    seq
......................................................................uauugcacucguccAggccugc................   3    1    seq
......................................................................uauGgcacucgucccggccugc................   51   1    seq
......................................................................uauugcacucgucccCgccugc................   1    1    seq
......................................................................uauCgcacucgucccggccugc................   9    1    seq
......................................................................uauugcacucgucUcggccugc................   5    1    seq
......................................................................uauugcacucAucccggccugc................   8    1    seq
......................................................................uauugcacucguUccggccugc................   2    1    seq
......................................................................uauugcacucgucccggccGgcc...............   1    1    seq
```
