# Supplementary material for: Identification and Characterization of MicroRNAs from Longitudinal Muscle and Respiratory Tree in Sea Cucumber (Apostichopus japonicus) Using High-Throughput Sequencing
Source: PLoS One. 2015 Aug 5;10(8):e0134899. doi: 10.1371/journal.pone.0134899 (PMC4526669; doi:10.1371/journal.pone.0134899)
Supplement: S2 File — (ZIP) [file pone.0134899.s003.zip › S2 File/The secondary structures of the novel miRNAs in RPT/Scaffold391_1239.pdf]

Provisional ID : Scaffold391_1239
Score total : 1.9
Score for star read(s) : -1.3
Score for read counts : 0
Score for mfe : 1.6
Score for randfold : 1.6
Score for cons. seed :
Total read count : 529
Mature read count : 529
Loop read count : 0
Star read count : 0

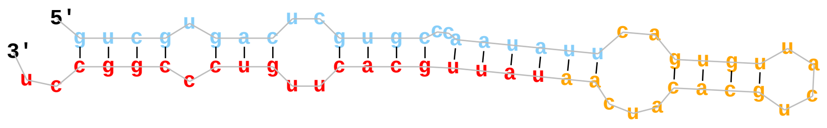

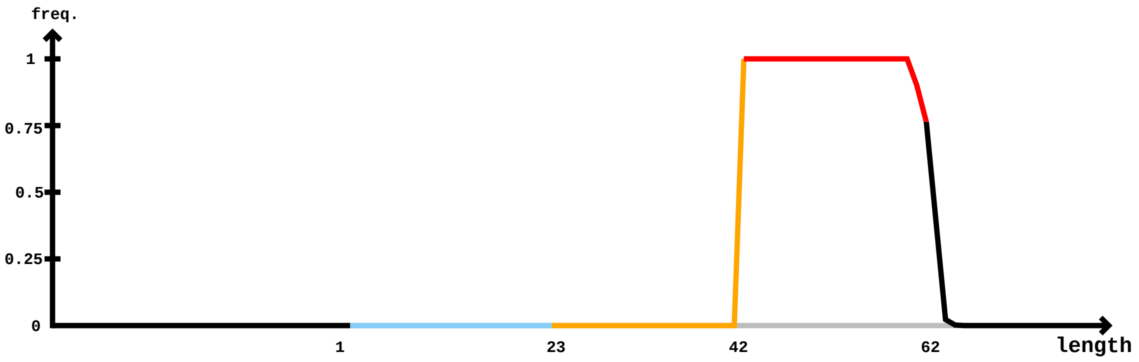

Star Mature

```
5'- agcuuuugucaacgaaucuugucuuguaggucgugacucgugcccaauauucaguguuacugcacaucaauauugcacuugucccggccuacuggauagguucuaaac   -3'   exp
    ............((((((.((((.((((((((.(((..((((..((((((..((((....))))...))))))))))..))).)))))))).)))))))))).....   reads   mm   sample
    ......................................................................uauugcacuuUucccggc....................   6    1   seq
    ......................................................................uauugcacuugucccggA....................   2    1   seq
    ......................................................................uauugcacuugAcccggc....................   7    1   seq
    ......................................................................uauugcacuugucccggU....................   3    1   seq
    ......................................................................uauGgcacuugucccggc....................   2    1   seq
    ......................................................................uauugcacuugucccggc....................   29   0   seq
    ......................................................................uauugcacuugCcccggc....................   1    1   seq
    ......................................................................uauCgcacuugucccggc....................   1    1   seq
    ......................................................................uaGugcacuugucccggc....................   1    1   seq
    ......................................................................uauugcacuugucccggcc...................   30   0   seq
    ......................................................................uauugcacuugCcccggcc...................   1    1   seq
    ......................................................................uauugcacCugucccggcc...................   1    1   seq
    ......................................................................uauugcacuuUucccggcc...................   9    1   seq
    ......................................................................uauugcacuugucccggcU...................   12   1   seq
    ......................................................................uauugcacuugucccggcA...................   5    1   seq
    ......................................................................uauugcacuuguccсAgcc...................   1    1   seq
    ......................................................................uauugcacuuCucccggcc...................   1    1   seq
    ......................................................................uauugcacuugAcccggcc...................   12   1   seq
    ......................................................................uauGgcacuugucccggcc...................   1    1   seq
    ......................................................................Gauugcacuugucccggccu..................   3    1   seq
    ......................................................................uauugcacuugCcccggccu..................   2    1   seq
    ......................................................................uauugcacuugGcccggccu..................   1    1   seq
    ......................................................................uauugcacuugucccggccC..................   14   1   seq
    ......................................................................uauugcacuugAcccggccu..................   165  1   seq
    ......................................................................uauugcacuuUucccggccu..................   91   1   seq
    ......................................................................uGuugcacuugucccggccu..................   1    1   seq
    ......................................................................uauugcacuugucccggccG..................   37   1   seq
    ......................................................................uUuugcacuugucccggccu..................   3    1   seq
    ......................................................................uauGgcacuugucccggccu..................   6    1   seq
    ......................................................................uauugcacuugucUcggccu..................   2    1   seq
    ......................................................................uauugcacuugucccggcAu..................   1    1   seq
    ......................................................................uauugcacuuguccAggccu..................   1    1   seq
    ......................................................................uaCugcacuugucccggccu..................   1    1   seq
    ......................................................................uauugcacuuCucccggccu..................   1    1   seq
```

```
                             Star                                        Mature
agcuuuugucaacgaaucuugucuuguaggucgugacucgugcccaauauucaguguuacugcacaucaauauugcacuugucccggccuacuggauagguucuaauc
......................................................................uauugcacuuguUccggccu..................   1    1    seq
......................................................................uaGugcacuugucccggccu..................   1    1    seq
......................................................................uauugcacuugucccggccA..................   7    1    seq
......................................................................uauugcacuuguccUggccua.................   1    1    seq
......................................................................uauugcacuugucccggccGa.................   19   1    seq
......................................................................uauugcGcuugucccggccua.................   1    1    seq
......................................................................uauugcacuuUucccggccua.................   15   1    seq
......................................................................uauugcacuugucccCgccua.................   1    1    seq
......................................................................uauugcacuugAcccggccua.................   11   1    seq
......................................................................uauugcacuuguccGggccua.................   1    1    seq
......................................................................uauugcacuugucccggccCa.................   1    1    seq
......................................................................uauAgcacuugucccggccua.................   1    1    seq
......................................................................uauGgcacuugucccggccua.................   2    1    seq
......................................................................uaGugcacuugucccggccua.................   1    1    seq
......................................................................uauugcacuugucccggccCac................   1    1    seq
......................................................................uauugcacuugAcccggccuac................   3    1    seq
......................................................................uauugcacuuAucccggccuac................   1    1    seq
......................................................................uauCgcacuugucccggccuac................   1    1    seq
......................................................................uauGgcacuugucccggccuac................   1    1    seq
......................................................................uauugcacuuguccUggccuac................   1    1    seq
......................................................................uaCugcacuugucccggccuac................   1    1    seq
......................................................................uauugcacuuUucccggccuac................   2    1    seq
......................................................................uauugcacuugAcccggccuacu...............   1    1    seq
```
