# Supplementary material for: Identification and Characterization of MicroRNAs from Longitudinal Muscle and Respiratory Tree in Sea Cucumber (Apostichopus japonicus) Using High-Throughput Sequencing
Source: PLoS One. 2015 Aug 5;10(8):e0134899. doi: 10.1371/journal.pone.0134899 (PMC4526669; doi:10.1371/journal.pone.0134899)
Supplement: S2 File — (ZIP) [file pone.0134899.s003.zip › S2 File/The secondary structures of the novel miRNAs in RPT/Scaffold391_1241.pdf]

Provisional ID	:	Scaffold391_1241
Score total	:	2.4
Score for star read(s)	:	-1.3
Score for read counts	:	0
Score for mfe	:	2.1
Score for randfold	:	1.6
Score for cons. seed	:	
Total read count	:	106
Mature read count	:	106
Loop read count	:	0
Star read count	:	0

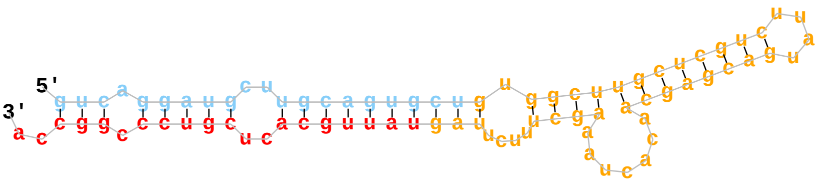

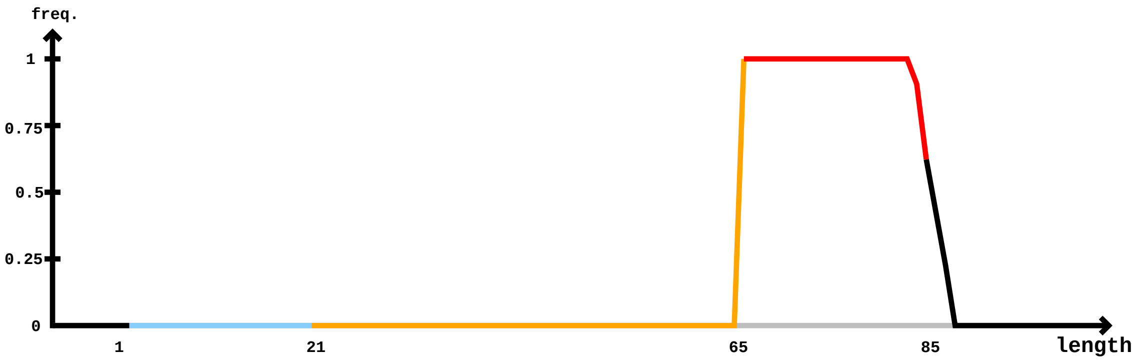

Star **Mature**

```
5'- gugcuggucaggaugcuugcagugcuguggcuugcucgucuuaugacgagcaacacuaaagcuuucuuaguauugcacucgucccggccaguaggguaucugguauaa -3'   exp
    .((((((((.(((((..((((((((((.((((((((((((....))))))))).......))))....))))))))))..))))).))))))))...............   reads   mm   sample
    ......................................................................uauugcacucgucccggU....................   2       1    seq
    ......................................................................uauugcacucgucccggc....................   7       0    seq
    ......................................................................uauugcacucgucccggA....................   1       1    seq
    ......................................................................uauugcacucgucccggcA...................   1       1    seq
    ......................................................................uauugcacucgucccggcc...................   18      0    seq
    ......................................................................uauGgcacucgucccggcc...................   1       1    seq
    ......................................................................uauugcacucUucccggcc...................   1       1    seq
    ......................................................................uauugcacucgucccggcU...................   9       1    seq
    ......................................................................uauugcacucgucccggccG..................   1       1    seq
    ......................................................................uauugcacucgucccggccC..................   25      1    seq
    ......................................................................uauugcacuUgucccggcca..................   7       1    seq
    ......................................................................uauugcacucgucccggcca..................   5       0    seq
    ......................................................................uauugcacucgucccggccGg.................   1       1    seq
    ......................................................................uauugcacucgucccggccCg.................   2       1    seq
    ......................................................................uauugcacuUgucccggccag.................   1       1    seq
    ......................................................................uauugcacucgucccggccCgu................   1       1    seq
    ......................................................................uauugcacuUgucccggccagu................   7       1    seq
    ......................................................................uauugcacucgucccggcAagu................   1       1    seq
    ......................................................................uauugcacucgucccggccagC................   2       1    seq
    ......................................................................uauugcacucgucccggccGgu................   13      1    seq
```
