# Supplementary material for: Identification and Characterization of MicroRNAs from Longitudinal Muscle and Respiratory Tree in Sea Cucumber (Apostichopus japonicus) Using High-Throughput Sequencing
Source: PLoS One. 2015 Aug 5;10(8):e0134899. doi: 10.1371/journal.pone.0134899 (PMC4526669; doi:10.1371/journal.pone.0134899)
Supplement: S2 File — (ZIP) [file pone.0134899.s003.zip › S2 File/The secondary structures of the novel miRNAs in RPT/Scaffold518_1542.pdf]

Provisional ID	:	Scaffold518_1542
Score total	:	61.5
Score for star read(s)	:	3.9
Score for read counts	:	59.8
Score for mfe	:	0
Score for randfold	:	-2.2
Score for cons. seed	:	
Total read count	:	129
Mature read count	:	121
Loop read count	:	0
Star read count	:	8

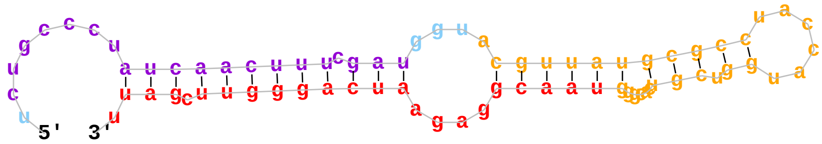

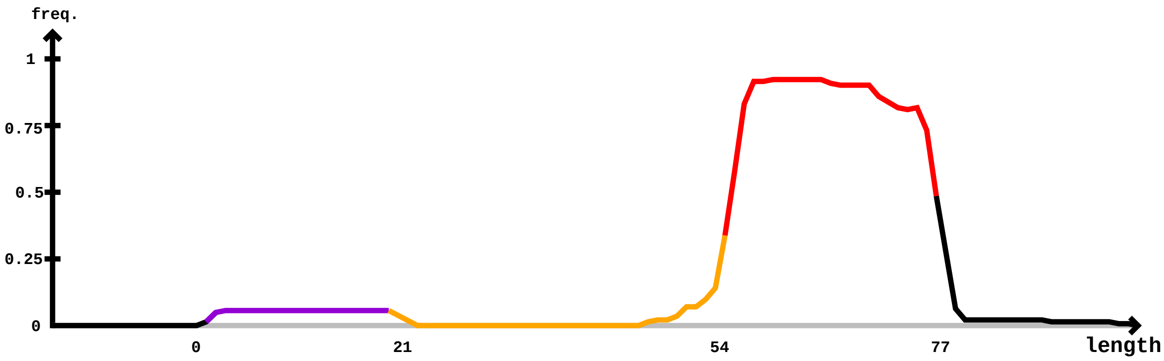

Star Mature

5'-	ggauccuucgaagucugcccuaucaacuuucgauggguacguuaugcgccuaccauggucgucacggguaacggagaaucaggguucgauuccggagagggagccugagaa	-3'	obs	
	ggauccuucgaaugucugcccuaucaacuuucgaugguacguuaugcgccuaccauggucgucacggguaacggagaaucaggguucgauuccggagagggagccugagaa		exp	
	((.((((((....(((.(((((......(((((.((...(((.(((.(((......)))))).)))..)).)))))....)))))..)))......)))))).))......	reads	mm	sample
	.............ucugcUcuaucaacuuucgau..	2	1	seq
	..............cugcUcuaucaacuuucgau..	3	1	seq
	..............cugcUcuaucaacuuucgaug...	2	1	seq
	...............ugcUcuaucaacuuucgau..	1	1	seq
	....guGacggguaacggagaau................................	1	1	seq
	....guUacggguaacggagaau................................	1	1	seq
	....uGacggguaacggagaauc...............................	1	1	seq
	....acggguaacggagaaucagg............................	1	0	seq
	....acgUguaacggagaaucagggu..........................	1	1	seq
	....cggguaacggagaaucaggA...........................	1	1	seq
	....cggguaacggagaaucaggg...........................	3	0	seq
	....cggUuaacggagaaucaggguucga......................	1	1	seq
	....gguaacggagaaucaggg...........................	2	0	seq
	....gguaacggGgaaucagggu..........................	2	1	seq
	....guaacggagaaucaggguu.........................	1	0	seq
	....guaacggagaaucaggguucgau.....................	2	0	seq
	....gGaacggagaaucaggguucgauu....................	1	1	seq
	....guaacggagaaucaggguucgauC....................	2	1	seq
	....uaacggagaaucaggguu.........................	2	0	seq
	....uaacggagaaucaggguuc........................	1	0	seq
	....uaacggagaaucaggguucgau.....................	3	0	seq
	....uGacggagaaucaggguucgauu....................	1	1	seq
	....uaUcggagaaucaggguucgauu....................	1	1	seq
	....uaacggagaaucaggguucgauu....................	20	0	seq
	....aacUgagaaucaggguucga......................	1	1	seq
	....aacggagaaucaggguucga......................	8	0	seq
	....aacggagaaucaggguucgau.....................	12	0	seq
	....aacggagaaucagggCucgau.....................	1	1	seq
	....aacggagaGucaggguucgau.....................	1	1	seq
	....aacUgagaaucaggguucgauu....................	1	1	seq
	....aacggagaaucaggguucgauu....................	10	0	seq
	....acggagaaucaggguucga......................	2	0	seq
	....acggagaaucaggguucgau.....................	8	0	seq

```
                Star                                                  Mature
ggauccuucgaaugucugcccuaucaacuuucgaugguacguuaugcgccuaccauggucgucacggguaacggagaaucaggguucgauuccggagagggagccugagaa
......................................................................acgUagaaucaggguucgau.....................   2    1    seq
......................................................................acggagaaucaggguucgauC....................   2    1    seq
......................................................................acgUagaaucaggguucgauu....................   2    1    seq
......................................................................acggagaaucaggguucgauu....................   14   0    seq
......................................................................acgUagaaucaggguucgauucc..................   2    1    seq
......................................................................acggagaaucaggguucgauucc..................   4    0    seq
.......................................................................cggagaaucaggguucgau.....................   5    0    seq
.......................................................................cggagaaucaggguuUgau.....................   1    1    seq
.......................................................................cgUagaaucaggguucgau.....................   1    1    seq
.......................................................................cggagaaucaggguucgauu....................   5    0    seq
.........................................................................gagaaucaggguucgauu....................   1    0    seq
...................................................................................guucgauuccggagaggga.........   1    0    seq
........................................................................................auuccggagaUggagccugagaa   1    1    seq
..........................................................................................uccggagagggagccugag..   1    0    seq
```
