# Supplementary material for: Identification and Characterization of MicroRNAs from Longitudinal Muscle and Respiratory Tree in Sea Cucumber (Apostichopus japonicus) Using High-Throughput Sequencing
Source: PLoS One. 2015 Aug 5;10(8):e0134899. doi: 10.1371/journal.pone.0134899 (PMC4526669; doi:10.1371/journal.pone.0134899)
Supplement: S2 File — (ZIP) [file pone.0134899.s003.zip › S2 File/The secondary structures of the novel miRNAs in RPT/Scaffold554_1605.pdf]

Provisional ID : Scaffold554_1605
Score total : 2.3
Score for star read(s) : -1.3
Score for read counts : 0
Score for mfe : 2
Score for randfold : 1.6
Score for cons. seed :
Total read count : 581
Mature read count : 581
Loop read count : 0
Star read count : 0

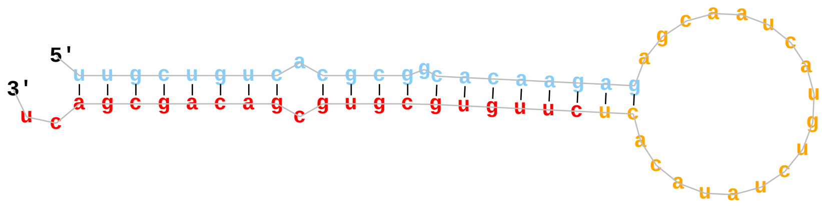

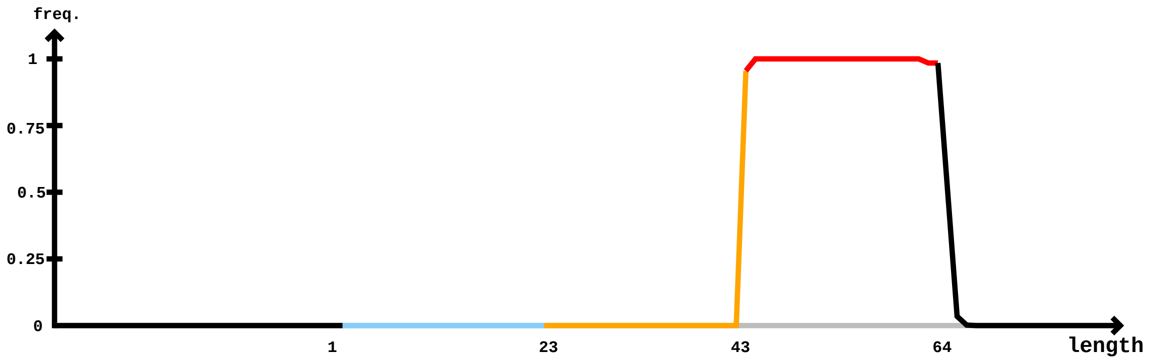

Star Mature

```
5'- uuaucaacauacccagcucucucguuaguugcugucacgcggcacaagagagcaaucaugucuauacacucuugugcgugcgacagcgacugauacagggcucccuuuc   -3'    exp
    ..............((((((...(((((((((((((.((((.((((((((..................)))))))))))).)))))))))))))..)))))).......    reads    mm    sample
    ......................................................................cuugugcgugcgacagcga....................    6        0     seq
    ......................................................................cuuUugcgugcgacagcga....................    1        1     seq
    ......................................................................Uuugugcgugcgacagcga....................    1        1     seq
    ......................................................................cuugugcAugcgacagcgacu..................    3        1     seq
    ......................................................................cuugugcguUcgacagcgacu..................    2        1     seq
    ......................................................................cuuguAcgugcgacagcgacu..................    2        1     seq
    ......................................................................cuugugcgugcgacagcgacC..................    149      1     seq
    ......................................................................cuugugcgugcgacagcgacA..................    20       1     seq
    ......................................................................cuugugcgugcgacagAgacu..................    1        1     seq
    ......................................................................cAugugcgugcgacagcgacu..................    19       1     seq
    ......................................................................cuAgugcgugcgacagcgacu..................    4        1     seq
    ......................................................................cuugugcgugGgacagcgacu..................    1        1     seq
    ......................................................................cuugugcgugcgacagcgacG..................    5        1     seq
    ......................................................................cuugugcgAgcgacagcgacu..................    1        1     seq
    ......................................................................cuugAgcgugcgacagcgacu..................    2        1     seq
    ......................................................................cCugugcgugcgacagcgacu..................    29       1     seq
    ......................................................................cGugugcgugcgacagcgacu..................    29       1     seq
    ......................................................................cuugugcgugcgacGgcgacu..................    8        1     seq
    ......................................................................cuuCugcgugcgacagcgacu..................    3        1     seq
    ......................................................................cuugugcgugcgacagcgaGu..................    1        1     seq
    ......................................................................cuugCgcgugcgacagcgacu..................    3        1     seq
    ......................................................................cuugGgcgugcgacagcgacu..................    5        1     seq
    ......................................................................cuugugcgugcgUcagcgacu..................    1        1     seq
    ......................................................................cuugugcgugcgacagUgacu..................    1        1     seq
    ......................................................................cuuUugcgugcgacagcgacu..................    204      1     seq
    ......................................................................cuugugcgugcgGcagcgacu..................    6        1     seq
    ......................................................................cuugugcgugcAacagcgacu..................    1        1     seq
    ......................................................................cuuAugcgugcgacagcgacu..................    2        1     seq
    ......................................................................cuugugcgugcgacagcgaUu..................    1        1     seq
    ......................................................................cuCgugcgugcgacagcgacu..................    10       1     seq
    ......................................................................cuugugUgugcgacagcgacu..................    4        1     seq
    ......................................................................cuugugcgugcgacagcgGcu..................    7        1     seq
    ......................................................................cuugugcgugAgacagcgacu..................    1        1     seq
    ......................................................................cuugugcguAcgacagcgacu..................    1        1     seq
```

```
                                Star                                    Mature
uuaucaacauacccagcucucucguuaguugcugucacgcggcacaagagagcaaucaugucuauacacucuugugcgugcgacagcgacugauacagggcucccuuuc
......................................................................cuGgugcgugcgacagcgacu..................     6     1     seq
......................................................................cuugugcgCgcgacagcgacu..................     6     1     seq
......................................................................cuugugcgugcgaUagcgacu..................     1     1     seq
......................................................................cuugGgcgugcgacagcgacug.................     1     1     seq
......................................................................cuuUugcgugcgacagcgacug.................     2     1     seq
......................................................................cuugugcgGgcgacagcgacug.................     1     1     seq
......................................................................cuuUugcgugcgacagcgacuga................     2     1     seq
......................................................................cuugugcgugcgacGgcgacuga................     1     1     seq
......................................................................cuuUugcgugcgacagcgacugau...............     1     1     seq
.......................................................................uugugcgugcgacagcga....................     1     0     seq
.......................................................................uugugcgugcgacagcgacC..................     3     1     seq
.......................................................................uugugcgugcgGcagcgacu..................     1     1     seq
.......................................................................uugugcguAcgacagcgacu..................     1     1     seq
.......................................................................uAgugcgugcgacagcgacu..................     1     1     seq
.......................................................................uugugcgugcgacagcgacA..................     1     1     seq
.......................................................................uugCgcgugcgacagcgacug.................     1     1     seq
.......................................................................uugugcgugcgacagcgacCg.................     1     1     seq
.......................................................................uuAugcgugcgacagcgacuga................     1     1     seq
.......................................................................uugugcgugcgacagcAacuga................     1     1     seq
.......................................................................uugugcgCgcgacagcgacuga................     1     1     seq
.......................................................................uugGgcgugcgacagcgacuga................     2     1     seq
.......................................................................uugCgcgugcgacagcgacuga................     1     1     seq
.......................................................................uugugcgugcgacagcgUcuga................     1     1     seq
.......................................................................uugugcgugcgacagcgGcuga................     1     1     seq
.......................................................................uugugcguAcgacagcgacuga................     1     1     seq
.......................................................................uugugcgugcgacGgcgacuga................     3     1     seq
.......................................................................uugugcgugcgGcagcgacuga................     2     1     seq
.......................................................................uugugUgugcgacagcgacuga................     2     1     seq
```
