# Supplementary material for: Identification and Characterization of MicroRNAs from Longitudinal Muscle and Respiratory Tree in Sea Cucumber (Apostichopus japonicus) Using High-Throughput Sequencing
Source: PLoS One. 2015 Aug 5;10(8):e0134899. doi: 10.1371/journal.pone.0134899 (PMC4526669; doi:10.1371/journal.pone.0134899)
Supplement: S2 File — (ZIP) [file pone.0134899.s003.zip › S2 File/The secondary structures of the novel miRNAs in RPT/Scaffold636_1736.pdf]

Provisional ID	:	Scaffold636_1736
Score total	:	1.7
Score for star read(s)	:	-1.3
Score for read counts	:	0
Score for mfe	:	1.4
Score for randfold	:	1.6
Score for cons. seed	:	
Total read count	:	604
Mature read count	:	604
Loop read count	:	0
Star read count	:	0

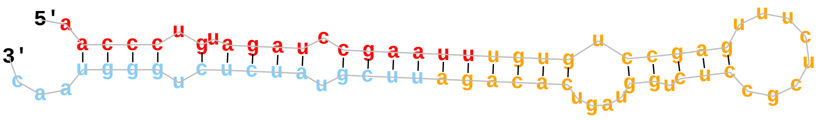

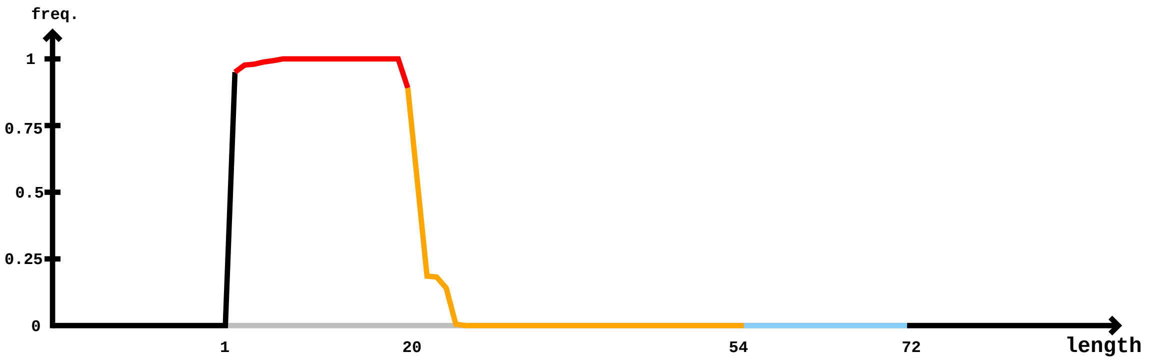

Mature   Star

```
5'- cuuuuugggcuuacaucaacccuguagauccgaauuugugucc gaguuucucgccucugguagucacagauucguaucucuggguaacuguaucccaggacuuacaacu   -3'   exp
..(((((((..((((...((((.(.((((.(((((((((.(((((........))).))....))))))))).))))).))))...)))).)))))))........        reads   mm   sample
.................Uacccuguagauccgaau..........................................................................       65      1    seq
.................Cacccuguagauccgaau..........................................................................       1       1    seq
.................Cacccuguagauccgaauu.........................................................................       1       1    seq
.................Gacccuguagauccgaauu.........................................................................       1       1    seq
.................Uacccuguagauccgaauu.........................................................................       418     1    seq
.................aacccuguagauccgaauC.........................................................................       1       1    seq
.................aacccuguagauUcgaauuu........................................................................       1       1    seq
.................Gacccuguagauccgaauuu........................................................................       4       1    seq
.................aacccuUuagauccgaauuug.......................................................................       1       1    seq
.................aGcccuguagauccgaauuug.......................................................................       1       1    seq
.................aacccCguagauccgaauuugu......................................................................       1       1    seq
.................aacccuguagauccgaauuCgu......................................................................       1       1    seq
.................aacUcuguagauccgaauuugu......................................................................       1       1    seq
.................aacccuUuagauccgaauuugu......................................................................       2       1    seq
.................aacccuguagauccgaaGuugu......................................................................       4       1    seq
.................aacccuguCgauccgaauuugu......................................................................       1       1    seq
.................aacccuguagaGccgaauuugu......................................................................       2       1    seq
.................aacccuguagaucUgaauuugu......................................................................       1       1    seq
.................aacccuguagauccgaauGugu......................................................................       1       1    seq
.................aacccuguagauccgaauCugu......................................................................       4       1    seq
.................aacccuguagGuccgaauuugu......................................................................       1       1    seq
.................aacccuCuagauccgaauuugu......................................................................       1       1    seq
.................aacGcuguagauccgaauuugu......................................................................       1       1    seq
.................aacccuAuagauccgaauuugug.....................................................................       1       1    seq
.................aacccuguagaucAgaauuugug.....................................................................       1       1    seq
.................aacccuguGgauccgaauuugug.....................................................................       2       1    seq
.................aacccuguagGuccgaauuugug.....................................................................       6       1    seq
.................aacccuguCgauccgaauuugug.....................................................................       2       1    seq
.................aacccuguagauccgaUuuugug.....................................................................       1       1    seq
.................aacccuguagauccUaauuugug.....................................................................       2       1    seq
.................aacccuguagaCccgaauuugug.....................................................................       2       1    seq
.................aacccuguagauUcgaauuugug.....................................................................       2       1    seq
.................aacccuUuagauccgaauuugug.....................................................................       19      1    seq
.................aacccuguagaucUgaauuugug.....................................................................       3       1    seq
```

```
                 Mature                                                Star
cuuuuugggcuuacaucaacccuguagauccgaauuugugucccgaguuucucgccucugguagucacagauucguaucucuggguaacuguaucccaggacuuacaacu
.................aacccuguagCuccgaauuugug.....................................................................	1	1	seq
.................aacccugAagauccgaauuugug.....................................................................	1	1	seq
.................aacccugGagauccgaauuugug.....................................................................	3	1	seq
.................aacccuCuagauccgaauuugug.....................................................................	4	1	seq
.................aacccuguagaGccgaauuugug.....................................................................	8	1	seq
.................aacccuguagaCccgaauuugugu....................................................................	1	1	seq
..................acccuguagauccgaauuuUu......................................................................	1	1	seq
..................acccugGagauccgaauuugug.....................................................................	1	1	seq
..................acccuguagaucUgaauuugug.....................................................................	2	1	seq
..................acccuguagauccgaauuCgug.....................................................................	1	1	seq
..................acccuguagGuccgaauuugug.....................................................................	1	1	seq
..................acccuguagaCccgaauuugug.....................................................................	1	1	seq
..................acccugCagauccgaauuugug.....................................................................	1	1	seq
..................accGuguagauccgaauuugug.....................................................................	2	1	seq
..................acccuguagauccgaCuuugug.....................................................................	2	1	seq
..................acccuguagauccgCauuugug.....................................................................	1	1	seq
..................acccCguagauccgaauuugug.....................................................................	1	1	seq
..................acccuguagauccUaauuugug.....................................................................	1	1	seq
..................acccuguagUuccgaauuugugu....................................................................	1	1	seq
...................cccuguagauccgaauuugA......................................................................	1	1	seq
...................ccUuguagauccgaauuugu......................................................................	1	1	seq
....................ccuguagauccgaauuuguA.....................................................................	2	1	seq
....................Ucuguagauccgaauuugug.....................................................................	1	1	seq
....................ccuUuagauccgaauuugug.....................................................................	2	1	seq
.....................cuguagauccgaauuugu......................................................................	1	0	seq
.....................cuguagauccgaauuugug.....................................................................	1	0	seq
.....................cuguagauccgaauuugugu....................................................................	1	0	seq
......................uguagauccgaauuuguA.....................................................................	1	1	seq
......................uUuagauccgaauuugug.....................................................................	1	1	seq
......................uguagauccgaauuuguU.....................................................................	1	1	seq
......................uguagauccgaauuugug.....................................................................	1	0	seq
```
