# Supplementary material for: Identification and Characterization of MicroRNAs from Longitudinal Muscle and Respiratory Tree in Sea Cucumber (Apostichopus japonicus) Using High-Throughput Sequencing
Source: PLoS One. 2015 Aug 5;10(8):e0134899. doi: 10.1371/journal.pone.0134899 (PMC4526669; doi:10.1371/journal.pone.0134899)
Supplement: S2 File — (ZIP) [file pone.0134899.s003.zip › S2 File/The secondary structures of the novel miRNAs in RPT/Scaffold652_1761.pdf]

Provisional ID : Scaffold652_1761
Score total : 2.5
Score for star read(s) : -1.3
Score for read counts : 0
Score for mfe : 2.2
Score for randfold : 1.6
Score for cons. seed :
Total read count : 6455
Mature read count : 6455
Loop read count : 0
Star read count : 0

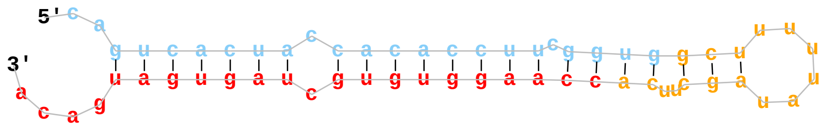

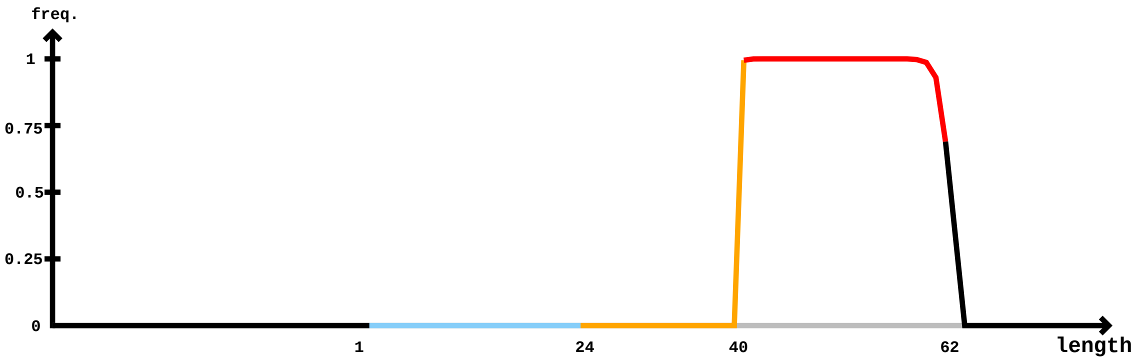

Star Mature

```
5'- gaagacgaucgcaccgugccuagucucccugcagucacuaccacaccuucggugg cuuuuuauagcuucaccaaggugugcuagugaugacagggggaugggaacaau   -3'    exp
    ..............((.((((.((((((((..((((((.(((((((.((((((......)))..)))))))))))).)))))))..)))))))))))).))...     reads      mm          sample
    ......................................................................cGaaggugugcuagugau....................     1          1           seq
    ......................................................................ccGaggugugcuagugau....................     1          1           seq
    ......................................................................ccaaggugugcuagugaC....................     2          1           seq
    ......................................................................cUaaggugugcuagugau....................     1          1           seq
    ......................................................................ccaaggugugcuagugau....................     12         0           seq
    ......................................................................ccaaggugugcuagugaug...................     17         0           seq
    ......................................................................ccaaggugugcuagugaCg...................     50         1           seq
    ......................................................................ccaaggGgugcuagugaug...................     2          1           seq
    ......................................................................ccaaggugAgcuagugauga..................     1          1           seq
    ......................................................................ccaaggugugcuagugaugG..................     1          1           seq
    ......................................................................cUaaggugugcuagugauga..................     1          1           seq
    ......................................................................ccaagguguAcuagugauga..................     1          1           seq
    ......................................................................ccGaggugugcuagugauga..................     1          1           seq
    ......................................................................ccaaggCgugcuagugauga..................     1          1           seq
    ......................................................................ccaaggugugcuagCgauga..................     2          1           seq
    ......................................................................Acaaggugugcuagugauga..................     1          1           seq
    ......................................................................ccaaggugugAuagugauga..................     1          1           seq
    ......................................................................ccaGggugugcuagugauga..................     1          1           seq
    ......................................................................ccaaAgugugcuagugauga..................     1          1           seq
    ......................................................................ccaagguguUcuagugauga..................     2          1           seq
    ......................................................................ccaaggugugcuagGgauga..................     1          1           seq
    ......................................................................ccaaggugugcuagugauAa..................     1          1           seq
    ......................................................................ccaaggAgugcuagugauga..................     4          1           seq
    ......................................................................ccaagguAugcuagugauga..................     1          1           seq
    ......................................................................ccaaggGgugcuagugauga..................     2          1           seq
    ......................................................................ccaaggugugcuagugaCga..................     335        1           seq
    ......................................................................ccaaggugugcuagugaugU..................     2          1           seq
    ......................................................................ccaaggugugGuagugauga..................     1          1           seq
    ......................................................................ccUaggugugcuagugauga..................     1          1           seq
    ......................................................................ccaaggugugcuagugauUa..................     2          1           seq
    ......................................................................ccaagAugugcuagugauga..................     1          1           seq
    ......................................................................ccaaggugugcCagugauga..................     1          1           seq
    ......................................................................ccaagguCugcuagugaugac.................     1          1           seq
    ......................................................................ccaaggugugcuagCgaugac.................     11         1           seq
```

Star Mature

```
gaagacgaucgcaccgugccuagucucccugcagucacuaccacaccuucggugcgcuuuuuauagcuucaccaaggugugcuagugaugacagggggaugggaacaau
......................................................................ccaaggugugcuagGgaugac.................  5     1  seq
......................................................................ccaaggugugcuagugaGgac.................  2     1  seq
......................................................................ccaaggugugcuagugGugac.................  17    1  seq
......................................................................ccaagguguUcuagugaugac.................  1     1  seq
......................................................................ccaaggugugAuagugaugac.................  1     1  seq
......................................................................ccUaggugugcuagugaugac.................  3     1  seq
......................................................................ccaagguAugcuagugaugac.................  1     1  seq
......................................................................ccaaggugugcuagAgaugac.................  3     1  seq
......................................................................ccaaggugugcCagugaugac.................  5     1  seq
......................................................................ccaagAugugcuagugaugac.................  2     1  seq
......................................................................cGaaggugugcuagugaugac.................  6     1  seq
......................................................................ccaaggCgugcuagugaugac.................  19    1  seq
......................................................................ccaaggGgugcuagugaugac.................  41    1  seq
......................................................................ccaaUgugugcuagugaugac.................  1     1  seq
......................................................................ccaaggugugcuagugCugac.................  1     1  seq
......................................................................cUaaggugugcuagugaugac.................  19    1  seq
......................................................................ccCaggugugcuagugaugac.................  1     1  seq
......................................................................ccaaggugugcuagugaCgac.................  1297  1  seq
......................................................................ccaaAgugugcuagugaugac.................  4     1  seq
......................................................................ccaagguUugcuagugaugac.................  2     1  seq
......................................................................ccaaggugugcuagugauAac.................  2     1  seq
......................................................................ccaCggugugcuagugaugac.................  1     1  seq
......................................................................ccaaggugGgcuagugaugac.................  3     1  seq
......................................................................ccaaggugugcuGgugaugac.................  11    1  seq
......................................................................ccaaggugugcuaguUaugac.................  1     1  seq
......................................................................ccaaggugugcuagugauUac.................  2     1  seq
......................................................................ccaaggugCgcuagugaugac.................  13    1  seq
......................................................................ccaaggugugcuagugaugUc.................  1     1  seq
......................................................................ccaaggugugcuagugaugGc.................  11    1  seq
......................................................................ccaagguguAcuagugaugac.................  5     1  seq
......................................................................ccaGggugugcuagugaugac.................  23    1  seq
......................................................................ccaUggugugcuagugaugac.................  4     1  seq
......................................................................ccaaggAgugcuagugaugac.................  19    1  seq
......................................................................ccGaggugugcuagugaugac.................  3     1  seq
......................................................................ccaaggugAgcuagugaugac.................  2     1  seq
......................................................................ccaaggugugcuaguAaugac.................  3     1  seq
......................................................................ccaaggugugcuagugCugaca................  2     1  seq
......................................................................ccaaggugugcuagAgaugaca................  9     1  seq
......................................................................ccaaggugugcuagugaAgaca................  4     1  seq
......................................................................ccaaggugAgcuagugaugaca................  28    1  seq
......................................................................ccaGggugugcuagugaugaca................  195   1  seq
......................................................................ccaagguguCcuagugaugaca................  1     1  seq
......................................................................ccaaggugugcuagugaCgaca................  2094  1  seq
......................................................................ccaaggugugcuaguAaugaca................  17    1  seq
......................................................................ccaaggugGgcuagugaugaca................  21    1  seq
......................................................................ccaaggugugcCagugaugaca................  43    1  seq
......................................................................ccaaggugugcuaUugaugaca................  1     1  seq
......................................................................ccaaggugugcuagugUugaca................  12    1  seq
......................................................................ccaaggugugcuaCugaugaca................  1     1  seq
......................................................................ccaaggugugGuagugaugaca................  4     1  seq
......................................................................ccaaggGgugcuagugaugaca................  376   1  seq
......................................................................ccaagguguAcuagugaugaca................  42    1  seq
......................................................................ccaaggugugcuaAugaugaca................  24    1  seq
......................................................................ccaaggugugcuaguUaugaca................  8     1  seq
......................................................................ccaaggugugcAagugaugaca................  5     1  seq
......................................................................ccaCggugugcuagugaugaca................  1     1  seq
......................................................................ccaaggugugcuCgugaugaca................  4     1  seq
......................................................................cGaaggugugcuagugaugaca................  40    1  seq
......................................................................ccaagCugugcuagugaugaca................  3     1  seq
......................................................................ccaaggugCgcuagugaugaca................  68    1  seq
......................................................................ccaaCgugugcuagugaugaca................  2     1  seq
......................................................................ccaaggugugcuagCgaugaca................  68    1  seq
......................................................................ccaagUugugcuagugaugaca................  10    1  seq
......................................................................ccaaggugugcuagugaugUca................  15    1  seq
......................................................................ccaaggugugcuagugaugCca................  4     1  seq
......................................................................ccaaggugugcuagugauUaca................  15    1  seq
......................................................................ccCaggugugcuagugaugaca................  4     1  seq
......................................................................ccaagguUugcuagugaugaca................  10    1  seq
......................................................................ccaUggugugcuagugaugaca................  23    1  seq
......................................................................ccaagAugugcuagugaugaca................  23    1  seq
```

Star Mature

```
gaagacgaucgcaccgugccuagucucccugcagucacuaccacaccuucggugugcuuuuuauagcuucaccaaggugugcuagugaugacagggggaugggaacaau
......................................................................ccaaggugugAuagugaugaca................   4      1    seq
......................................................................ccaagguguUcuagugaugaca................   56     1    seq
......................................................................ccUaggugugcuagugaugaca................   45     1    seq
......................................................................ccaaggugugcuUgugaugaca................   9      1    seq
......................................................................ccaaggugugcuagugGugaca................   109    1    seq
......................................................................ccaaggugugcuagugaGgaca................   12     1    seq
......................................................................cAaaggugugcuagugaugaca................   2      1    seq
......................................................................ccGaggugugcuagugaugaca................   60     1    seq
......................................................................ccaaggugugcuagugaugGca................   97     1    seq
......................................................................ccaagguAugcuagugaugaca................   20     1    seq
......................................................................ccaaggugugcuagugauAaca................   13     1    seq
......................................................................ccaaUgugugcuagugaugaca................   11     1    seq
......................................................................ccaaggAgugcuagugaugaca................   243    1    seq
......................................................................ccaaggugugcuGgugaugaca................   68     1    seq
......................................................................ccaaggugugcuagGgaugaca................   44     1    seq
......................................................................ccaaggugugcuaguCaugaca................   1      1    seq
......................................................................ccaaAgugugcuagugaugaca................   26     1    seq
......................................................................ccaaggugugcGagugaugaca................   7      1    seq
......................................................................ccaagguCugcuagugaugaca................   3      1    seq
......................................................................ccaaggCgugcuagugaugaca................   251    1    seq
......................................................................cUaaggugugcuagugaugaca................   165    1    seq
.......................................................................caagguAugcuagugaugac.................   1      1    seq
.......................................................................caaggugCgcuagugaugac.................   1      1    seq
.......................................................................caaggCgugcuagugaugac.................   1      1    seq
.......................................................................caaggugCgcuagugaugaca................   1      1    seq
.......................................................................cCaggugugcuagugaugaca................   2      1    seq
.......................................................................caaggugugcuagugaCgaca................   10     1    seq
.......................................................................cGaggugugcuagugaugaca................   2      1    seq
.......................................................................caaggGgugcuagugaugaca................   4      1    seq
.......................................................................caaggugugcuagugaugGca................   1      1    seq
.......................................................................caaUgugugcuagugaugaca................   6      1    seq
.......................................................................Gaaggugugcuagugaugaca................   1      1    seq
.......................................................................caGggugugcuagugaugaca................   1      1    seq
.......................................................................caaggCgugcuagugaugaca................   1      1    seq
........................................................................aaggugugcuagugaugac.................   1      0    seq
```
