# Supplementary material for: Identification and Characterization of MicroRNAs from Longitudinal Muscle and Respiratory Tree in Sea Cucumber (Apostichopus japonicus) Using High-Throughput Sequencing
Source: PLoS One. 2015 Aug 5;10(8):e0134899. doi: 10.1371/journal.pone.0134899 (PMC4526669; doi:10.1371/journal.pone.0134899)
Supplement: S2 File — (ZIP) [file pone.0134899.s003.zip › S2 File/The secondary structures of the novel miRNAs in RPT/Scaffold759_1903.pdf]

Provisional ID	:	Scaffold759_1903
Score total	:	121.4
Score for star read(s)	:	3.9
Score for read counts	:	114.3
Score for mfe	:	1.6
Score for randfold	:	1.6
Score for cons. seed	:	
Total read count	:	236
Mature read count	:	163
Loop read count	:	0
Star read count	:	73

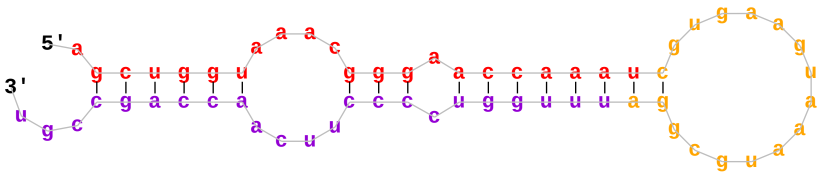

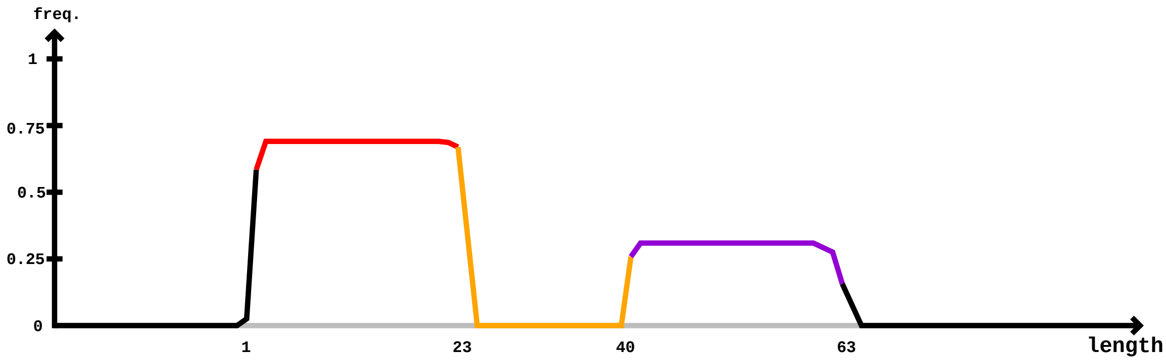

Mature   Star

```
5'- ccggaaaauguagcgcuuuagcugguaaacgggaaccaaaucgugaaguaaaugcggauuuggucccuucaaccagccguagcacugcauucuagacaacucaccuacag  -3'  obs
    ccggaaaauguagcgcuuuagcugguaaacgggaaccaaaucgugaaguaaaugcggauuuggucccuucaaccagccguagcacugcauucuagacaacucaccuacag        exp
    ......(((((((.(((...((((((....(((.((((((((..............)))))))).)))....))))))...))).)))))))..................  reads  mm  sample
    ..................uagcugguaaacgggaaccaaau.....................................................................  2      0   seq
    ..................uagcugguaaacgggaaccaaauc....................................................................  2      0   seq
    ..................uagcugguaaacgggaaccaaauU....................................................................  2      1   seq
    ...................agcugguaaacgggaaccaa.......................................................................  1      0   seq
    ...................agcugguaaacgggaaccaaa......................................................................  3      0   seq
    ...................agcugguaaacgggaaUcaaa......................................................................  1      1   seq
    ...................aCcugguaaacgggaaccaaau.....................................................................  1      1   seq
    ...................agcugguaaacgggUaccaaau.....................................................................  2      1   seq
    ...................agcugguaUacgggaaccaaau.....................................................................  1      1   seq
    ...................aAcugguaaacgggaaccaaau.....................................................................  1      1   seq
    ...................agcugguaaacgggaaccaaaC.....................................................................  7      1   seq
    ...................agcGgguaaacgggaaccaaau.....................................................................  2      1   seq
    ...................agcugguaaacgggaaccaaau.....................................................................  85     0   seq
    ...................agcugguaaacAggaaccaaau.....................................................................  1      1   seq
    ...................aUcugguaaacgggaaccaaau.....................................................................  7      1   seq
    ...................agcCgguaaacgggaaccaaau.....................................................................  1      1   seq
    ...................agcugguaaacgggaaUcaaauc....................................................................  1      1   seq
    ...................agcugguaaacgggaaccaaauU....................................................................  3      1   seq
    ...................agcugguaaacgggaaccaaauc....................................................................  13     0   seq
    ...................agcugguaaacgggaaccaaauA....................................................................  1      1   seq
    ...................aUcugguaaacgggaaccaaauc....................................................................  1      1   seq
    ....................gcugguaaacgggaaccaaau.....................................................................  7      0   seq
    ....................gcugguaaacgggaaccaaauU....................................................................  1      1   seq
    ....................gcuUguaaacgggaaccaaauc....................................................................  1      1   seq
    ....................gcugguaaacgggaaccaaauc....................................................................  16     0   seq
    ..........................................................uCuggucccuucaaccagc.................................  1      1   seq
    ..........................................................uuuggucccuuUaaccagc.................................  1      1   seq
    ..........................................................uCuggucccuucaaccagcc................................  1      1   seq
    ..........................................................uuuggucccuucaaccagUc................................  1      1   seq
    ..........................................................uuuUgucccuucaaccagcc................................  2      1   seq
    ..........................................................uuuggucccuucCaccagccg...............................  1      1   seq
    ..........................................................uuuggucccuCcaaccagccg...............................  1      1   seq
    ..........................................................uuuggucccCucaaccagccg...............................  2      1   seq
```

```
                  Mature                                   Star
ccggaaaauguagcgcuuuagcugguaaacgggaaccaaaucgugaaguaaaugcggauuugguccccuucaaccagccguagcacugcauucuagacaacucaccuacag
..........................................................uuAgguccccuucaaccagccg...............................    1    1    seq
..........................................................uuugguccccuucaaccagUcg...............................    2    1    seq
..........................................................uuugguccccuucaGccagccg...............................    1    1    seq
..........................................................uuugguccccuucaaccaAccg...............................    1    1    seq
..........................................................uuuUguccccuucaaccagccg...............................   13    1    seq
..........................................................uuuggCccccuucaaccagccg...............................    2    1    seq
..........................................................uuuggAccccuucaaccagccg...............................    1    1    seq
..........................................................uuuAguccccuucaaccagccg...............................    1    1    seq
..........................................................uuugguUcccuucaaccagccgu..............................    2    1    seq
..........................................................uuugguccUcuucaaccagccgu..............................    3    1    seq
..........................................................uuugguccccuCcaaccagccgu..............................    8    1    seq
..........................................................uuuggucUccuucaaccagccgu..............................    2    1    seq
..........................................................uuugguccccCucaaccagccgu..............................    2    1    seq
..........................................................uuugguccccuucGaccagccgu..............................    1    1    seq
..........................................................uuuggucGccuucaaccagccgu..............................    1    1    seq
..........................................................uuugguccccuucaGccagccgu..............................    4    1    seq
..........................................................uuuggucccGuucaaccagccgu..............................    1    1    seq
..........................................................uuugguccccuucaaUcagccgu..............................    2    1    seq
..........................................................uuugguccccuucCaccagccgu..............................    3    1    seq
...........................................................uugguccccuucaaccagA.................................    1    1    seq
...........................................................uugguccccuucaaccagc.................................    1    0    seq
...........................................................uuggAccccuucaaccagccg...............................    1    1    seq
...........................................................uugUuccccuucaaccagccg...............................    1    1    seq
...........................................................uugguccccuucaaccGgccgu..............................    1    1    seq
...........................................................uugguccccuucGaccagccgu..............................    1    1    seq
...........................................................uuggCcccuucaaccagccgu...............................    2    1    seq
...........................................................uugUuccccuucaaccagccgu..............................    4    1    seq
```
