# Supplementary material for: Identification and Characterization of MicroRNAs from Longitudinal Muscle and Respiratory Tree in Sea Cucumber (Apostichopus japonicus) Using High-Throughput Sequencing
Source: PLoS One. 2015 Aug 5;10(8):e0134899. doi: 10.1371/journal.pone.0134899 (PMC4526669; doi:10.1371/journal.pone.0134899)
Supplement: S2 File — (ZIP) [file pone.0134899.s003.zip › S2 File/The secondary structures of the novel miRNAs in RPT/Scaffold759_1906.pdf]

Provisional ID	:	Scaffold759_1906
Score total	:	1.1
Score for star read(s)	:	-1.3
Score for read counts	:	0
Score for mfe	:	0.8
Score for randfold	:	1.6
Score for cons. seed	:	
Total read count	:	5735
Mature read count	:	5735
Loop read count	:	0
Star read count	:	0

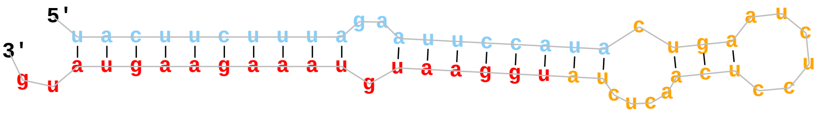

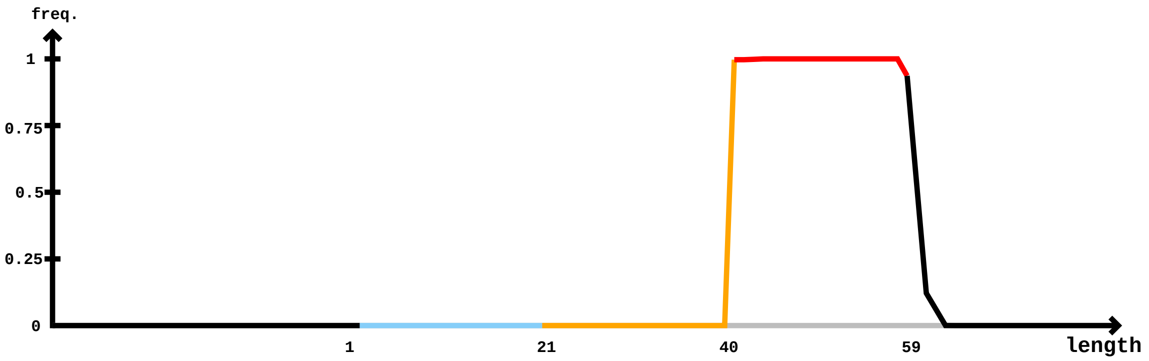

Star   Mature

```
5'- accaucuucaccauaucccaugccauaacauacuucuuuagaauuccauacugaaucuccucaacucuauggaauguaaagaaguauguauugaaacugggaccucaca   -3'   exp
    ...............(((((...((..((((((((((((..((((((((.(((......)))....)))))))).))))))))))))..))....))))).......    reads   mm   sample
    .....................................................................uggGauguaaagaaguau......................    14      1    seq
    .....................................................................uggaauguaaGgaaguau......................    4       1    seq
    .....................................................................uCgaauguaaagaaguau......................    1       1    seq
    .....................................................................uggaauguaaagUaguau......................    1       1    seq
    .....................................................................ugUaauguaaagaaguau......................    6       1    seq
    .....................................................................uggaauguaaagaaAuau......................    1       1    seq
    .....................................................................uggaauguaaagaagAau......................    1       1    seq
    .....................................................................uggaaCguaaagaaguau......................    2       1    seq
    .....................................................................uggaauguaaagaaguau......................    291     0    seq
    .....................................................................uggaauguaGagaaguau......................    1       1    seq
    .....................................................................uggaauguaaagaaguaC......................    13      1    seq
    .....................................................................uUgaauguaaagaaguau......................    16      1    seq
    .....................................................................uggaauguaaagGaguau......................    5       1    seq
    .....................................................................uggaauguaaagaaguaG......................    1       1    seq
    .....................................................................uggaauguaaagaagCau......................    2       1    seq
    .....................................................................uggaauguGaagaaguau......................    2       1    seq
    .....................................................................Aggaauguaaagaaguau......................    1       1    seq
    .....................................................................uggaauguaaagaaguaA......................    1       1    seq
    .....................................................................uggaaugCaaagaaguau......................    1       1    seq
    .....................................................................uggaauguGaagaaguaug.....................    2       1    seq
    .....................................................................uggaauguaaaUaaguaug.....................    1       1    seq
    .....................................................................uggaGuguaaagaaguaug.....................    4       1    seq
    .....................................................................uggaauguaGagaaguaug.....................    3       1    seq
    .....................................................................uggaauguaaagGaguaug.....................    2       1    seq
    .....................................................................uUgaauguaaagaaguaug.....................    29      1    seq
    .....................................................................uggaaugAaaagaaguaug.....................    1       1    seq
    .....................................................................uggaauguaaagaaguauA.....................    33      1    seq
    .....................................................................uggaauguaaagaagCaug.....................    1       1    seq
    .....................................................................uggaauUuaaagaaguaug.....................    2       1    seq
    .....................................................................uggaauguaaagaaguGug.....................    3       1    seq
    .....................................................................uCgaauguaaagaaguaug.....................    4       1    seq
    .....................................................................Cggaauguaaagaaguaug.....................    2       1    seq
    .....................................................................uggaauguaaGgaaguaug.....................    5       1    seq
    .....................................................................ugUaauguaaagaaguaug.....................    14      1    seq
```

Star Mature

```
accaucuucaccauaucccaugccauaacauacuucuuuagaauuccauacugaaucucccucaacucuauggaauguaaagaaguauguauugaaacugggaccucaca
......................................................................uggaauguaaagaaguaug.....................   967   0   seq
......................................................................uggGauguaaagaaguaug.....................   36    1   seq
......................................................................Gggaauguaaagaaguaug.....................   2     1   seq
......................................................................uggaauguaaagaUguaug.....................   1     1   seq
......................................................................uggaauguaaagaagAaug.....................   1     1   seq
......................................................................uggaaCguaaagaaguaug.....................   5     1   seq
......................................................................uAgaauguaaagaaguaug.....................   2     1   seq
......................................................................uggaauguaaagaaguauU.....................   13    1   seq
......................................................................uggaauguaaagaGguaug.....................   3     1   seq
......................................................................uggUauguaaagaaguaug.....................   2     1   seq
......................................................................uggaauguCaagaaguaug.....................   1     1   seq
......................................................................uggaGuguaaagaaguaugu....................   101   1   seq
......................................................................uCgaauguaaagaaguaugu....................   40    1   seq
......................................................................uggaauguaaaAaaguaugu....................   2     1   seq
......................................................................ugAaauguaaagaaguaugu....................   24    1   seq
......................................................................uggaauUuaaagaaguaugu....................   48    1   seq
......................................................................uggaauguaaagaagCaugu....................   56    1   seq
......................................................................uggaauguaaagaaAuaugu....................   12    1   seq
......................................................................uggaaugCaaagaaguaugu....................   48    1   seq
......................................................................uggaauguaaagCaguaugu....................   7     1   seq
......................................................................uggaauguaCagaaguaugu....................   2     1   seq
......................................................................uggaauguaaagUaguaugu....................   9     1   seq
......................................................................uggaauguaaCgaaguaugu....................   9     1   seq
......................................................................uggaauguaaagaagAaugu....................   55    1   seq
......................................................................uggaauguaaagaaCuaugu....................   3     1   seq
......................................................................Gggaauguaaagaaguaugu....................   51    1   seq
......................................................................uggaauguaaagaaguaGgu....................   8     1   seq
......................................................................ugUaauguaaagaaguaugu....................   441   1   seq
......................................................................uggaauguaaagaaguCugu....................   2     1   seq
......................................................................Cggaauguaaagaaguaugu....................   64    1   seq
......................................................................uggaauguaaagGaguaugu....................   79    1   seq
......................................................................uggaaGguaaagaaguaugu....................   6     1   seq
......................................................................uggaaugGaaagaaguaugu....................   28    1   seq
......................................................................uAgaauguaaagaaguaugu....................   60    1   seq
......................................................................uggaauguaaagaaguaugA....................   149   1   seq
......................................................................ugCaauguaaagaaguaugu....................   21    1   seq
......................................................................uggUauguaaagaaguaugu....................   45    1   seq
......................................................................uggaauguaGagaaguaugu....................   118   1   seq
......................................................................uggaauguCaagaaguaugu....................   19    1   seq
......................................................................uggaauguaaagaaguauAu....................   16    1   seq
......................................................................uggaauCuaaagaaguaugu....................   1     1   seq
......................................................................uUgaauguaaagaaguaugu....................   539   1   seq
......................................................................uggCauguaaagaaguaugu....................   7     1   seq
......................................................................uggaauguaaagaaguauCu....................   2     1   seq
......................................................................uggaUuguaaagaaguaugu....................   5     1   seq
......................................................................uggaauguaaGgaaguaugu....................   198   1   seq
......................................................................Aggaauguaaagaaguaugu....................   15    1   seq
......................................................................uggaauguaaagaagGaugu....................   54    1   seq
......................................................................uggGauguaaagaaguaugu....................   867   1   seq
......................................................................uggaauguaaagaaUuaugu....................   4     1   seq
......................................................................uggaauguaaaUaaguaugu....................   5     1   seq
......................................................................uggaauAuaaagaaguaugu....................   13    1   seq
......................................................................uggaauguaUagaaguaugu....................   9     1   seq
......................................................................uggaauguaaagaaguaAgu....................   3     1   seq
......................................................................uggaauguaaagaaguUugu....................   5     1   seq
......................................................................uggaaCguaaagaaguaugu....................   71    1   seq
......................................................................uggaaAguaaagaaguaugu....................   2     1   seq
......................................................................uggaauguaaagaaguauUu....................   13    1   seq
......................................................................uggaauguGaagaaguaugu....................   75    1   seq
......................................................................uggaaugAaaagaaguaugu....................   5     1   seq
......................................................................uggaauguaaagaUguaugu....................   10    1   seq
......................................................................uggaauguaaagaGguaugu....................   99    1   seq
......................................................................uggaCuguaaagaaguaugu....................   5     1   seq
......................................................................uggaauguaaagaCguaugu....................   6     1   seq
......................................................................uggaauguaaaUaaguaugua...................   2     1   seq
......................................................................uggaaugCaaagaaguaugua...................   5     1   seq
......................................................................ugAaauguaaagaaguaugua...................   2     1   seq
......................................................................uggaauguaaagaaUuaugua...................   1     1   seq
......................................................................uggaauguaaagaagGaugua...................   7     1   seq
......................................................................uggGauguaaagaaguaugua...................   77    1   seq
```

```
                                                  Star                                      Mature
accaucuucaccauaucccaugccauaacauacuucuuuagaauuccauacugaaucuccucaacucuauggaauguaaagaaguauguauugaaacugggaccucaca
.....................................................................uggaauguaaagaUguaugua...................   2    1   seq
.....................................................................uCgaauguaaagaaguaugua...................   3    1   seq
.....................................................................uggaaCguaaagaaguaugua...................   7    1   seq
.....................................................................uggaauguGaagaaguaugua...................   5    1   seq
.....................................................................uggaauguaaGgaaguaugua...................   23   1   seq
.....................................................................uggaauguaaagaaguaGgua...................   2    1   seq
.....................................................................uUgaauguaaagaaguaugua...................   65   1   seq
.....................................................................uggaauAuaaagaaguaugua...................   3    1   seq
.....................................................................uggaauguaaagGaguaugua...................   9    1   seq
.....................................................................uggaauguaaagaCguaugua...................   2    1   seq
.....................................................................uggaauguaaagaaAuaugua...................   3    1   seq
.....................................................................uggaauguaGagaaguaugua...................   16   1   seq
.....................................................................uggaauguaaagaagAaugua...................   12   1   seq
.....................................................................uAgaauguaaagaaguaugua...................   9    1   seq
.....................................................................uggaauguaCagaaguaugua...................   2    1   seq
.....................................................................uggUauguaaagaaguaugua...................   5    1   seq
.....................................................................uggaauguaaagaaguauAua...................   2    1   seq
.....................................................................uggaaGguaaagaaguaugua...................   2    1   seq
.....................................................................uggaauguaaagaGguaugua...................   11   1   seq
.....................................................................uggaauguaaagCaguaugua...................   1    1   seq
.....................................................................ugCaauguaaagaaguaugua...................   3    1   seq
.....................................................................ugUaauguaaagaaguaugua...................   30   1   seq
.....................................................................uggaaugGaaagaaguaugua...................   6    1   seq
.....................................................................uggaGuguaaagaaguaugua...................   14   1   seq
.....................................................................uggaauguaUagaaguaugua...................   2    1   seq
.....................................................................uggaauUuaaagaaguaugua...................   5    1   seq
.....................................................................uggaauguaaagaagCaugua...................   6    1   seq
.....................................................................uggaauguaaagaaguauUua...................   2    1   seq
.....................................................................uggaauguCaagaaguaugua...................   1    1   seq
.....................................................................ugUaauguaaagaaguauguau..................   28   1   seq
.....................................................................ugAaauguaaagaaguauguau..................   1    1   seq
.....................................................................uggaauguaUagaaguauguau..................   2    1   seq
.....................................................................uggaauguaaagCaguauguau..................   2    1   seq
.....................................................................uggaauguaaagaagGauguau..................   4    1   seq
.....................................................................uggaauguaaagaagAauguau..................   5    1   seq
.....................................................................uggaauguaCagaaguauguau..................   1    1   seq
.....................................................................uggaauUuaaagaaguauguau..................   105  1   seq
.....................................................................uggaaugCaaagaaguauguau..................   5    1   seq
.....................................................................uggaauguaaagaaUuauguau..................   6    1   seq
.....................................................................uggaauguaaagGaguauguau..................   7    1   seq
.....................................................................ugCaauguaaagaaguauguau..................   1    1   seq
.....................................................................uggGauguaaagaaguauguau..................   65   1   seq
.....................................................................uggaGuguaaagaaguauguau..................   13   1   seq
.....................................................................uggaauguGaagaaguauguau..................   7    1   seq
.....................................................................uggaauguaaaAaaguauguau..................   2    1   seq
.....................................................................uggaauguaaagaCguauguau..................   1    1   seq
.....................................................................uggaauguaaagaGguauguau..................   6    1   seq
.....................................................................uggaaugAaaagaaguauguau..................   1    1   seq
.....................................................................uggaauguaaagaaAuauguau..................   1    1   seq
.....................................................................uggaaugGaaagaaguauguau..................   3    1   seq
.....................................................................uggaauguaaagaaguauAuau..................   2    1   seq
.....................................................................uggaauguaaagaaguaugAau..................   3    1   seq
.....................................................................uggaauguCaagaaguauguau..................   12   1   seq
.....................................................................uggaaCguaaagaaguauguau..................   6    1   seq
.....................................................................uggaauguaaaUaaguauguau..................   3    1   seq
.....................................................................uggaauguaaGgaaguauguau..................   15   1   seq
.....................................................................uggaauguaaagaaguauUuau..................   2    1   seq
.....................................................................uggUauguaaagaaguauguau..................   3    1   seq
.....................................................................uggaauguaGagaaguauguau..................   12   1   seq
.....................................................................uggaauguaaagaagCauguau..................   4    1   seq
.....................................................................uggaauAuaaagaaguauguau..................   3    1   seq
.......................................................................gaauguaaagaaguauguGu..................   7    1   seq
.......................................................................gaauguaaagaaguauguGuu.................   1    1   seq
........................................................................aauguaaagaaguauguGu..................   10   1   seq
```
