# Supplementary material for: Identification and Characterization of MicroRNAs from Longitudinal Muscle and Respiratory Tree in Sea Cucumber (Apostichopus japonicus) Using High-Throughput Sequencing
Source: PLoS One. 2015 Aug 5;10(8):e0134899. doi: 10.1371/journal.pone.0134899 (PMC4526669; doi:10.1371/journal.pone.0134899)
Supplement: S2 File — (ZIP) [file pone.0134899.s003.zip › S2 File/The secondary structures of the novel miRNAs in RPT/Scaffold762_1911.pdf]

Provisional ID	:	Scaffold762_1911
Score total	:	2.1
Score for star read(s)	:	-1.3
Score for read counts	:	0
Score for mfe	:	1.8
Score for randfold	:	1.6
Score for cons. seed	:	
Total read count	:	74312
Mature read count	:	74312
Loop read count	:	0
Star read count	:	0

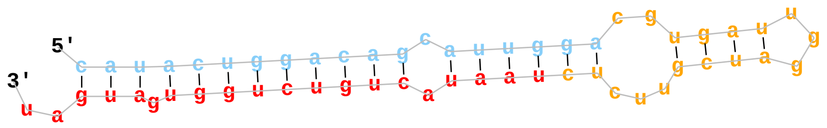

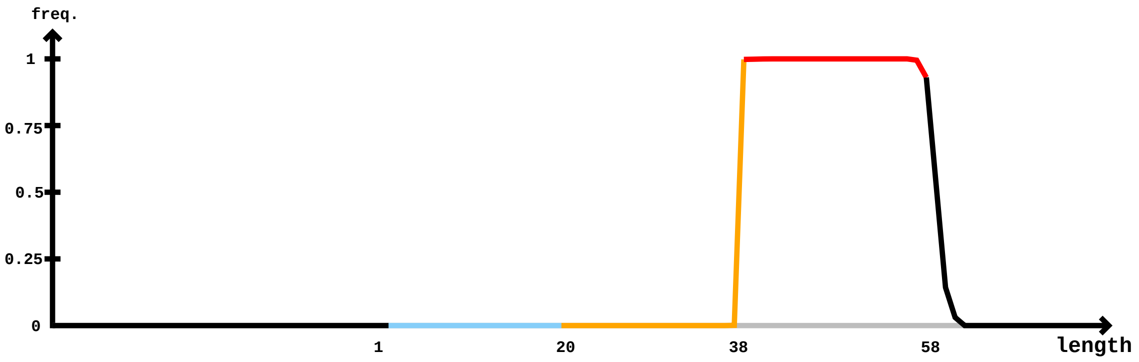


```
                                             Star                        Mature
5'- guguuugcgauuuguugagccuaguggugacaucauacuggacagcauuggacgugauuggaucguucucuaauacugucuggugaugauguugacacuggcuuuguu   -3'    exp
    ...............(((((.((((..(((((((((((((((((.((((((..((((...))))...)))))).))))))))).))))))))..)))))))))....   reads      mm        sample
    .....................................................................cuaUuacugucuggugauga...................   1          1         seq
    .....................................................................cuaauacugucuggugauga...................   6          0         seq
    .....................................................................cGaauacugucuggugaugau..................   4          1         seq
    .....................................................................cuaGuacugucuggugaugau..................   3          1         seq
    .....................................................................cCaauacugucuggugaugau..................   5          1         seq
    .....................................................................cuaaCacugucuggugaugau..................   3          1         seq
    .....................................................................cuaauacugucuggGgaugau..................   3          1         seq
    .....................................................................cuaauacugucuggugaugGu..................   1          1         seq
    .....................................................................cuaauacugucuggCgaugau..................   1          1         seq
    .....................................................................cuaauacugucuggugaugaA..................   1          1         seq
    .....................................................................cuaauacugucuggugGugau..................   1          1         seq
    .....................................................................cuaauacugCcuggugaugau..................   1          1         seq
    .....................................................................cuaauacugucCggugaugau..................   1          1         seq
    .....................................................................cuaauacugucuggugaugaC..................   19         1         seq
    .....................................................................cuaauacugucuggAgaugau..................   1          1         seq
    .....................................................................cuaauacuguGggugaugaug.................   1          1         seq
    .....................................................................cuaauacugucuggGgaugaug.................   1          1         seq
    .....................................................................cuaauacugucuggugauUaug.................   2          1         seq
    .....................................................................cuaauacCgucuggugaugaug.................   1          1         seq
    .....................................................................cuaauGcugucuggugaugaugu................   1          1         seq
    .....................................................................cuaaCacugucuggugaugaugu................   1          1         seq
    .....................................................................cuaGuacugucuggugaugaugu................   1          1         seq
    ......................................................................uaauacugucuggugauA....................   16         1         seq
    ......................................................................uaauacugucuAgugaug....................   1          1         seq
    ......................................................................uaauacugucuggGgaug....................   2          1         seq
    ......................................................................uaauacugucuggAgaug....................   1          1         seq
    ......................................................................uaauacugucCggugaug....................   1          1         seq
    ......................................................................uGauacugucuggugaug....................   2          1         seq
    ......................................................................Gaauacugucuggugaug....................   3          1         seq
    ......................................................................uUauacugucuggugaug....................   5          1         seq
    ......................................................................uaaGacugucuggugaug....................   8          1         seq
    ......................................................................uaauacugucuggugGug....................   3          1         seq
    ......................................................................uaauacugucuggugauC....................   2          1         seq
    ......................................................................uaauacugucuggugauU....................   12         1         seq
```

```
                                 Star                                      Mature
guguuugcgauuuguugagccuaguggugacaucauacuggacagcauuggacgugauuggaucguucucuaauacugucuggugaugauguugacacuggcuuuguu
......................................................................uaauacugucugAugaug....................  2     1   seq
......................................................................uaauacAgucuggugaug....................  1     1   seq
......................................................................uaauacugucGggugaug....................  1     1   seq
......................................................................uaGuacugucuggugaug....................  1     1   seq
......................................................................uaauacugucuggugaCg....................  1     1   seq
......................................................................uaauacuAucuggugaug....................  1     1   seq
......................................................................uaauacugucuggugaug....................  303   0   seq
......................................................................uaauacugucuggCgaug....................  2     1   seq
......................................................................uaUuacugucuggugaug....................  1     1   seq
......................................................................uaauacugucuggugCug....................  1     1   seq
......................................................................uaauacugucuggugauUa...................  5     1   seq
......................................................................uaauacugucugguUauga...................  4     1   seq
......................................................................uaauacuguUuggugauga...................  4     1   seq
......................................................................uaauacugucuggCgauga...................  28    1   seq
......................................................................uaauacugucAggugauga...................  3     1   seq
......................................................................Gaauacugucuggugauga...................  23    1   seq
......................................................................uaauacugucugAugauga...................  5     1   seq
......................................................................uaauacugucugguAauga...................  3     1   seq
......................................................................uaauacugucCggugauga...................  8     1   seq
......................................................................uaauacuguAuggugauga...................  3     1   seq
......................................................................uaauacugAcuggugauga...................  3     1   seq
......................................................................uaauacugucuggugauAa...................  3     1   seq
......................................................................uaaAacugucuggugauga...................  1     1   seq
......................................................................uGauacugucuggugauga...................  37    1   seq
......................................................................uaauacugCcuggugauga...................  10    1   seq
......................................................................uaauaAugucuggugauga...................  1     1   seq
......................................................................uaauaUugucuggugauga...................  2     1   seq
......................................................................uaUuacugucuggugauga...................  12    1   seq
......................................................................uUauacugucuggugauga...................  37    1   seq
......................................................................uaauacugucuggugaAga...................  4     1   seq
......................................................................uaGuacugucuggugauga...................  28    1   seq
......................................................................uaauacGgucuggugauga...................  7     1   seq
......................................................................uaauacugucugUugauga...................  1     1   seq
......................................................................uaaGacugucuggugauga...................  114   1   seq
......................................................................uaauacugucuAgugauga...................  7     1   seq
......................................................................uaauacugucuggugCuga...................  11    1   seq
......................................................................uaauacugucuggugaugC...................  14    1   seq
......................................................................uaauacugucuggugaugU...................  78    1   seq
......................................................................uaauacugucuggugGuga...................  27    1   seq
......................................................................uaauacugucuggugaCga...................  13    1   seq
......................................................................uaauacugucuggugUuga...................  1     1   seq
......................................................................uaauGcugucuggugauga...................  7     1   seq
......................................................................uaaCacugucuggugauga...................  12    1   seq
......................................................................uaauCcugucuggugauga...................  4     1   seq
......................................................................uaauacuCucuggugauga...................  1     1   seq
......................................................................uaauacugucuggugaugG...................  32    1   seq
......................................................................uaauacAgucuggugauga...................  1     1   seq
......................................................................uaauaGugucuggugauga...................  3     1   seq
......................................................................Caauacugucuggugauga...................  14    1   seq
......................................................................uaauacuAucuggugauga...................  3     1   seq
......................................................................uaauacuUucuggugauga...................  4     1   seq
......................................................................uaauacugucuggGgauga...................  20    1   seq
......................................................................Aaauacugucuggugauga...................  2     1   seq
......................................................................uaauacugucugguCauga...................  2     1   seq
......................................................................uaCuacugucuggugauga...................  1     1   seq
......................................................................uaauUcugucuggugauga...................  1     1   seq
......................................................................uaauacugucGggugauga...................  4     1   seq
......................................................................uaauacugucuggAgauga...................  22    1   seq
......................................................................uaauacugucuggugauga...................  4139  0   seq
......................................................................uaauacCgucuggugauga...................  18    1   seq
......................................................................uaauacugucuggugaGga...................  2     1   seq
......................................................................uaauacugGcuggugauga...................  7     1   seq
......................................................................uaauUcugucuggugaugau..................  14    1   seq
......................................................................uaauacuUucuggugaugau..................  65    1   seq
......................................................................Aaauacugucuggugaugau..................  108   1   seq
......................................................................uaauacuguUuggugaugau..................  77    1   seq
......................................................................uaauacugucugUugaugau..................  13    1   seq
......................................................................uaGuacugucuggugaugau..................  720   1   seq
......................................................................uaauacugCcuggugaugau..................  545   1   seq
......................................................................uaauacugucuggugaugUu..................  61    1   seq
```

Star Mature

guguuugcgauuuguugagccuaguggugacaucauacuggacagcauuggacgugauuggaucguucucuaauacugucuggugaugauguugacacuggcuuuguu			
....uaauacugucuggugaugaC..................	8064	1	seq
....uaauacugucugCugaugau..................	7	1	seq
....uaauaAugucuggugaugau..................	15	1	seq
....uaauacuguGuggugaugau..................	41	1	seq
....uaCuacugucuggugaugau..................	31	1	seq
....uaauacugucuggugauUau..................	94	1	seq
....uaauacCgucuggugaugau..................	471	1	seq
....uaauacugucuggugaugGu..................	577	1	seq
....uaauacugucuggGgaugau..................	654	1	seq
....uaauacugucuggugaAgau..................	26	1	seq
....uaauacugucuggugaGgau..................	44	1	seq
....uaauCcugucuggugaugau..................	71	1	seq
....uCauacugucuggugaugau..................	15	1	seq
....uaauacugucugguUaugau..................	187	1	seq
....uaaGacugucuggugaugau..................	2773	1	seq
....uaauacuguAuggugaugau..................	34	1	seq
....uaauacugucAggugaugau..................	70	1	seq
....uaauacuCucuggugaugau..................	11	1	seq
....uaauacugGcuggugaugau..................	73	1	seq
....uaauacugAcuggugaugau..................	77	1	seq
....uaauacugucuggugUugau..................	58	1	seq
....uaauacGgucuggugaugau..................	124	1	seq
....Caauacugucuggugaugau..................	440	1	seq
....uaauacAgucuggugaugau..................	39	1	seq
....uaaAacugucuggugaugau..................	76	1	seq
....uaauacugucugAugaugau..................	114	1	seq
....uaauGcugucuggugaugau..................	323	1	seq
....uaauacugucuggAgaugau..................	678	1	seq
....uaauacuAucuggugaugau..................	108	1	seq
....uaUuacugucuggugaugau..................	378	1	seq
....uaauaUugucuggugaugau..................	85	1	seq
....uUauacugucuggugaugau..................	1004	1	seq
....uaauaGugucuggugaugau..................	11	1	seq
....uaauacugucuggugCugau..................	356	1	seq
....uaauacugucuggugauAau..................	86	1	seq
....Gaauacugucuggugaugau..................	560	1	seq
....uGauacugucuggugaugau..................	751	1	seq
....uaauacugucGggugaugau..................	68	1	seq
....uaaCacugucuggugaugau..................	256	1	seq
....uaauacugucuggugauCau..................	7	1	seq
....uaauacugucuggugaugCu..................	30	1	seq
....uaauacugucCggugaugau..................	375	1	seq
....uaauacugucuggugGugau..................	701	1	seq
....uaauacugucuCgugaugau..................	8	1	seq
....uaauacugucuggCgaugau..................	765	1	seq
....uaauacugucugguCaugau..................	9	1	seq
....uaauacugucuAgugaugau..................	171	1	seq
....uaauacugucuggugaugaA..................	624	1	seq
....uaauacugucuggugaCgau..................	277	1	seq
....uaauacugucuggugaugaG..................	287	1	seq
....uaauacugucuUgugaugau..................	60	1	seq
....uaauacugucugAugaugaug.................	350	1	seq
....uaauacuguUuggugaugaug.................	231	1	seq
....uaauacugucuggugaugaGg.................	357	1	seq
....uaUuacugucuggugaugaug.................	987	1	seq
....uaauacugucGggugaugaug.................	155	1	seq
....uaauacugucCggugaugaug.................	1007	1	seq
....uaauacugucuggCgaugaug.................	2129	1	seq
....uaaCacugucuggugaugaug.................	749	1	seq
....uaauacugGcuggugaugaug.................	225	1	seq
....uaauacugucuggugaCgaug.................	878	1	seq
....uGauacugucuggugaugaug.................	2073	1	seq
....uaGuacugucuggugaugaug.................	1022	1	seq
....uaauUcugucuggugaugaug.................	51	1	seq
....uaauacugucuCgugaugaug.................	25	1	seq
....uaauacugucugUugaugaug.................	38	1	seq
....uaauacugCcuggugaugaug.................	1436	1	seq
....uaauacugucuggugCugaug.................	736	1	seq
....uaauacugucAggugaugaug.................	204	1	seq
....uaauacugucugguUaugaug.................	423	1	seq

```
                                                 Star                                   Mature
guguuugcgauuuguugagccuaguggugacaucauacuggacagcauuggacgugauuggaucguucucuaauacugucuggugaugauguugacacuggcuuuguu
......................................................................uaauacuUucuggugaugaug.................  223   1   seq
......................................................................uaauacugucuggugauAaug.................  263   1   seq
......................................................................uaauacugucuggugaugCug.................  89    1   seq
......................................................................uaauacugucuUgugaugaug.................  176   1   seq
......................................................................uaauacGgucuggugaugaug.................  341   1   seq
......................................................................uaaAacugucuggugaugaug.................  160   1   seq
......................................................................uaauacugucuggugaAgaug.................  99    1   seq
......................................................................uaauacugucuggugUugaug.................  111   1   seq
......................................................................uaauaGugucuggugaugaug.................  67    1   seq
......................................................................uaauacugucuggugaugaAg.................  121   1   seq
......................................................................uaauacugucuggugGugaug.................  1690  1   seq
......................................................................uaauacugucuggGgaugaug.................  1803  1   seq
......................................................................uaauacugucugCugaugaug.................  19    1   seq
......................................................................uaauCcugucuggugaugaug.................  155   1   seq
......................................................................uaauacugucuAgugaugaug.................  454   1   seq
......................................................................uaauacCgucuggugaugaug.................  1249  1   seq
......................................................................uaauacugAcuggugaugaug.................  208   1   seq
......................................................................uaauacugucuggugaGgaug.................  125   1   seq
......................................................................uaauGcugucuggugaugaug.................  868   1   seq
......................................................................uaauacugucuggugaugaCg.................  845   1   seq
......................................................................uaauacuCucuggugaugaug.................  33    1   seq
......................................................................uaauacAgucuggugaugaug.................  102   1   seq
......................................................................uUauacugucuggugaugaug.................  2998  1   seq
......................................................................uCauacugucuggugaugaug.................  34    1   seq
......................................................................uaauaAugucuggugaugaug.................  32    1   seq
......................................................................uaauaUugucuggugaugaug.................  230   1   seq
......................................................................uaauacuguGuggugaugaug.................  95    1   seq
......................................................................uaauacugucuggugaugGug.................  1590  1   seq
......................................................................uaauacuguAuggugaugaug.................  61    1   seq
......................................................................uaaGacugucuggugaugaug.................  4597  1   seq
......................................................................uaCuacugucuggugaugaug.................  48    1   seq
......................................................................uaauacuAucuggugaugaug.................  191   1   seq
......................................................................uaauacugucuggugauCaug.................  13    1   seq
......................................................................uaauacugucuggAgaugaug.................  2016  1   seq
......................................................................uaauacugucuggugauUaug.................  233   1   seq
......................................................................uaauacugucuggugaugUug.................  132   1   seq
......................................................................uaauacugucugguCaugaug.................  37    1   seq
......................................................................uaauacugucugguCaugaugu................  6     1   seq
......................................................................uaauacugucuggugaugaAgu................  29    1   seq
......................................................................uaauacuguUuggugaugaugu................  77    1   seq
......................................................................uaauacugucuCgugaugaugu................  9     1   seq
......................................................................uaauacuguGuggugaugaugu................  44    1   seq
......................................................................uaGuacugucuggugaugaugu................  268   1   seq
......................................................................uaauacGgucuggugaugaugu................  93    1   seq
......................................................................uaauacugucuggugauAaugu................  54    1   seq
......................................................................uaauacugAcuggugaugaugu................  47    1   seq
......................................................................uaauacugucuAgugaugaugu................  124   1   seq
......................................................................uaauaUugucuggugaugaugu................  70    1   seq
......................................................................uaauaAugucuggugaugaugu................  9     1   seq
......................................................................uaauacugucugguUaugaugu................  156   1   seq
......................................................................uaauUcugucuggugaugaugu................  12    1   seq
......................................................................uaauacugucugUugaugaugu................  8     1   seq
......................................................................uaauacugucGggugaugaugu................  49    1   seq
......................................................................uaauacugucuggugGugaugu................  471   1   seq
......................................................................uaauacugucuUgugaugaugu................  54    1   seq
......................................................................uaauaGugucuggugaugaugu................  7     1   seq
......................................................................uaauacugucuggAgaugaugu................  516   1   seq
......................................................................uaaCacugucuggugaugaugu................  204   1   seq
......................................................................uaauacuAucuggugaugaugu................  48    1   seq
......................................................................uaauacugucuggGgaugaugu................  444   1   seq
......................................................................uaauacugucuggugauUaugu................  64    1   seq
......................................................................uaauacugucugAugaugaugu................  105   1   seq
......................................................................uaauacugucugCugaugaugu................  4     1   seq
......................................................................uaauacugucuggugaugaCgu................  216   1   seq
......................................................................uaauacAgucuggugaugaugu................  27    1   seq
......................................................................uaauacugucuggugUugaugu................  28    1   seq
......................................................................uaauacugucuggugCugaugu................  346   1   seq
......................................................................uaauacCgucuggugaugaugu................  365   1   seq
......................................................................uaauCcugucuggugaugaugu................  31    1   seq
......................................................................uaCuacugucuggugaugaugu................  16    1   seq
```

```
                                    Star                                 Mature
guguuugcgauuuguugagccuaguggugacaucauacuggacagcauuggacgugauuggaucguucucuaauacugucuggugaugauguugacacuggcuuuguu
......................................................................uaauacugucAggugaugaugu................   65     1    seq
......................................................................uaauacugucuggugaugaGgu................   81     1    seq
......................................................................uaauacuCucuggugaugaugu................   14     1    seq
......................................................................uaauacugucCggugaugaugu................   323    1    seq
......................................................................uaauacugucuggugaCgaugu................   312    1    seq
......................................................................uaaGacugucuggugaugaugu................   1323   1    seq
......................................................................uaauGcugucuggugaugaugu................   261    1    seq
......................................................................uaauacugucuggugaugUugu................   28     1    seq
......................................................................uaaAacugucuggugaugaugu................   45     1    seq
......................................................................uaauacugucuggugaugCugu................   16     1    seq
......................................................................uaauacugucuggugauCaugu................   9      1    seq
......................................................................uaauacuUucuggugaugaugu................   60     1    seq
......................................................................uaauacugucuggugaugGugu................   410    1    seq
......................................................................uaauacuguAuggugaugaugu................   13     1    seq
......................................................................uaUuacugucuggugaugaugu................   264    1    seq
......................................................................uaauacugucuggugaGgaugu................   38     1    seq
......................................................................uaauacugCcuggugaugaugu................   382    1    seq
......................................................................uaauacugGcuggugaugaugu................   55     1    seq
......................................................................uaauacugucuggugaAgaugu................   35     1    seq
......................................................................uaauacugucuggCgaugaugu................   592    1    seq
......................................................................uaauacugucuAgugaugauguu...............   56     1    seq
......................................................................uaauacugCcuggugaugauguu...............   178    1    seq
......................................................................uaauacugucuggugUugauguu...............   17     1    seq
......................................................................uaauacugucugAugaugauguu...............   43     1    seq
......................................................................uaauacugucGggugaugauguu...............   19     1    seq
......................................................................uaauacugucuggCgaugauguu...............   285    1    seq
......................................................................uaauacugGcuggugaugauguu...............   29     1    seq
......................................................................uaauacugucuggugaAgauguu...............   13     1    seq
......................................................................uaauacuguGuggugaugauguu...............   6      1    seq
......................................................................uaauacugucCggugaugauguu...............   120    1    seq
......................................................................uaauacugucugguCaugauguu...............   4      1    seq
......................................................................uaauacugucuggugaGgauguu...............   12     1    seq
......................................................................uaauacuguUuggugaugauguu...............   30     1    seq
......................................................................uaauacugucugUugaugauguu...............   2      1    seq
......................................................................uaauacugucugguUaugauguu...............   50     1    seq
......................................................................uaauacugucuggGgaugauguu...............   278    1    seq
......................................................................uaauacuguAuggugaugauguu...............   9      1    seq
......................................................................uaauacugucuCgugaugauguu...............   4      1    seq
......................................................................uaauacugucuggugCugauguu...............   111    1    seq
......................................................................uaauacugucuggAgaugauguu...............   292    1    seq
......................................................................uaauacugucAggugaugauguu...............   31     1    seq
......................................................................uaauacugAcuggugaugauguu...............   25     1    seq
......................................................................uaauacuUucuggugaugauguu...............   20     1    seq
......................................................................uaauacugucuggugaCgauguu...............   142    1    seq
......................................................................uaauacCgucuggugaugauguu...............   153    1    seq
......................................................................uaauacugucuggugGugauguu...............   198    1    seq
......................................................................uaauacGgucuggugaugauguu...............   43     1    seq
......................................................................uaauacugucuUgugaugauguu...............   28     1    seq
......................................................................uaauacAgucuggugaugauguu...............   27     1    seq
......................................................................uaauacugucugCugaugauguu...............   3      1    seq
......................................................................uaauacuAucuggugaugauguu...............   32     1    seq
.......................................................................aauacugucuggugauga...................   1      0    seq
.......................................................................Uauacugucuggugaugau..................   8      1    seq
.......................................................................aauacugGcuggugaugau..................   1      1    seq
.......................................................................Gauacugucuggugaugau..................   1      1    seq
.......................................................................aauacugucuggugaugau..................   10     0    seq
.......................................................................aauacugCcuggugaugau..................   1      1    seq
.......................................................................aauacugucuggGgaugaug.................   1      1    seq
.......................................................................aauGcugucuggugaugaug.................   1      1    seq
.......................................................................aauacugucCggugaugaug.................   1      1    seq
.......................................................................aauacugucuggugaugauA.................   4      1    seq
.......................................................................aauacugCcuggugaugaug.................   1      1    seq
.......................................................................aaGacugucuggugaugaug.................   1      1    seq
.......................................................................Uauacugucuggugaugaug.................   17     1    seq
.......................................................................aauacugucuggugaugGug.................   1      1    seq
.......................................................................aauacugucuggugaugauUu................   1      1    seq
.......................................................................aauGcugucuggugaugaugu................   1      1    seq
.......................................................................aauacugucCggugaugaugu................   1      1    seq
.......................................................................aGuacugucuggugaugaugu................   1      1    seq
.......................................................................aCuacugucuggugaugaugu................   1      1    seq
```

Star **Mature**

```
guguuugcgauuuguugagccuaguggugacaucauacuggacagcauuggacgugauuggaucguucucuaauacugucuggugaugauguugacacuggcuuuguu
.......................................................................aauacugucuggugaCgaugu................	1	1	seq
.......................................................................aauacugucuggugaugaCgu................	1	1	seq
.......................................................................aUuacugucuggugaugaugu................	1	1	seq
.......................................................................aauacugucuggCgaugaugu................	1	1	seq
.......................................................................aauacugucuggGgaugauguu...............	1	1	seq
.......................................................................aauacugucuggugaugauUuu...............	1	1	seq
.......................................................................aauacugucuggAgaugauguu...............	1	1	seq
.......................................................................aauacuUucuggugaugauguu...............	1	1	seq
.......................................................................aauacugucuggugGugauguu...............	1	1	seq
........................................................................auacugucuggugaugau..................	17	0	seq
........................................................................auacugucuggugaugaC..................	2	1	seq
........................................................................aGacugucuggugaugau..................	1	1	seq
........................................................................auacugCcuggugaugaug.................	2	1	seq
........................................................................auacugucuggugaugauA.................	3	1	seq
........................................................................auacugucuggugaugaug.................	27	0	seq
........................................................................auacugucuggugaugaugC................	1	1	seq
........................................................................auacugucuggugaugaCgu................	1	1	seq
........................................................................auacGgucuggugaugaugu................	1	1	seq
........................................................................auacugucuggGgaugauguu...............	1	1	seq
........................................................................aAacugucuggugaugauguu...............	1	1	seq
........................................................................aGacugucuggugaugauguu...............	2	1	seq
........................................................................aCacugucuggugaugauguu...............	1	1	seq
........................................................................auacugucuggugaugaugGu...............	1	1	seq
.........................................................................uacugucugguAaugaug.................	1	1	seq
.........................................................................uacugucuggugaugaug.................	10	0	seq
.........................................................................uacugucCggugaugaugu................	1	1	seq
.........................................................................uacugucuggugaugaugu................	13	0	seq
```
