# Supplementary material for: Identification and Characterization of MicroRNAs from Longitudinal Muscle and Respiratory Tree in Sea Cucumber (Apostichopus japonicus) Using High-Throughput Sequencing
Source: PLoS One. 2015 Aug 5;10(8):e0134899. doi: 10.1371/journal.pone.0134899 (PMC4526669; doi:10.1371/journal.pone.0134899)
Supplement: S2 File — (ZIP) [file pone.0134899.s003.zip › S2 File/The secondary structures of the novel miRNAs in RPT/Scaffold823_1975.pdf]

Provisional ID	:	Scaffold823_1975
Score total	:	2.4
Score for star read(s)	:	-1.3
Score for read counts	:	0
Score for mfe	:	2.1
Score for randfold	:	1.6
Score for cons. seed	:	
Total read count	:	23
Mature read count	:	23
Loop read count	:	0
Star read count	:	0

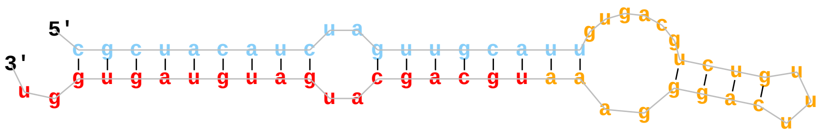

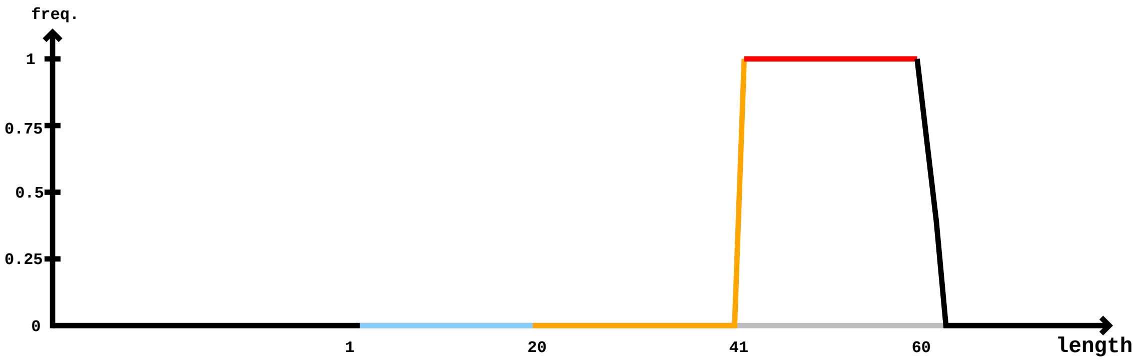

Star   Mature

```
5'- aagcugucauugaugucuacuguucuacgccgcuacaucuaguugcauugugacgucuguuucagggaaaugcagcaugauguagugguguagcucaguaagaccauu   -3'    exp
    .............((((((((..((((((((((((((..((((((((......((((...))))..))))))))..))))))))))))))..)))).))))....   reads      mm         sample
    ......................................................................ugcagcaugauguaguggu...................   8          0          seq
    ......................................................................ugcagcaugauguaguggG...................   1          1          seq
    ......................................................................ugcagcauAauguaguggu...................   1          1          seq
    ......................................................................ugcagcaugauguaguggC...................   1          1          seq
    ......................................................................ugcagcaugauguagugguA..................   1          1          seq
    ......................................................................ugUagcaugauguaguggug..................   1          1          seq
    ......................................................................uUcagcaugauguaguggug..................   1          1          seq
    ......................................................................ugcagcaugauguagGggugu.................   1          1          seq
    ......................................................................ugcagcaugaugAaguggugu.................   1          1          seq
    ......................................................................ugcGgcaugauguaguggugu.................   1          1          seq
    ......................................................................uUcagcaugauguaguggugu.................   3          1          seq
    ......................................................................ugcaCcaugauguaguggugu.................   1          1          seq
    ......................................................................ugcagcGugauguaguggugu.................   1          1          seq
    ......................................................................ugcaAcaugauguaguggugu.................   1          1          seq
```
