# Supplementary material for: Identification and Characterization of MicroRNAs from Longitudinal Muscle and Respiratory Tree in Sea Cucumber (Apostichopus japonicus) Using High-Throughput Sequencing
Source: PLoS One. 2015 Aug 5;10(8):e0134899. doi: 10.1371/journal.pone.0134899 (PMC4526669; doi:10.1371/journal.pone.0134899)
Supplement: S2 File — (ZIP) [file pone.0134899.s003.zip › S2 File/The secondary structures of the novel miRNAs in RPT/Scaffold838_1989.pdf]

Provisional ID	:	Scaffold838_1989
Score total	:	1.2
Score for star read(s)	:	-1.3
Score for read counts	:	0
Score for mfe	:	0.9
Score for randfold	:	1.6
Score for cons. seed	:	
Total read count	:	16431
Mature read count	:	16431
Loop read count	:	0
Star read count	:	0

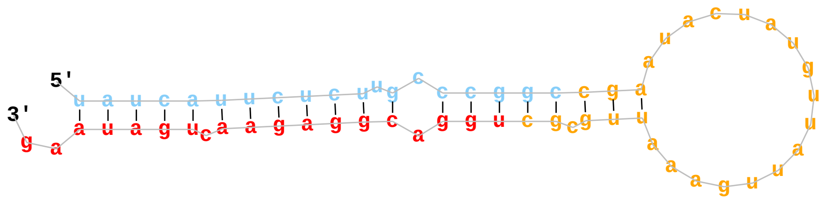

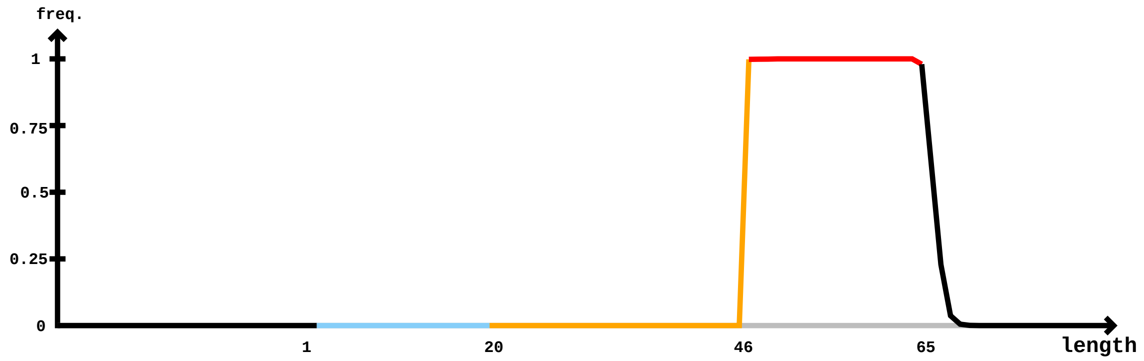

Star **Mature**

```
5'- aucauauuugucuuuuaucuuuccuuaucauucucuugcccggccgaauacuauguuauugaaauugcgcuggacggagaacugauaagggcuuauauaagacacgug   -3'   exp
    ....(((.((((((.(((...((((((((((((((.(.((((((((.................))).))))).))))))).)))))))))...))).)))))).)))   reads   mm   sample
    .....................................................................cCggacggagaacugauaagg..................   1       1    seq
    ......................................................................uAgacggagaacugauaa....................   1       1    seq
    ......................................................................uUgacggagaacugauaa....................   10      1    seq
    ......................................................................uggacggagCacugauaa....................   1       1    seq
    ......................................................................uggacggCgaacugauaa....................   1       1    seq
    ......................................................................uggacggagaGcugauaa....................   1       1    seq
    ......................................................................uggacggagaaUugauaa....................   1       1    seq
    ......................................................................uggacggagaacuAauaa....................   1       1    seq
    ......................................................................uggacggGgaacugauaa....................   2       1    seq
    ......................................................................uggacggagaacugauaa....................   224     0    seq
    ......................................................................Gggacggagaacugauaa....................   1       1    seq
    ......................................................................uggacggagaacugauGa....................   2       1    seq
    ......................................................................ugUacggagaacugauaa....................   3       1    seq
    ......................................................................uggacggagaacugauaU....................   55      1    seq
    ......................................................................Cggacggagaacugauaa....................   2       1    seq
    ......................................................................uggacggagaacugauaG....................   9       1    seq
    ......................................................................uggacggagGacugauaa....................   2       1    seq
    ......................................................................uCgacggagaacugauaa....................   2       1    seq
    ......................................................................uggacggagaacugauaC....................   2       1    seq
    ......................................................................uggGcggagaacugauaa....................   8       1    seq
    ......................................................................uggacggagaacugUuaa....................   1       1    seq
    ......................................................................ugUacggagaacugauaag...................   76      1    seq
    ......................................................................uggacggagaacugauGag...................   12      1    seq
    ......................................................................uggacggagaacugauaaU...................   83      1    seq
    ......................................................................uAgacggagaacugauaag...................   9       1    seq
    ......................................................................uggacggagaacAgauaag...................   6       1    seq
    ......................................................................uggacggagaacuAauaag...................   2       1    seq
    ......................................................................uggacggagaacCgauaag...................   10      1    seq
    ......................................................................uUgacggagaacugauaag...................   104     1    seq
    ......................................................................uggacggagaacugauaag...................   3322    0    seq
    ......................................................................uCgacggagaacugauaag...................   8       1    seq
    ......................................................................uggacggagUacugauaag...................   1       1    seq
    ......................................................................uggacgAagaacugauaag...................   1       1    seq
    ......................................................................uggacggagaacugGuaag...................   19      1    seq
```

```
                          Star                                           Mature
aucauauuugucuuuuaucuuuccuuaucauucucuugcccggccgaauacuauguuauugaaauugcgcuggacggagaacugauaagggcuuauauaagacacgug
......................................................................uggacggagaCcugauaag...................  1     1  seq
......................................................................uggacggCgaacugauaag...................  1     1  seq
......................................................................uggacggagGacugauaag...................  16    1  seq
......................................................................Aggacggagaacugauaag...................  2     1  seq
......................................................................uggGcggagaacugauaag...................  78    1  seq
......................................................................uggacggaAaacugauaag...................  4     1  seq
......................................................................uggacggagaacugauaaC...................  5     1  seq
......................................................................uggacggagaaUugauaag...................  2     1  seq
......................................................................uggacggagCacugauaag...................  2     1  seq
......................................................................uggacggagaacugCuaag...................  1     1  seq
......................................................................uggacggagaacugauaaA...................  147   1  seq
......................................................................uggacgUagaacugauaag...................  3     1  seq
......................................................................uggacggagaacugaGaag...................  1     1  seq
......................................................................uggacAgagaacugauaag...................  6     1  seq
......................................................................ugCacggagaacugauaag...................  3     1  seq
......................................................................Gggacggagaacugauaag...................  14    1  seq
......................................................................uggacggagaacGgauaag...................  1     1  seq
......................................................................uggaUggagaacugauaag...................  5     1  seq
......................................................................uggacggagaacugauaGg...................  13    1  seq
......................................................................ugAacggagaacugauaag...................  5     1  seq
......................................................................uggacggGgaacugauaag...................  14    1  seq
......................................................................uggacggUgaacugauaag...................  1     1  seq
......................................................................uggacCgagaacugauaag...................  1     1  seq
......................................................................uggCcggagaacugauaag...................  1     1  seq
......................................................................uggacggagaacugauUag...................  3     1  seq
......................................................................uggacggagaGcugauaag...................  19    1  seq
......................................................................uggaAggagaacugauaag...................  1     1  seq
......................................................................uggacggagaacugaCaag...................  5     1  seq
......................................................................uggacggagaacugauaUg...................  5     1  seq
......................................................................uggacggagaacugUuaag...................  2     1  seq
......................................................................Cggacggagaacugauaag...................  3     1  seq
......................................................................uCgacggagaacugauaagg..................  111   1  seq
......................................................................uggacggagaacugauaaCg..................  8     1  seq
......................................................................uggacggagaCcugauaagg..................  5     1  seq
......................................................................uggacggagaacugGuaagg..................  199   1  seq
......................................................................uggacggagaacugauUagg..................  17    1  seq
......................................................................uggacggagaGcugauaagg..................  180   1  seq
......................................................................uggacggagaacugauaagA..................  2536  1  seq
......................................................................ugUacggagaacugauaagg..................  859   1  seq
......................................................................uUgacggagaacugauaagg..................  1041  1  seq
......................................................................Cggacggagaacugauaagg..................  139   1  seq
......................................................................uggaAggagaacugauaagg..................  4     1  seq
......................................................................uggacggGgaacugauaagg..................  230   1  seq
......................................................................Gggacggagaacugauaagg..................  152   1  seq
......................................................................uggacggagaacugUuaagg..................  11    1  seq
......................................................................uggacgUagaacugauaagg..................  110   1  seq
......................................................................uggacggCgaacugauaagg..................  10    1  seq
......................................................................uggGcggagaacugauaagg..................  1195  1  seq
......................................................................uggacggagaacugauaagU..................  147   1  seq
......................................................................uggacggagGacugauaagg..................  224   1  seq
......................................................................uggacggagaacCgauaagg..................  116   1  seq
......................................................................uggacggagaacugCuaagg..................  10    1  seq
......................................................................uggacggagaaGugauaagg..................  6     1  seq
......................................................................uggaGggagaacugauaagg..................  5     1  seq
......................................................................uggacgCagaacugauaagg..................  5     1  seq
......................................................................uggacggagaacGgauaagg..................  6     1  seq
......................................................................uggacggagaacugauaaUg..................  17    1  seq
......................................................................uggacggagaacugaAaagg..................  4     1  seq
......................................................................uggacAgagaacugauaagg..................  97    1  seq
......................................................................ugCacggagaacugauaagg..................  37    1  seq
......................................................................uggacggagaacuAauaagg..................  29    1  seq
......................................................................uggCcggagaacugauaagg..................  12    1  seq
......................................................................uggacggagUacugauaagg..................  20    1  seq
......................................................................uggacUgagaacugauaagg..................  18    1  seq
......................................................................uggacggagaacuUauaagg..................  3     1  seq
......................................................................uggacggaAaacugauaagg..................  23    1  seq
......................................................................uggacggagaaAugauaagg..................  3     1  seq
......................................................................uggacgAagaacugauaagg..................  20    1  seq
......................................................................uggacggaCaacugauaagg..................  3     1  seq
......................................................................uggacggagaacugauGagg..................  134   1  seq
```

Star Mature

aucauauuugucuuuuaucuuuccuuaucauucucuugcccggccgaauacuauguuauugaaauugcgcuggacggagaacugauaagggcuuauauaagacacgug			
....uggacggagaacugauaCgg..................	10	1	seq
....Aggacggagaacugauaagg..................	26	1	seq
....uggacggagaacugaCaagg..................	54	1	seq
....uggacggagaUcugauaagg..................	7	1	seq
....ugAacggagaacugauaagg..................	48	1	seq
....uggacggagaacugauaagC..................	78	1	seq
....uggacggagaacugaGaagg..................	16	1	seq
....uAgacggagaacugauaagg..................	109	1	seq
....uggaUggagaacugauaagg..................	65	1	seq
....uggacggagaacugauCagg..................	4	1	seq
....uggacggagaacuCauaagg..................	1	1	seq
....uggUcggagaacugauaagg..................	69	1	seq
....uggacggagaacAgauaagg..................	14	1	seq
....uggacCgagaacugauaagg..................	2	1	seq
....uggacggagaacugauaUgg..................	28	1	seq
....uggacggagCacugauaagg..................	19	1	seq
....uggacggUgaacugauaagg..................	14	1	seq
....uggacggaUaacugauaagg..................	6	1	seq
....uggUcggagaacugauaaggg.................	54	1	seq
....uggacggagaacuAauaaggg.................	15	1	seq
....uggacgAagaacugauaaggg.................	12	1	seq
....uggacggagUacugauaaggg.................	21	1	seq
....uggCcggagaacugauaaggg.................	4	1	seq
....uggacggagaacugauaagAg.................	42	1	seq
....ugUacggagaacugauaaggg.................	309	1	seq
....uAgacggagaacugauaaggg.................	99	1	seq
....uggacggagaacugGuaaggg.................	154	1	seq
....uggacggagaGcugauaaggg.................	127	1	seq
....uggacggagaacCgauaaggg.................	105	1	seq
....uggacggagaacugauaCggg.................	7	1	seq
....uggacggagaacugauaagCg.................	8	1	seq
....uggacggagaacuCauaaggg.................	1	1	seq
....uggacggagaUcugauaaggg.................	5	1	seq
....uggacggagaacugaAaaggg.................	2	1	seq
....uggacggUgaacugauaaggg.................	13	1	seq
....ugCacggagaacugauaaggg.................	18	1	seq
....uggacggCgaacugauaaggg.................	7	1	seq
....uggacggagaacugCuaaggg.................	7	1	seq
....uggacggagaaGugauaaggg.................	3	1	seq
....uggacggagaaAugauaaggg.................	2	1	seq
....uggacAgagaacugauaaggg.................	50	1	seq
....uggacggagaacuUauaaggg.................	6	1	seq
....uggacggagaacugaCaaggg.................	59	1	seq
....uggacggaCaacugauaaggg.................	4	1	seq
....uggacggGgaacugauaaggg.................	155	1	seq
....uggacggagaCcugauaaggg.................	6	1	seq
....uggacggaUaacugauaaggg.................	6	1	seq
....uggaUggagaacugauaaggg.................	37	1	seq
....uggGcggagaacugauaaggg.................	557	1	seq
....uggacggagaacugauaUggg.................	12	1	seq
....uCgacggagaacugauaaggg.................	73	1	seq
....uggacggagaacugaGaaggg.................	17	1	seq
....uggacCgagaacugauaaggg.................	2	1	seq
....uggacggagGacugauaaggg.................	137	1	seq
....ugAacggagaacugauaaggg.................	27	1	seq
....uggacggaAaacugauaaggg.................	13	1	seq
....uggacggagaacugauGaggg.................	88	1	seq
....uggacggagaacugauaaCgg.................	3	1	seq
....uggacggagaacugauaaUgg.................	12	1	seq
....uggacggagaacGgauaaggg.................	3	1	seq
....uggacggagaacugauCaggg.................	6	1	seq
....uUgacggagaacugauaaggg.................	694	1	seq
....uggacggagaacugauaagUg.................	21	1	seq
....uggaAggagaacugauaaggg.................	5	1	seq
....uggacggagaacugauUaggg.................	13	1	seq
....uggacggagaacAgauaaggg.................	10	1	seq
....uggacggagCacugauaaggg.................	6	1	seq
....uggaGggagaacugauaaggg.................	4	1	seq
....uggacggagaacugUuaaggg.................	13	1	seq
....uggacgUagaacugauaaggg.................	75	1	seq

```
                  Star                                          Mature
aucauauuugucuuuuaucuuuccuuaucauucucuugcccggccgaauacuauguuauugaaauugcgcuggacggagaacugauaagggcuuauauaagacacgug
......................................................................uggacUgagaacugauaaggg.................  11   1   seq
......................................................................uggacgCagaacugauaaggg.................  2    1   seq
......................................................................uggacggagGacugauaagggc................  35   1   seq
......................................................................uggacggagaacugauaagAgc................  5    1   seq
......................................................................uggacggagaacuUauaagggc................  1    1   seq
......................................................................uggacggagaacGgauaagggc................  3    1   seq
......................................................................uggacggagaacuAauaagggc................  4    1   seq
......................................................................uggacgAagaacugauaagggc................  4    1   seq
......................................................................uggacggagCacugauaagggc................  2    1   seq
......................................................................uggacggagaacugauGagggc................  25   1   seq
......................................................................uggacggagaacugaCaagggc................  8    1   seq
......................................................................uggacggagaUcugauaagggc................  1    1   seq
......................................................................uggUcggagaacugauaagggc................  11   1   seq
......................................................................uggacggagaacugaGaagggc................  2    1   seq
......................................................................uggacggagaacAgauaagggc................  6    1   seq
......................................................................uggacggagaGcugauaagggc................  40   1   seq
......................................................................ugCacggagaacugauaagggc................  5    1   seq
......................................................................uggacggCgaacugauaagggc................  2    1   seq
......................................................................uggacUgagaacugauaagggc................  3    1   seq
......................................................................uggacggagaacugauaagUgc................  11   1   seq
......................................................................uggCcggagaacugauaagggc................  4    1   seq
......................................................................uggGcggagaacugauaagggc................  127  1   seq
......................................................................uggacggaAaacugauaagggc................  8    1   seq
......................................................................uggacggagaacugCuaagggc................  2    1   seq
......................................................................uggacggagaacugauaUgggc................  3    1   seq
......................................................................uggacggaUaacugauaagggc................  5    1   seq
......................................................................uggacggagaacuCauaagggc................  1    1   seq
......................................................................uggacgUagaacugauaagggc................  19   1   seq
......................................................................uggacggagaacugauaaUggc................  5    1   seq
......................................................................ugAacggagaacugauaagggc................  9    1   seq
......................................................................uggacggagUacugauaagggc................  4    1   seq
......................................................................uggacggagaacugauUagggc................  1    1   seq
......................................................................uggacggagaacugGuaagggc................  32   1   seq
......................................................................uggaUggagaacugauaagggc................  12   1   seq
......................................................................uggacggUgaacugauaagggc................  4    1   seq
......................................................................uggacggagaacugUuaagggc................  6    1   seq
......................................................................uggacggagaacCgauaagggc................  10   1   seq
......................................................................uggacggagaacugauaaCggc................  2    1   seq
......................................................................uggacggagaacugauCagggc................  1    1   seq
......................................................................uggacggagaaGugauaagggc................  1    1   seq
......................................................................uggaGggagaacugauaagggc................  1    1   seq
......................................................................ugUacggagaacugauaagggc................  63   1   seq
......................................................................uggacAgagaacugauaagggc................  10   1   seq
......................................................................uggacggGgaacugauaagggc................  26   1   seq
......................................................................uggacgCagaacugauaagggcu...............  1    1   seq
......................................................................uggacggagaacugaCaagggcu...............  1    1   seq
......................................................................uggacggagaacugaGaagggcu...............  1    1   seq
......................................................................uggacggagaacCgauaagggcu...............  1    1   seq
......................................................................uggacgAagaacugauaagggcu...............  3    1   seq
......................................................................uggacggGgaacugauaagggcu...............  6    1   seq
......................................................................uggacggagaGcugauaagggcu...............  5    1   seq
......................................................................uggUcggagaacugauaagggcu...............  2    1   seq
......................................................................ugAacggagaacugauaagggcu...............  1    1   seq
......................................................................uggacggagaacugauaagUgcu...............  1    1   seq
......................................................................uggGcggagaacugauaagggcu...............  14   1   seq
......................................................................uggacggagaacugGuaagggcu...............  2    1   seq
......................................................................uggacAgagaacugauaagggcu...............  5    1   seq
......................................................................uggacggagaaAugauaagggcu...............  1    1   seq
......................................................................uggacggagaacugauaagAgcu...............  1    1   seq
......................................................................uggacggagaacugauGagggcu...............  1    1   seq
......................................................................uggacggCgaacugauaagggcu...............  1    1   seq
......................................................................uggacggagUacugauaagggcu...............  1    1   seq
......................................................................uggacggagGacugauaagggcu...............  5    1   seq
......................................................................uggacgUagaacugauaagggcu...............  4    1   seq
......................................................................ugUacggagaacugauaagggcu...............  12   1   seq
......................................................................uggGcggagaacugauaagggcuu..............  2    1   seq
......................................................................ugUacggagaacugauaagggcuu..............  1    1   seq
.......................................................................Ugacggagaacugauaag...................  1    1   seq
.......................................................................ggacggagaacugauaagg..................  2    0   seq
.......................................................................Ugacggagaacugauaagg..................  5    1   seq
```

Star Mature

```
aucauauuugucuuuuaucuuuccuuaucauucucuugcccggccgaauacuauguuauugaaauugcgcuggacggagaacugauaagggcuuauauaagacacgug
.......................................................................Ugacggagaacugauaaggg.................   2   1   seq
........................................................................gacggagaacugauaagg..................   2   0   seq
........................................................................Uacggagaacugauaaggg.................   1   1   seq
........................................................................gacUgagaacugauaagggcu...............   1   1   seq
........................................................................gacggagaacuUauaagggcu...............   1   1   seq
.........................................................................acggagaacugauaaggg.................   2   0   seq
.........................................................................acggagaacugauaaggA.................   1   1   seq
.........................................................................acggagaacugauaagggcA...............   2   1   seq
.........................................................................acggagaacugauaagggcCu..............   1   1   seq
.........................................................................acggagaacugauaagggcuu..............   6   0   seq
.........................................................................acggagaacugauaagggcuA..............   2   1   seq
```
