# Supplementary material for: Identification and Characterization of MicroRNAs from Longitudinal Muscle and Respiratory Tree in Sea Cucumber (Apostichopus japonicus) Using High-Throughput Sequencing
Source: PLoS One. 2015 Aug 5;10(8):e0134899. doi: 10.1371/journal.pone.0134899 (PMC4526669; doi:10.1371/journal.pone.0134899)
Supplement: S2 File — (ZIP) [file pone.0134899.s003.zip › S2 File/The secondary structures of the novel miRNAs in RPT/Scaffold912_2075.pdf]

Provisional ID	:	Scaffold912_2075
Score total	:	3352.2
Score for star read(s)	:	3.9
Score for read counts	:	3344.6
Score for mfe	:	2.1
Score for randfold	:	1.6
Score for cons. seed	:	
Total read count	:	6572
Mature read count	:	6371
Loop read count	:	0
Star read count	:	201

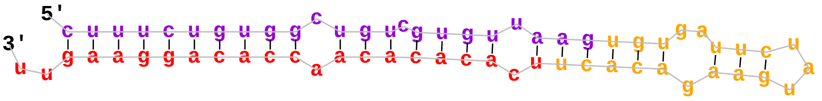

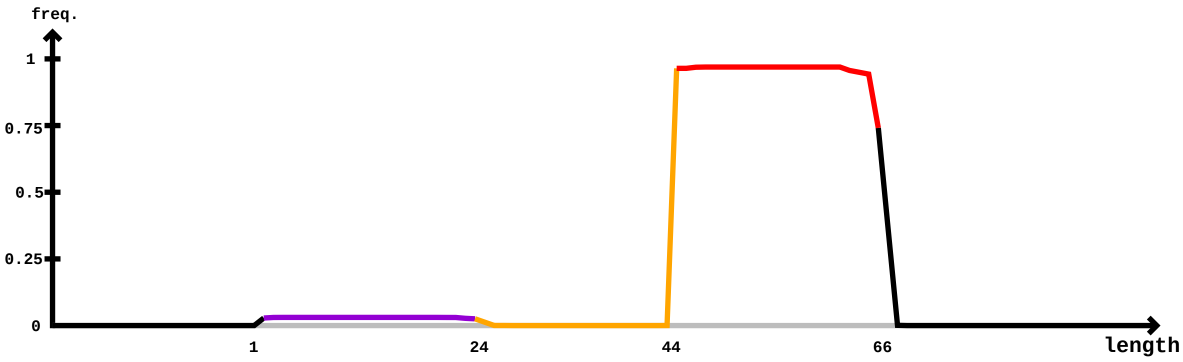

Star **Mature**

```
5'- aagagaggcuacuugcggaccuuucuguggcugucguguuaagugugauucuaugaagacacuucacacacaaccacaggaaguuuugcacgguagccaugacaacgcauccg   -3'   obs
    aagagaggcuacuugcggaccuuucuguggcugucguguuaagugugauucuaugaagacacuucacacacaaccacaggaaguuuugcacgguagccaugacaacgcauccg         exp
    ......(((((((((((((.((((((((((.(((.((((.((((((..(((...))).)))))).))))))).)))))))))).)))))).)))))))...............    reads   mm   sample
    ....................cuuucuguggcugucgugu..........................................................................    1       0    seq
    ....................cuuucuguggcugucguguu.........................................................................    1       0    seq
    ....................cuuucuguggcugucguguua........................................................................    11      0    seq
    ....................cuuuUguggcugucguguua........................................................................    2       1    seq
    ....................cuuucuguggUugucguguua........................................................................    2       1    seq
    ....................cuCucuguggcugucguguua........................................................................    1       1    seq
    ....................cuuucAguggcugucguguua........................................................................    1       1    seq
    ....................cGuucuguggcugucguguua........................................................................    1       1    seq
    ....................cuuucuguggcugucguguuaU.......................................................................    1       1    seq
    ....................cuuucuguggcugucguguuaa.......................................................................    8       0    seq
    ....................cuuucuguggUugucguguuaa.......................................................................    3       1    seq
    ....................cGuucuguggcugucguguuaag......................................................................    1       1    seq
    ....................cuuuUguggcugucguguuaag......................................................................    5       1    seq
    ....................cCuucuguggcugucguguuaag......................................................................    3       1    seq
    ....................cuuucuguggcugucguguuaGg......................................................................    6       1    seq
    ....................cuuucuguggUugucguguuaag......................................................................    15      1    seq
    ....................cuuucuguggcugucgCguuaag......................................................................    1       1    seq
    ....................cuuucuguggcugucguguuaaA......................................................................    3       1    seq
    ....................cuuucuguggcugucgugCuaag......................................................................    1       1    seq
    ....................cuuGcuguggcugucguguuaag......................................................................    2       1    seq
    ....................cuuucuguggcugAcguguuaag......................................................................    1       1    seq
    ....................cuuucuguggcugucguguuaag......................................................................    100     0    seq
    ....................cuCucuguggcugucguguuaag......................................................................    1       1    seq
    ....................cAuucuguggcugucguguuaag......................................................................    1       1    seq
    ....................cuuucuguggcugucAuguuaag......................................................................    1       1    seq
    ....................cuuCcuguggcugucguguuaag......................................................................    1       1    seq
    ....................cuuucuguggcuguGguguuaag......................................................................    1       1    seq
    ....................cuuucuguggcugucguguuaagA.....................................................................    1       1    seq
    ....................cuuuUguggcugucguguuaagu.....................................................................    1       1    seq
    ....................cuuucuguggcugucguguuaagu.....................................................................    3       0    seq
    ....................cuuucuguggUugucguguuaagu.....................................................................    3       1    seq
    ....................cuGucuguggcugucguguuaagu.....................................................................    1       1    seq
    ....................cuuucuguggcugucguguuaagugu...................................................................    2       0    seq
```

Star **Mature**

```
aagagaggcuacuugcggaccuuucuguggcugucguguuaagugugauucuaugaagacacuucacacacaaccacaggaaguuuugcacgguagccaugacaacgcauccg
.....................uCucuguggcugucguguua........................................................................ 1 1 seq
.....................uuucuguggcugucguguua........................................................................ 2 0 seq
.....................uuucuguggcugucguguuaaU...................................................................... 2 1 seq
.....................uGucuguggcugucguguuaag...................................................................... 1 1 seq
.....................uuuUuguggcugucguguuaag...................................................................... 1 1 seq
.....................uuucuguggcugucguguuaag...................................................................... 5 0 seq
.....................uuucuguggcugucgGguuaag...................................................................... 1 1 seq
.....................uuucuguggcugucguguuaagu..................................................................... 1 0 seq
......................uuUuguggcugucguguuaagu..................................................................... 1 1 seq
..............................................................uucGcacacaaccacaggaagu............................. 1 1 seq
...............................................................ucacacacaaccacagga................................ 76 0 seq
...............................................................ucaGacacaaccacagga................................ 1 1 seq
...............................................................ucacacacaacUacagga................................ 1 1 seq
...............................................................ucacacacCaccacagga................................ 1 1 seq
...............................................................ucacacacaaccacaggG................................ 1 1 seq
...............................................................ucacacUcaaccacagga................................ 1 1 seq
...............................................................ucUcacacaaccacagga................................ 1 1 seq
...............................................................uGacacacaaccacagga................................ 1 1 seq
...............................................................Ccacacacaaccacagga................................ 1 1 seq
...............................................................ucacacacaaccacaggGa............................... 1 1 seq
...............................................................ucacacacaaccacaggaU............................... 1 1 seq
...............................................................uUacacacaaccacaggaa............................... 1 1 seq
...............................................................ucacacacCaccacaggaa............................... 1 1 seq
...............................................................ucaGacacaaccacaggaa............................... 1 1 seq
...............................................................ucacacacaacUacaggaa............................... 1 1 seq
...............................................................ucacacacaaccacaggaa............................... 39 0 seq
...............................................................ucacacacCaccacaggaag.............................. 4 1 seq
...............................................................ucacacacaaccacaggaaU.............................. 17 1 seq
...............................................................Gcacacacaaccacaggaag.............................. 2 1 seq
...............................................................ucacacacaaccacaggaaC.............................. 3 1 seq
...............................................................Acacacacaaccacaggaag.............................. 1 1 seq
...............................................................uGacacacaaccacaggaag.............................. 2 1 seq
...............................................................ucacacacaaccaUaggaag.............................. 1 1 seq
...............................................................ucacacacaaccacagAaag.............................. 1 1 seq
...............................................................uUacacacaaccacaggaag.............................. 2 1 seq
...............................................................ucacacacaaccacGggaag.............................. 2 1 seq
...............................................................ucaGacacaaccacaggaag.............................. 5 1 seq
...............................................................ucacacacaaccacaggaaA.............................. 3 1 seq
...............................................................ucUcacacaaccacaggaag.............................. 1 1 seq
...............................................................ucacacacaaccacaggCag.............................. 1 1 seq
...............................................................ucacacGcaaccacaggaagu............................. 75 1 seq
...............................................................ucacGcacaaccacaggaagu............................. 71 1 seq
...............................................................ucacaAacaaccacaggaagu............................. 1 1 seq
...............................................................ucacacacaaccacaAgaagu............................. 17 1 seq
...............................................................ucacacacCaccacaggaagu............................. 109 1 seq
...............................................................ucacacacaaccCcaggaagu............................. 4 1 seq
...............................................................ucacaUacaaccacaggaagu............................. 21 1 seq
...............................................................ucacacacaaccacagAaagu............................. 12 1 seq
...............................................................ucacacacaaccacCggaagu............................. 4 1 seq
...............................................................ucacacacaaccacUggaagu............................. 14 1 seq
...............................................................ucacacacaaccacaggGagu............................. 63 1 seq
...............................................................ucacacCcaaccacaggaagu............................. 2 1 seq
...............................................................ucacacaGaaccacaggaagu............................. 2 1 seq
...............................................................ucacacacaacUacaggaagu............................. 30 1 seq
...............................................................uGacacacaaccacaggaagu............................. 49 1 seq
...............................................................ucaGacacaaccacaggaagu............................. 89 1 seq
...............................................................ucacacacaaAcacaggaagu............................. 2 1 seq
...............................................................ucacacacaaGcacaggaagu............................. 3 1 seq
...............................................................ucacacacaacAacaggaagu............................. 2 1 seq
...............................................................ucacacacaaccaAaggaagu............................. 4 1 seq
...............................................................uUacacacaaccacaggaagu............................. 149 1 seq
...............................................................ucacacacaaccacaggCagu............................. 9 1 seq
...............................................................ucacacacaaccacaggaaUu............................. 25 1 seq
...............................................................ucacacacaCccacaggaagu............................. 3 1 seq
...............................................................ucacacacaaccacGggaagu............................. 84 1 seq
...............................................................ucacacacaaccacaggaaCu............................. 1 1 seq
...............................................................ucacacacUaccacaggaagu............................. 9 1 seq
...............................................................ucacacacaaccacaggaUgu............................. 2 1 seq
...............................................................ucGcacacaaccacaggaagu............................. 76 1 seq
...............................................................ucaAacacaaccacaggaagu............................. 4 1 seq
```

```
                    Star                                         Mature
aagagaggcuacuugcggaccuuucuguggcugucguguuaagugugauucuaugaagacacuucacacacaaccacaggaaguuuugcacgguagccaugacaacgcauccg
...............................................................ucacacaUaaccacaggaagu.............................    20    1    seq
...............................................................ucacacacGaccacaggaagu.............................    73    1    seq
...............................................................ucacacacaUccacaggaagu.............................    8     1    seq
...............................................................ucaUacacaaccacaggaagu.............................    26    1    seq
...............................................................uAacacacaaccacaggaagu.............................    1     1    seq
...............................................................ucacacacaaccUcaggaagu.............................    2     1    seq
...............................................................ucacacacaaccacaggaCgu.............................    2     1    seq
...............................................................ucacacacaaccacaCgaagu.............................    1     1    seq
...............................................................ucacaGacaaccacaggaagu.............................    1     1    seq
...............................................................ucacacacaaccacaggaaAu.............................    13    1    seq
...............................................................ucacacacaaccacaUgaagu.............................    9     1    seq
...............................................................ucacacacaaccacaggUagu.............................    1     1    seq
...............................................................ucacacacaGccacaggaagu.............................    60    1    seq
...............................................................ucacacacaaccaUaggaagu.............................    8     1    seq
...............................................................ucUcacacaaccacaggaagu.............................    42    1    seq
...............................................................ucCcacacaaccacaggaagu.............................    2     1    seq
...............................................................ucacacacaaccacaggaGgu.............................    66    1    seq
...............................................................ucacacaAaaccacaggaagu.............................    1     1    seq
...............................................................ucacacacaaUcacaggaagu.............................    16    1    seq
...............................................................ucacacUcaaccacaggaagu.............................    8     1    seq
...............................................................ucacUcacaaccacaggaagu.............................    7     1    seq
...............................................................ucacCcacaaccacaggaagu.............................    10    1    seq
...............................................................ucacacacaaccacagUaagu.............................    2     1    seq
...............................................................ucacGcacaaccacaggaaguu...........................    270   1    seq
...............................................................ucacacacaaccacagAaaguu...........................    42    1    seq
...............................................................ucacacacaaccacaggaUguu...........................    8     1    seq
...............................................................ucacacacaaccacUggaaguu...........................    43    1    seq
...............................................................ucacacGcaaccacaggaaguu...........................    329   1    seq
...............................................................ucacacacaaccacaAgaaguu...........................    84    1    seq
...............................................................ucacacacCaccacaggaaguu...........................    550   1    seq
...............................................................ucacacacaaccGcaggaaguu...........................    196   1    seq
...............................................................ucacacacaGccacaggaaguu...........................    235   1    seq
...............................................................ucacacacaaccacaCgaaguu...........................    8     1    seq
...............................................................ucacacaAaaccacaggaaguu...........................    2     1    seq
...............................................................ucacacacaaccUcaggaaguu...........................    24    1    seq
...............................................................ucacacacaCccacaggaaguu...........................    11    1    seq
...............................................................ucacacacaacGacaggaaguu...........................    11    1    seq
...............................................................ucacCcacaaccacaggaaguu...........................    31    1    seq
...............................................................ucacacacaaAcacaggaaguu...........................    11    1    seq
...............................................................ucaUacacaaccacaggaaguu...........................    100   1    seq
...............................................................ucacacacaaccacagCaaguu...........................    3     1    seq
...............................................................ucacacacaaccacaggaGguu...........................    222   1    seq
...............................................................ucacacacaaccacaggaaUuu...........................    134   1    seq
...............................................................ucacacacaaccacagUaaguu...........................    9     1    seq
...............................................................ucacacaGaaccacaggaaguu...........................    14    1    seq
...............................................................ucacacacaaccCcaggaaguu...........................    18    1    seq
...............................................................ucacacacaaccaAaggaaguu...........................    26    1    seq
...............................................................ucacacaUaaccacaggaaguu...........................    74    1    seq
...............................................................ucacacacaacUacaggaaguu...........................    113   1    seq
...............................................................ucacacacaaGcacaggaaguu...........................    15    1    seq
...............................................................ucacaGacaaccacaggaaguu...........................    17    1    seq
...............................................................ucacacacaaccacaggaaCuu...........................    14    1    seq
...............................................................ucacacacaaccacGgaaguu...........................    287   1    seq
...............................................................ucacacCcaaccacaggaaguu...........................    15    1    seq
...............................................................ucacacacaaccaUaggaaguu...........................    57    1    seq
...............................................................ucacacacaaccaGaggaaguu...........................    13    1    seq
...............................................................ucUcacacaaccacaggaaguu...........................    190   1    seq
...............................................................ucacacacUaccacaggaaguu...........................    38    1    seq
...............................................................ucacacacaaccacaggaaAuu...........................    29    1    seq
...............................................................ucacacacaaccacaggCaguu...........................    17    1    seq
...............................................................ucCcacacaaccacaggaaguu...........................    20    1    seq
...............................................................ucacacacaaccacaggGaguu...........................    250   1    seq
...............................................................ucacacacaaccacCggaaguu...........................    14    1    seq
...............................................................ucacacacaaccacaUgaaguu...........................    40    1    seq
...............................................................ucacacacaaUcacaggaaguu...........................    54    1    seq
...............................................................ucacaAacaaccacaggaaguu...........................    8     1    seq
...............................................................ucacUcacaaccacaggaaguu...........................    36    1    seq
...............................................................ucacacacGaccacaggaaguu...........................    276   1    seq
...............................................................ucacaUacaaccacaggaaguu...........................    87    1    seq
...............................................................ucacacacaaccacaggaCguu...........................    17    1    seq
```

Star **Mature**

```
aagagaggcuacuugcggaccuuucuguggcugucguguuaagugugauucuaugaagacacuucacacacaaccacaggaaguuuugcacgguagccaugacaacgcauccg
...............................................................ucacacacaacAacaggaaguu............................    4    1    seq
...............................................................ucaAacacaaccacaggaaguu............................    9    1    seq
...............................................................ucacacacaaccacaggUaguu............................    12   1    seq
...............................................................ucGcacacaaccacaggaaguu............................    237  1    seq
...............................................................ucaGacacaaccacaggaaguu............................    344  1    seq
...............................................................ucacacacaUccacaggaaguu............................    23   1    seq
...............................................................ucacacUcaaccacaggaaguu............................    29   1    seq
...............................................................ucacacacaaccGcaggaaguuu...........................    9    1    seq
...............................................................ucacacacaaccacaAgaaguuu...........................    1    1    seq
...............................................................ucacUcacaaccacaggaaguuu...........................    1    1    seq
...............................................................ucacacacGaccacaggaaguuu...........................    6    1    seq
...............................................................ucacacacaaccacaggGaguuu...........................    6    1    seq
...............................................................ucacacacaaUcacaggaaguuu...........................    3    1    seq
...............................................................ucCcacacaaccacaggaaguuu...........................    1    1    seq
...............................................................ucUcacacaaccacaggaaguuu...........................    5    1    seq
...............................................................ucacacacaGccacaggaaguuu...........................    3    1    seq
...............................................................ucacacacaacUacaggaaguuu...........................    1    1    seq
...............................................................ucacacacaaccacagAaaguuu...........................    3    1    seq
...............................................................ucacacCcaaccacaggaaguuu...........................    1    1    seq
...............................................................ucacacGcaaccacaggaaguuu...........................    7    1    seq
...............................................................ucacacacaaccacaggaGguuu...........................    8    1    seq
...............................................................ucacacacaaccaUaggaaguuu...........................    3    1    seq
...............................................................ucGcacacaaccacaggaaguuu...........................    3    1    seq
...............................................................ucaUacacaaccacaggaaguuu...........................    3    1    seq
...............................................................ucaGacacaaccacaggaaguuu...........................    13   1    seq
...............................................................ucacacacaaccacaggaUguuu...........................    1    1    seq
...............................................................ucacGcacaaccacaggaaguuu...........................    9    1    seq
...............................................................ucacacacaaGcacaggaaguuu...........................    1    1    seq
...............................................................ucacacaUaaccacaggaaguuu...........................    5    1    seq
...............................................................ucacaUacaaccacaggaaguuu...........................    2    1    seq
...............................................................ucacaAacaaccacaggaaguuu...........................    1    1    seq
...............................................................ucacacacUaccacaggaaguuu...........................    1    1    seq
...............................................................ucacacacCaccacaggaaguuu...........................    15   1    seq
...............................................................ucacacacaUccacaggaaguuu...........................    2    1    seq
...............................................................ucacaGacaaccacaggaaguuu...........................    1    1    seq
...............................................................ucacacacaaccacaggaaUuuu...........................    2    1    seq
...............................................................ucaAacacaaccacaggaaguuu...........................    1    1    seq
...............................................................ucacacacaaccacaggUaguuu...........................    1    1    seq
...............................................................ucacacacaaccacGggaaguuu...........................    4    1    seq
...............................................................ucacacacaaccacaggGaguuuu..........................    1    1    seq
...............................................................ucacacacaaccacaggaaAuuuu..........................    1    1    seq
...............................................................ucacacacCaccacaggaaguuuu..........................    2    1    seq
...............................................................ucacacacaaccacGggaaguuuu..........................    2    1    seq
.................................................................Ucacacaaccacaggaagu.............................    9    1    seq
.................................................................acacacaaccacaggaagu.............................    2    0    seq
.................................................................acaGacaaccacaggaaguu............................    1    1    seq
.................................................................Ucacacaaccacaggaaguu............................    15   1    seq
..................................................................cacacaaccacaggaagu.............................    1    0    seq
..................................................................cacacaaccacaggaaguu............................    3    0    seq
..................................................................cacacaaccacaggaaguuu...........................    1    0    seq
```
