# Supplementary material for: Identification and Characterization of MicroRNAs from Longitudinal Muscle and Respiratory Tree in Sea Cucumber (Apostichopus japonicus) Using High-Throughput Sequencing
Source: PLoS One. 2015 Aug 5;10(8):e0134899. doi: 10.1371/journal.pone.0134899 (PMC4526669; doi:10.1371/journal.pone.0134899)
Supplement: S2 File — (ZIP) [file pone.0134899.s003.zip › S2 File/The secondary structures of the novel miRNAs in RPT/Scaffold97_413.pdf]

| | | |
|---|---|---|
| Provisional ID | : | Scaffold97_413 |
| Score total | : | 2 |
| Score for star read(s) | : | -1.3 |
| Score for read counts | : | 0 |
| Score for mfe | : | 1.7 |
| Score for randfold | : | 1.6 |
| Score for cons. seed | : | |
| Total read count | : | 4027 |
| Mature read count | : | 4027 |
| Loop read count | : | 0 |
| Star read count | : | 0 |

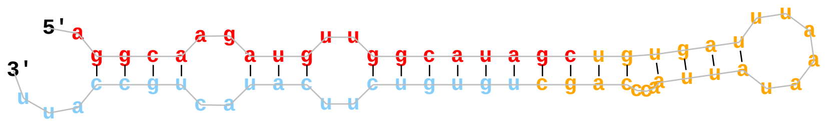

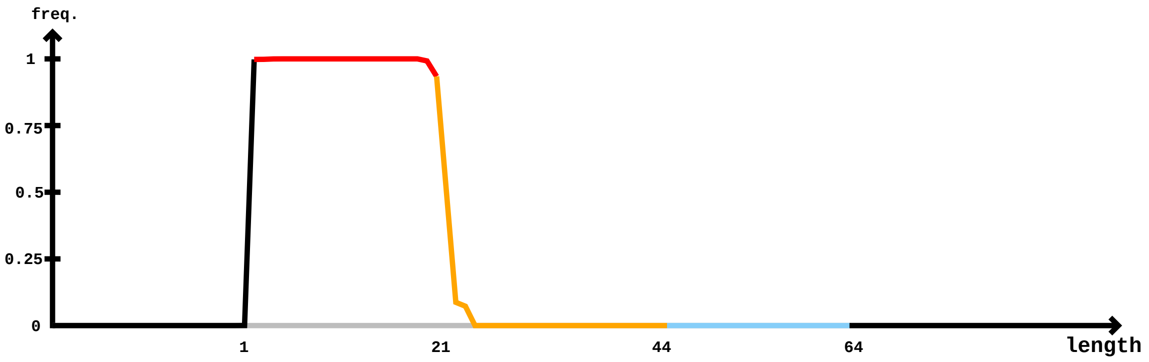


Mature Star

```
5'- guacaaaccaguggagaaaaggcaagauguuggcauagcugugauuuaaauauuaacccagcugugucuucauacugccauuuauucacuugguuguucgcccagaauc  -3'   exp
    .....((((((((((.(((.((((..(((..((((((((((((((......))))...))))))))))..)))..)))).))).))))).)))))((((.....)))).   reads   mm   sample
    ..................aaggcaagauguuggcauagU......................................................................   1       1    seq
    ...................aggcaagauguuggcaua........................................................................   22      0    seq
    ...................aggcaagauguugAcaua........................................................................   1       1    seq
    ...................aggcaagauguuggcauC........................................................................   1       1    seq
    ...................agUcaagauguuggcaua........................................................................   2       1    seq
    ...................aUgcaagauguuggcaua........................................................................   2       1    seq
    ...................aggcaagauguuggcaGa........................................................................   1       1    seq
    ...................aggcaagauguuggcauU........................................................................   2       1    seq
    ...................aggcaagauguuggAauag.......................................................................   1       1    seq
    ...................aggcaagUuguuggcauag.......................................................................   1       1    seq
    ...................aCgcaagauguuggcauag.......................................................................   2       1    seq
    ...................aggcaagauUuuggcauag.......................................................................   1       1    seq
    ...................aggGaagauguuggcauag.......................................................................   3       1    seq
    ...................aggcaagGuguuggcauag.......................................................................   2       1    seq
    ...................aggcaagauguuggcaCag.......................................................................   1       1    seq
    ...................agUcaagauguuggcauag.......................................................................   6       1    seq
    ...................aUgcaagauguuggcauag.......................................................................   10      1    seq
    ...................aggcaaUauguuggcauag.......................................................................   1       1    seq
    ...................aggcaagauguuggcauag.......................................................................   192     0    seq
    ...................aggcaagauguuggcauaA.......................................................................   10      1    seq
    ...................aggcaagauguuggcGuag.......................................................................   1       1    seq
    ...................aggcaagaCguuggcauag.......................................................................   1       1    seq
    ...................aggcaagauguuggcauaC.......................................................................   1       1    seq
    ...................aggcaagauguCggcauag.......................................................................   1       1    seq
    ...................aggGaagauguuggcauagc......................................................................   65      1    seq
    ...................aggcaagauguugAcauagc......................................................................   9       1    seq
    ...................aAgcaagauguuggcauagc......................................................................   12      1    seq
    ...................aggcaCgauguuggcauagc......................................................................   1       1    seq
    ...................aggcaaAauguuggcauagc......................................................................   3       1    seq
    ...................aggcaagauguuAgcauagc......................................................................   7       1    seq
    ...................aggcGagauguuggcauagc......................................................................   28      1    seq
    ...................aggcaGgauguuggcauagc......................................................................   27      1    seq
    ...................aggcaagauguuggcauGgc......................................................................   10      1    seq
    ...................aggcaagauguuggcauaCc......................................................................   1       1    seq
```

```
                   Mature                                   Star
guacaaaccaguggagaaaaggcaagauguuggcauagcugugauuuaaauauuaacccagcugugucuucauacugccauuuauucacuugguuguucgcccagaauc
...................agCcaagauguuggcauagc......................................................................    4     1    seq
...................aggcaagaugAuggcauagc......................................................................    2     1    seq
...................agAcaagauguuggcauagc......................................................................    8     1    seq
...................aggcaagauguuggUauagc......................................................................    8     1    seq
...................aggcaagauguGggcauagc......................................................................    2     1    seq
...................aggcaagauguuggcGuagc......................................................................    19    1    seq
...................aCgcaagauguuggcauagc......................................................................    16    1    seq
...................aggcaagauguuggcauagA......................................................................    113   1    seq
...................aggcaagauguuggcauaUc......................................................................    3     1    seq
...................aggcaagCuguuggcauagc......................................................................    1     1    seq
...................aggcaagauguAggcauagc......................................................................    8     1    seq
...................aggcaagauguuggcaCagc......................................................................    15    1    seq
...................aggcaaUauguuggcauagc......................................................................    11    1    seq
...................aggcaUgauguuggcauagc......................................................................    7     1    seq
...................agUcaagauguuggcauagc......................................................................    125   1    seq
...................aggcCagauguuggcauagc......................................................................    3     1    seq
...................aggcaagauguuggcauagG......................................................................    5     1    seq
...................aggcaagGuguuggcauagc......................................................................    22    1    seq
...................aggcaagaAguuggcauagc......................................................................    1     1    seq
...................aggcaagauguuggcauagU......................................................................    648   1    seq
...................aggcaagauguuggcaGagc......................................................................    2     1    seq
...................aggcaagauguuggcaAagc......................................................................    2     1    seq
...................aggcaagaCguuggcauagc......................................................................    22    1    seq
...................aUgcaagauguuggcauagc......................................................................    134   1    seq
...................aggcaagauguuggcauCgc......................................................................    2     1    seq
...................aggUaagauguuggcauagc......................................................................    5     1    seq
...................aggcaagauguuggcCuagc......................................................................    4     1    seq
...................aggcaagauguuggcauUgc......................................................................    2     1    seq
...................aggcaagauguuUgcauagc......................................................................    1     1    seq
...................aggcaagauguuggAauagc......................................................................    5     1    seq
...................aggcaagaugGuggcauagc......................................................................    5     1    seq
...................aggcaagauAuuggcauagc......................................................................    2     1    seq
...................aggcaagauguuUgcauagcu.....................................................................    8     1    seq
...................aggcaagauCuuggcauagcu.....................................................................    1     1    seq
...................aggcaGgauguuggcauagcu.....................................................................    88    1    seq
...................aggcaaUauguuggcauagcu.....................................................................    36    1    seq
...................aggcaagauguugCcauagcu.....................................................................    3     1    seq
...................aggcaagauguuggcauaUcu.....................................................................    11    1    seq
...................aUgcaagauguuggcauagcu.....................................................................    570   1    seq
...................aggcaaAauguuggcauagcu.....................................................................    17    1    seq
...................aggcaagUuguuggcauagcu.....................................................................    14    1    seq
...................aggGaagauguuggcauagcu.....................................................................    114   1    seq
...................aggcaagauguuggcauagAu.....................................................................    5     1    seq
...................aggcaagauguuggGauagcu.....................................................................    5     1    seq
...................aAgcaagauguuggcauagcu.....................................................................    46    1    seq
...................aggcaagauAuuggcauagcu.....................................................................    23    1    seq
...................aggcaagaugGuggcauagcu.....................................................................    22    1    seq
...................aggcaagauguuggcauagUu.....................................................................    32    1    seq
...................aggcaagauguuggcUuagcu.....................................................................    2     1    seq
...................aggcaagauguuggcaCagcu.....................................................................    36    1    seq
...................aggcaagauguuggcGuagcu.....................................................................    75    1    seq
...................aggcaagGuguuggcauagcu.....................................................................    103   1    seq
...................aggcaaCauguuggcauagcu.....................................................................    6     1    seq
...................aggcaagauguuggAauagcu.....................................................................    7     1    seq
...................aggcaagauguuCgcauagcu.....................................................................    1     1    seq
...................aggcaagauguGggcauagcu.....................................................................    18    1    seq
...................aggcaagaCguuggcauagcu.....................................................................    56    1    seq
...................agAcaagauguuggcauagcu.....................................................................    35    1    seq
...................aggcaagauguuAgcauagcu.....................................................................    37    1    seq
...................agCcaagauguuggcauagcu.....................................................................    20    1    seq
...................aggUaagauguuggcauagcu.....................................................................    24    1    seq
...................aggcaagauguuggcauaCcu.....................................................................    6     1    seq
...................aggcaagaugAuggcauagcu.....................................................................    8     1    seq
...................aggcaagaGguuggcauagcu.....................................................................    10    1    seq
...................aggcaagauguuggcauGgcu.....................................................................    42    1    seq
...................aggcaUgauguuggcauagcu.....................................................................    12    1    seq
...................aggcaagCuguuggcauagcu.....................................................................    2     1    seq
...................aggcaCgauguuggcauagcu.....................................................................    6     1    seq
...................aggcGagauguuggcauagcu.....................................................................    112   1    seq
...................aggcaagaAguuggcauagcu.....................................................................    6     1    seq
```

```
                   Mature                                    Star
guacaaaccaguggagaaaaggcaagauguuggcauagcugugauuuaaauauuaacccagcugugucuucauacugccauuuauucacuugguuguucgcccagaauc
...................aggcaagauguugUcauagcu.....................................................................     9     1     seq
...................aggcaagauguuggUauagcu.....................................................................     39    1     seq
...................agUcaagauguuggcauagcu.....................................................................     202   1     seq
...................aggcaagauguuggcauUgcu.....................................................................     8     1     seq
...................aggcaagauguugAcauagcu.....................................................................     22    1     seq
...................aggcaagauguuggcauCgcu.....................................................................     4     1     seq
...................aggcaagauUuuggcauagcu.....................................................................     7     1     seq
...................aggcaagauguuggcCuagcu.....................................................................     14    1     seq
...................aggcCagauguuggcauagcu.....................................................................     3     1     seq
...................aggcaagauguAggcauagcu.....................................................................     14    1     seq
...................aggcaagauguuggcauagGu.....................................................................     8     1     seq
...................aggcaagauguuggcaGagcu.....................................................................     5     1     seq
...................aggcUagauguuggcauagcu.....................................................................     12    1     seq
...................aCgcaagauguuggcauagcu.....................................................................     66    1     seq
...................aggcaagauAuuggcauagcug...................................................................     3     1     seq
...................aggcaagauguuggcauagAug...................................................................     2     1     seq
...................aggcGagauguuggcauagcug...................................................................     3     1     seq
...................aggcaagauguuggcauaUcug...................................................................     1     1     seq
...................aggcaagauguuggcaCagcug...................................................................     2     1     seq
...................aggcaagauguuggcauagGug...................................................................     1     1     seq
...................aggcaGgauguuggcauagcug...................................................................     4     1     seq
...................agAcaagauguuggcauagcug...................................................................     1     1     seq
...................aggcaaUauguuggcauagcug...................................................................     2     1     seq
...................agUcaagauguuggcauagcug...................................................................     5     1     seq
...................aggcaagauguuAgcauagcug...................................................................     2     1     seq
...................aggcaagauguAggcauagcug...................................................................     1     1     seq
...................aggGaagauguuggcauagcug...................................................................     3     1     seq
...................aggcaagauguuggcauGgcug...................................................................     4     1     seq
...................aggcaagauguuggcGuagcug...................................................................     5     1     seq
...................aggcaagauguuggcauaCcug...................................................................     1     1     seq
...................aggcaagCuguuggcauagcug...................................................................     1     1     seq
...................aggcaagaCguuggcauagcug...................................................................     3     1     seq
...................aggcaagauguuggGauagcug...................................................................     1     1     seq
...................aggcaagGuguuggcauagcug...................................................................     2     1     seq
...................aggUaagauguuggcauagcug...................................................................     1     1     seq
...................aggcaagaugGuggcauagcug...................................................................     3     1     seq
...................aggcaCgauguuggcauagcug...................................................................     1     1     seq
...................aggcaUgauguuggcauagcug...................................................................     1     1     seq
...................aggcaagauguGggcauagcug...................................................................     1     1     seq
...................aggcaagauguuggAauagcug...................................................................     1     1     seq
...................aggcaaAauguuggcauagcug...................................................................     1     1     seq
...................aggcaagauguuggUauagcug...................................................................     3     1     seq
...................aggcaagauguuggcGuagcugu..................................................................     43    1     seq
...................aggcaagauguuggcaAagcugu..................................................................     2     1     seq
...................aggcaaUauguuggcauagcugu..................................................................     22    1     seq
...................aggcaagaugAuggcauagcugu..................................................................     1     1     seq
...................aggcaagauguuggcaCagcugu..................................................................     23    1     seq
...................aggcaagaugGuggcauagcugu..................................................................     11    1     seq
...................aggcaagauguuggUauagcugu..................................................................     15    1     seq
...................aggcaaCauguuggcauagcugu..................................................................     2     1     seq
...................aggcaagaAguuggcauagcugu..................................................................     3     1     seq
...................aggcaagaCguuggcauagcugu..................................................................     26    1     seq
...................aggcaagauguugUcauagcugu..................................................................     3     1     seq
...................aggcaagauguugAcauagcugu..................................................................     11    1     seq
...................aggcaagauguuggAauagcugu..................................................................     6     1     seq
...................aggcaagauguugCcauagcugu..................................................................     1     1     seq
...................aggcaagauCuuggcauagcugu..................................................................     2     1     seq
...................aggcaagauguuAgcauagcugu..................................................................     14    1     seq
...................aggcaagauAuuggcauagcugu..................................................................     12    1     seq
...................aggcaagaGguuggcauagcugu..................................................................     4     1     seq
...................aggcaagauguuggcCuagcugu..................................................................     3     1     seq
...................aggcaagauguuggGauagcugu..................................................................     1     1     seq
...................aggcaagauguuggcaGagcugu..................................................................     2     1     seq
...................aggcaagauguGggcauagcugu..................................................................     9     1     seq
...................aggcaagauguuUgcauagcugu..................................................................     1     1     seq
...................aggcaagCuguuggcauagcugu..................................................................     1     1     seq
...................aggcaagGuguuggcauagcugu..................................................................     50    1     seq
...................aggcaagauguAggcauagcugu..................................................................     8     1     seq
...................aggcaagauguuggcUuagcugu..................................................................     1     1     seq
...................aggcaaAauguuggcauagcugu..................................................................     9     1     seq
```

```
                   Mature                                      Star
guacaaaccaguggagaaaaggcaagauguuggcauagcugugauuuaaauauuaacccagcugugucuucauacugccauuuauucacuugguuguucgcccagaauc
...................aggcaagauUuuggcauagcugu...................................................................   6   1   seq
....................Agcaagauguuggcauagcu.....................................................................   1   1   seq
.....................gcaagauguuggcauagcC.....................................................................   1   1   seq
.....................gcaagauguuggcauagcu.....................................................................   5   0   seq
......................caagauguuggcauagcu.....................................................................   1   0   seq
```
